# Energy-transfer photoproximity labelling in live cells using an organic cofactor

**DOI:** 10.1038/s41557-025-01931-8

**Published:** 2025-09-17

**Authors:** Leander B. Crocker, Jan Vincent V. Arafiles, Judith M. Müchler, Max Ruwolt, Kristin Kemnitz-Hassanin, Kilian Roßmann, Christian E. Stieger, Fan Liu, Nataliya Archipowa, Roger Jan Kutta, Christian P. R. Hackenberger

**Affiliations:** 1 Leibniz-Forschungsinstitut für Molekulare Pharmakologie (FMP), Berlin, Germany; 2 Institut für Chemie, Humboldt-Universität zu Berlin, Berlin, Germany; 3 Institute of Biophysics and Physical Biochemistry, Faculty of Biology and Preclinical Medicine, University of Regensburg, Regensburg, Germany; 4 Institute of Theoretical and Physical Chemistry, Faculty of Chemistry and Pharmacy, University of Regensburg, Regensburg, Germany

**Keywords:** Photocatalysis, Chemical modification, Chemical tools, Target identification, Peptides

## Abstract

Photocatalytic proximity labelling has emerged as a powerful tool to resolve a variety of biomolecular and cellular interactions. Although the use of high-resolution probes, such as diazirines, enables cell-surface protein labelling with nanometre precision, intracellular applications are limited by either the intrinsic toxicity of metal-based photocatalysts or by the lower resolution when long-lived reactive intermediates are used. Here we describe the discovery, characterization and application of an organic flavin cofactor derivative, deazaflavin, that activates diazirine to generate carbenes via triplet energy transfer and offers excellent biocompatibility. We demonstrate deazaflavin–diazirine energy-transfer labelling (DarT labelling) for cell surfaceome mapping and, most importantly, for intracellular interactome mapping as exemplified for cell-penetrating peptides. We successfully map the localization of linear and cyclic polyarginine cell-penetrating peptides, identifying putative membrane interactors. Furthermore, we show the applicability of DarT labelling over an extended time period by mapping the intracellular trafficking of a stable cyclic derivative to reveal its eventual exocytosis from the cell. We anticipate that DarT labelling could be used to profile intracellular dynamics across diverse biological systems with high spatio-temporal control.

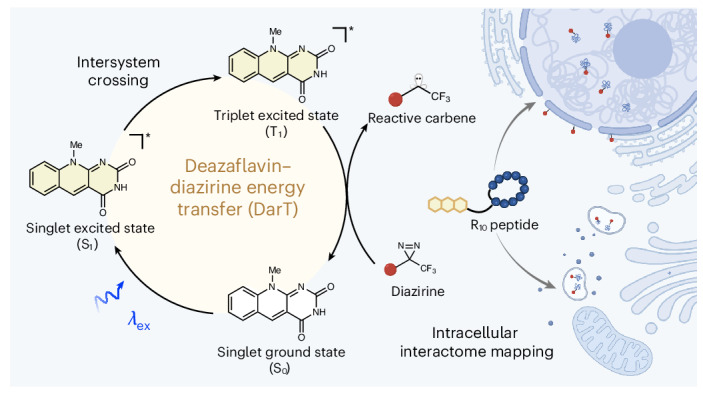

## Abstract (web summary)

Current photoproximity labelling methods often require metal-based catalysts to map protein interactomes but, owing to their toxicity, they have limited intracellular applicability. A deazaflavin cofactor has now been developed as a biocompatible alternative for diazirine activation inside living cells, offering accurate mapping of protein interactors and dynamics with excellent spatio-temporal control.

## Main

Understanding biomolecular interactions in cellular functions is key to elucidating disease pathology, the discovery of biomarkers and therapeutic development^1^. To achieve this, proximity-labelling (PL) methods, combined with recent advances in mass spectrometry have become powerful tools to decipher protein, RNA and DNA interaction networks within living cells^2,3^. Classical PL methods, such as BioID (proximity-dependent biotin identification), involve the genetic fusion of an enzyme to a protein of interest to generate reactive intermediates that label proximal biomolecules. Although extremely valuable, BioID suffers from a lack of temporal control due to exogenous biotin treatment for extended periods^4^. Systems based on ascorbate peroxidase (APEX) offer faster labelling times, but the H_2_O_2_ treatment required for generating the reactive phenoxyl radicals for labelling compromises the cell viability^4^. Photocatalytic proximity labelling or photoproximity labelling (PPL) is a useful alternative to such methods^5,6^, offering short labelling times, tunable labelling radii^7^, long-wavelength reactivity^8–10^ and the ability to be applied in vivo^11^. In addition, photoreceptor domains such as mini singlet oxygen generator (miniSOG)^12–15^ and light-oxygen-voltage (LOV)^16^ can be genetically incorporated into live cells. However, the fusion of an enzyme to a protein of interest could alter the protein’s native structure, function or intracellular trafficking and potentially interfere with its biomolecular interactions.

Microenvironment mapping (µMap) utilizes an iridium photocatalyst to activate diazirine-based probes via Dexter energy transfer to generate short-half-life carbenes (*t*_1/2_ = 2 ns) to determine protein interactomes at nanometre resolution^17^. Although highly efficient, the use of iridium photocatalysts in PPL is curtailed by the material sustainability and cytotoxicity. Other approaches have therefore emerged that use organic photocatalysts such as flavin^18–20^, acridinium and fluorescein derivatives^21–24^, which offer better suitability for intracellular PPL^25^. So far, subcellular compartments such as mitochondria^23,24^ and nuclei^21,22^ have been investigated via PPL, taking advantage of inherent photocatalyst cellular localization^23^, organelle-targeting moieties^21,24^ or protein tags^22^. However, these reports use reactive intermediates that have lower resolution compared with carbenes such as phenoxyl radicals (t_1/2_ ≤ 1 ms)^26^, nitrene-derived reactive intermediates (10 µs)^27^ or singlet oxygen (^1^O_2_; 0.2–3 µs)^28,29^. Intracellular examples of carbene generation via energy transfer have been described using iridium photocatalysts for drug target identification^30^ and stress granule interactome mapping^31^, although these suffer from cytotoxicity^30,32^ and mitochondrial localization^33,34^.

To overcome these limitations, we sought to identify a readily accessible organic photocatalyst with the following characteristics: (1) efficient energy transfer with diazirines for high-resolution PPL, (2) excellent biocompatibility and (3) spatio-temporal control over labelling. In this Article we report the discovery of a flavin cofactor analogue, deazaflavin^35^, that is capable of activating diazirines, as well as other major probes, via energy transfer in live cells. Owing to the catalyst’s biocompatibility and minimalist design, we demonstrated both extra and intracellular deazaflavin–diazirine energy-transfer labelling (DarT labelling) in live cells. DarT labelling enabled us to elucidate the protein interactome of polyarginine cell-penetrating peptides (CPPs), revealing insights into their uptake, localization and intracellular trafficking.

## Results and discussion

### Photocatalyst performance and characterization

**Fig. 1:**
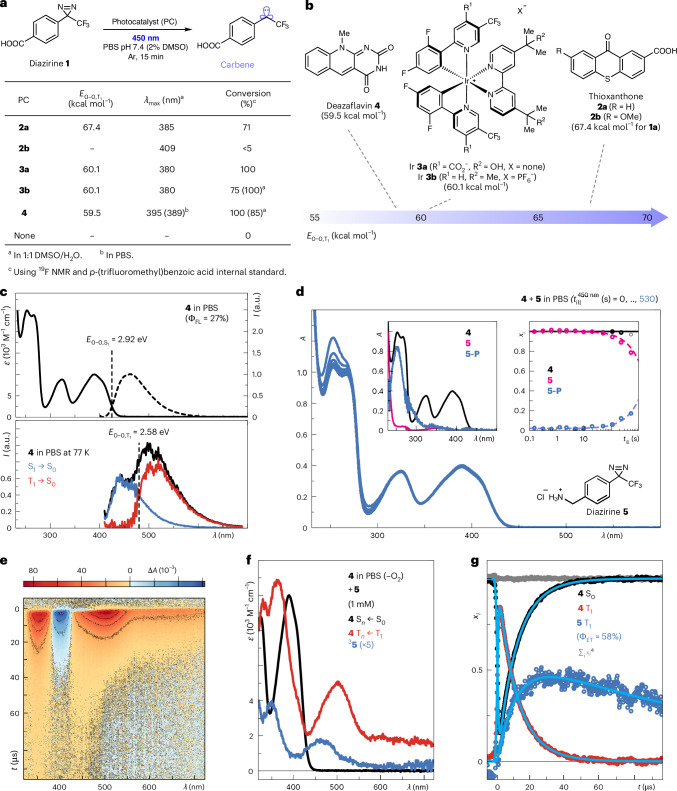
Photocatalyst performance, photophysics and validation of energy transfer. **a**, Photocatalytic conversion of diazirine **1** using photocatalysts **2**–**4**, with their λ_max_ values of the corresponding first absorption band and associated triplet energies ($E_{0\textit{--}0,{\mathrm{T}}_1}$) listed. **b**, Structures of photocatalysts **2**–**4** with their corresponding triplet energy ($E_{0\textit{--}0,{\mathrm{T}}_1}$) values. **c**, Top: steady-state absorption (solid line) and emission (dashed line) spectra in the ultraviolet/visible (UV/vis) spectral range of **4** in PBS at 295 K, showing the excited singlet emission (fluorescence) quantum yield (Φ_FL_) at 295 K and the excited singlet-state energy above the ground state ($E_{0\textit{--}0,{\mathrm{S}}_1}$). Bottom: emission spectrum of **4** in PBS at 77 K, showing the triplet-state energy above the ground state ($E_{0\textit{--}0,{\mathrm{T}}_1}$) at 77 K. *ε*, molar absorption coefficient; *I*, intensity; S_0_, singlet ground state; S_1_ (T_1_), singlet (triplet) excited state. **d**, Sequence of steady-state absorption spectra in the UV/vis spectral range for the stepwise illumination of **4** in PBS in the presence of diazirine **5** at 450 nm as indicated. Insets show the species-associated spectra contributing to the data (left) and the profiles of the corresponding mole fraction (*x*_*i*_) over time (right). The dashed lines represent the sum of exponential decays, as a guide to the eye. *A*, absorbance; *t*_ill_, illumination time; **5-P**, product of **5** after energy transfer. **e**, Transient absorption spectra in the UV/vis spectral range of **4** in PBS in the presence of **5** following excitation at 410 nm. **f**,**g**, Transient absorption spectra from **e** decomposed into their species-associated spectra (**f**) and corresponding concentration–time profiles (**g**). The cyan lines in **g** represent the global fits to the data.

We first investigated thioxanthones^36–38^ as potential triplet energy sensitizers to activate diazirine **1** (with a triplet energy (*E*_T_) of ≥60.1 kcal mol^−1^)^17^ in phosphate-buffered saline (PBS) upon irradiation at 450 nm using a light-emitting diode (LED) (Fig. 1a,b). Thioxanthone **2a** (*E*_T_ = 67.4 kcal mol^−1^)^39^ provided a good conversion (>70%) of diazirine **1** upon irradiation for 15 min in PBS, despite little absorbance overlap with the incident photons (maximum of the first electronic absorption transition (*λ*_max_) of **2a** = 385 nm; Fig. 1a and Supplementary Fig. 1a). Attempts to increase the blue light absorption of **2a** through methoxy substitution (to thioxanthone **2b**; Fig. 1a,b and Supplementary Fig. 1a) resulted in a diminished conversion of **1** (<5%, Fig. 1a), possibly by lowering the excited triplet energy^39^. As expected, the water-soluble iridium photocatalyst **3a** provided complete conversion of **1** in PBS, whereas the more hydrophobic version **3b** was most effective in a 1:1 mixture of dimethyl sulfoxide and water (DMSO/H_2_O) (Fig. 1a)^17,40^.

Subsequently, we prepared the high-triplet-energy flavin derivative deazaflavin **4** (Fig. 1c; *E*_T_ = 59.5 kcal mol^−1^)^41^, and the complete conversion of **1** after irradiation at 450 nm for 15 min in PBS was observed via ^19^F NMR (Fig. 1a and Supplementary Fig. 2). This was comparable to the iridium catalyst **3a**, but **4** did not achieve full conversion of **1** in 1:1 DMSO/H_2_O (85%) after 15 min compared with iridium catalyst **3b**. Further analysis revealed that the photocatalytic conversion of DMSO to dimethyl sulfone by deazaflavin **4** in 1:1 DMSO/H_2_O enhanced its decomposition when compared with PBS, thereby explaining this result (Supplementary Fig. 3)^42^. For the conversion of diazirine **1**, other flavin-derived photocatalysts were also tested, such as riboflavin tetraacetate (RTA)^18–20^, deazaflavin **S1** (ref. ^43^) and alloxazine **S2** (refs. ^44,45^) (Supplementary Figs. 1b and 4), but these were unable to react efficiently (<50% conversions), probably due to lower excited triplet energies. Thus, deazaflavin **4**, which was also readily synthesized in two steps, emerged as the optimal organic photocatalyst among all of the tested photocatalysts for diazirine activation.

In addition, the conversion of aryl azide **S3** (*E*_T_ ≥ 43.8 kcal mol^−1^)^7–9,23^ was explored for our panel of photocatalysts, from which 100% conversion was observed for deazaflavin **4**, and >75% was seen for most of the others (Supplementary Fig. 4). However, control experiments without any photocatalyst showed the blue-light-induced photolysis of aryl azide **S3** (13%), which has been reported previously^9^. On the other hand, diazirine activation is dependent on the presence of **4** and blue light (Fig. 1a and Supplementary Fig. 5, entry 2) and can be completed in 5 min under identical conditions (Supplementary Fig. 5, entry 3). Substitution of **4** at the N-3 position had no effect on its catalytic activity (Supplementary Fig. 5, entry 4), indicating that this position could therefore be used for attachment to a targeting modality for PPL application. Finally, other diazirine probes containing amine and biotin handles can be activated using **4** (Supplementary Fig. 5, entries 5 and 6, respectively), and these showed reactivity in 10 mM glutathione (GSH) to resemble intracellular conditions, although with reduced efficacy (55%, Supplementary Fig. 5, entry 7).

### Mechanistic investigation of deazaflavin photocatalysis

**Extended Data Fig. 1:**
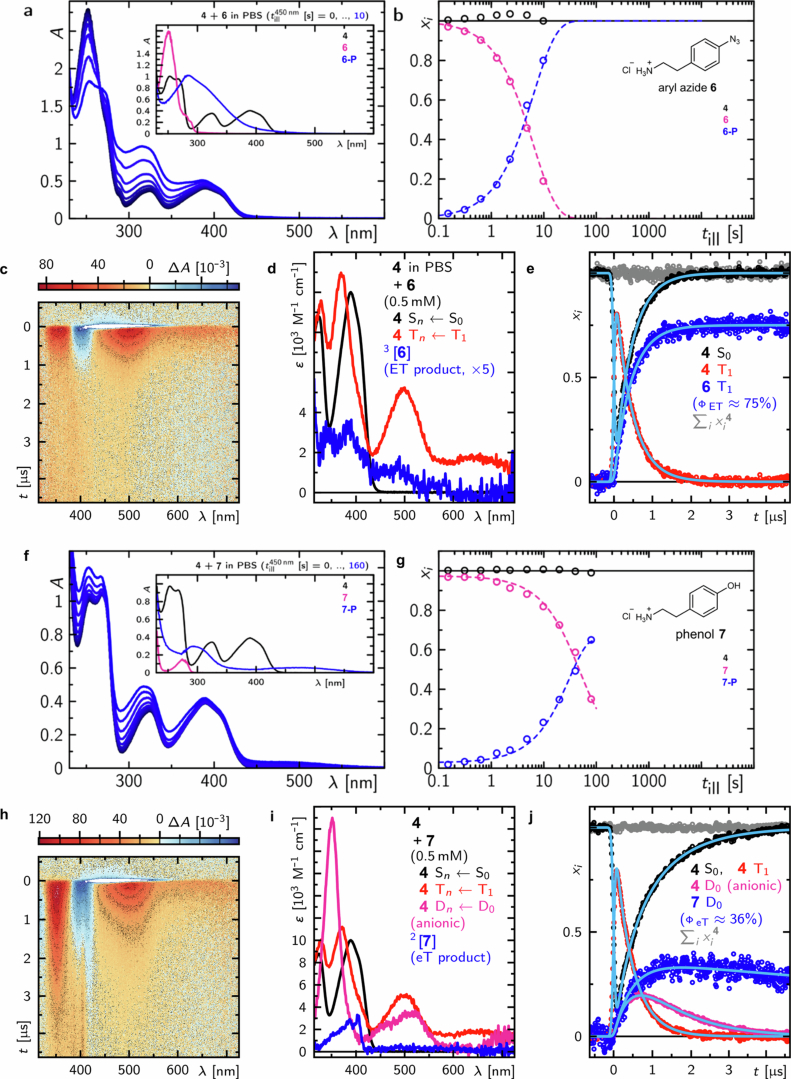
Photocatalytic activity of deazaflavin 4 towards aryl azide 6 and phenol 7. (**a**) Sequence of steady state absorption spectra in the UV/Vis spectral range of **4** in PBS buffer in the presence of **6** with stepwise illumination at 450 nm and (**b**) corresponding mole fraction over time profile. (**c**) Transient absorption spectra in the UV/Vis spectral range of **4** in PBS buffer in the presence of **6** following excitation at 410 nm. The spectra are decomposed into (**d**) species-associated spectra and (**e**) corresponding concentration time profiles. (**f**) Sequence of steady state absorption spectra in the UV/Vis spectral range of **4** in PBS buffer in the presence of **7** with stepwise illumination at 450 nm and (**g**) corresponding mole fraction over time profile. (**h**) Transient absorption spectra in the UV/Vis spectral range of **4** in PBS buffer in the presence of **7** following excitation at 410 nm. The spectra are decomposed into (**i**) species associated spectra and (**j**) corresponding concentration time profiles. The cyan lines in (**e**) and (**j**) represent the global fit to the data.

As elucidated by transient absorption spectroscopy, deazaflavin **4** efficiently forms its triplet state (yield of intersystem crossing Φ_ISC_ ≈ 65%) with a lifetime (*τ*) of 1.8 μs in non-degassed PBS, mainly decaying via diffusion-controlled energy transfer to form ^1^O_2_ (Φ_Δ_ = 62% in PBS; Supplementary Fig. 6). This efficient formation explains the eventual decomposition of **4** under prolonged illumination (Supplementary Fig. 3a). The respective excited-state dynamics of **4** in the presence of model photolabelling probes diazirine **5**, aryl azide **6** and phenol **7** was first investigated via the stepwise illumination of **4** (Fig. 1d and Extended Data Fig. 1). Product formation was observed without photocatalyst degradation at various reaction efficiencies, as **6** > **7** > **5** (Fig. 1d and Extended Data Fig. 1b,f). Furthermore, the conversion of diazirine **5** and aryl azide **6** is initiated by triplet energy transfer, as shown by the triplet state of deazaflavin **4** decaying back to its ground state and the simultaneous rise of the substrate triplet state (Fig. 1e–g and Extended Data Fig. 1c–e). The quantum efficiency values for triplet energy transfer (Φ_ET_) are 58% for diazirine **5** and 75% for aryl azide **6** (at 500 μM substrate concentration), which agree with the conversion rates observed on a steady-state level (Fig. 1d and Extended Data Fig. 1a,b,f,g). In the case of phenol **7**, the conversion is initiated via single-electron transfer (SET) with a quantum yield of electron transfer (Φ_eT_) of 36% (at 500 μM substrate concentration, Extended Data Fig. 1h–j). This agrees with the reported redox potentials of phenol moieties (Tyr/Tyr^**•**+^ = 1.08 V versus the saturated calomel electrode (SCE))^20^ and excited **4** (4*/**4**^**•**^^−^ ≈ 1.20 V versus SCE)^45^ leading to favourable electron transfer, similar to other flavins^16,18–20^.

Considering that GSH is a major intracellular reductant, its reactivity with **4** was investigated to reveal efficient electron transfer from GSH (50 mM) to the triplet state of **4** (Φ_eT_ = 73%; Supplementary Fig. 7). Notably, the forward electron transfer is also accompanied by fast intersystem crossing within the radical pair and finally radical pair recombination. Therefore, intracellular diazirine conversion will depend on the relative concentrations of GSH and diazirine, as both compete for reaction with the triplet state of **4**. Given the reversible nature of its reaction with GSH, multiple excitation cycles of **4** would still permit photocatalytic diazirine conversion even at high GSH concentrations. This finding, supported by control experiments (Supplementary Fig. 5, entry 7), underlines the suitability of deazaflavin **4** towards intracellular PPL applications.

### In vitro protein photolabelling

**Fig. 2:**
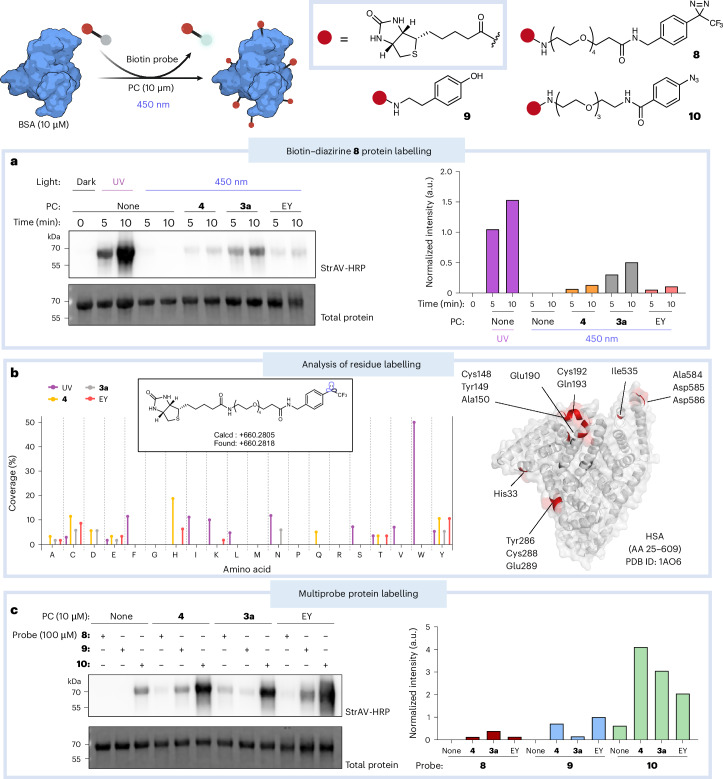
In vitro protein photolabelling. Schematic of the photolabelling of BSA (Protein Data Bank (PDB) ID: 4F5S) using biotin–diazirine probes (**8**, **9** and **10**). **a**, Results of the photolabelling of BSA with biotin–diazirine probe **8** (100 µM) in PBS using either UV light (365 nm) or blue light (450 nm) with deazaflavin **4**, iridium catalyst **3a** or EY (10 µM). The graph (right) shows the normalized band intensity of streptavidin-horseradish peroxidase (StrAV-HRP) versus the total protein of the representative blot (left). **b**, Left: results of LC-MS/MS analysis of the labelling of recombinant HSA residues by diazirine **8** (inset structure) using either UV light (365 nm, 10 min) or blue light (450 nm, 10 min) with deazaflavin **4**, iridium catalyst **3a** or EY (10 µM). Coverage (%) represents the percentage of a specific residue labelled within the total count of that residue in the protein. Right: residues labelled using deazaflavin **4** are marked on HSA (PDB ID: 1AO6). AA, amino acids. **c**, Multiprobe protein labelling of BSA using probes **8**–**10** with blue light (450 nm) with deazaflavin **4**, iridium catalyst **3a** or EY. The graph (right) shows the normalized band intensity of StrAV-HRP versus the total protein of the representative blot (left). Schematic panel created with BioRender.com. Source data

**Extended Data Fig. 2:**
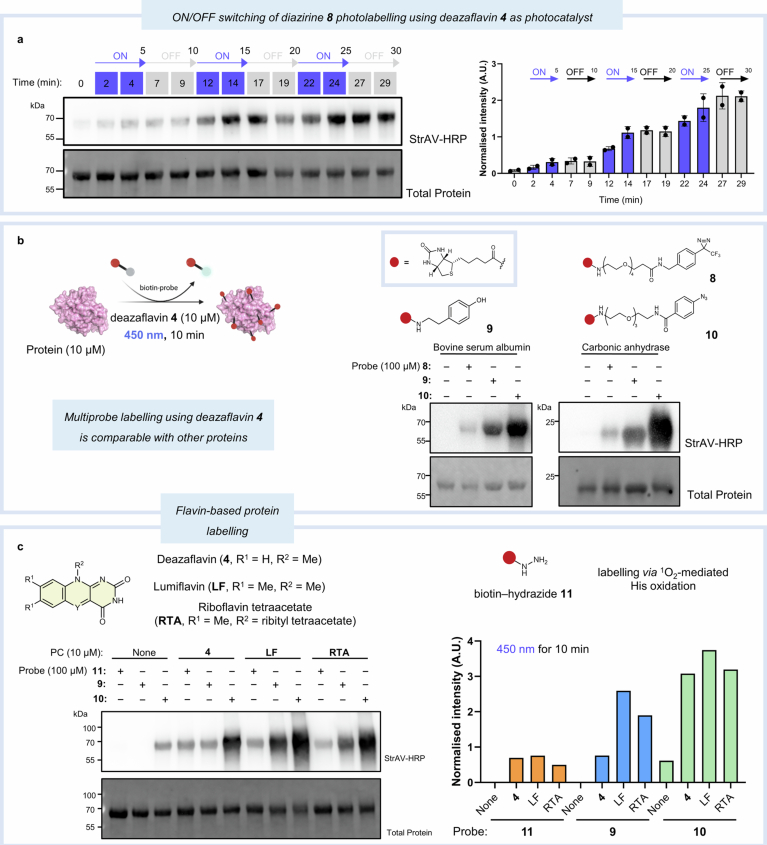
*In vitro* protein photolabelling analyses. (**a**) Western blot and quantification of ON/OFF illumination switching at 450 nm of BSA (10 µM) photolabelling using deazaflavin **4** (10 µM) and diazirine **8** (100 µM). Graph shows the normalised intesity of bands from strepavidin-HRP (StrAv-HRP) blot versus total protein bands. Data presented as mean ± standard deviation of two independent measurements. (**b**) Western blots of BSA and carbonic anhydrase (PDB: 3KS3) multiprobe (**8**-**10**) photolabelling (conditions as indicated). (**c**) Western blot of flavin-based photolabelling using probes **9**-**11** (100 µM) and BSA (10 µM) in PBS (pH = 7.4) at 450 nm for 10 min. Graph shows the normalised intesities of bands from StrAv-HRP blot versus total protein bands of a representative blot of two independent assays. Source data

We next examined protein photolabelling using bovine serum albumin (BSA), deazaflavin **4** and biotin–diazirine probe **8**, benchmarking its activity against iridium catalyst **3a** and the recently reported organic photocatalyst eosin Y (EY)^46^. Western blot analysis of 5 and 10 min irradiation times revealed that iridium catalyst **3a** elicits the greatest degree of photolabelling compared with deazaflavin **4** or EY, which have comparable efficacies (Fig. 2a). These differences can be explained by the slightly higher triplet energy of iridium catalyst **3a** (*E*_T_ = 60.1 kcal mol^−1^) and the robust photostability of iridium polypyridyl complexes in general^47^. UV light (365 nm) irradiation of diazirine **8** alone displayed the greatest degree of biotinylation, which is expected due to global activation of the compound in solution. We further demonstrated the light-driven nature of BSA biotinylation by deazaflavin **4** and diazirine **8** by alternating light and dark incubations over time (Extended Data Fig. 2a).

The exact residue labelling of biotin–diazirine **8** was explored using recombinant human serum albumin (HSA) via liquid chromatography-tandem mass spectrometry (LC-MS/MS) using either UV light (365 nm for 10 min) or blue light (450 nm for 10 min) with the deazaflavin **4**, iridium **3a** or EY photocatalyst. As expected, carbenes generated by UV irradiation labelled the protein with a greater coverage of available residues compared with the photocatalysts across various amino-acid residues (Fig. 2b). Polar nucleophilic residues such as cysteine, glutamic acid and tyrosine were labelled more prominently by all three photocatalysts, in addition to some non-polar residues such as alanine, which aligns with reported aryl diazirine reactivity^48^. Furthermore, using a smaller acetylated benzylamine diazirine as a model probe (compound **S4**; Supplementary Fig. 8) resulted in a wider coverage of labelled residues with UV-light activation; the same was true with deazaflavin **4** and iridium **3a** photocatalysis, which showed similar labelling trends with other amino acids such as glycine, lysine, phenylalanine and arginine (Supplementary Fig. 8). Overall, the photocatalytic activation of aryl diazirine probes resulted in similar trends of amino-acid labelling when compared with direct activation using UV light. However, the overall efficiency of photocatalytic activation and the structure of the probe affects the residue labelling considerably.

Furthermore, protein photolabelling of BSA and carbonic anhydrase using multiple biotin probes **8**–**10** in the presence of **4** confirmed the photocatalytic ability of deazaflavin **4** (Fig. 2c and Extended Data Fig. 2b). In general, diazirine **8** labelled proteins to a lesser extent due to the short reactive half-life of carbenes (1–2 ns), as shown by the less intense western blot bands when compared with the aryl azide **10** and phenol **9** probes. Additional benchmarking showed that iridium catalyst **3a** was the most efficient at diazirine activation, whereas protein labelling via SET using phenol **9** was not as efficient as **4** or EY. Aryl azide **10** showed the highest degree of labelling in all cases, but it also exhibited reactivity in the control experiments without a photocatalyst, aligning with our previous findings (Supplementary Fig. 4).

Flavin photocatalysts or photoreceptors have previously been used for protein labelling with either phenol probes or hydrazide probes to label oxidized histidines (Extended Data Fig. 2c)^12–15,18–20^. Our initial screening showed that RTA activates model aryl azide **S3** upon irradiation but not diazirine **1** (Supplementary Fig. 4, entry 8). Thus, we compared the protein labelling ability of deazaflavin **4** with RTA and lumiflavin (LF) using hydrazide (**11**), phenol (**9**) and aryl azide (**10**) biotin probes. Protein labelling using biotin–hydrazide **11** and biotin–aryl azide **10** was comparable using all flavins; however, LF and RTA were more efficient SET catalysts for biotin–phenol **9** oxidation (Extended Data Fig. 2c). This is in line with the less oxidizing nature of deazaflavins compared with the parent flavins (excited state reduction potential (*E*_red_*) of RTA = 1.67 V versus SCE)^18–20,49^. Altogether, these data demonstrate that deazaflavin **4** is capable of multiscale PPL that has been shown to provide variable resolution in interactome profiling^46^. However, given the limitations of aryl azide photolysis using blue light and the lower-oxidizing SET ability of deazaflavins compared with parent flavins, diazirines are the most suitable probe for deazaflavin-based PPL, thereby taking advantage of their distinctive energy-transfer capability. The approach is henceforth termed DarT labelling (deazaflavin–diazirine energy-transfer labelling).

**Extended Data Fig. 3:**
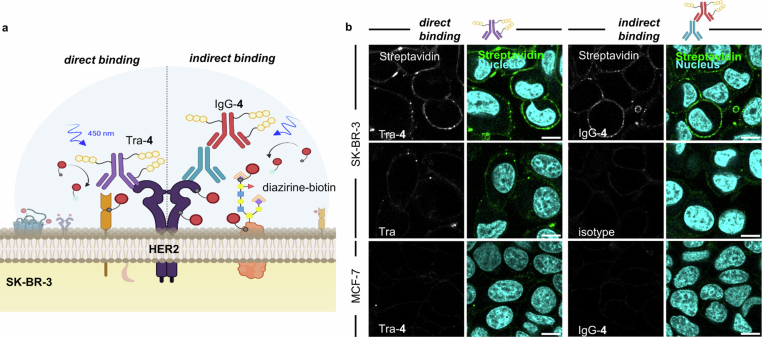
Extracellular protein photolabelling using deazaflavin-antibody conjugates. (**a**) Schematic presentation of cell surface photolabelling using HER2-targeted labelling employing either primary antibody-flavin conjugate Tra-**4** or secondary IgG-**4** in a direct and indirect approach, respectively. (**b**) Representative confocal images of SK-BR-3 (HER2-positive) and MCF-7 (HER2-negative) cells treated with directly binding Tra-**4** or unmodified Trastuzumab as a control (left); or with the indirect primary/secondary system by using anti-HER2 antibody and IgG-**4** or a non-targeting isotype control (right). All conditions include subsequent incubation with 250 µM diazirine-biotin **8**, followed by irradiation at 450 nm for 10 min. Cells were additionally imaged for nuclei (Hoechst stain, blue); Scale bars=10 μm.

We initially validated the DarT labelling of proteins in a biological system using deazaflavin–antibody conjugates targeting a cell-surface receptor (that is, human epidermal growth factor receptor 2 (HER2); Extended Data Fig. 3)^50^. We used two different immunolabelling approaches: direct binding using a deazaflavin–trastuzumab conjugate (Tra-**4**) and indirect binding using a polyclonal anti-mouse immunoglobulin G (IgG) conjugate with deazaflavin **4** (IgG-**4**) (Supplementary Sections 3.1.1 and 3.4.2, respectively). After antibody treatment, DarT labelling was performed by treating HER2-overexpressing SK-BR-3 cells with biotin–diazirine probe **8** and irradiation at 450 nm for 10 min. Cell-surface biotinylation was visualized through confocal microscopy imaging using a streptavidin–Alexa Fluor 488 conjugate that revealed a clear fluorescent signal for Tra-**4**- and IgG-**4**-treated cells (Extended Data Fig. 3b). Control experiments with unmodified trastuzumab (Tra), an isotype primary IgG and HER2-negative cell line MCF-7 showed no clear fluorescent signal, which provided clear evidence of the targeted DarT labelling of extracellular proteins (Extended Data Fig. 3b).

### Surfaceome mapping via DarT labelling

**Fig. 3:**
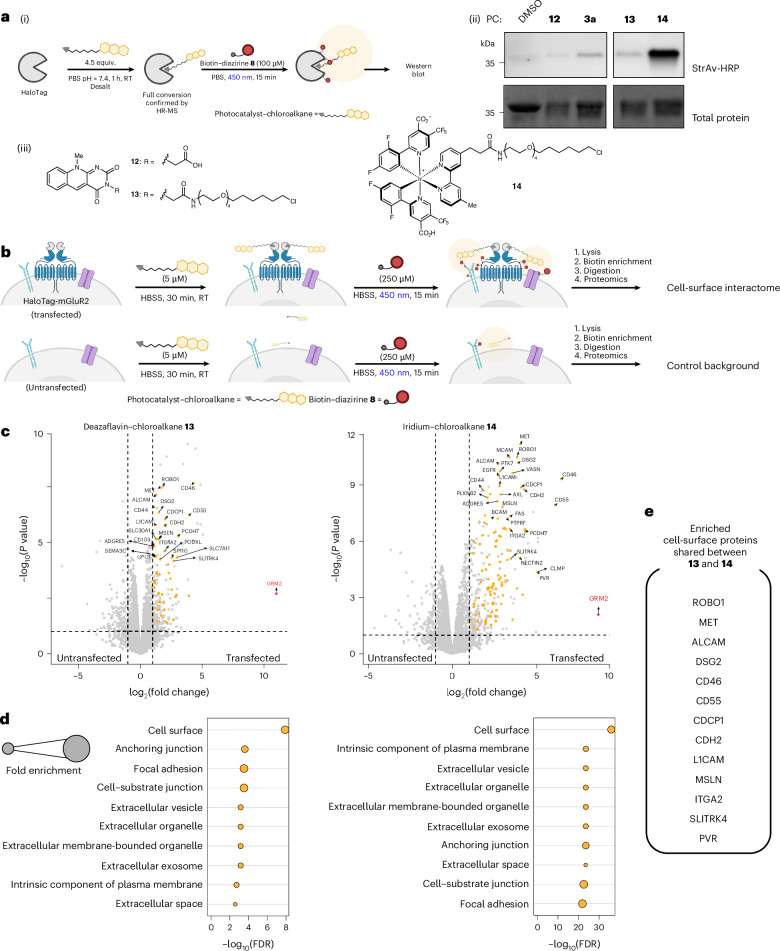
Extracellular DarT labelling of HeLa cells expressing HaloTag-mGluR2. **a**, Schematic of biotin–diazirine labelling of the photocatalyst–chloroalkane-conjugated HaloTag (i). Representative western blot of the biotin-labelled HaloTag. The assay was replicated with similar results (ii). Structures of extracellular photocatalyst–chloroalkane analogues (iii). **b**, Schematic of cell-surface DarT labelling on HeLa cells expressing HaloTag-mGluR2 (HeLa-HaloTag-mGluR2). HBSS, Hank’s balanced salt solution. **c**, Volcano plots from the LFQ analysis of HeLa cells (untransfected control) versus HeLa-HaloTag-mGluR2 (transfected) treated with deazaflavin–chloroalkane **13** (left) and iridium–chloroalkane **14** (right) for 30 min at 22 °C, followed by treatment with diazirine **8** (250 µM) and 15 min of irradiation at 450 nm. Significantly enriched proteins (*P* < 0.05 and log_2_(fold change) > 1) are coloured, with the most significantly enriched proteins from the GO term ‘cellular compartment’ labelled relating to the cell surface (orange). The top 20 significantly enriched proteins are labelled. *P* values were calculated through FragPipe-Analyst^78^ using an empirical Bayes moderation of two-sided *t*-statistics without further adjustments. Data were obtained from three independent replicates. **d**, GO analysis of significantly enriched proteins relating to cellular compartment for deazaflavin–chloroalkane **13** (left) and iridium–chloroalkane **14** (right). FDR, false discovery rate. **e**, List of enriched cell-surface proteins shared between treatments with deazaflavin–chloroalkane **13** and iridium–chloroalkane **14**. Panel **b** created with BioRender.com. Source data

Cell-surface interactome mapping via PPL has progressed greatly in recent years, revealing various novel interaction networks on and between cell surfaces^9,17,18,46,51^. We therefore applied DarT labelling for surfaceome mapping using a cell-surface HaloTag fused to the N terminus of metabotropic glutamate receptor 2 (HeLa-HaloTag-mGluR2)^52^, which also enables straightforward benchmarking to established PPL systems such as iridium-based µMap. We first validated the HaloTag self-labelling with deazaflavin–chloroalkane **13** or iridium–chloroalkane **14** using high-resolution mass spectrometry (Supplementary Fig. 9a–d) and subsequently performed in vitro photolabelling (Fig. 3a(i)). Western blot analysis of the HaloTag incubated with control compounds **3a** and **12** showed minimal biotinylation, whereas the HaloTag with covalently attached **13** or **14** showed substantially more biotinylation (Fig. 3a(ii)). Consistent with the in vitro photolabelling (Fig. 2a), the iridium-based photocatalyst **14** displayed a higher efficacy of diazirine activation compared with deazaflavin **13**. Still, these data confirm that DarT labelling can be activated following covalent self-labelling of the HaloTag protein. Consequently, a microscopy-based competitive binding assay confirmed that the photocatalyst–chloroalkane conjugates bind to cell-surface HaloTag fusion proteins (Supplementary Fig. 9e,f).

**Extended Data Fig. 4:**
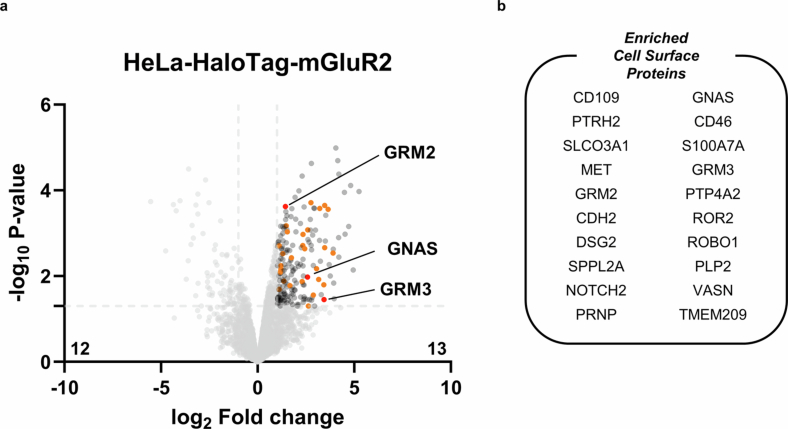
Extracellular DarT-labelling of HeLa cells expressing HaloTag-mGluR2 using a deazaflavin control. (**a**) Label-free quantification (LFQ) volcano plot of HeLa-HaloTag-mGluR2 cells treated with triethyleneglycol-deazaflavin **12** (control) versus chloroalkane-deazaflavin **13** treatment. P-values were calculated through FragPipe-Analyst using an empirical Bayes moderation of two-sided *t* statistics without further adjustments. Significantly enriched proteins are determined by P-value < 0.05 and log2 fold-change > 1. Orange points represent cell surface proteins. (**b**) Top 20 enriched cell surface proteins.

For proteomic surfaceome mapping, HeLa cells (untransfected control) and HeLa-HaloTag-mGluR2 cells (transfected) were treated with deazaflavin–chloroalkane **13** followed by the addition of biotin–diazirine **8** (250 µM) and irradiation (450 nm, 15 min). Biotinylated proteins were then enriched and digested for LC-MS/MS analysis (Fig. 3b). Label-free quantification (LFQ) provided volcano plots that showed 169 significantly enriched proteins (*P* < 0.05 and log_2_(fold change) > 1) in transfected cells treated with deazaflavin–chloroalkane **13**, among which, 51 hits (30% of the enriched proteins) were annotated as cell-surface proteins (orange dots, Fig. 3c, left). Furthermore, Gene Ontology (GO) enrichment analysis verified cell-surface protein enrichment (Fig. 3d, left). Repetition of the workflow using HeLa-HaloTag-mGluR2 cells treated with the chloroalkane-free deazaflavin analogue **12** or deazaflavin–chloroalkane **13** (Extended Data Fig. 4) confirmed that the significant enrichment of mGLuR2 (GRM2 (red dot) in the volcano plots) was due to the specific conjugation of deazaflavin–chloroalkane **13** to HaloTag-mGluR2. In addition, the majority of the top 20 enriched cell-surface proteins (Extended Data Fig. 4b) matched the experiments with the untransfected control cells (Fig. 3e), confirming the specificity of DarT labelling.

Next, we compared extracellular DarT labelling with the cell-impermeable iridium–chloroalkane conjugate **14** (right of Fig. 3c,d). Treatment with **14** resulted in a larger number of significantly enriched proteins in the transfected cells (259 with 107 cell-surface proteins—41% of the total enriched proteins), which is consistent with the higher degree of biotinylation observed with in vitro protein labelling. For both labelling methods, mGluR2 was significantly enriched (GRM2 (red dot) in the volcano plots), with multiple shared cell-surface protein hits (Fig. 3e). Comparing the significantly enriched proteins from both treatments showed 85 overlapping hits that are largely annotated as cell-surface proteins (Supplementary Fig. 10). Unique hits for deazaflavin–chloroalkane **13** were found to be nucleus-associated proteins (Supplementary Fig. 10c), indicating the partial cell permeability of this chloroalkane conjugate and the capacity of flavin derivatives to interact with oligonucleotides^53–55^.

### Deazaflavin biocompatibility and cell permeability

**Fig. 4:**
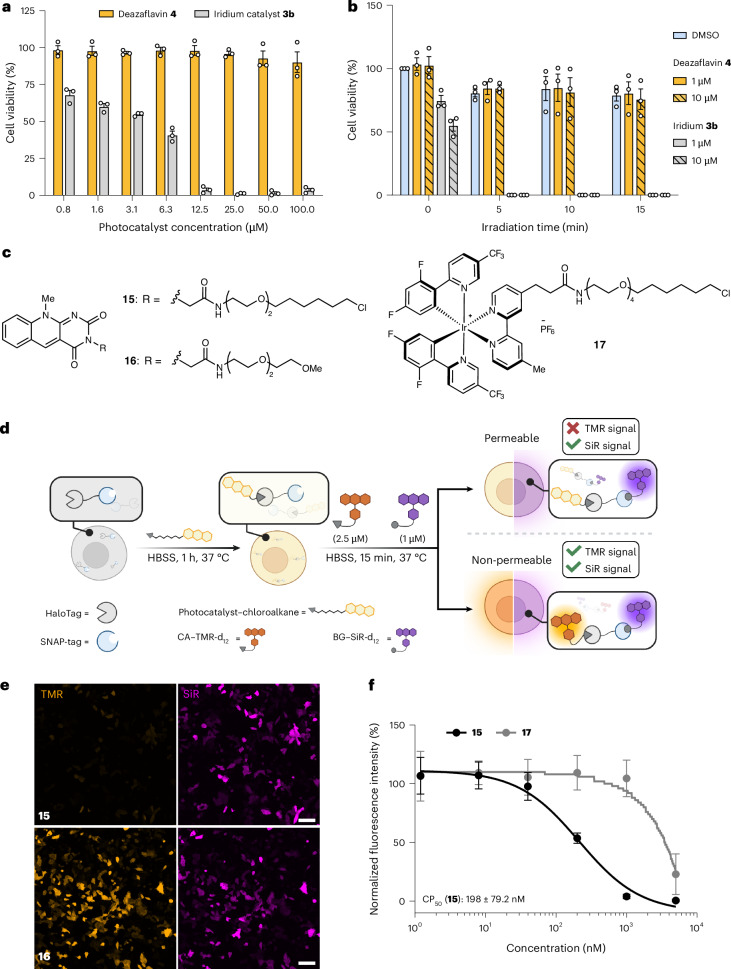
Cellular biocompatibility and permeability of deazaflavin 4. **a**, Cytotoxicity assay (WST-1) of iridium catalyst **3b** and deazaflavin **4** after 24 h of incubation in HeLa cells. Data are presented as the mean ± standard error of the mean (SEM) of three biological replicates. **b**, Light-induced toxicity of **3b** and **4**. HeLa cells were treated with the photocatalysts for 1 h, washed and then irradiated (450 nm) for 0, 5, 10 and 15 min, followed by recovery for 24 h in fresh cell culture medium before WST-1 assay. Data are presented as the mean ± SEM of three biological replicates. **c**, Structures of the photocatalysts for the CAPA assay. **d**, Schematic of the modified CAPA assay. Permeability is validated by treatment with CA–TMR-d_12_ and BG–SiR-d_12_. **e**, Tile-scan confocal microscopy images of modified CAPA reporter HeLa cells treated with **15** or **16** (5 µM) for 1 h, followed by CA–TMR-d_12_ (2.5 µM) and BG–SiR-d_12_ (1 µM) for 15 min. Scale bars, 100 µm. **f**, Normalized fluorescence intensity (TMR) of reporter HeLa cells following treatment with **15** and **17** (1.2, 8, 40, 200, 1,000 and 5,000 µM), monitored using CA–TMR-d_12_ (2.5 µM) and BG–SiR-d_12_ (1 µM) determined via flow cytometry. The CP_50_ value for treatment with **15** is provided. Data points are presented as the mean ± standard deviation of three biological replicates. Panel **d** created with BioRender.com.

**Extended Data Fig. 5:**
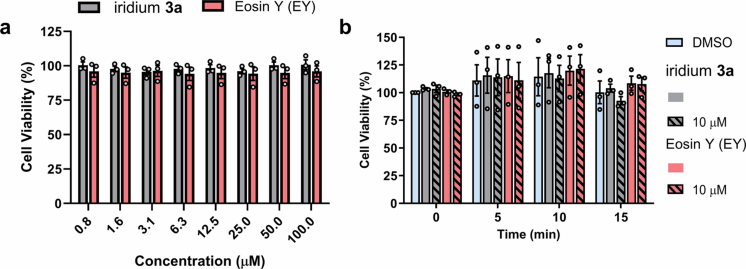
Biocompatibility of iridium 3a and Eosin Y. (**a**) Cytotoxicity assay (WST-1) of iridium **3a** and Eosin Y (EY) after 24 h incubation in HeLa cells. Data presented as mean ± standard error of mean (SEM) of three biological replicates. (**b**) Light-induced toxicity of **3a** and EY. HeLa cells were treated with photocatalysts for 1 h, washed then irradiated (450 nm) for 0, 5, 10, 15 min, followed by 24 h recovery prior to WST-1 assay. Data presented as mean ± SEM of three biological replicates.

We next evaluated the biocompatibility of deazaflavin **4** in live cells before any intracellular application of DarT labelling. Initially, the cytotoxicity of **4** was compared with cell-permeable iridium photocatalyst **3b** (Fig. 4a)^30^ as well as cell-impermeable iridium photocatalyst **3a** and EY (Extended Data Fig. 5a). The viability of HeLa cells was measured via WST-1 assay after incubation of the catalysts for 24 h, revealing that, even at a very high concentration (100 µM), the cells maintained a viability of >85% in the presence of deazaflavin **4**, confirming its excellent biocompatibility (Fig. 4a). On the other hand, iridium catalyst **3b** proved to be highly cytotoxic, where it was shown that a concentration of **3b** as low as 0.78 µM reduced the cell viability to <70%. This is in contrast to previous work using the same catalyst^33^, although a more recent report aligns with our findings^31^. Cell-impermeable iridium catalyst **3a** and EY do not induce cytotoxicity as they are unable to interfere with intracellular metabolic processes (Extended Data Fig. 5a).

We next investigated phototoxicity by pre-incubating the catalysts for 1 h with HeLa cells before irradiating them with 450 nm light for between 0 and 15 min. The cell viability measured after 24 h showed that, after irradiation for 5 min, the cell-permeable iridium catalyst **3b** completely reduced the cell viability to 0% at concentrations of both 1 and 10 µM (Fig. 4b). This could be related to highly efficient cytotoxic ^1^O_2_ generation (Φ_Δ_ = 93% in acetonitrile (Supplementary Fig. 11)) and/or oxygen-independent photoredox mechanisms that are known for cationic iridium complexes^56^. On the other hand, cells treated with deazaflavin **4** demonstrated low phototoxicity after irradiation for 5 min (>80% viability at 1 and 10 µM), which are comparable to the DMSO control and the cell-impermeable catalysts **3a** and EY (Extended Data Fig. 5b). Blue-light irradiation alone reduced the cell viability to 79% after 15 min due to endogenous photosensitizers inducing oxidative stress^57^. Deazaflavin **4** displays little cellular phototoxicity, despite being an efficient oxygen sensitizer (Φ_Δ_ = 62% in PBS (Supplementary Fig. 3); Φ_Δ_ = 64% in acetonitrile (Supplementary Fig. 11)). Therefore, intracellular oxygen sensitization by deazaflavin **4** is probably in competition with electron-transfer processes to its triplet state by reducing compounds such as GSH (vide supra; Supplementary Fig. 7).

**Extended Data Fig. 6:**
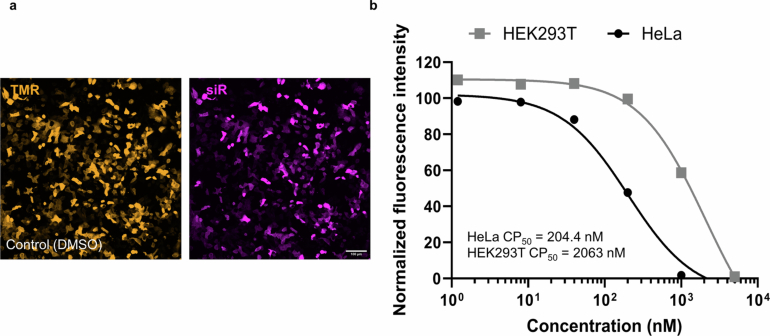
Assessment of deazaflavin cell penetration using modified chloroalkane penetration assay. (**a**) Confocal microscopy images of CAPA reporter cells treated with DMSO (negative control). Scale bar = 100 µm. (**b**) Normalised fluorescence intensity (TMR) of reporter HeLa cells or HEK293T cells following treatment with **16** (1.2, 8, 40, 200, 1000, 5000 µM), followed by CA-TMR-d12 (2.5 µM) and BG-SiR-d12 (1 µM) determined by flow cytometry. CP50 values for treatment are provided. Representative data of two biological replicates is presented.

The cell permeability of deazaflavin **4** was assessed using a modified pulse-chase chloroalkane penetration assay (CAPA) in HeLa and HEK293T cell lines^58^. Deazaflavin–chloroalkane conjugate **15** (Fig. 4c) was used to pulse reporter cells expressing a cytoplasmic HaloTag–SNAP-tag fusion protein, and cell-permeable red dye chloroalkane–tetramethylrhodamine-d_12_ (CA–TMR-d_12_) was used to chase the unoccupied HaloTag. Far-red benzylguanine–silicon rhodamine-d_12_ (BG–SiR-d_12_)^59^ was used normalize for transient transfection efficiency (Fig. 4d). The cell permeability of **15** was confirmed by the observation of a lower TMR signal compared with the triethylene glycol derivative **16** (Fig. 4e) and DMSO (Extended Data Fig. 6a) via confocal microscopy. Using flow cytometry, we determined the CP_50_ (the treatment concentration where 50% of the HaloTags are labelled) of **15** in HeLa cells to be 198 ± 79.2 nM, and 2,063 nM in HEK293T cells (Extended Data Fig. 6b). The appreciable difference in CP_50_ values indicates a variable degree of deazaflavin permeability due to the altered cell line physiology.

As a benchmark, the cell-permeable chloroalkane-functionalized iridium photocatalyst **17** had a CP_50_ of 3,345 nM in HeLa cells (Fig. 4f)^30,31^. The large size and hydrophobic structure of this iridium complex may explain its high CP_50_ value. Other recently introduced homoleptic iridium complexes with reduced cytotoxicity have demonstrated an even lower cell permeability than iridium catalyst **17**, thereby requiring overnight incubation at high concentrations for intracellular PPL experiments^60^. Overall, the excellent biocompatibility and cell permeability of deazaflavin **4** makes DarT labelling a highly suitable methodology for intracellular PPL applications.

### Intracellular mapping of the decaarginine CPP interactome via DarT labelling

**Fig. 5:**
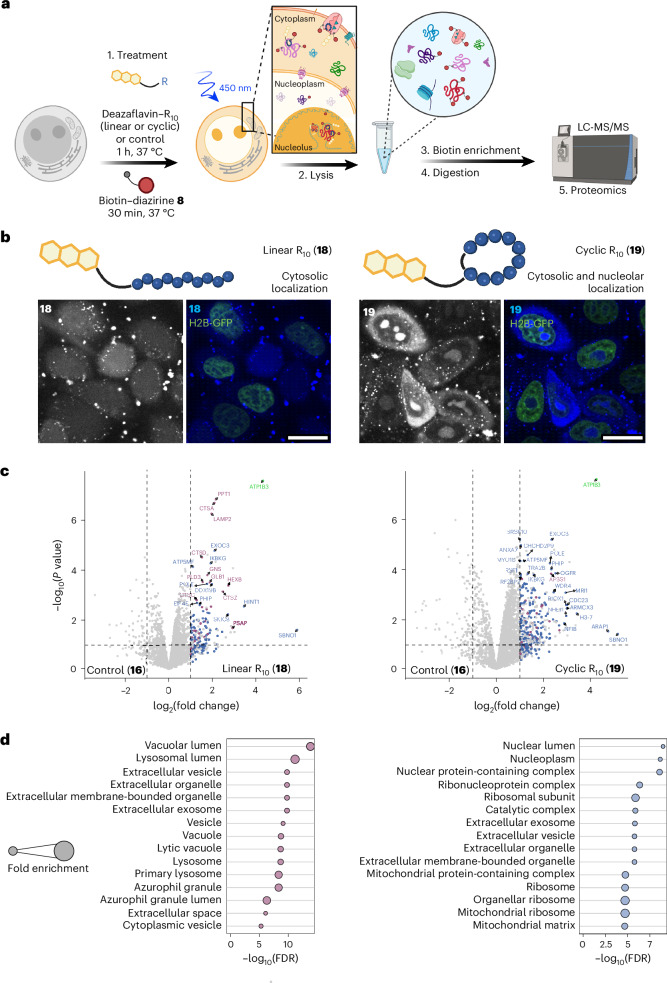
Mapping CPP interactomes via DarT labelling. **a**, Intracellular DarT labelling workflow for polyarginine CPP interactome mapping. **b**, Confocal microscopy images of HeLa cells stably expressing the histone 2B-green fluorescent protein (H2B-GFP) fusion protein treated with the deazaflavin–linear R_10_ conjugate **18** (20 µM) (left) and the deazaflavin–cyclic R_10_ conjugate **19** (20 µM) (right). Leftmost monochrome images show the fluorescence signal of **18** and **19** alone. Scale bars, 20 µm. **c**, Volcano plots from LFQ analysis of HeLa cells treated with **18** versus deazaflavin **16** (5 µM) (left) and with **19** versus deazaflavin **16** (5 µM) (right) for 1 h using biotin–diazirine **8** (250 µM) and 15 min of irradiation (450 nm). Significantly enriched proteins (*P* < 0.05 and log_2_(fold change) > 1) are coloured, with the most significantly enriched proteins from the cellular compartment GO term labelled relating to lysosome (purple), the nucleus (blue) and the membrane ion channel (green). *P* values were calculated through FragPipe-Analyst^78^ using an empirical Bayes moderation of two-sided *t*-statistics without further adjustments. Data were obtained from three independent replicates. **d**, GO analysis of significantly enriched proteins relating to cellular compartment for the deazaflavin–linear R_10_ conjugate **18** versus deazaflavin **16** (left) and the deazaflavin–cyclic R_10_ conjugate **19** versus deazaflavin **16** (right) datasets. Panel **a** created with BioRender.com.

CPPs have been widely utilized for the delivery of various functional extracellular cargoes into cells^61^. Polyarginine CPPs are among the most widely applied and studied type, although their specific modes of action and intracellular interacting partners are still under debate^62–64^. Diazirine-based photocrosslinking using UV light identified intra- and extracellular interactors of octaarginine (R_8_) CPPs, and we therefore questioned whether DarT labelling could be performed to label the interactomes of linear and cyclic decaarginine (R_10_) CPPs (Fig. 5a)^65,66^. Furthermore, the biocompatibility of deazaflavin **4** would enable studies over longer periods of time in living cells, which is not possible with cytotoxic iridium–CPP conjugates^32^. Recently, iridium–CPP conjugates have been reported for mapping intracellular trafficking pathways^67^, but uptake experiments were limited to 1 h as cytotoxic iridium complexes were used.

We started our investigation by attaching deazaflavin **12** to the N terminus of each R_10_ CPP and treated HeLa cells with the resulting conjugates (linear (**18**) and cyclic (**19**), 20 µM) for 1 h. Confocal microscopy confirmed the effective delivery and anticipated localization patterns of the deazaflavin–CPP conjugates (Fig. 5b), whereby the intrinsic fluorescence of the deazaflavin moiety (*λ*_em_ = 450 nm; Fig. 1b) was observed in the cytosol for both the linear (**18**) and the cyclic (**19**) derivatives, as well as in the nucleolus for **19**, similar to previous findings^68^. The subcellular localization of triethylene glycol deazaflavin **16** was similarly assessed (Supplementary Fig. 12), revealing weak cytosolic fluorescence signals with trace punctuated signals (most likely endo/lysosomes). Deazaflavin **16** was therefore chosen as a suitable control for further proteomic experiments to account for intracellular deazaflavin–protein interactors and to better resolve the R_10_-specific interactors.

To validate intracellular DarT labelling, cells were incubated with **18**, **19** or control **16** (5 µM, 1 h), and subsequently washed and treated with biotin–diazirine **8** (30 min, 250 µM) before irradiation at 450 nm for various illumination times. Western blots showed the observable biotinylation of intracellular proteins from 10 min of irradiation onwards (Supplementary Fig. 13), and fractionated lysates revealed clear trends in the biotinylated proteins that correlate to the confocal microscopy imaging (Supplementary Fig. 14). Cyclic derivative **19** elicited the highest degree of labelling across all of the fractions, especially within nuclear extracts when compared with linear derivative **18** and the control **16**.

We subsequently performed intracellular DarT labelling experiments for LFQ proteomic analysis after 1 h of incubation for the deazaflavin–CPPs or **16** (Fig. 5a). The volcano plots that were obtained showed a large number of significantly enriched proteins (log_2_(fold change) > 1) for both CPP conjugates, with linear derivative **18** displaying a number of significantly enriched lysosomal proteins, such as lysosomal protective proteins, cathepsins A and D (CTSA and CTSD, respectively), lysosome-associated membrane glycoprotein 2 (LAMP2) and palmitoyl-protein thioesterase 1 (PPT1) (Fig. 5c, left). On the other hand, cyclic R_10_ derivative **19** featured more proteins associated with the nucleus and nucleolus, such as strawberry notch homologue 1 (SBNO1), histone H3-7, PH domain-binding protein (PHIP) and WD repeat domain 4 (WDR4) (Fig. 5c, right).

GO enrichment analysis confirmed the trends in the enriched proteins to cellular compartments aligning with their expected subcellular localization (Fig. 5d). For example, linear derivative **18** displayed a high degree of endosomal and lysosomal enrichment (Fig. 5d, left), whereas cyclic derivative **19** featured primarily nuclear and nucleolus-associated proteins (Fig. 5d, right). A Venn diagram comparing the significantly enriched proteins from both CPPs revealed greater than three times the number of unique proteins associated with cyclic derivative **19** compared with linear derivative **18** due to the greater uptake efficacy of the cyclic derivative and prominent nucleolar localization (Supplementary Fig. 15). As both CPPs display cytosolic localization, we observed a large number of shared significantly enriched proteins (around 27%) that relate to lysosomal and granule-associated proteins (Supplementary Fig. 15c,d). Altogether, this demonstrates that DarT labelling enables the unbiased proteomic identification of R_10_-interacting proteins across multiple subcellular compartments, which aligns with the confocal microscopy images.

**Extended Data Fig. 7:**
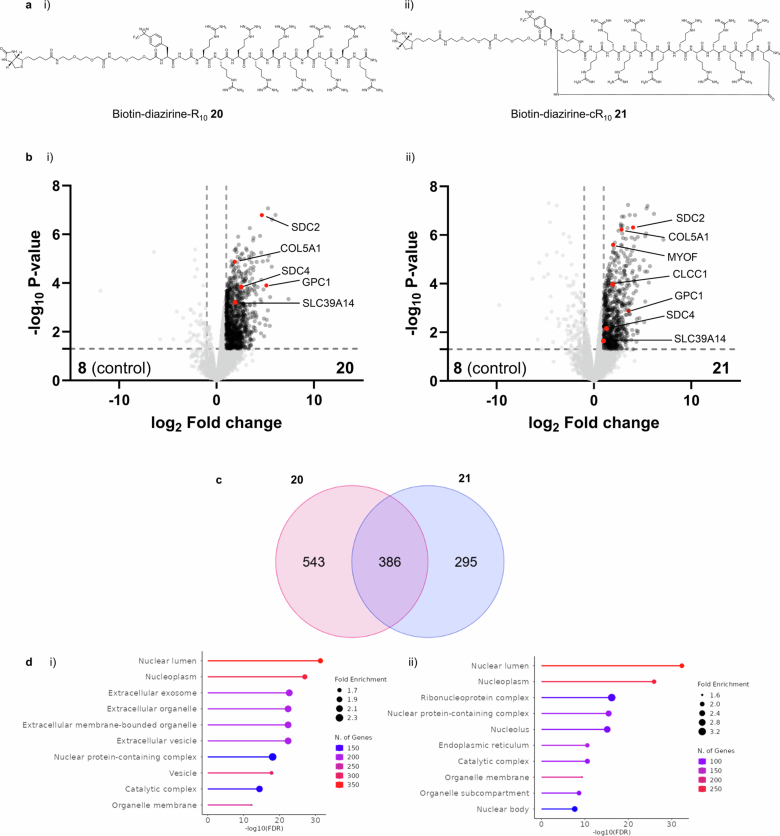
Protein photo-crosslinking of biotin-diazirine-R_10_ peptides using UV light. (**a**) Structures of (i) linear diazirine-R_10_ peptide **20** and (ii) cyclic diazirine-cR_10_ peptide **21**. (**b**) Volcano plots from LFQ analysis of HeLa cells treated with (i) **20** or (ii) **21** using diazirine **8** as the control with reported polyarginine interactors highlighted. P-values were calculated through FragPipe-Analyst using an empirical Bayes moderation of two-sided *t* statistics without further adjustments. Significantly enriched proteins are determined by P-value < 0.05 and log2 fold-change > 1. (**c**) Pie chart of shared and unique significantly enriched proteins from the datasets gathered. (**d**) GO enrichment plots of significantly enriched proteins from (i) linear diazirine-R_10_ peptide **20** and (ii) cyclic diazirine-cR_10_ peptide **21** photocrosslinking.

Previously, the UV-light activation of aryl diazirines incorporated into R_8_ CPPs identified protein interactors such as syndecan-4 (SDC4), a receptor for clathrin-mediated endocytosis^66^. Using a similar photocrosslinking peptide design (Extended Data Fig. 7a), we found that both linear R_10_ and cyclic R_10_ interact with SDC4 and other putative R_8_ interactors (Extended Data Fig. 7b)^66^. Owing to the global activation of diazirine probe moieties upon UV irradiation, a large number of proteins were significantly enriched for both peptides (929 for linear derivative **20** and 681 for cyclic derivative **21**; Extended Data Fig. 7c), given the same cut-off criteria as the DarT labelling experiments. However, we were unable to resolve the localization trends observed via microscopy after GO term enrichment for cellular compartments (Extended Data Fig. 7d). It is therefore difficult to delineate true interacting partners from false positives using this photocrosslinking method, whereas DarT labelling identified interactomes that were more specific to the subcellular location of the CPPs (Fig. 5d).

We also observed the significant enrichment of a cell membrane protein common to both linear and cyclic R_10_ datasets that could be involved in the uptake of the R_10_ CPPs. ATPase Na^+^/K^+^-transporting subunit beta 3 (ATP1B3; green dot, Fig. 5c) is an integral membrane protein that is responsible for electrochemical Na^+^/K^+^ gradients, osmoregulation and the sodium-coupled transport of various organic and inorganic solutes^69,70^. It is ranked within the top 25% of abundant proteins within the cell line used (HeLa; Supplementary Fig. 16) and has recently been identified as a key regulator of TAT CPP cellular uptake^71^. We therefore hypothesize that this ion pump is involved in regulating the uptake of R_10_ CPPs regardless of their structure, and future work will aim to biologically validate these findings.

### DarT labelling in live cells after 24 h

Recognizing the high biocompatibility of deazaflavin **4**, we applied DarT labelling to long-term incubation experiments to map the dynamic intracellular events of CPPs. Confocal microscopy of fluorescent linear and cyclic R_10_ peptides after 24 h post-treatment reveals stark differences in cellular clearance. The linear derivative undergoes lysosomal degradation and peptidolysis (Supplementary Fig. 17), whereas the cyclic derivative persists within intracellular vesicles with some nucleolar localization (Supplementary Fig. 17). The latter can be rationalized by resistance to peptidolysis due to its cyclic nature and the incorporation of d-amino acids into the R_10_ sequence^72^. With this in mind, we questioned whether DarT labelling can be used to map interactors of the cyclic R_10_ derivative **19** after 24 h, highlighting its suitability towards mapping dynamic intracellular trafficking^73,74^.

**Fig. 6:**
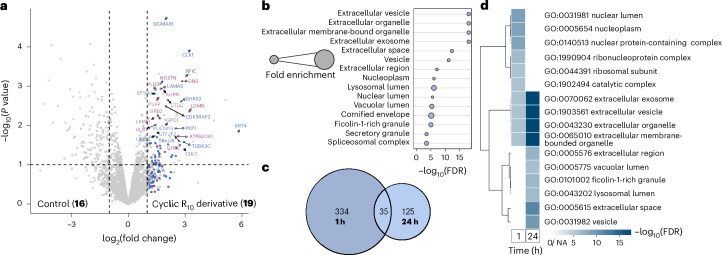
Mapping the cyclic R_10_ interactome after 24 h. **a**, Volcano plot from LFQ analysis of HeLa cells treated with the deazaflavin–cyclic R_10_ conjugate **19** versus deazaflavin **16** (5 µM) 24 h post-treatment using biotin–diazirine **8** (250 µM) and 15 min of irradiation (450 nm). Significantly enriched proteins (*P* < 0.05 and log_2_(fold change) > 1) are coloured, with the most significantly enriched proteins from the cellular compartment GO term labelled relating to lysosome (purple) and nucleus (blue). *P* values were calculated through FragPipe-Analyst^78^ using an empirical Bayes moderation of two-sided *t*-statistics without further adjustments. Data were obtained from three independent replicates. **b**, Cellular compartment GO terms of significantly enriched proteins relating to **19** versus control **16** at 24 h post-treatment. **c**, Venn diagram comparing the significantly enriched proteins from DarT labelling after 1 h incubation and 24 h post-treatment of **19**. **d**, Clustered heatmap of cellular compartment GO terms after 1 h incubation and 24 h post-treatment of **19**.

We therefore treated cells with **19** for 1 h, added fresh media and performed intracellular DarT labelling after 24 h. LFQ analysis of the proteomic data provided a volcano plot that revealed a number of enriched proteins found in exosomes and lysosomes, such as glucosamine (*N*-acetyl)-6-sulfatase (GNS), lysosome-associated membrane glycoprotein 1 (LAMP1), prosaposin (PSAP) and cathepsin C (CTSC) (Fig. 6a). Notably, we only detected significant enrichment of intravesicular and transmembrane proteins associated with exosomes and lysosomes. Other typical intracellular vesicle markers, such as Rab proteins, were not significantly enriched (log_2_(fold change) < 1). This implies that the conjugate is entrapped within the vesicle and does not reside outside the membrane.

GO analysis of the enriched proteins in relation to their cellular compartment consolidates these findings, displaying a significant enrichment of exosome, granule and lysosome-associated proteins, as well as some nucleus-associated proteins (Fig. 6b). These findings align with the confocal microscopy images of fluorescent cyclic R_10_ peptides (Supplementary Fig. 17), which show the loss of nuclear and nucleolar fluorescent signals over time with pronounced localization within lysosomes. This indicates that the CPP undergoes lysosomal exocytosis, a common cellular process for maintaining homeostasis and reducing the accumulation of toxic products, such as heavy metals or aggregated proteins^75^.

To gain further evidence of this phenomenon, we compared the overall number of proteins enriched after 1 h incubation and 24 h post-treatment (Fig. 6c). More than half the number of proteins were enriched after 24 h when compared with the 1 h treatment, which aligns with the lysosomal exocytosis of internalized cyclic R_10_ **19** over time. Lastly, comparing the enriched GO terms from 1 h incubation and 24 h post-treatment (Supplementary Fig. 18) and subsequently clustering the enrichment FDR (that is, the false discovery rate of the adjusted *P* value) of the terms, enabled us to better observe the trafficking of the peptide (Fig. 6d). A clear decrease in nuclear and nucleolus-associated proteins is apparent, coupled with an increase in lysosomal and exocytosis-associated proteins, further underlining the lysosomal exocytosis of cyclic R_10_ derivative **19**.

## Conclusions

In summary, we have demonstrated that deazaflavin **4** is a readily accessible organic photocatalyst that is capable of activating diazirine probes via triplet energy transfer to enable the labelling of both extra- and intracellular proteins in live cells for precise interactome mapping (termed DarT labelling). Furthermore, photoexcited deazaflavin **4** undergoes energy transfer with aryl azides and electron transfer with phenols. We applied this multifaceted reactivity to the in vitro photolabelling of proteins, showing efficacies similar to those of other reported photocatalysts across diverse probe types.

Most notably, deazaflavin **4** offers excellent intrinsic cell permeability, biocompatibility and minimal phototoxicity when compared with cell-permeable iridium photocatalysts. This enabled the use of deazaflavin conjugates for live-cell diazirine-based intracellular protein labelling with extended incubation times. Moreover, benchmarking DarT labelling to iridium-based PPL through extracellular surfaceome mapping revealed similar enrichment of the target protein, with the total number of cell-surface proteins enriched correlating to the in vitro labelling efficacy.

We demonstrated intracellular DarT labelling by probing the interactome of linear and cyclic arginine-rich CPPs across multiple subcellular compartments. LFQ proteomic analysis resulted in a number of significantly enriched proteins, for both CPPs, that correlated with their expected localization as visualized by the intrinsic fluorescence of **4**. Further GO enrichment revealed the classification of proteins by cellular compartment, confirming that endosomal and lysosomal proteins were primarily targeted by linear CPPs, whereas the cyclic derivatives predominantly interacted with nucleolar proteins. By contrast, the UV-light activation of diazirine-containing R_10_ peptides to directly crosslink interacting proteins provided a greater number of significant hits but lacked the spatial resolution of enriched proteins. Owing to fundamental differences in protein labelling mechanisms, a direct comparison of these techniques is difficult, and should be considered complementary for thorough interactome profiling. Using DarT labelling, we were able to identify the interaction of both CPPs with the Na^+^/K^+^ ion channel ATP1B3, which has previously been proposed to regulate TAT peptide uptake into cells^71^, suggesting that this ion channel is more generally associated with arginine-rich CPP-uptake mechanisms.

Finally, we investigated the intracellular fate of the cyclic R_10_ derivative, supporting the use of DarT labelling to map live-cell trafficking after extended time points. After 24 h post-treatment of the cyclic CPP, we enriched proteins related to exosomes, granules and lysosomes, pointing towards exocytosis of the undigested cyclic R_10_. There are only a few reports regarding the intracellular fate of such species, and more knowledge concerning these factors could enhance their translation to biopharmaceutical applications^76,77^. This further highlights the excellent biocompatibility of deazaflavin **4** and its ability for diazirine-based intracellular PPL with high spatio-temporal control.

Overall, DarT labelling offers a powerful and precise method for dynamic interactome mapping, to aid our understanding of essential intracellular mechanisms and elucidate complex disease-related biomolecular networks. At the current stage, a key consideration of the technology is the balance between photostability of the deazaflavin catalyst and photocatalytic activation of the labelling probe. Improving catalyst photostability would thereby enhance the resolution and elucidation of low-abundance or highly dynamic protein–protein interactions, which is currently under investigation. Furthermore, given the toxicity and limited tissue penetration of blue light, further development of benign organic photocatalysts that absorb low-energy wavelengths (>600 nm) would enable proximity labelling within disease-specific tissues, organoids and living organisms.

## Methods

### Chemical synthesis

Details of the synthesis and characterization of all photocatalysts, probes and peptides are given in Supplementary Section 3.1.

### Diazirine conversion and analysis via ^19^F NMR

To an 8 ml crimp neck vial with a magnetic stirrer bar was added a solution of diazirine (100 µM, 10 µl of 10 mM DMSO stock) and photocatalyst (10 µM, 10 µl of 1 mM DMSO stock) in PBS (pH = 7.4, 1 ml). The vial was sealed and sparged with Ar for 10 min before being irradiated with 450 nm light (30 W LED; EvoluChem, PhotoRedOx Box) for 15 min. The solution was then spiked with *p*-(trifluoromethyl)benzoic acid as the internal standard (100 µM, chemical shift (*δ*) = −61.35 ppm) and analysed via ^19^F NMR to determine the conversion of diazirine (*δ* = −64.23 ppm) to the carbinol product (*δ* = −76.64 ppm)^17^. Conversions are represented as the average of duplicate runs.

### Stationary and time-resolved absorption and emission spectroscopy

Details of the stationary and time-resolved absorption and emission spectroscopy procedures used for the mechanistic studies are given in Supplementary Section 3.3.

### Western blot procedure

Gel electrophoresis was performed using a Bio-Rad Mini-PROTEAN Tetra Vertical Electrophoresis Cell, a Bio-Rad PowerPac Basic Power Supply and 4–20% Mini-PROTEAN TGX Precast Protein Gels. After electrophoresis (typically 35 min at 200 V), the protein bands were transferred to polyvinylidene fluoride (PVDF) membranes (Bio-Rad Trans-Blot Turbo Transfer Pack) using a Bio-Rad Trans-Blot Turbo Transfer System. The membranes were washed with water and the total protein loading was measured using No-Stain Protein Labelling Reagent (Invitrogen) or 0.5% Ponceau S (Sigma-Aldrich) and 1% acetic acid solution for 10 min. Excess stain was decanted, and the membranes were washed with water before imaging with a Bio-Rad ChemiDoc MP System and Image Lab software. The membranes were washed with Tris-buffered saline (TBST: 10 mM Tris, 150 mM NaCl, 0.1% w/v Tween-20, 3 × 10 min), immersed in 5% BSA (biotin-free) in TBST blocking buffer and incubated for 1 h at room temperature. The blocking solution was discarded, and a solution of primary antibody (1:1,000) in fresh blocking buffer was added. The membrane was incubated for 18 h at 4 °C with gentle rocking. The membranes were then washed with TBST (3 × 10 min), and a solution of secondary antibody-HRP conjugate (1:10,000) in blocking buffer was added and rocked for 1 h at room temperature. Membranes were then finally washed with 1× TBTS (3 × 10 min) and developed using SuperSignal West Pico PLUS Chemiluminescent Substrate solutions (Thermo Fisher) and imaged using the Bio-Rad ChemiDoc MP System with Image Lab software. For analysing biotinylated proteins, streptavidin-HRP (Abcam, 1:5,000) in blocking buffer was added to freshly blocked membranes and imaged as described above.

### General procedure for in vitro protein photolabelling

The photocatalyst (10 µM) and biotin probe (**8**, **9**, **10**, **11** or **S4**) (100 µM) were added to a solution of BSA (Merck), carbonic anhydrase (bovine, Merck) or HSA (recombinant albumin, New England Biolabs) in PBS (10 µM protein, 100 µl total volume). The solutions were then irradiated using 450 nm light (30 W LED, EvoluChem PhotoRedOx Box) for 5–30 min at room temperature (maintained with a cooling fan). Samples without photocatalyst were irradiated with 365 nm light (18 W LED, EvoluChem PhotoRedOx Box). For gel and western blot analysis, samples (30 μl) were removed, mixed with 10 μl aliquots of 4× Laemmli sample buffer (containing 5% ß-mercaptoethanol) and heated at 70 °C for 10 min. Each prepared sample (10 μl) was loaded onto a precast SDS–PAGE (sodium dodecyl sulfate–polyacrylamide gel electrophoresis) gel and subsequently analysed via western blot as described above. For LC-MS/MS analysis, samples (total 20 µg protein) were taken for SP3 (single-pot, solid-phase-enhanced sample-preparation) digestion as described below.

### LC-MS/MS analysis protein photolabelling

Photolabelled HSA was digested using the SP3 protocol^79^. Proteins (20 µg, 30 µl from the above solutions) were denatured with 4% SDS, reduced with 5 mM tris(2-carboxyethyl)phosphine (TCEP) and alkylated with 40 mM chloroacetamide (final volume of 96 µl) for 1 h at room temperature. Sera-Mag beads (4 µl from 50 mg ml^−1^ stock, Cytiva) were added to the sample, followed by 50% acetonitrile. After a brief incubation, the supernatants were removed and the beads were washed twice with ethanol (200 µl) and once with acetonitrile (200 µl). Proteins were digested in 50 mM triethylammonium bicarbonate buffer (200 µl, pH = 8.5) with Lys-C and trypsin proteases (both in an enzyme:protein ratio of 1:50 (wt:wt)) for 16 h at 37 °C. Beads were washed twice with an excess of acetonitrile (200 µl). Peptides were eluted with 5% DMSO (200 µl) and dried until further use.

Details of the LC-MS/MS analysis procedure are given in Supplementary Section 2.9.

The obtained raw data were searched against a FASTA database (version 36) containing the HSA sequence and common contaminants with the following settings: precursor mass tolerance: ±10 ppm; fragment mass tolerance: ±20 ppm; mass calibration and parameter optimization enabled; isotope error: 0/1/2; enzyme: trypsin (cuts after K and R, no cut before P), peptide length: AA 5–50; peptide mass range: 500–5,000 Da; and variable modifications: oxidation (M, 15.9949 Da, up to 3×), acetylation (N terminal, 42.0106 Da), diazirine probe **S4** or **8** (on all amino-acid residues, 229.0726 Da, or 660.2818 Da, respectively, up to 3×), carbamidomethylation (C, 57.02146 Da). The post-translational modification (PTM) site localization probability threshold was set at >0.75. Validation was performed using Percolator^80^ and ProteinProphet^81^ software and an FDR of 1% was applied. IonQuant^82^ software was used for LFQ, with match-between-runs (MBR; FDR of 1%) and normalization across the runs enabled. Subsequent data processing was carried out using RStudio v2024.04.2. The PTM site tables were filtered to retain only HSA entries, while contaminants were excluded. A PTM site was considered identified if its intensity was greater than zero. Coverage was calculated as the percentage of unique, identified modified amino acids in HSA relative to the total number of available amino acids in HSA.

### Antibody-based cell-surface protein labelling

The IgG-**4** and Tra-**4** antibody conjugations were prepared as described in Supplementary Section 3.2. For the microscopy experiments, 1.5 × 10^5^ cells per well were seeded in an eight-well glass-bottom slide (IBIDI) and were incubated for 24 h. For all washing steps, cold PBS containing 1 mM CaCl_2_ and 0.5 mM MgCl_2_ was used, and for each treatment or washing, the default volume used was 200 µl.

Cells were washed and incubated with anti-HER2 antibody (10 µg; Invitrogen MA513105) or Mouse IgG Isotype (Invitrogen P3.6.2.8.1) in DMEM growth medium F-12 with 1% fetal calf serum (FCS) for 30 min at 4 °C. Cells were then washed twice, followed by incubation with IgG-**4** (10 µg) for 30 min at 4 °C. For direct binding experiments, cells were treated as described with Tra-**4** or unmodified Tra. Treated cells were washed twice and treated with biotin–diazirine **8** (250 µM) in PBS (diluted from 100 mM DMSO stock), followed by irradiation with 450 nm light (30 W LED; EvoluChem, PhotoRedOx Box) in a cold room (4 °C) for 10 min. After irradiation, the cells were washed twice and incubated with streptavidin–Alexa Fluor 488 conjugate (5 µg; Invitrogen) for 30 min at room temperature. Cells were counterstained with 10 µg ml^−1^ Hoechst 33342 (Invitrogen) in FluoroBrite DMEM (Gibco) for 10 min and imaged in FluoroBrite DMEM. Imaging was performed using a Zeiss LSM780 confocal microscope with a ×40, 1.4-NA Plan-Apochromat oil immersion objective at room temperature. Image analysis and processing was performed using FIJI software (version 2.16.0/1.54p).

### Recombinant HaloTag self-labelling and diazirine photolabelling

To a 1.5 ml Eppendorf tube was added PBS (7.36 µl), recombinant HaloTag (10 µM, 3.64 µl of 55 µM stock in 20 mM HEPES, 150 mM NaCl) followed by either deazaflavin–chloroalkane conjugate **13** (45 µM, 9.00 µl from 100 µM stock in 1% DMSO in PBS) or iridium catalyst–chloroalkane conjugate **14** (45 µM, 9.00 µl from 100 µM stock in 1% DMSO in PBS). The mixture was incubated at 25 °C for 1 h and analysed via high-resolution mass spectrometry (quadrupole time-of-flight) to confirm self-labelling (as shown in Supplementary Fig. 9). Samples were then buffer exchanged to PBS using ZebaSpin 7000 MWCO Micro Spin Columns according to the manufacturer’s procedure (12 µl + 3 µl stacking solvent). Self-labelled HaloTag–photocatalyst conjugates (5 µM, 7.5 µl of 10 µM stock in PBS) were then added to a solution of biotin–diazirine **8** (100 µM, 7.5 µl of 200 µM stock in DPBS solution) and irradiated for 15 min at 450 nm using the EvoluChem PhotoRedOx Box. Control samples containing free photocatalysts deazflavin **12** or iridium **3a** (5 µM, 0.75 µl from 100 µM stocks in 1% DMSO in DPBS) were prepared with unlabelled HaloTag (5 µM, 1.36 µl of 55 µM stock in 20 mM HEPES, 150 mM NaCl), biotin–diazirine **8** (100 µM, 7.5 µl of 200 µM stock in PBS) in PBS (15 µl total volume), and irradiated as described. All samples were diluted with 4× Laemlli buffer with β-mercaptoethanol (to 5 µl) and heated at 70 °C for 10 min. The samples were cooled to room temperature, briefly centrifuged and analysed via western blots following the protocol above.

### Extracellular DarT labelling

HeLa cells were transfected according to Supplementary Section 2.8. HeLa cells (untransfected) and HaloTag-mGluR2-expressing HeLa cells (transfected) were seeded onto 6 cm cell culture dishes and were allowed to reach 90% confluency. The dishes were cooled at 4 °C before the assay. Cells were washed twice with PBS before being treated with 5 µM **12** or **13** in HBSS at 23 °C for 30 min. The treatment solution was discarded, and the cells were washed with PBS before the addition of biotin–diazirine **8** (250 µM) in HBSS (diluted from 100 mM DMSO stock). The dishes were immediately transferred to the cold room (4 °C) and irradiated as stated in Supplementary Section 2.1 for 15 min. Irradiated cells were placed on ice and washed twice with ice-cold PBS, making sure to aspirate out all residual liquid. Cell lysates were made by adding radioimmunoprecipitation assay (RIPA) buffer (200 µl; 50 mM Tris–HCl pH = 7.6, 150 mM NaCl, 1 mM EDTA, 1% Triton X-100, 0.1% SDS and 0.1% sodium deoxycholate) with 1× cOmplete Protease Inhibitor (Roche) and 1× Benzonase Nuclease (Merck) to each plate and subsequent harvesting of the cells by scraping. Lysates were collected into pre-chilled 1.5 ml microcentrifuge tubes and incubated at room temperature (~25 °C) for 15 min in a table-top shaker. The lysates were centrifuged at 12,000 × *g*, 5 min, 4 °C, and the supernatants were transferred into new pre-chilled 1.5 ml microcentrifuge tubes. Total protein concentrations were determined via BCA protein assay (Pierce, Thermo Fisher) according to the manufacturer’s specifications. Lysates were diluted to 2 mg ml^−1^ with 8 M urea before flash-freezing with liquid nitrogen, and storage at −80 °C until used for subsequent experiments.

### Pulldown and proteomic workflow

Pulldowns were performed using a modified SP2E protocol.^83^ Combined hydrophobic and hydrophilic carboxylate-coated magnetic beads (1:1, 50 mg ml^−1^, Sera-Mag, Cytiva) were washed thrice with water (1 ml). Lysates (500 µg) were transferred directly onto the washed combined hydrophobic and hydrophilic carboxylate-coated magnetic beads (100 µl), and 100% ethanol was added to a final volume of 2 ml (note that the final ethanol concentration must be >60% to ensure protein precipitation onto the beads). After resuspending the beads by vortexing, the suspension was incubated for 5 min at room temperature on a table-top shaker at 950 revolutions per min. The beads were washed thrice with 80% ethanol in water (1 ml) using a magnetic rack, and the proteins were eluted separately via the addition of 0.2% SDS in PBS (250 µl). This procedure was repeated twice, and the supernatants were combined (to a total of 500 µl) and added to a 50 μl volume of equilibrated streptavidin-coated magnetic beads (three times prewashed with 0.2% SDS in PBS (1 ml), New England Biolabs), followed by incubation for 2 h at 23 °C and 950 revolutions per min. The bead mixture was washed thrice with 0.1% NP-40 detergent in PBS (1 ml), twice with 6 M urea (1 ml) and twice with water (1 ml) on a magnetic rack. The washed bead mixtures were resuspended in 125 mM ammonium bicarbonate buffer (56 μl) and transferred to new tubes before reducing and alkylating the proteins by the addition of 100 mM TCEP (7 μl) and 400 mM chloroacetamide (7 μl), followed by incubation for 5 min at 95 °C. The proteins were digested overnight at 37 °C with sequencing grade trypsin (1 μl; 0.5 mg ml^−1^, Promega). The beads were placed on a magnetic rack for 30 min, and the supernatants were collected separately and acidified with formic acid (1 μl). Before proceeding, a quality-control run was conducted using a XEVO G2-XS QToF instrument (Waters) to ensure complete digestion. The samples were then diluted based on this to ensure equal loading.

LC-MS/MS analysis for the pulldown studies was performed as described in Supplementary Section 2.9; however, fragment ion spectra were acquired in the linear ion trap in rapid mode.

The obtained raw data were analysed using FragPipe (v22.0) using the built-in LFQ-MBR workflow using the whole human proteome as the search space and applying the following MSFragger^84^ settings: precursor mass tolerance: ±20 ppm; fragment mass tolerance: ±20 ppm; mass calibration and parameter optimization enabled; isotope error: 0/1/2; enzyme: trypsin (cuts after K and R, no cut before P), peptide length: AA 7–50; peptide mass range: 500–5,000 Da; variable modifications: oxidation (M, 15.9949 Da, up to 3×), acetylation (N terminal, 42.0106 Da); carbamidomethylation of C was set as fixed modification (57.02146 Da). Validation was performed using Percolator^80^ and ProteinProphet^81^, and an FDR of 1% was applied. IonQuant^82^ was used for LFQ, with MBR (FDR of 1%) and normalization across the runs enabled. The analysed files were further processed using FragPipe-Analyst^78^ as follows: proteins must be quantified in >66% of the files for at least one condition and in >66% of the files globally. Median-centred normalization of the MaxLFQ intensity extracted from the FragPipe results was enabled, and Perseus-type imputation and Benjamini–Hochberg-type FDR correction were enabled. Unless stated otherwise, the following significance cut-offs were used: log_2_(fold change) > 1; *P* < 0.05.

GO cellular component enrichment analysis was carried out using the default settings in ShinyGO 0.80 (ref. ^85^), importing only significantly enriched proteins from either of the two conditions against a complete human background. Both the Venn diagram and volcano plots were created using RStudio v2024.04.2 and the packages EnhancedVolcano and VennDiagram. The clustered GO term heatmap was obtained by extracting separately the top ten enriched GO cellular component terms with the lowest adjusted *P* value from ShinyGO 0.80 against a human background. The adjusted *P* value (‘enrichment FDR’) was log-transformed and visualized in R using the pheatmap package with row clustering enabled.

### Cell viability assay

The effect on cell viability of the photocatalysts iridium **3a** and **3b**, deazaflavin **4** and EY was determined using the commercially available WST-1 assay (Takara, MK400). Cells were seeded into clear 96-well plates (Corning) containing 5,000 cells per well in growth medium (100 µl, DMEM high glucose with 10% FCS) and cultured for 24 h at 37 °C and 5% CO_2_. Subsequently, the cells were treated with various concentrations of photocatalysts (0.01–100 μM) diluted in growth media containing 1% DMSO. After a further 24 h of incubation at 37 °C and 5% CO_2_, WST-1 reagent (10 µl) was added to each well and incubated at 37 °C, 5% CO_2_ for 0.5–2 h, according to the manufacturer’s instructions. The absorbance of each well was measured at 440 nm using a plate reader (Spark, Tecan). Control experiments were performed using cells with just DMEM, cells with DMEM containing 1% DMSO, cell-free culture media (blank), 10 µM staurosporine and cell-free sample dilutions in culture media to evaluate potential sample interferences with the WST-1 assay. All experiments were conducted in biological triplicate. The percentage cell viability was calculated according to equation (1) and normalized to 1% DMSO (100%) and staurosporine controls (0%):

1 $$\begin{array}{l}{\rm{Cell}}\;{\rm{viability}}\left(\%\right)\\=100x\left(\displaystyle\frac{{\rm{Absorbance}}\; {\rm{of}}\; {\rm{treated}}\; {\rm{cells}}-{\rm{Absorbance}}\; {\rm{of}}\; {\rm{blank}}}{{\rm{Absorbance}}\; {\rm{of}}\; {\rm{control}}-{\rm{Absorbance}}\; {\rm{of}}\; {\rm{blank}}}\right).\end{array}$$

### Phototoxicity assay

Cells were seeded into black 96-well plates with a clear bottom (Corning) containing 5,000 cells per well in growth medium (100 µl, DMEM high glucose/10% FCS) and cultured for 24 h at 37 °C and 5% CO_2_. The next day, the growth medium was removed, and cells were treated with iridium catalyst **3a**, iridium catalyst **3b**, deazaflavin **4** or EY (at either 1 or 10 μM) diluted in growth medium containing 1% DMSO. The treated cells were incubated in the dark for 1 h at 37 °C and 5% CO_2_. The cells were then washed with FluoroBrite DMEM, and fresh FluoroBrite DMEM (100 µl) was added. Plates were then illuminated with 450 nm LED light as described in Supplementary Section 2.1 for 5, 10 or 15 min, or incubated further in the dark for 15 min (dark control). After irradiation, fresh growth medium was added to the cells, and they were incubated overnight at 37 °C and 5% CO_2_. Subsequently, WST-1 reagent (10 µl) was added to each well and incubated at 37 °C and 5% CO_2_ for 0.5–2 h the absorbance of each well was then measured at 440 nm according to the protocol above. Control experiments were performed using cells with just DMEM, cells with DMEM containing 1% DMSO, cell-free culture media (blank) and 10 µM staurosporine. All experiments were conducted in biological triplicates. The percentage cell viability was calculated according to equation (1) and normalized to 1% DMSO (100%) and staurosporine controls (0%).

### Modified chloroalkane penetration assay

CA–TMR-d_12_ and BG–SiR-d_12_ were prepared as described previously^59^. HeLa or HEK293T cells were seeded onto 10 cm cell culture dishes and allowed to attach for 24 h under standard cell culture maintenance conditions. The culture medium was replaced with fresh DMEM (5 ml, high glucose) with 10% FCS without antibiotics followed by plasmid (pcDNA5-HaloTag-Pro30-SNAP-tag) transfection using Lipofectamine 3000 (Thermo) according to Supplementary Section 2.8. After 24 h, the transfected cells were trypsinized, seeded into 24-well cell culture plates (1 × 10^5^ cells per well) and incubated again for 24 h. Reporter cells were washed twice with PBS, followed by treatment with 5 µM **16** (negative control), 5 µM CA–PEG_2_-NHBoc (positive control) and 5,000, 1,000, 200, 40, 8 or 1.2 nM **15** or **17** in FluoroBrite DMEM (diluted from 10 mM DMSO stocks) for 1 h, with 5 µM CA–TMR-d_12_ for 15 min and with 1 µM BG–SiR-d_12_ (diluted from 10 mM DMSO stocks) in FluoroBrite DMEM for 15 min; washing steps were carried out in between treatments. Treated reporter cells were trypsinized, collected in 1.5 ml microcentrifuge tubes, washed twice with PBS and pelleted via centrifugation (800 × *g*, 5 min, 4 °C). Cell pellets were resuspended in PBS (400 µl) and analysed via flow cytometry (FACS Fortessa). The mean fluorescence intensity (MFI) values in the TMR and Cy5 (SiR) channels of live cells were determined. The MFI of SiR was used as an expression normalization control for all measurements. Equation (2) below was used to determine the percentage fluorescence intensity:

2 $$\begin{array}{l}{\rm{Fluorescence}}\;{\rm{intensity}}\;\left( \% \right)\\=\displaystyle\frac{({{\rm{photocatalyst}}-{\rm{chloroalkane}}{\rm{MFI}}}_{{\rm{TMR}}})-({\rm{photocatalyst}}{\rm{MFI}}_{{\rm{TMR}}})}{({{\rm{CA}}-{\rm{NHBoc}}{\rm{MFI}}}_{{\rm{TMR}}})-({\rm{photocatalyst}}{\rm{MFI}}_{{\rm{TMR}}})}\times 100.\end{array}$$

For microscopy imaging, transfected HeLa cells were seeded onto eight-well glass-bottom slides (IBIDI). Tile-scan images were captured using a Leica LSM780 confocal laser scanning microscope with a ×40, 1.4-NA Plan-Apochromat oil immersion objective.

### Intracellular DarT labelling

HeLa cells were seeded onto 10 cm dishes and were allowed to attach for 48 h or until 80% confluence. Cells were washed twice with PBS before treating with 5 µM **16**, **18** or **19** in serum-free FluoroBrite DMEM (diluted from 10 mM DMSO stocks) at 37 °C for 1 h. The treatment solution was discarded and the cells were washed with PBS before the addition of biotin–diazirine **8** (250 µM) in FluoroBrite DMEM (diluted from 100 mM DMSO stock) and further incubation for 30 min. The dishes were transferred to the cold room and irradiated as stated in Supplementary Section 2.1 for 15 min. Irradiated cells were placed on ice and washed twice with ice-cold PBS, making sure to aspirate out all residual liquid. Cell lysates were made by adding RIPA buffer (500 µl; 50 mM Tris–HCl (pH = 7.6), 150 mM NaCl, 1 mM EDTA, 1% Triton X-100, 0.1% SDS and 0.1% sodium deoxycholate) with 1× cOmplete Protease Inhibitor and 1× Benzonase Nuclease to each plate and scraping the cells. Lysates were collected into pre-chilled 1.5 ml microcentrifuge tubes and incubated at room temperature (~25 °C) for 15 min in a table-top shaker. The lysates were centrifuged at 12,000 × *g*, 5 min, 4 °C. The supernatants were transferred into new pre-chilled 1.5 ml microcentrifuge tubes, flash frozen with liquid nitrogen and stored at −80 °C until used for subsequent experiments. Biotin pulldown and proteomic analyses were performed as stated above.

### Photocrosslinking with biotin–diazirine–R_10_ conjugates

HeLa cells were seeded onto 10 cm dishes and were allowed to attach for 48 h or until 80% confluence. Cells were washed twice with PBS before being treated with 5 µM **20**, **21** or **8** (control) in HBSS at 37 °C for 1 h. The dishes were irradiated with 365 nm light (LED Cube 100 IC, Dr. Hönle) for 10 s. Subsequent washings, lysis, biotin pulldown and proteomic analyses follow the procedures above.

### Statistical information

All biological experiments were performed in replicates as stated in the figure legends. The statistical tests and values of *n* are reported in the figure legends where appropriate. Graphs, CP_50_ values and statistical significance data were determined using GraphPad Prism 10.

### Reporting summary

Further information on research design is available in the Nature Portfolio Reporting Summary linked to this article.

## Online content

Any methods, additional references, Nature Portfolio reporting summaries, source data, extended data, supplementary information, acknowledgements, peer review information; details of author contributions and competing interests; and statements of data and code availability are available at 10.1038/s41557-025-01931-8.

## Supplementary information

Supplementary Information Supplementary Figs. 1–18, Discussion, General information, Experimental procedures and Source data for Supplementary Figs. 13 and 14.

Reporting Summary

## Source data

Source Data Fig. 2 Unprocessed western blots.

Source Data Fig. 2 and Source Data Extended Data Fig. 2 Band intensity quantitative representation.

Source Data Fig. 3 Unprocessed western blots.

Source Data Extended Data Fig. 2 Unprocessed western blots.

## Data Availability

All experimental data, materials and methods, analytical procedures, cell assays and copies of spectra are available in the Article and its Supplementary Information. The mass spectrometry proteomics data have been deposited by the PRIDE partner repository and are available via the ProteomeXchange Consortium with the dataset identifier PXD056778. Source data are provided with this paper.

## References

[1] Chen, L., Wang, R.-S. & Zhang, X.-S. *Biomolecular Networks: Methods and Applications in Systems Biology* (Wiley, 2009); 10.1002/9780470488065

[2] Bludau, I. & Aebersold, R. Proteomic and interactomic insights into the molecular basis of cell functional diversity. *Nat. Rev. Mol. Cell Biol.* **21**, 327–340 (2020). 32235894 10.1038/s41580-020-0231-2

[3] Kang, M.-G. & Rhee, H.-W. Molecular spatiomics by proximity labeling. *Acc. Chem. Res.* **55**, 1411–1422 (2022). 35512328 10.1021/acs.accounts.2c00061 PMC9118551

[4] Bosch, J. A., Chen, C.-L. & Perrimon, N. Proximity-dependent labeling methods for proteomic profiling in living cells: an update. *Wiley Interdiscip. Rev. Dev. Biol.* **10**, e392 (2020). 32909689 10.1002/wdev.392 PMC8142282

[5] Fang, Y. & Zou, P. Photocatalytic proximity labeling for profiling the subcellular organization of biomolecules. *ChemBioChem* **24**, e202200745 (2023). 36762434 10.1002/cbic.202200745

[6] Knutson, S. D., Buksh, B. F., Huth, S. W., Morgan, D. C. & MacMillan, D. W. C. Current advances in photocatalytic proximity labeling. *Cell Chem. Biol.* **31**, 1145–1161 (2024). 38663396 10.1016/j.chembiol.2024.03.012 PMC11193652

[7] Oakley, J. V. et al. Radius measurement via super-resolution microscopy enables the development of a variable radii proximity labeling platform. *Proc. Natl Acad. Sci. USA* **119**, e2203027119 (2022). 35914173 10.1073/pnas.2203027119 PMC9371666

[8] Buksh, B. F. et al. μMap-Red: proximity labeling by red light photocatalysis. *J. Am. Chem. Soc.* **144**, 6154–6162 (2022). 35363468 10.1021/jacs.2c01384 PMC9843638

[9] Tay, N. E. S. et al. Targeted activation in localized protein environments via deep red photoredox catalysis. *Nat. Chem.* **15**, 101–109 (2023). 36216892 10.1038/s41557-022-01057-1 PMC9840673

[10] Ryu, K. A. et al. Near-infrared photoredox catalyzed fluoroalkylation strategy for protein labeling in complex tissue environments. *ACS Catal.* **14**, 3482–3491 (2024).

[11] Takato, M. et al. Photoproximity labeling of endogenous receptors in the live mouse brain in minutes. *Nat. Chem. Biol.*10.1038/s41589-024-01692-4 (2024). 39090312 10.1038/s41589-024-01692-4

[12] Shu, X. et al. A genetically encoded tag for correlated light and electron microscopy of intact cells, tissues, and organisms. *PLoS Biol.* **9**, e1001041 (2011). 21483721 10.1371/journal.pbio.1001041 PMC3071375

[13] To, T.-L. et al. Photoactivatable protein labeling by singlet oxygen mediated reactions. *Bioorg. Med. Chem. Lett.* **26**, 3359–3363 (2016). 27220724 10.1016/j.bmcl.2016.05.034 PMC4903891

[14] Zhai, Y. et al. Spatiotemporal-resolved protein networks profiling with photoactivation dependent proximity labeling. *Nat. Commun.* **13**, 4906 (2022). 35987950 10.1038/s41467-022-32689-z PMC9392063

[15] Zheng, F., Yu, C., Zhou, X. & Zou, P. Genetically encoded photocatalytic protein labeling enables spatially-resolved profiling of intracellular proteome. *Nat. Commun.* **14**, 2978 (2023). 37221179 10.1038/s41467-023-38565-8 PMC10205723

[16] Hananya, N., Ye, X., Koren, S. & Muir, T. W. A genetically encoded photoproximity labeling approach for mapping protein territories. *Proc. Natl Acad. Sci. USA* **120**, e2219339120 (2023). 37036999 10.1073/pnas.2219339120 PMC10120045

[17] Geri, J. B. et al. Microenvironment mapping via Dexter energy transfer on immune cells. *Science* **367**, 1091–1097 (2020). 32139536 10.1126/science.aay4106 PMC7336666

[18] Oslund, R. C. et al. Detection of cell–cell interactions via photocatalytic cell tagging. *Nat. Chem. Biol.* **18**, 850–858 (2022). 35654846 10.1038/s41589-022-01044-0

[19] Bechtel, T. J. et al. Proteomic mapping of intercellular synaptic environments via flavin-dependent photoredox catalysis. *Org. Biomol. Chem.* **21**, 98–106 (2022). 36477737 10.1039/d2ob02103j

[20] Hope, T. O. et al. Targeted proximity-labelling of protein tyrosines via flavin-dependent photoredox catalysis with mechanistic evidence for a radical–radical recombination pathway. *Chem. Sci.* **14**, 7327–7333 (2023). 37416718 10.1039/d3sc00638g PMC10321502

[21] Tamura, T., Takato, M., Shiono, K. & Hamachi, I. Development of a photoactivatable proximity labeling method for the identification of nuclear proteins. *Chem. Lett.* **49**, 145–148 (2020).

[22] Tsushima, M. et al. Intracellular photocatalytic-proximity labeling for profiling protein–protein interactions in microenvironments. *Chem. Commun.* **58**, 1926–1929 (2022). 10.1039/d1cc05764b 35040832

[23] Wang, H. et al. Selective mitochondrial protein labeling enabled by biocompatible photocatalytic reactions inside live cells. *JACS Au* **1**, 1066–1075 (2021). 34467350 10.1021/jacsau.1c00172 PMC8395695

[24] Wang, H. et al. A photo-oxidation driven proximity labeling strategy enables profiling of mitochondrial proteome dynamics in living cells. *Chem. Sci.* **13**, 11943–11950 (2022). 36320915 10.1039/d2sc04087e PMC9580500

[25] Zhai, Y. et al. Global profiling of functional histidines in live cells using small-molecule photosensitizer and chemical probe relay labelling. *Nat. Chem.* **16**, 1546–1557 (2024). 38834725 10.1038/s41557-024-01545-6

[26] Folkes, L. K., Trujillo, M., Bartesaghi, S., Radi, R. & Wardman, P. Kinetics of reduction of tyrosine phenoxyl radicals by glutathione. *Arch. Biochem. Biophys.* **506**, 242–249 (2011). 21147061 10.1016/j.abb.2010.12.006

[27] Rizk, M. S., Shi, X. & Platz, M. S. Lifetimes and reactivities of some 1,2-didehydroazepines commonly used in photoaffinity labeling experiments in aqueous solutions. *Biochemistry* **45**, 543–551 (2006). 16401083 10.1021/bi0516632

[28] Gorman, A. A. & Rodgers, M. A. J. New trends in photobiology. Current perspectives of singlet oxygen detection in biological environments. *J. Photochem. Photobiol. B* **14**, 159–176 (1992). 1432388 10.1016/1011-1344(92)85095-c

[29] Skovsen, E., Snyder, J. W., Lambert, J. D. C. & Ogilby, P. R. Lifetime and diffusion of singlet oxygen in a cell. *J. Phys. Chem. B* **109**, 8570–8573 (2005). 16852012 10.1021/jp051163i

[30] Trowbridge, A. D. et al. Small molecule photocatalysis enables drug target identification via energy transfer. *Proc. Natl Acad. Sci. USA* **119**, e2208077119 (2022). 35969791 10.1073/pnas.2208077119 PMC9407219

[31] Pan, C., Knutson, S. D., Huth, S. W. & MacMillan, D. W. C. µMap proximity labeling in living cells reveals stress granule disassembly mechanisms. *Nat. Chem. Biol.*10.1038/s41589-024-01721-2 (2024). 39215100 10.1038/s41589-024-01721-2 PMC11868469

[32] Dolan, C. et al. Cell uptake and cytotoxicity of a novel cyclometalated iridium(III) complex and its octaarginine peptide conjugate. *J. Inorg. Biochem.* **119**, 65–74 (2013). 23201851 10.1016/j.jinorgbio.2012.11.001

[33] Huang, Z. et al. Bioorthogonal photocatalytic decaging-enabled mitochondrial proteomics. *J. Am. Chem. Soc.* **143**, 18714–18720 (2021). 34709827 10.1021/jacs.1c09171

[34] Liu, Z. et al. Bioorthogonal photocatalytic proximity labeling in primary living samples. *Nat. Commun* **15**, 2712 (2024). 38548729 10.1038/s41467-024-46985-3 PMC10978841

[35] Walsh, C. Naturally occurring 5-deazaflavin coenzymes: biological redox roles. *Acc. Chem. Res.* **19**, 216–221 (1986).

[36] Nikitas, N. F., Gkizis, P. L. & Kokotos, C. G. Thioxanthone: a powerful photocatalyst for organic reactions. *Org. Biomol. Chem.* **19**, 5237–5253 (2021). 34047729 10.1039/d1ob00221j

[37] Alonso, R. & Bach, T. A chiral thioxanthone as an organocatalyst for enantioselective [2 + 2] photocycloaddition reactions induced by visible light. *Angew. Chem. Int. Ed.* **53**, 4368–4371 (2014). 10.1002/anie.201310997 24648167

[38] Tröster, A., Alonso, R., Bauer, A. & Bach, T. Enantioselective intermolecular [2 + 2] photocycloaddition reactions of 2(1*H*)-quinolones induced by visible light irradiation. *J. Am. Chem. Soc.* **138**, 7808–7811 (2016). 27268908 10.1021/jacs.6b03221 PMC4929526

[39] Elliott, L. D., Kayal, S., George, M. W. & Booker-Milburn, K. Rational design of triplet sensitizers for the transfer of excited state photochemistry from UV to visible. *J. Am. Chem. Soc.* **142**, 14947–14956 (2020). 32786778 10.1021/jacs.0c05069

[40] Huth, S. W. et al. μMap photoproximity labeling enables small molecule binding site mapping. *J. Am. Chem. Soc.* **145**, 16289–16296 (2023). 37471577 10.1021/jacs.3c03325 PMC10809032

[41] Bliese, M., Launikonis, A., Loder, J. W., Mau, A. W. H. & Sasse, W. H. F. Photoreduction of deazaflavin. Spectroscopic investigations. *Aust. J. Chem.* **36**, 1873–1883 (1983).

[42] Lutkus, L. V., Rickenbach, S. S. & McCormick, T. M. Singlet oxygen quantum yields determined by oxygen consumption. *J. Photochem. Photobiol. A* **378**, 131–135 (2019).

[43] Graml, A. et al. Deazaflavin reductive photocatalysis involves excited semiquinone radicals. *Nat. Commun.* **11**, 3174 (2020). 32576821 10.1038/s41467-020-16909-y PMC7311442

[44] Mojr, V. et al. Tailoring flavins for visible light photocatalysis: organocatalytic [2+2] cycloadditions mediated by a flavin derivative and visible light. *Chem. Commun.* **51**, 12036–12039 (2015). 10.1039/c5cc01344e 26121238

[45] Mojr, V. et al. Flavin photocatalysts for visible-light [2+2] cycloadditions: structure, reactivity and reaction mechanism. *ChemCatChem* **10**, 849–858 (2018).

[46] Lin, Z. et al. Multiscale photocatalytic proximity labeling reveals cell surface neighbors on and between cells. *Science* **385**, eadl5763 (2024). 39024454 10.1126/science.adl5763 PMC12517702

[47] Arias-Rotondo, D. M. & McCusker, J. K. The photophysics of photoredox catalysis: a roadmap for catalyst design. *Chem. Soc. Rev.* **45**, 5803–5820 (2016). 27711624 10.1039/c6cs00526h

[48] West, A. V. et al. Labeling preferences of diazirines with protein biomolecules. *J. Am. Chem. Soc.* **143**, 6691–6700 (2021). 33876925 10.1021/jacs.1c02509 PMC11647638

[49] Mühldorf, B. & Wolf, R. Photocatalytic benzylic C–H bond oxidation with a flavin scandium complex. *Chem. Commun.* **51**, 8425–8428 (2015). 10.1039/c5cc00178a 25647055

[50] Gutierrez, C. & Schiff, R. HER2 biology, detection, and clinical implications. *Arch. Pathol. Lab. Med.* **135**, 55–62 (2011). 21204711 10.1043/2010-0454-RAR.1 PMC3242418

[51] Bartholow, T. G. et al. Photoproximity labeling from single catalyst sites allows calibration and increased resolution for carbene labeling of protein partners in vitro and on cells. *ACS Cent. Sci.* **10**, 199–208 (2024). 38292613 10.1021/acscentsci.3c01473 PMC10823516

[52] Roßmann, K., Birke, R., Levitz, J., Jones, B. & Broichhagen, J. Red and far-red cleavable fluorescent dyes for self-labelling enzyme protein tagging and interrogation of GPCR co-internalization. *RSC Chem. Biol.* **6**, 11–20 (2025). 39610654 10.1039/d4cb00209a PMC11599839

[53] Kuratomi, K. & Kobayashi, Y. Studies on the interactions between DNA and flavins. *Biochim. Biophys. Acta* **476**, 207–217 (1977). 328045 10.1016/0005-2787(77)90004-1

[54] Ikeda, H., Yoshida, K., Ozeki, M. & Saito, I. Synthesis and characterization of flavin-tethered peptide nucleic acid. *Tetrahedron Lett.* **42**, 2529–2531 (2001).

[55] Merkle, T., Sinn, M. & Hartig, J. S. Interactions between flavins and quadruplex nucleic acids. *ChemBioChem* **16**, 2437–2440 (2015). 26426822 10.1002/cbic.201500463

[56] Huang, H. et al. Targeted photoredox catalysis in cancer cells. *Nat. Chem.* **11**, 1041–1048 (2019). 31548671 10.1038/s41557-019-0328-4

[57] Toh, K. et al. Chemoproteomic identification of blue-light-damaged proteins. *J. Am. Chem. Soc.* **144**, 20171–20176 (2022). 36306265 10.1021/jacs.2c07180

[58] Peraro, L. et al. Cell penetration profiling using the chloroalkane penetration assay. *J. Am. Chem. Soc.* **140**, 11360–11369 (2018). 30118219 10.1021/jacs.8b06144 PMC6205923

[59] Roßmann, K. et al. *N*-Methyl deuterated rhodamines for protein labelling in sensitive fluorescence microscopy. *Chem. Sci.* **13**, 8605–8617 (2022). 35974762 10.1039/d1sc06466e PMC9337740

[60] Knutson, S. D. et al. Parallel proteomic and transcriptomic microenvironment mapping (μMap) of nuclear condensates in living cells. *J. Am. Chem. Soc.* **147**, 488–497 (2024). 39707993 10.1021/jacs.4c11612 PMC11792175

[61] Oba, M. & Demizu, Y. (eds) *Cell-Penetrating Peptides: Design, Development and Applications* (Wiley, 2022); 10.1002/9783527835997

[62] Herce, H. D. et al. Cell-permeable nanobodies for targeted immunolabelling and antigen manipulation in living cells. *Nat. Chem.* **9**, 762–771 (2017). 28754949 10.1038/nchem.2811

[63] Schneider, A. F. L., Wallabregue, A. L. D., Franz, L. & Hackenberger, C. P. R. Targeted subcellular protein delivery using cleavable cyclic cell-penetrating peptides. *Bioconjug. Chem.* **30**, 400–404 (2019). 30616339 10.1021/acs.bioconjchem.8b00855

[64] Arafiles, J. V. V. et al. Cell-surface-retained peptide additives for the cytosolic delivery of functional proteins. *J. Am. Chem. Soc.* **145**, 24535–24548 (2023). 37906525 10.1021/jacs.3c05365 PMC10655119

[65] Kawaguchi, Y. et al. Identification of cellular proteins interacting with octaarginine (R8) cell-penetrating peptide by photo-crosslinking. *Bioorg. Med. Chem. Lett.* **23**, 3738–3740 (2013). 23726025 10.1016/j.bmcl.2013.05.008

[66] Kawaguchi, Y. et al. Syndecan-4 is a receptor for clathrin-mediated endocytosis of arginine-rich cell-penetrating peptides. *Bioconjug. Chem.* **27**, 1119–1130 (2016). 27019270 10.1021/acs.bioconjchem.6b00082

[67] Morgan, D. C., Knutson, S. D., Pan, C. (R.) & MacMillan, D. W. C. Temporal microenvironment mapping (μMap) of intracellular trafficking pathways of cell-penetrating peptides across the blood–brain barrier. Preprint at *bioRxiv*10.1101/2025.01.15.633151 (2025).

[68] Martin, R. M. et al. Principles of protein targeting to the nucleolus. *Nucleus* **6**, 314–325 (2015). 26280391 10.1080/19491034.2015.1079680 PMC4615656

[69] Jørgensen, P. L. Mechanism of the Na^+^, K^+^ pump protein structure and conformations of the pure (Na^+^ + K^+^)-ATPase. *Biochim. Biophys. Acta* **694**, 27–68 (1982). 6289898 10.1016/0304-4157(82)90013-2

[70] Kühlbrandt, W. Biology, structure and mechanism of P-type ATPases. *Nat. Rev. Mol. Cell Biol.* **5**, 282–295 (2004). 15071553 10.1038/nrm1354

[71] Trofimenko, E. et al. Genetic, cellular, and structural characterization of the membrane potential-dependent cell-penetrating peptide translocation pore. *eLife* **10**, e69832 (2021). 34713805 10.7554/eLife.69832 PMC8639150

[72] Gentilucci, L., Marco, R., De & Cerisoli, L. Chemical modifications designed to improve peptide stability: incorporation of non-natural amino acids, pseudo-peptide bonds, and cyclization. *Curr. Pharm. Des.* **16**, 3185–3203 (2010). 20687878 10.2174/138161210793292555

[73] Qin, W. et al. Dynamic mapping of proteome trafficking within and between living cells by TransitID. *Cell* **186**, 3307–3324.e30 (2023). 37385249 10.1016/j.cell.2023.05.044 PMC10527209

[74] Lee, S.-Y. et al. Engineered allostery in light-regulated LOV-Turbo enables precise spatiotemporal control of proximity labeling in living cells. *Nat. Methods* **20**, 908–917 (2023). 37188954 10.1038/s41592-023-01880-5 PMC10539039

[75] Trojani, M.-C., Santucci-Darmanin, S., Breuil, V., Carle, G. F. & Pierrefite-Carle, V. Lysosomal exocytosis: from cell protection to protumoral functions. *Cancer Lett.* **597**, 217024 (2024). 38871244 10.1016/j.canlet.2024.217024

[76] Ji, X., Nielsen, A. L. & Heinis, C. Cyclic peptides for drug development. *Angew. Chem. Int. Ed.* **63**, e202308251 (2024). 10.1002/anie.202308251 37870189

[77] Hickey, J. L., Sindhikara, D., Zultanski, S. L. & Schultz, D. M. Beyond 20 in the 21st century: prospects and challenges of non-canonical amino acids in peptide drug discovery. *ACS Med. Chem. Lett.* **14**, 557–565 (2023). 37197469 10.1021/acsmedchemlett.3c00037 PMC10184154

[78] Hsiao, Y. et al. Analysis and visualization of quantitative proteomics data using FragPipe-Analyst. *J. Proteome Res.* **23**, 4303–4315 (2024). 39254081 10.1021/acs.jproteome.4c00294 PMC13142904

[79] Hughes, C. S. et al. Single-pot, solid-phase-enhanced sample preparation for proteomics experiments. *Nat. Protoc.* **14**, 68–85 (2019). 30464214 10.1038/s41596-018-0082-x

[80] The, M., MacCoss, M. J., Noble, W. S. & Käll, L. Fast and accurate protein false discovery rates on large-scale proteomics data sets with Percolator 3.0. *J. Am. Soc. Mass Spectrom.* **27**, 1719–1727 (2016). 27572102 10.1007/s13361-016-1460-7 PMC5059416

[81] Keller, A., Nesvizhskii, A. I., Kolker, E. & Aebersold, R. Empirical statistical model to estimate the accuracy of peptide identifications made by MS/MS and database search. *Anal. Chem.* **74**, 5383–5392 (2002). 12403597 10.1021/ac025747h

[82] Yu, F., Haynes, S. E. & Nesvizhskii, A. I. IonQuant enables accurate and sensitive label-free quantification with FDR-controlled match-between-runs. *Mol. Cell. Proteomics* **20**, 100077 (2021). 33813065 10.1016/j.mcpro.2021.100077 PMC8131922

[83] Becker, T. et al. Transforming chemical proteomics enrichment into a high-throughput method using an SP2E workflow. *JACS Au* **2**, 1712–1723 (2022). 35911458 10.1021/jacsau.2c00284 PMC9326820

[84] Kong, A. T., Leprevost, F. V., Avtonomov, D. M., Mellacheruvu, D. & Nesvizhskii, A. I. MSFragger: ultrafast and comprehensive peptide identification in mass spectrometry-based proteomics. *Nat. Methods* **14**, 513–520 (2017). 28394336 10.1038/nmeth.4256 PMC5409104

[85] Ge, S. X., Jung, D., Jung, D. & Yao, R. ShinyGO: a graphical gene-set enrichment tool for animals and plants. *Bioinformatics* **36**, 2628–2629 (2020). 31882993 10.1093/bioinformatics/btz931 PMC7178415

