# Supplementary material for: Energy-transfer photoproximity labelling in live cells using an organic cofactor
Source: Nat Chem. 2025 Sep 17;17(12):1928–40. doi: 10.1038/s41557-025-01931-8 (PMC12669049; doi:10.1038/s41557-025-01931-8)

nature chemistry

Supplementary information

https://doi.org/10.1038/s41557-025-01931-8

Energy-transfer photoproximity labelling in live cells using an organic cofactor

In the format provided by the authors and unedited

Table of Contents

Table of Contents ... 1
1 Supplementary Figures and Discussion ... 3
Supplementary Figure 1: UV-Vis absorption and emission spectra ... 3
Supplementary Figure 2: NMR spectra of diazirine 1 conversion ... 5
Supplementary Figure 3: Photochemical stability of deazaflavin 4 ... 8
Supplementary Figure 4: Additional photocatalyst screening ... 9
Supplementary Figure 5: Control experiments ... 10
Supplementary Figure 6: Photophysical characterisation of deazaflavin 4 ... 11
Supplementary Figure 7: Photochemistry of deazaflavin 4 in the presence of glutathione ... 13
Supplementary Figure 8: LC-MS/MS of HSA labelling ... 14
Supplementary Figure 9: HaloTag self-labelling and HaloTag-GluR2 expression ... 15
Supplementary Figure 10: Comparison of deazaflavin and iridium extracellular labelling GO terms 16
Supplementary Figure 11: Transient absorption spectroscopy of triplet state quenching by molecular oxygen ... 17
Supplementary Figure 12: Cell microscopy image of deazaflavin 16 ... 19
Supplementary Figure 13: Optimisation of intracellular DarT-labelling irradiation time using deazaflavin-R_{10} conjugates ... 20
Supplementary Figure 14: Western blot of fractionated cells lysates after intracellular DarT-labelling with deazaflavin-R_{10} conjugates ... 21
Supplementary Figure 15: Comparison of linear and cyclic R_{10} GO terms ... 22
Supplementary Figure 16: Scatter plot of protein abundance ranked by intensity ... 23
Supplementary Figure 17: Intracellular fate of R_{10} peptides ... 24
Supplementary Figure 18: Comparison of GO terms over time ... 25
2 General Information ... 26
2.1 Light source and irradiation setups ... 26
2.2 Materials ... 27
2.3 Characterisation techniques ... 27
2.4 Preparative HPLC ... 27
2.5 Analytical HPLC ... 27
2.6 Plasmid information ... 28
2.7 General cell culture ... 28
2.8 Transfection ... 29
2.9 LC-MS/MS ... 29
3 Experimental Procedures ... 30
3.1 Organic Synthesis ... 30
3.1.1 Photocatalysts ... 30

3.1.2 Biotin and photoaffinity probes 55
3.2 Photocatalyst Screening 59
3.2.1 Phenyl azide conversion *via* HPLC 59
3.3 Mechanistic Studies 60
3.3.1 Stationary Absorption and Emission Spectroscopy 60
3.3.2 Time-resolved Emission Spectroscopy 60
3.3.3 Sub-picosecond Pump/Supercontinuum-Probe Spectroscopy 60
3.3.4 Nanosecond to millisecond transient absorption spectroscopy 61
3.3.5 Analysis of transient absorption data and modelling 61
3.3.6 Discussion on bimolecular reactions with the excited singlet state of 4 controlled by diffusion according to Smoluchowski theory 61
3.4 Antibody conjugation 63
3.4.1 Cysteine modification of Trastuzumab with deazaflavin 4 63
3.4.2 Lysine modification of polyclonal anti-mouse IgG with deazaflavin 4 64
3.5 HaloTag-GluR2 expression and self-labelling validation by microscopy 65
3.6 Peptide Synthesis 66
3.7 Intracellular DarT-labelling for western blot analysis 69
References 70
4 Source data for Supplementary Figure 13 73
5 Source data for Supplementary Figure 14 73

1 Supplementary Figures and Discussion

Supplementary Figure 1: UV-Vis absorption and emission spectra

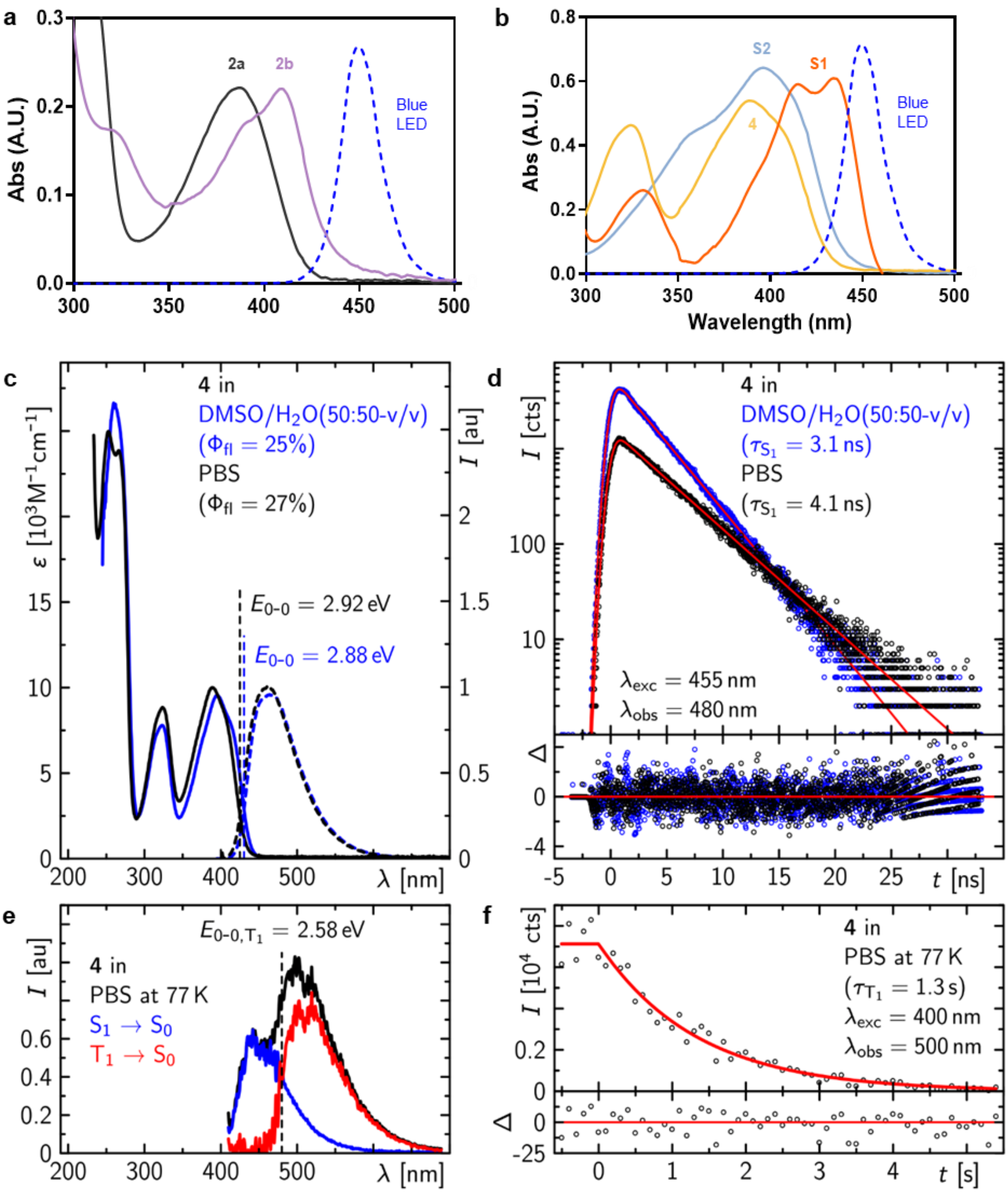

Steady-state absorption and emission spectra in the UV/Vis spectral range of (**a**) thioxanthones **2a** and **2b** (50 µM) in PBS (2% DMSO) with emission spectrum of blue LED used; (**b**) deazaflavins **4**, **S1** and alloxazine **S2** (50 µM) PBS (2% DMSO) with emission spectrum of blue LED used; (**c**,**e**) of **4** and the corresponding emission decay (**d**,**f**) in either DMSO/H_2O (50:50-v/v) (blue) or PBS buffer (black). The excited singlet emission quantum yields (Φ_{fl}), the excited singlet state energy above the ground state (E_{0-0}), the triplet state energy above the ground state at 77 K ($E_{0\text{-}0,T1}$), the excited singlet state lifetimes (τ_{S1}) , and the triplet state lifetimes at 77 K (τ_{T1}) are given in the corresponding panels. The red lines in **d** and **f** are mono-exponential fits to the data. The corresponding residuals (weighted by the square-root of the counted events at each time-point accounting for Poissonian statistics) are given in the lower parts of **d** and **f**.

Discussion: Deazaflavin **4** in solution shows three electronic transitions in the UV/Vis spectral range with the $S_1 \leftarrow S_0$ absorption band peaking at around 400 nm (**Fig. S1a**) similar to other known deazaflavins.[1] The emission roughly images the first absorption band indicating a rather stiff molecular scaffold as expected from its Lewis structure. In a buffered water environment, the emission quantum yield is *ca.* 27% and the excited singlet state decays with a lifetime of 4.1 ns. With increasing amount of DMSO in the water environment the absorption and emission redshifts. At DMSO/H_2O (50:50-v/v) the emission quantum yield slightly drops to 25% and the excited singlet lifetime reduces to 3.1 ns. Accordingly, the radiative rate constant of **4** slightly increases from $6.59 \cdot 10^7$ s^{-1} to $8.06 \cdot 10^7$ s^{-1} going from PBS buffer to DMSO/H_2O (50:50-v/v). The emission quantum yields already provide an upper limit for the potential intersystem crossing yield (Φ_{isc}) of 73% to 75% depending on the solvent environment. At 77 K the phosphorescence of **4** is observed redshifted to the emission band by *ca.* 0.3 eV giving an estimated triplet energy of 2.58 eV (59.5 kcal/mol) that corresponds to previous reported values.[2]

Supplementary Figure 2: NMR spectra of diazirine 1 conversion

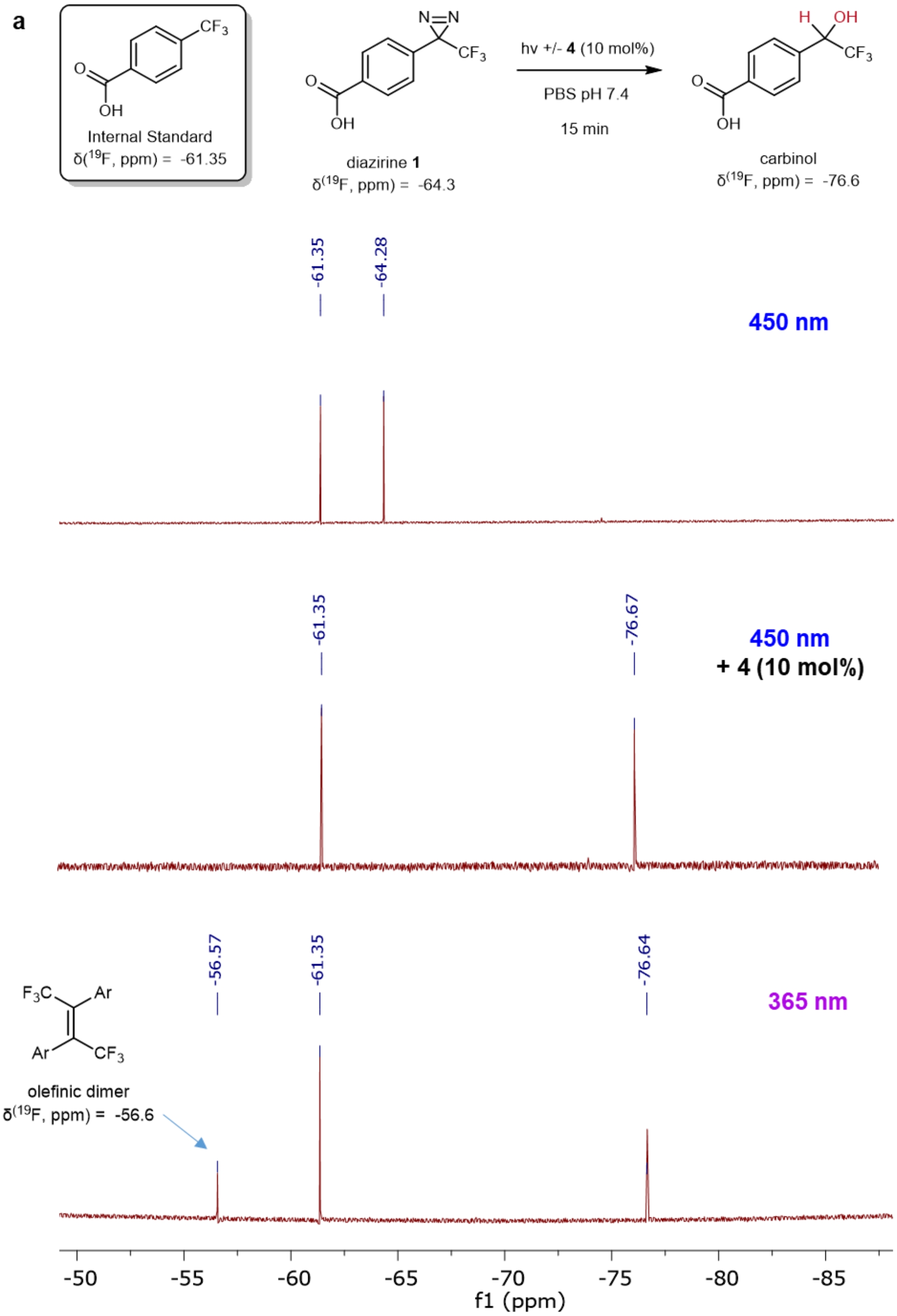

b

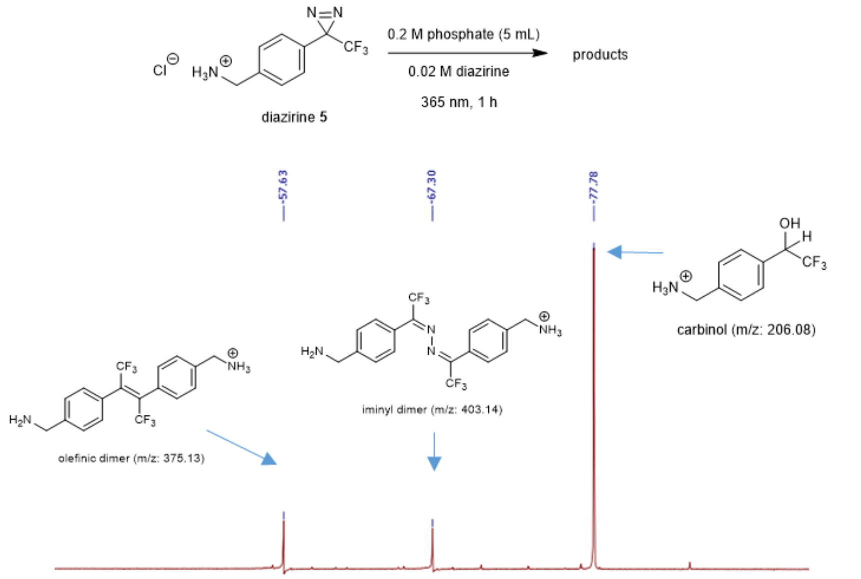

0.2 M phosphate (5 mL)
0.02 M diazirine
365 nm, 1 h
products
diazirine 5
-57.63
-67.30
-77.78
carbinol (m/z: 206.08)
iminyl dimer (m/z: 403.14)
olefinic dimer (m/z: 375.13)

c

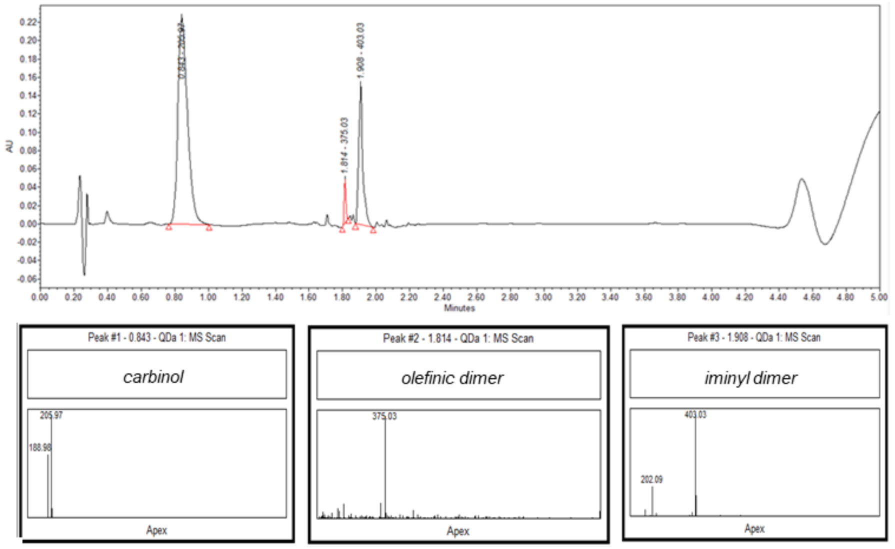

AU
Minutes
0.843 - 205.97
1.814 - 375.03
1.908 - 403.03
Peak #1 - 0.843 - QDa 1: MS Scan
carbinol
205.97
188.98
Apex
Peak #2 - 1.814 - QDa 1: MS Scan
olefinic dimer
375.03
Apex
Peak #3 - 1.908 - QDa 1: MS Scan
iminyl dimer
403.03
202.09
Apex

d

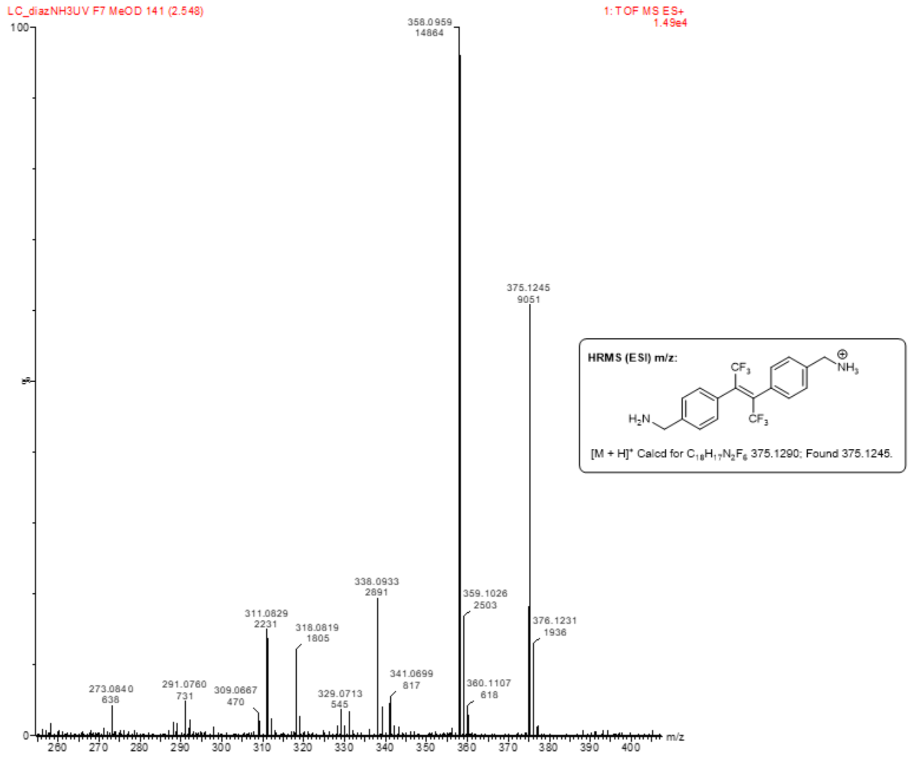

LC_diazNH3UV F7 MeOD 141 (2.548)
1: TOF MS ES+
1.49e4
358.0959
14864
375.1245
9051
338.0933
2891
359.1026
2503
311.0829
2231
376.1231
1936
318.0819
1805
341.0699
817
291.0760
731
273.0840
638
360.1107
618
329.0713
545
309.0667
470
m/z
HRMS (ESI) m/z:
[M + H]+ Calcd for C18H17N2F6 375.1290; Found 375.1245.

Exemplary ^{19}F NMR spectra comparing diazirine **1** conversion to carbinol product using either **4** (10 mol%) and 450 nm irradiation or without photocatalyst and 365 nm irradiation that also results in olefinic dimer formation (bottom panel, **Fig. S2a**). Evidence of this dimer formation was gathered by performing a larger scale reaction using diazirine **5** in phosphate buffer that showed an analogous ^{19}F NMR chemical shift (-57.6 ppm in 9:1 H_2O/D_2O, **b**) after photolysis with irradiation at 365 nm, as well as expected chemical shifts for carbinol and imimyl dimer (-77.8 ppm and -67.3 ppm)[3] that were also observed by LC-MS (**c**). HPLC separation of the reaction mixture and HRMS analysis (**d**) of fractions points towards olefinic dimer formation with ^{19}F chemical shifts aligning to equivalent olefinic dimers.[4,5]

Supplementary Figure 3: Photochemical stability of deazaflavin 4

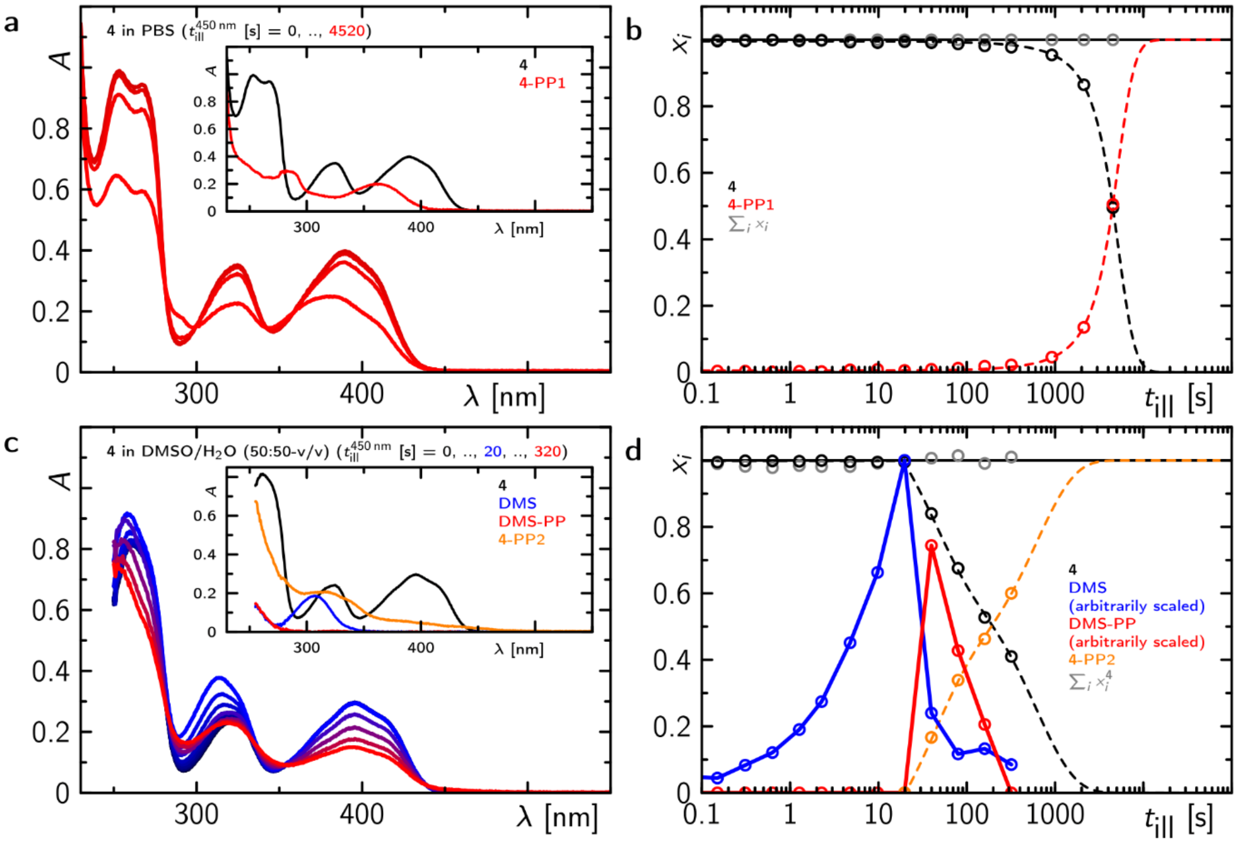

Stepwise illumination of **4** in PBS buffer (**a**-**b**) or DMSO/H_2O (50:50-v/v) (**c**-**d**) at 450 nm as indicated. **a**,**c**: Sequence of absorption spectra in the UV/Vis spectral range. Inset shows the species associated spectra contributing to the data. **b**,**d**: Corresponding mole fraction over time profiles. The dashed lines in **b** and **d** represent either autocatalytic functions of the form $A/(1+\exp(k(x-x_0)))$ (**b**) or exponential decays from a constant offset (**d**) to guide the eye. PP = photoproduct.

Discussion: Illumination of **4** in either PBS buffer or DMSO/H_2O (50:50-v/v) under identical illumination conditions revealed different photostability and different photoproducts (**Fig. S2**). In the case of PBS as solvent, **4** starts to decompose after an accumulative illumination time of *ca.* 100 s with an accelerating kinetics.[6,7] We speculate that with prolonged illumination intervals, the amount of molecular singlet oxygen ($^1O_2$) exceeds a critical concentration that causes **4** radical and peroxy species formation and, thus, accelerated decomposition. In contrast in the case of DMSO/H_2O (50:50-v/v) as solvent, **4** initially photo-catalytically converts DMSO into dimethyl sulfone (DMS, **Fig. S2c**).[8,9] However, after a prolonged illumination time **4** also starts to decompose accompanied by a simultaneous decrease of DMS (**Fig. S2d**). Here, the involvement of formed DMS together with the excess amount of molecular singlet oxygen $^1O_2$ causes the formation of a different **4** photodecomposition product (**4**-PP2, **Fig. S2c** and **S2d**).

Supplementary Figure 4: Additional photocatalyst screening

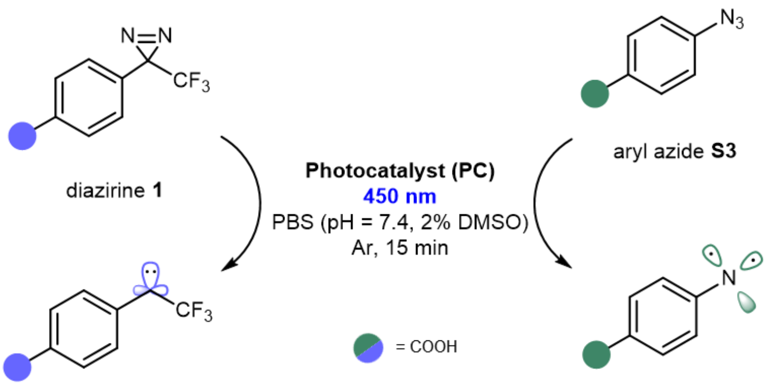

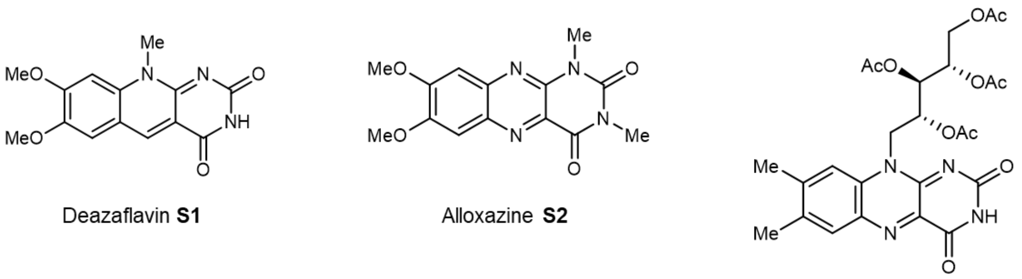

Entry	Photocatalyst (PC)	E_T (kcal/mol)	λ_{max} (nm)	Conversion (%) *	Conversion (%) †
1	thioxanthone (**2a**)	67.4	387	71	77
2	MeO-thioxanthone (**2b**)	-	409	<5	61
3	Ir[dFCF$_3$CO$_2$Hppy]$_2$[diolbpy] (**3a**)	60.1	380	100	84
4	[Ir(dF(CF$_3$)ppy)$_2$(dtbbpy)]PF$_6$ (**3b**)	60.1	380[a]	75 (100)[a]	55 (79)[b]
5	deazaflavin (**4**)	59.6	389	100	100
6	diMeO-deazaflavin (**S1**)	-	418	44	79
7	diMeO-alloxazine (**S2**)	-	396	12	94
8	Riboflavin tetraacetate (**RTA**)	49.8	450	0	69
9	None	-	-	0	13

[a] 1:1 DMSO/H_2O, [b] 10% DMSO

* using ^{19}F NMR and *p*-(trifluoromethyl)benzoic acid as internal standard

† using HPLC calibration curves and *p*-(trifluoromethyl)benzoic acid as internal standard

Supplementary Figure 5: Control experiments

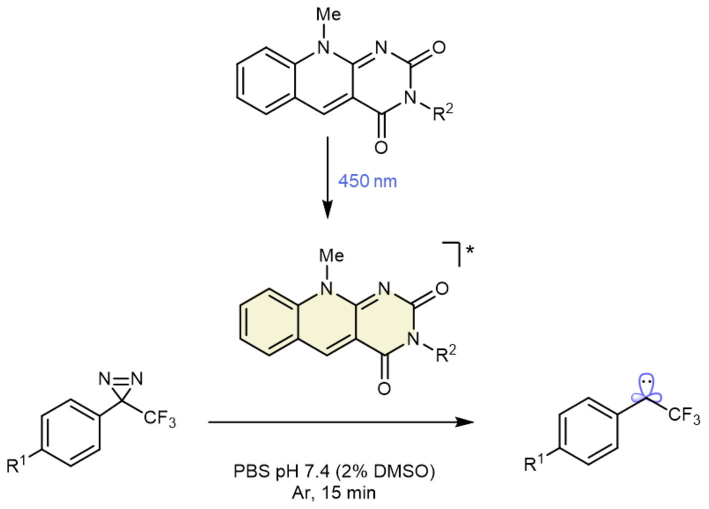

Entry	Condition	Conversion (%) †
1	standard (R^1 = COOH, R^2 = H)	100
2	no light	0
3	5 min irradiation	100
4	R^2 = CH_2COOH (**12**)	100
5	R^1 = CH_2NH_3Cl (**5**)	82
6	R^1 = CH_2-NHC(O)-PEG_4-Biotin (**8**)	100
7	10 mM GSH	55

† using ^{19}F NMR and *p*-(trifluoromethyl)benzoic acid as internal standard

Supplementary Figure 6: Photophysical characterisation of deazaflavin 4

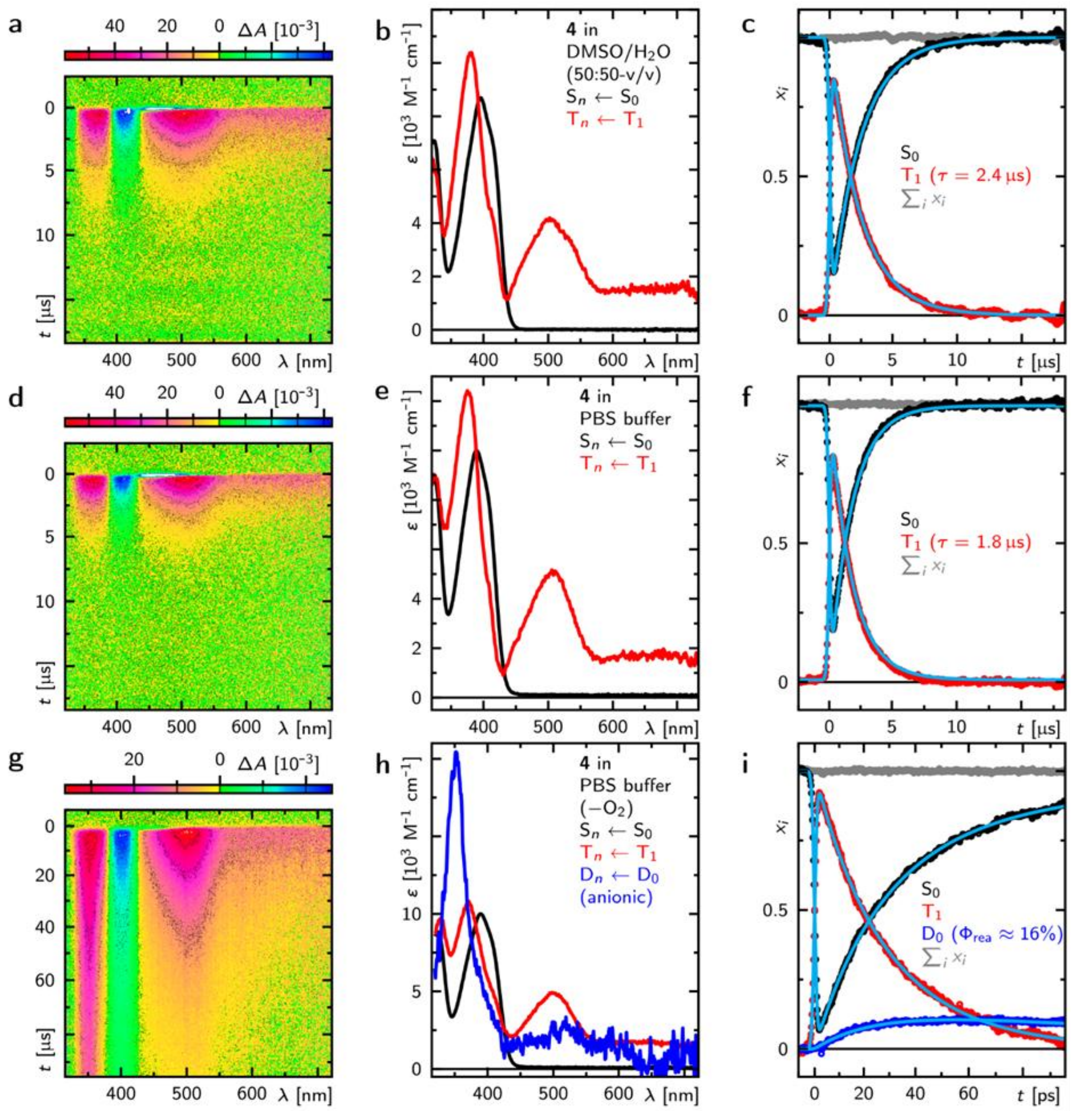

Transient absorption spectra in the UV/Vis spectral range of **4** in DMSO/H_2O (50:50-v/v) (**a**-**c**), PBS buffer (**d**-**f**), degassed PBS buffer (**g**-**i**) following excitation at 410 nm. The TA spectra are decomposed into its species associated spectra (**b**,**e**,**h**) and corresponding concentration time profiles (**c**,**f**,**i**) showing that the triplet in non-degassed environments is mainly quenched by a bimolecular diffusion controlled reaction with dissolved molecular oxygen.

Discussion: Following the absorption changes of **4** in DMSO/H_2O (50:50-v/v) on a fs to 6.5 ns time frame after excitation at 420 nm (**Fig. S3**) shows immediately within the excitation the formation of the first excited singlet state, which accompanied by minor internal vibrational energy redistribution and vibrational cooling converts partially to the **4** triplet and partially back to the ground state **4**. The triplet yield in DMSO/H_2O (50:50-v/v) is *ca.* 65%. On longer time scales the triplet state decays back into its ground state under non-degassed conditions with a lifetime of 2.4 µs or 1.8 µs in DMSO/H_2O (50:50-v/v) or in PBS buffer, respectively (**Fig. S3a-f**). In the absence of molecular oxygen, the triplet lifetime becomes significantly longer demonstrating that the lifetime under non-degassed conditions is mainly determined by a diffusion-controlled reaction with molecular oxygen forming mainly singlet oxygen after energy transfer on encounter. In degassed solution the triplet lifetime becomes so long that the triplet

even undergoes either a triplet-triplet disproportionation or a triplet-ground state reaction (**Fig. S3i**). Since the kinetics are described adequately by an exponential ansatz the latter process is most likely due to its expected pseudo-first order conditions. Under the used conditions the yield for the resulting product is *ca.* 16% and from this the intrinsic back intersystem crossing rate of the **4** triplet amounts to $2.97 \cdot 10^4$ s^{-1} (τ_{bisc} = 33.7 µs). Correspondingly, the efficiency for the bimolecular diffusion-controlled reaction between **4** in its triplet state and molecular oxygen in PBS can be calculated to:

$(1.8\ \mu s)^{-1}/((1.8\ \mu s)^{-1} + (33.7\ \mu s)^{-1}) = 95\%$.

Accordingly, total quantum yield for singlet oxygen formation is ϕ_Δ = 0.65 x 0.95 = 62%.

Supplementary Figure 7: Photochemistry of deazaflavin 4 in the presence of glutathione

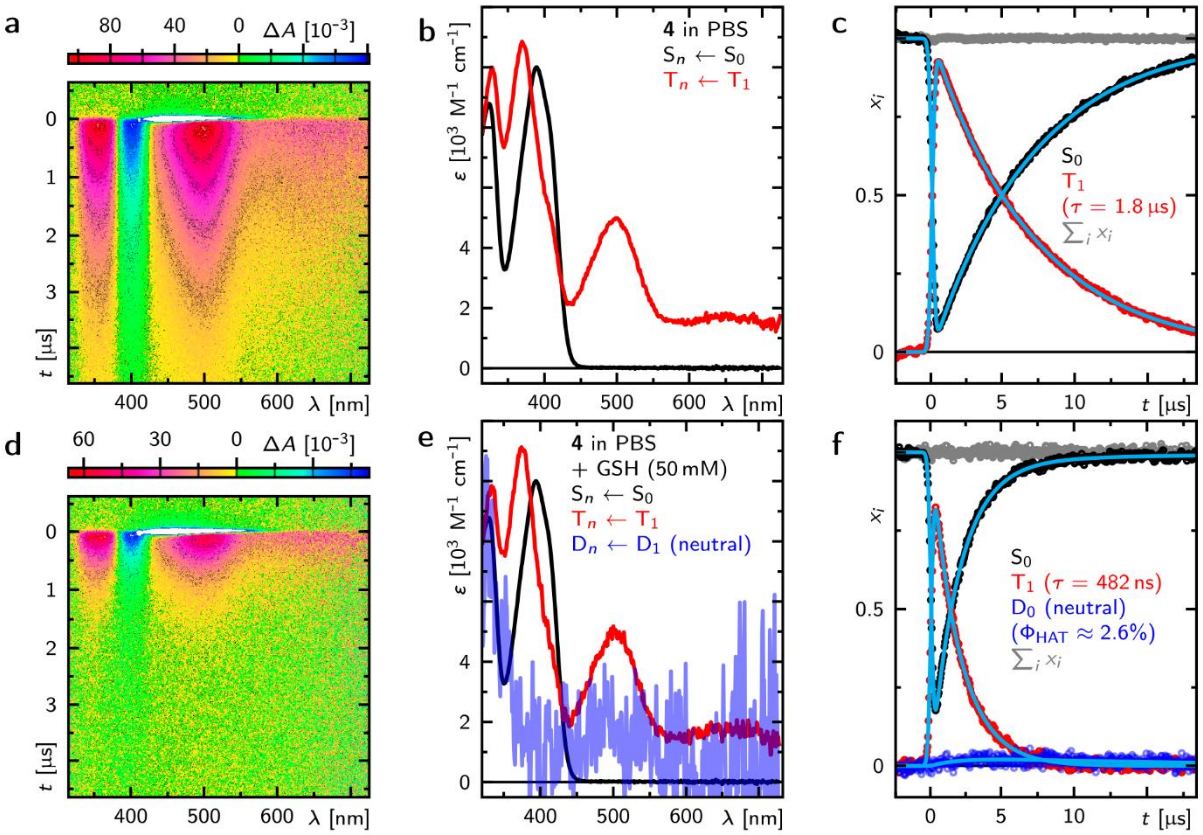

Transient absorption spectra in the UV/Vis spectral range of **4** in PBS buffer in the absence (**a**-**c**) or presence of glutathione (50 mM GSH, **d**-**f**) following excitation at 410 nm. The transient absorption spectra are decomposed into its species associated spectra (**b**,**e**) and corresponding concentration time profiles (**c**,**f**). The cyan lines in c and f represent the global fit to the data.

Discussion: At a GSH concentration of 50 mM the **4** triplet is quenched down to 482 ns with almost no observable product formation of either a **4** intermediate or GSH intermediate. To only about 2.6% a species spectrum is detectable that resembles most likely the neutral radical of **4** after hydrogen atom transfer or a proton coupled electron transfer. Since the stark triplet quenching indicates a quantum yield of ϕ_{eT} = 1 – (482 ns)/(1.8 µs) = 73%, the actual low product formation shows that radical pair recombination is very efficient in this case, which is potentially due to the rather high spin orbit coupling introduced by the sulphur within the GSH moiety.

Supplementary Figure 8: LC-MS/MS of HSA labelling

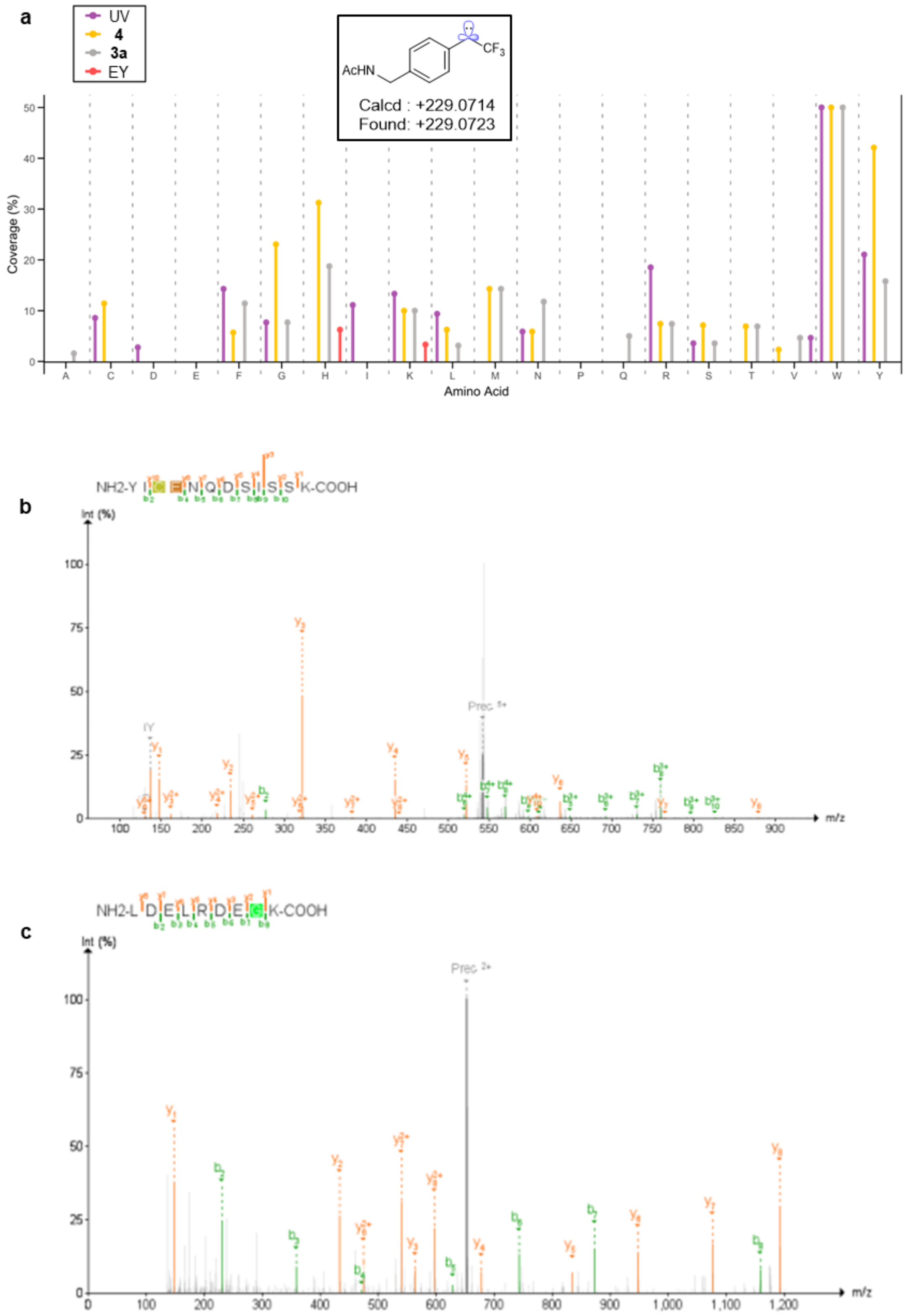

(**a**) LC-MS/MS analysis of recombinant HSA residue labelling by diazirine **S4** with either UV light (365 nm, 10 min) or blue light (450 nm, 10 min) with deazaflavin **4**, iridium **3a** or EY (10 µM). Representative modified peptide MS/MS data using deazaflavin **4** and (**b**) diazirine **8** or (**c**) diazirine **S8**.

Supplementary Figure 9: HaloTag self-labelling and HaloTag-GluR2 expression

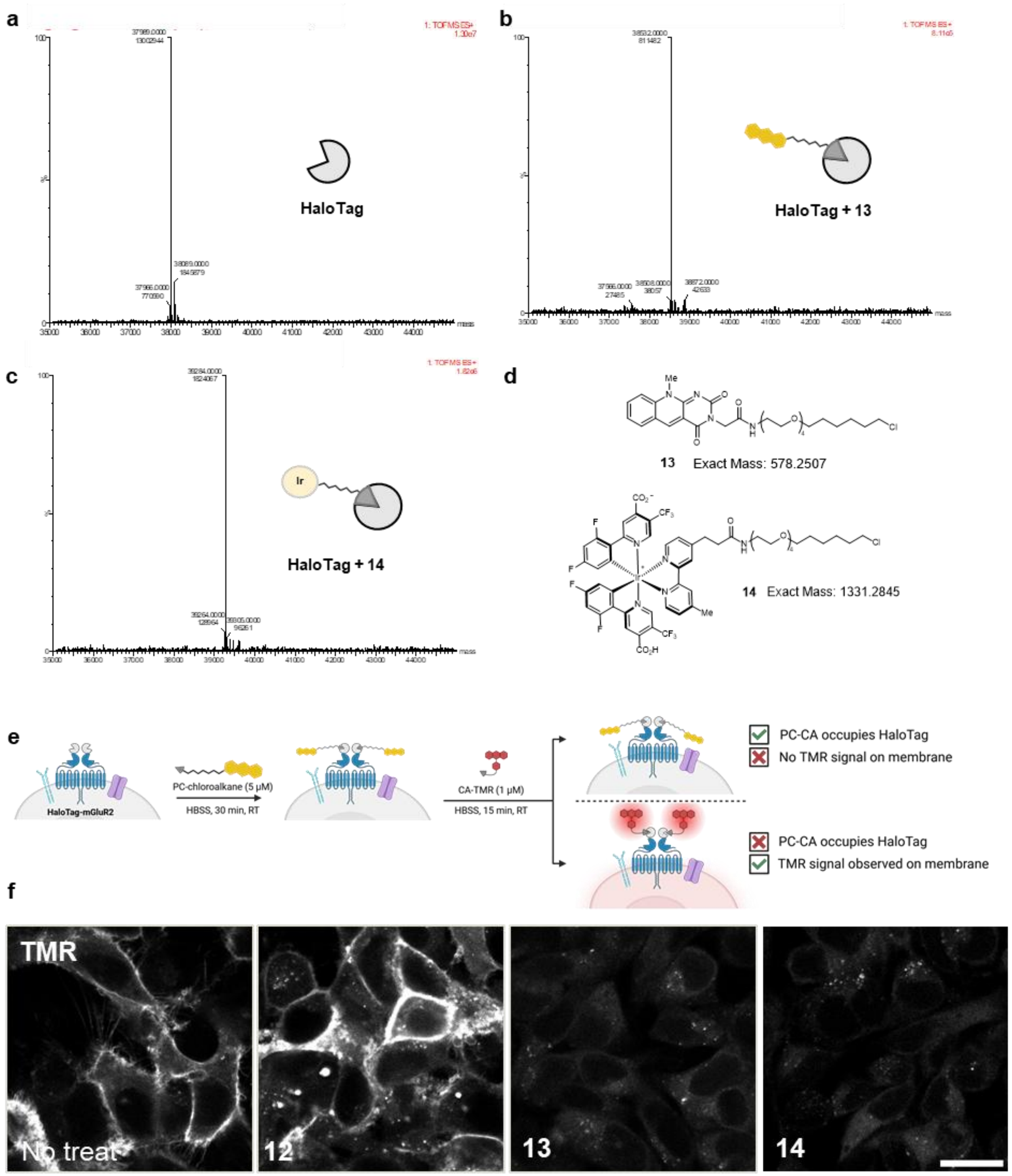

Deconvoluted HR-MS spectra of (**a**) HaloTag protein alone, (**b**) HaloTag + **13** and (**c**) HaloTag + **14** after self-labelling experiments, (**d**) Structures and masses of chloroalkane catalysts used. (**e**) Scheme of microscopic observation of extracellular HaloTag occupancy. (**f**) HeLa-HaloTag-mGluR2 cells treated with **12**, **13**, or **14** (5 μM) for 20 min at room temperature, followed by treatment with chloroalkane-tetramethylrhodamine (CA-TMR, 1 μM) for 15 min. Images captured by confocal laser scanning microscopy. Scale bar = 20 μm.

Supplementary Figure 10: Comparison of deazaflavin and iridium extracellular labelling GO terms

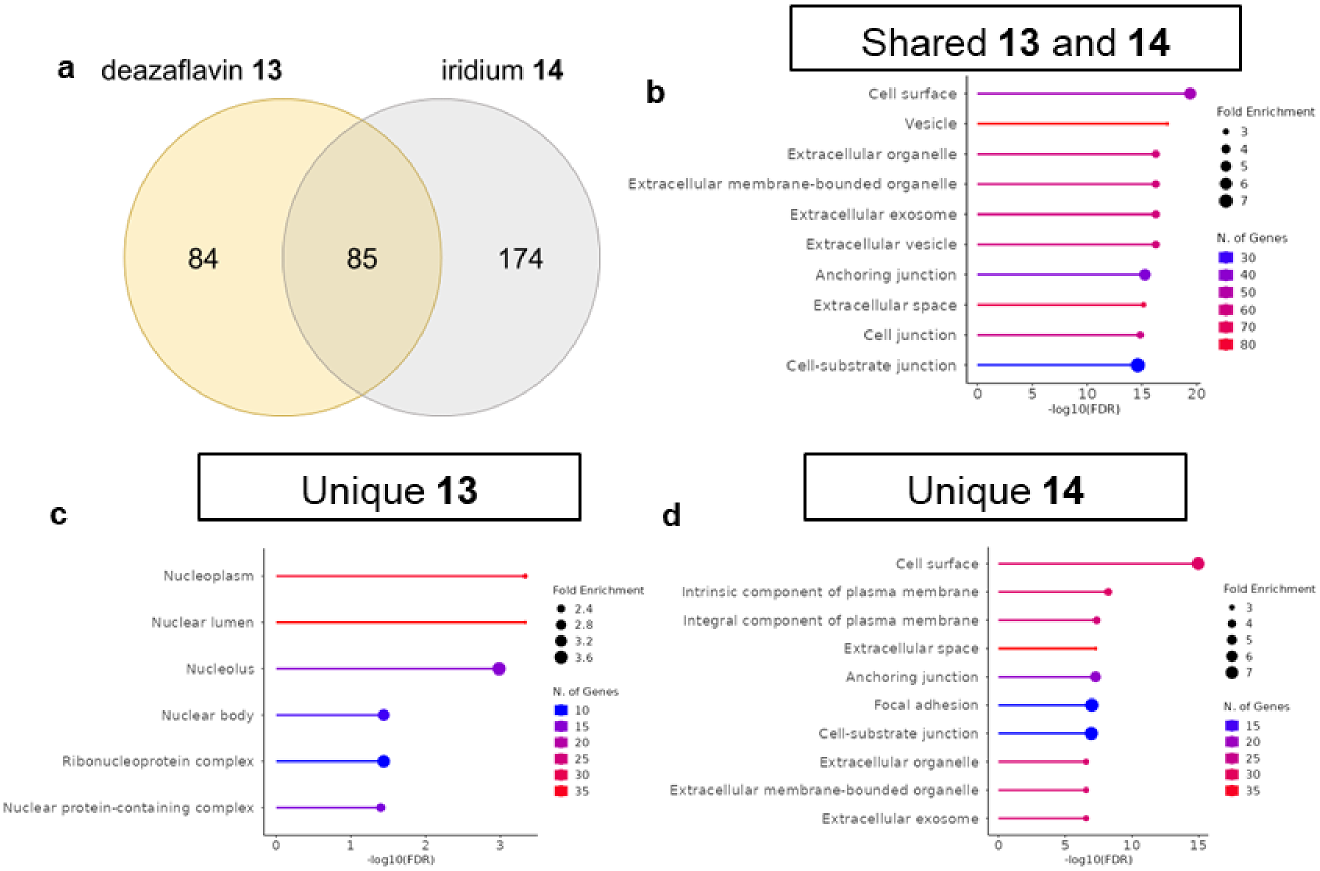

(**a**) Venn diagram comparing significantly enriched proteins from HeLa-HaloTag-mGLuR2 treated with deazaflavin **13** and iridium **14** showing the numbers of unique and shared protein hits. (**b**) GO terms (cellular compartment) for significantly enriched proteins shared between **13** and **14**. (**c**) GO terms (cellular compartment) for significantly enriched proteins unique to **13**. (**c**) GO terms (cellular compartment) for significantly enriched proteins unique to **14**.

Supplementary Figure 11: Transient absorption spectroscopy of triplet state quenching by molecular oxygen

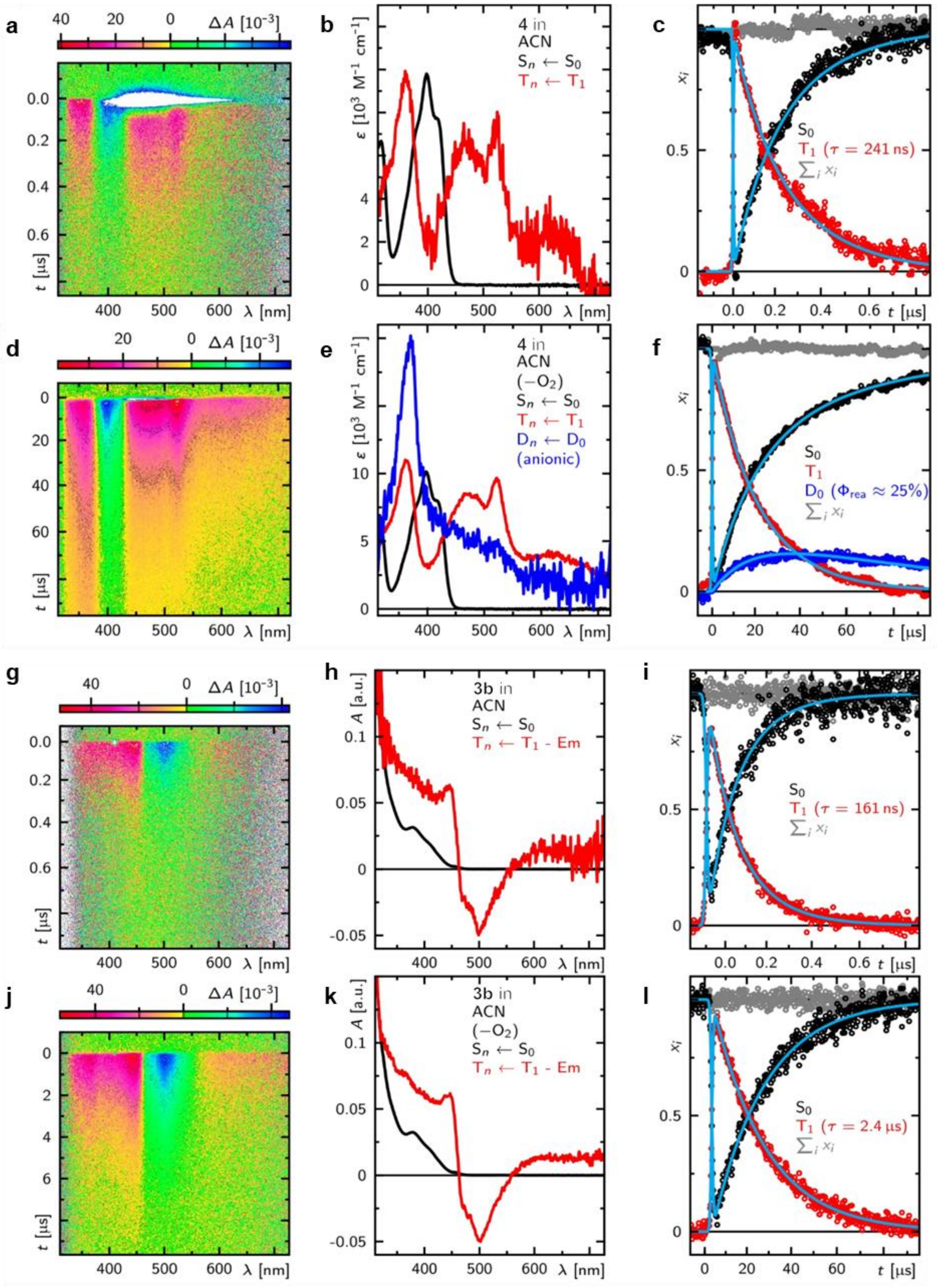

Transient absorption spectra in the UV/Vis spectral range of **4** in ACN (**a**-**c**) and degassed ACN (**d**-**f**), as well as iridium **3b** in ACN (**g**-**i**) and degassed ACN (**j**-**l**) following excitation at 410 nm. The transient absorption spectra are decomposed into their species associated spectra (**b**,**e**,**h**,**k**) and corresponding concentration time profiles (**c**,**f**,**i**,**l**) showing that the triplet in non-degassed environments is mainly quenched by a bimolecular diffusion controlled reaction with dissolved molecular oxygen.

Discussion: In the case of deazaflavin **4** in degassed solution, the triplet lifetime becomes so long that the triplet even undergoes either a triplet-triplet disproportionation or a triplet-ground state reaction (**Fig. S11d**-**f**). Since the kinetics are described adequately by an exponential ansatz the latter process is most likely due to its expected pseudo-first order conditions. Under the used conditions the yield for the resulting product is *ca.* 25% in ACN and from this the intrinsic back intersystem crossing rates of the **4** triplet amounts to $3.59 \cdot 10^4$ s^{-1} (τ_{bisc} = 27.9 µs) in ACN. Correspondingly, the efficiency for the bimolecular diffusion-controlled reaction between **4** in its triplet state and molecular oxygen can also be calculated to $1 - (27.9\ \mu s)^{-1}/(241\ ns)^{-1}$ = 99%. The total quantum yield for singlet oxygen formation (ϕ_Δ) is therefore 0.65 x 0.99 = 64%.

For the Ir complex **3b** in ACN under non-degassed conditions the triplet state decays with a lifetime of 161 ns in ACN (**Fig. S11g-i**). In the absence of molecular oxygen, the triplet lifetime becomes significantly longer demonstrating that the lifetime under non-degassed conditions is also in this case mainly determined by a diffusion-controlled reaction with molecular oxygen forming mainly singlet oxygen after energy transfer on encounter (**Fig. S11j-l**). Accordingly, the intrinsic back intersystem crossing rate is given by the lifetime under degassed conditions. Then, the efficiency for singlet oxygen formation from the triplet state can be calculated to $1 - (2.4\ \mu s)^{-1}/(161\ ns)^{-1}$ = 93% for **3b** in ACN. Considering the known triplet yield of 100% for triplet formation in Ir complexes, the singlet oxygen formation efficiencies from the triplet state correspond also to the absolute quantum yield for this process.

Supplementary Figure 12: Cell microscopy image of deazaflavin 16

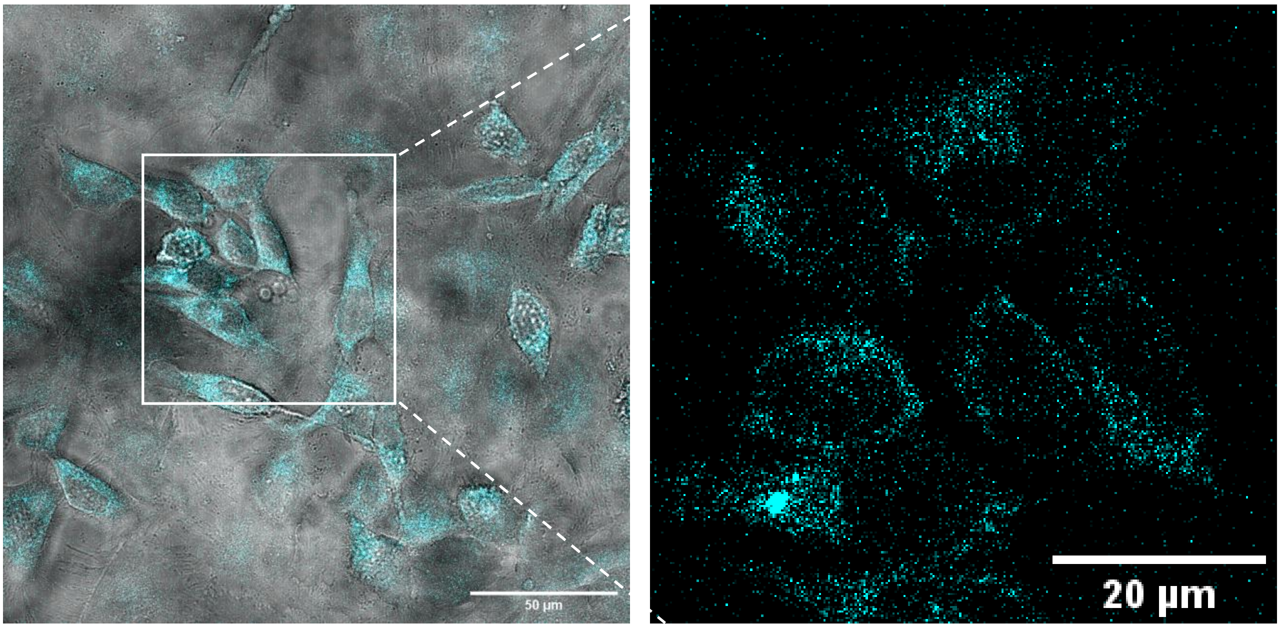

HeLa cells treated with deazaflavin **16** (20 µM) for 1 h. Images captured by laser scanning confocal microscopy. Scale bar = 50 µm and 20 µm (enlarged section).

Supplementary Figure 13: Optimisation of intracellular DarT-labelling irradiation time using deazaflavin-R_{10} conjugates

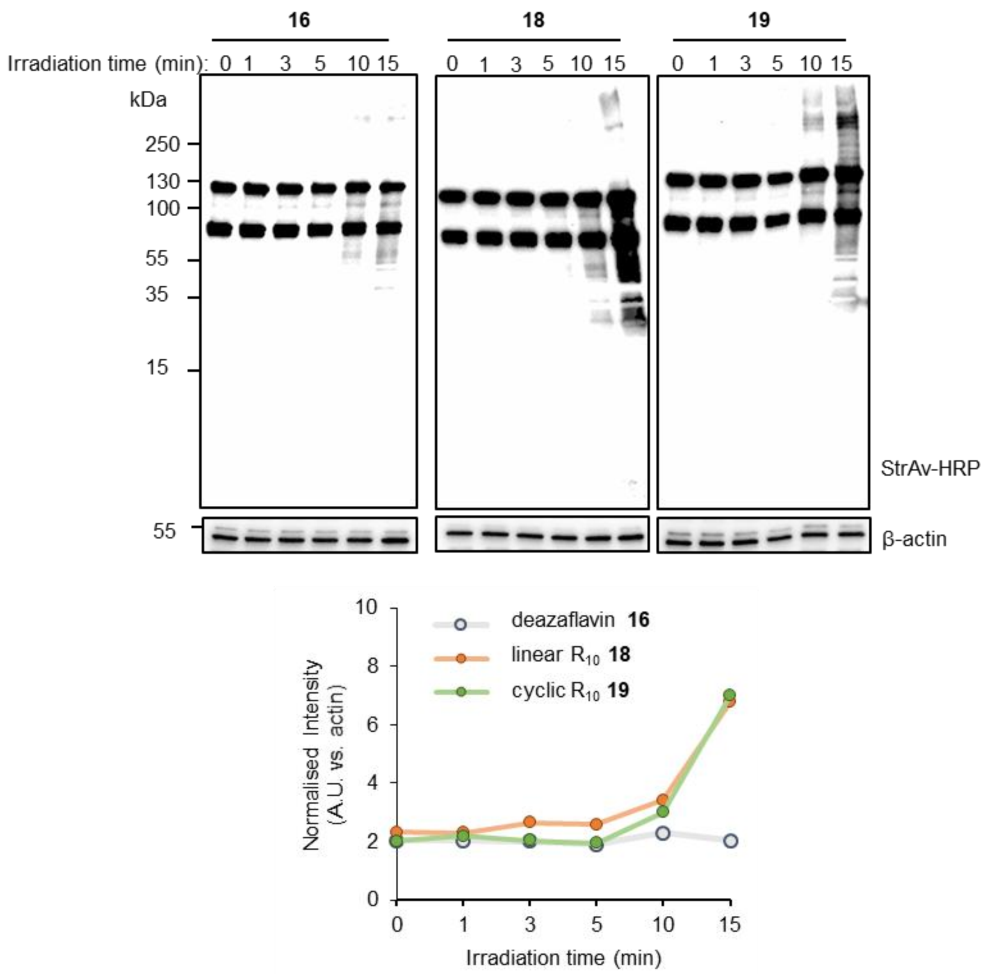

(**a**) Representative western blot of HeLa cell lysate after treatment with **16**, **18**, or **19** (5 µM) for 1 h followed by diazirine **8** (250 uM) and increasing irradiation time. Representative blot of two biological replicates. (**b**) Normalised labelling intensities of each lanes against loading control (β-actin). Assay performed twice independently with similar results.

Supplementary Figure 14: Western blot of fractionated cells lysates after intracellular DarT-labelling with deazaflavin-R$_{10}$ conjugates

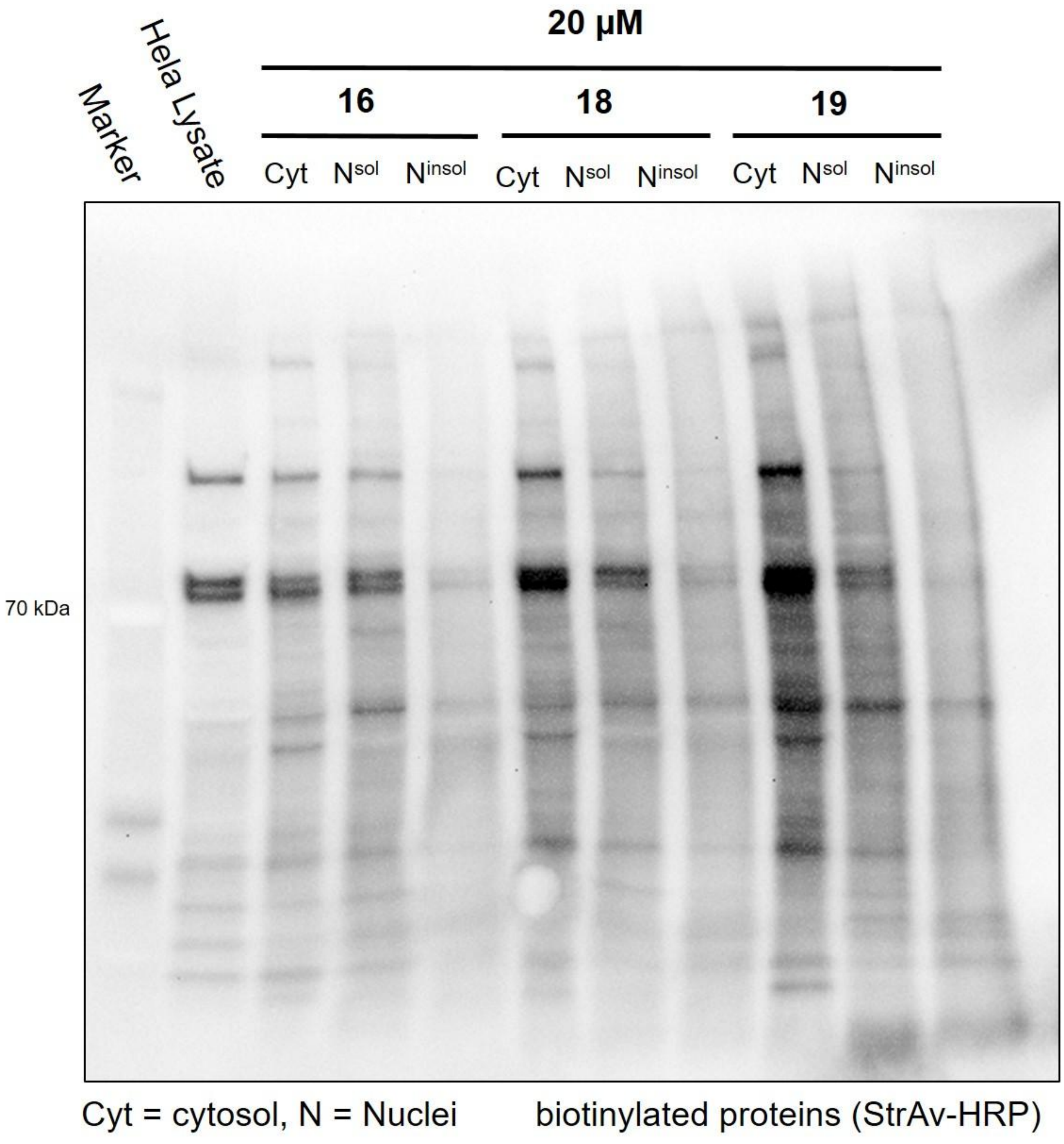

Representative western blot of fractionated HeLa cell lysate fractions after treatment with **16**, **18**, or **19** (20 µM) for 1 h followed by diazirine **8** (250 µM) and 15 min irradiation. The assay was performed twice independently with similar results.

Supplementary Figure 15: Comparison of linear and cyclic R_{10} GO terms

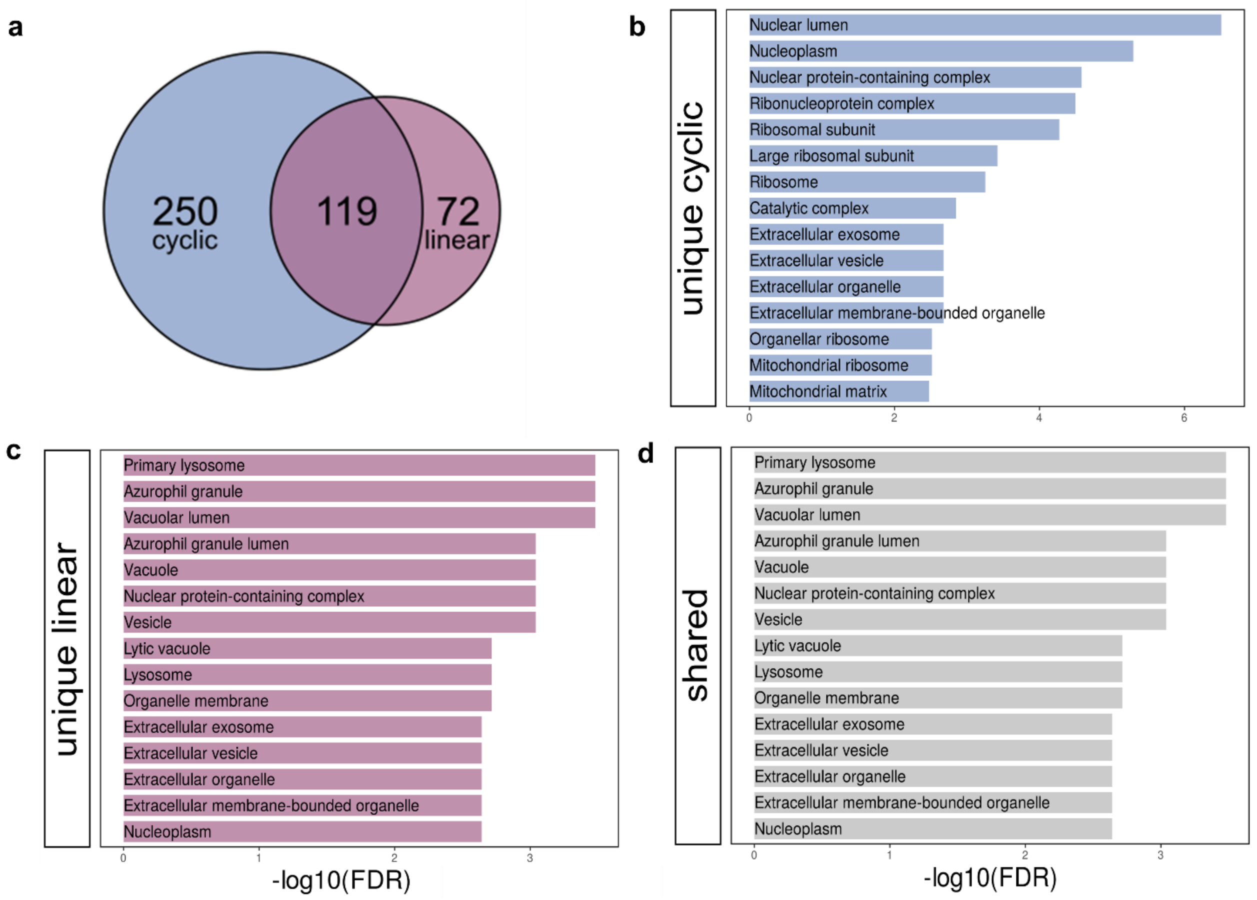

(**a**) Venn diagram comparing significantly enriched proteins after DarT labelling for linear (**18**) and cyclic (**19**) R_{10} data sets. (**b**) GO terms (cellular compartment) for significantly enriched proteins unique to cyclic R_{10} (**19**). (**c**) GO terms (cellular compartment) for significantly enriched proteins unique to linear R10 (**18**). (**d**) GO terms (cellular compartment) for significantly enriched proteins shared between cyclic and linear CPPs.

Supplementary Figure 16: Scatter plot of protein abundance ranked by intensity

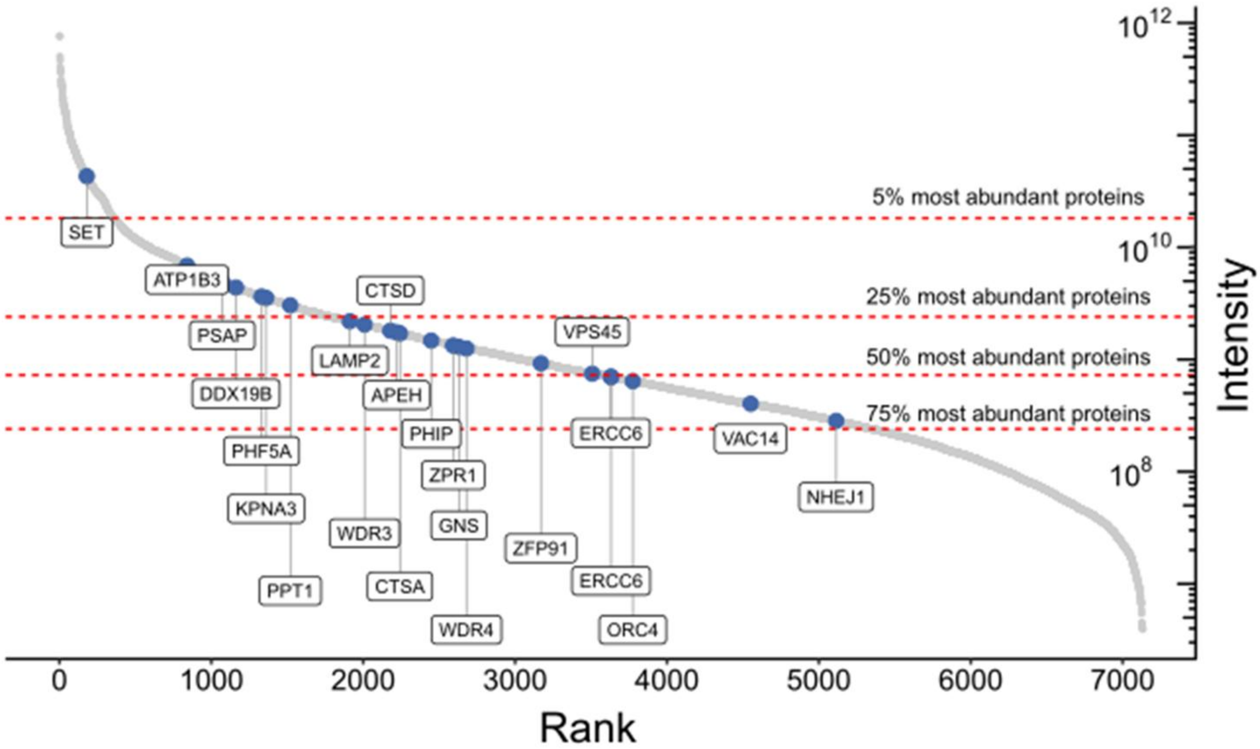

Intensity-based ranking of protein abundance from HeLa cells from a 180 min standard proteomics acquisition (Orbitrap) and sorted from largest to smallest with all proteins assigned an abundance rank. The most significantly enriched proteins from DarT-enabled CPP interactome (1 h incubation) mapping are highlighted.

Supplementary Figure 17: Intracellular fate of R_{10} peptides

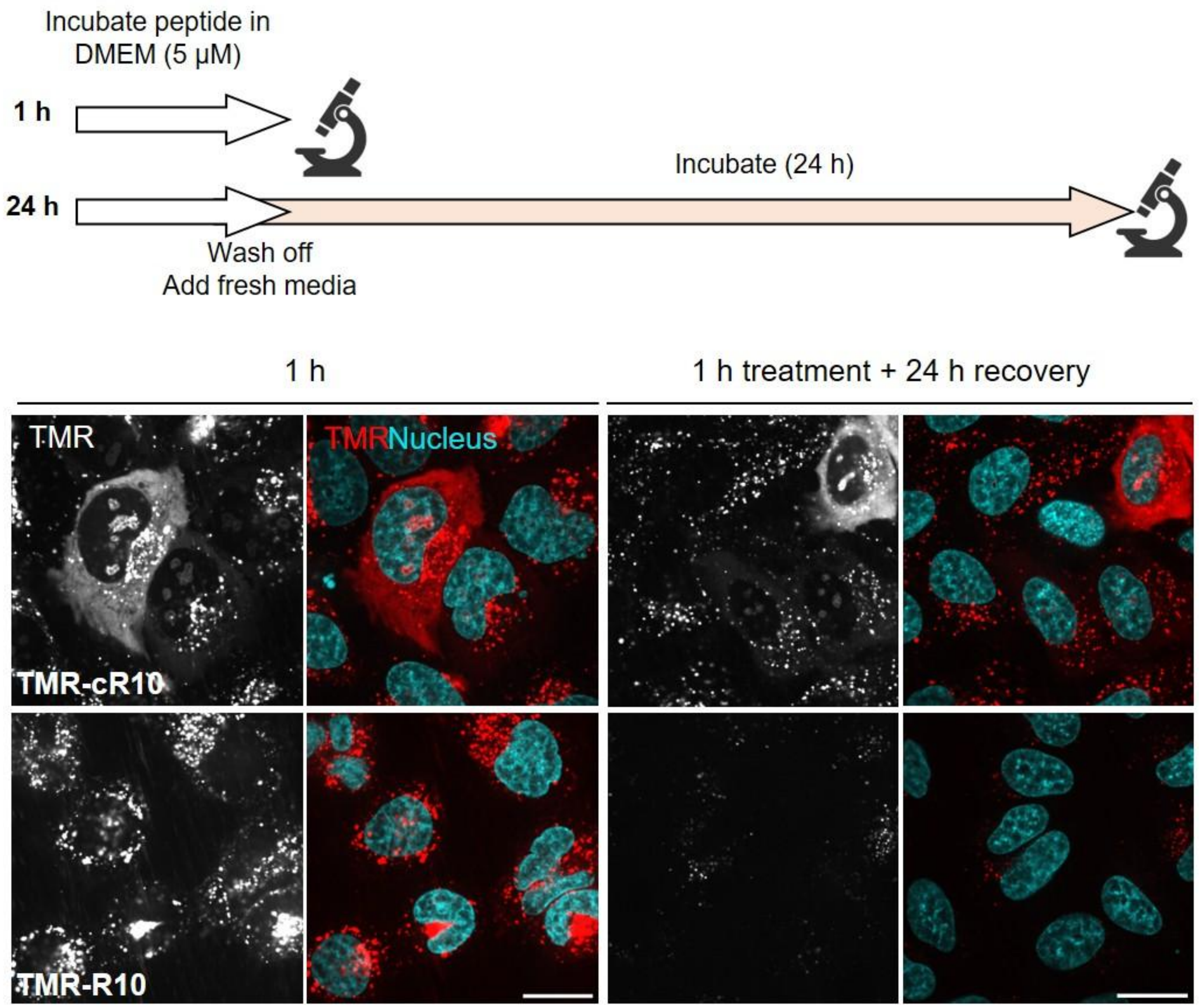

Representative confocal images of HeLa cells treated with 5 µM TMR-R_{10} or TMR-cyclic R_{10} for 1 h followed by 24 h post-treatment recovery in complete cell culture medium. Scale bar at 20 µm. The assay was repeated twice with similar results.

Supplementary Figure 18: Comparison of GO terms over time

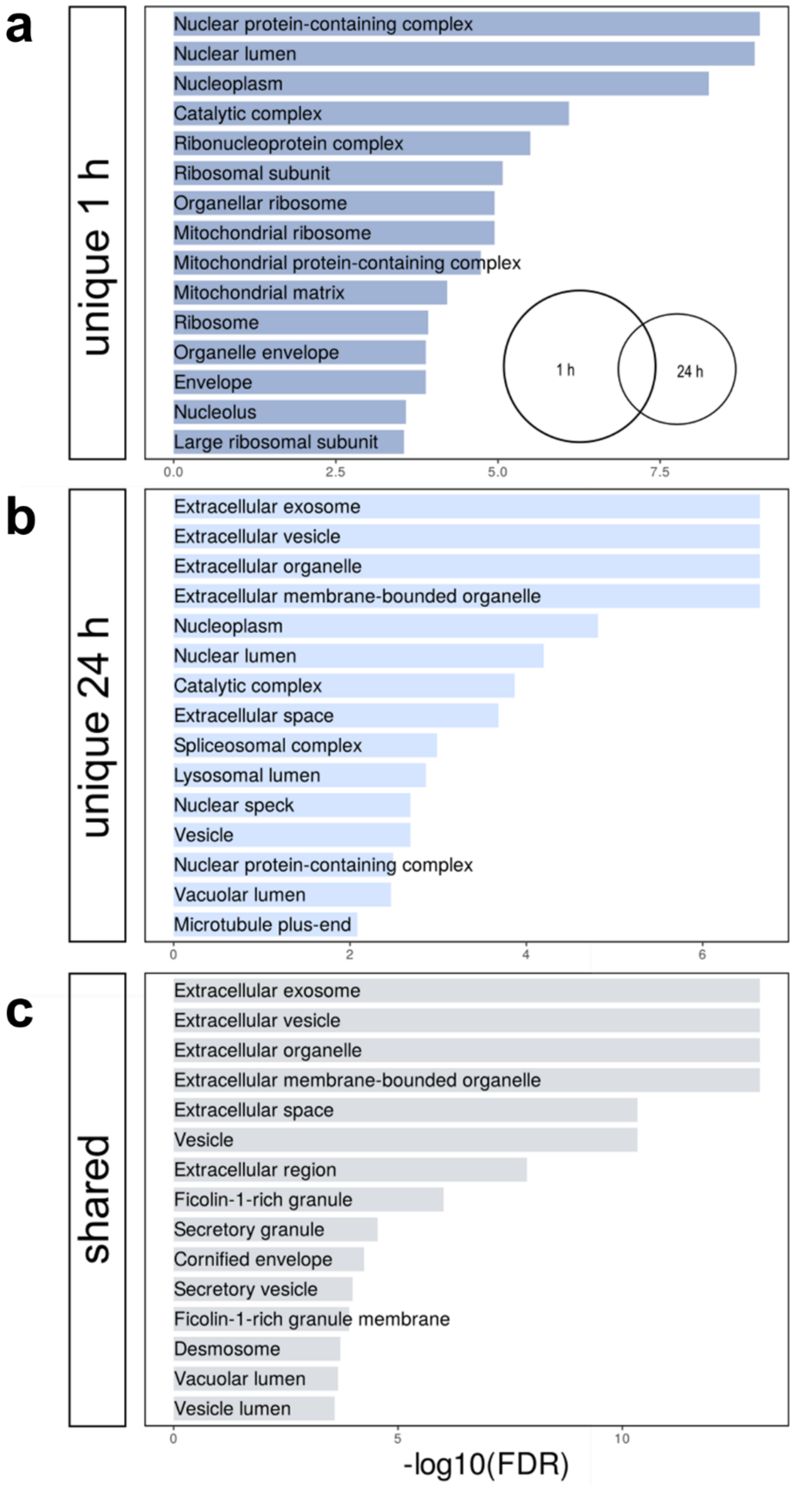

GO terms (cellular compartment) for significantly enriched proteins after DarT labelling of cyclic R_{10} **19** that are: (**a**) unique after 1 h; (**b**) unique after 24 h post-treatment and (**c**) shared between both time points.

2 General Information

2.1 Light source and irradiation setups

The light source used for experiments was an EvoluChem 450 nm Blue LED (30W, λ_{max} = 445 nm, Hepatochem, US) with emission spectrum as shown in **Figure S1a-b**. Photocatalyst screening was performed using the EvoluChem PhotoRedOx Box™. Cells were illuminated inside a 96 well plate using the EvoluChem PhotoRedOx Box™ at room temperature. To ensure equal distribution of light intensity only a 6x3 section of the plate was used:

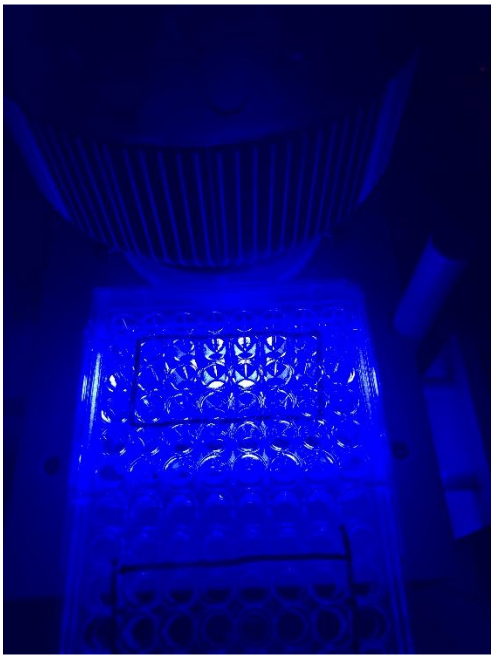

Irradiation of cell experiments using 10 cm dishes were performed in a cold room (4 ^{o}C) using one LED lamp emitting 450 nm (30W, EvoluChem), per dish at ~5 cm above the dish:

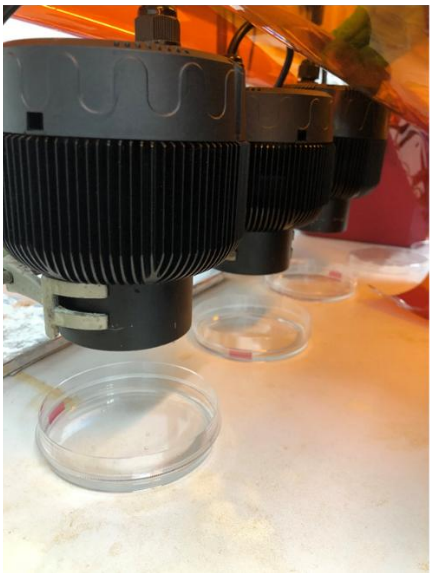

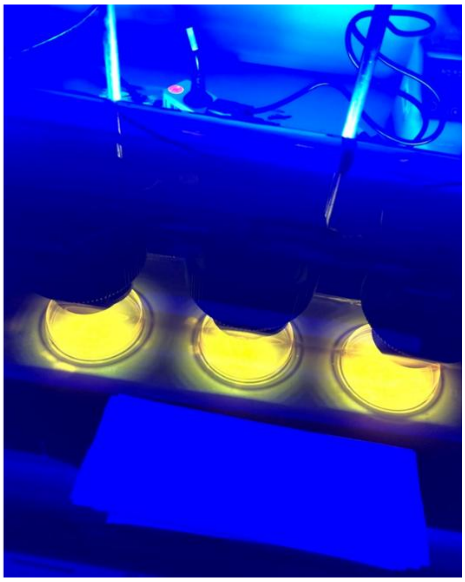

2.2 Materials

All materials were purchased from either Fisher Scientific (DE), BLD Pharma (DE), Sigma-Aldrich (DE) or TCI Chemicals (BE) in the highest purity available and used without further purification. For column chromatography 40-53 µm silica gel (VWR) was used as stationary phase. Analytical thin layer chromatography (TLC) was performed on aluminum foil pre-coated with SiO_2-60 F254 (Macherey-Nagel) and visualized with a UV-lamp (254 and 365 nm).

2.3 Characterisation techniques

NMR spectra were either measured with a Bruker UltrashieldTM 300 MHz spectrometer, a Bruker AVIII instrument or a Bruker UltrashieldTM 600 MHz (all Bruker Corp., USA) at ambient temperature. The resonance multiplicity is abbreviated as: s (singlet), d (doublet), t (triplet), q (quadruplet), quint (quintet), sext (sextet), sep (septet), m (multiplet) and br (broad). The chemical shift δ is expressed in "ppm" and the coupling constant J is in "Hz". The chemical shifts are referenced to the residual solvent peak as the internal standard. High resolution mass spectra (HRMS) was recorded on an Acquity UPLC® system (Water Corp., USA) with a Xevo® G2-XS Quadrupole time-of-flight (QTof) mass spectrometer (Water Corp., USA). The following gradient was used A = H_2O + 0.1% formic acid, B = MeCN + 0.1% formic acid, 10 → 90% B. For small molecules and peptides, an Acquity UPLC BEH C18 column (1.7 µm, 2.1 mm x 50 mm) was used. For intact protein MS a ACQUITY UPLC Protein BEH C4 Column, 300 Å, 1.7 µm, 2.1 mm x 100 mm was used. Proteins were eluted with a flow rate of 0.3 mL/min. The following gradient was used: A: 0.01% FA in H_2O; B: 0.01% FA in MeCN. 5-95% B 0-6 min. Mass analysis was conducted with a Waters XEVO G2-XS QTof analyzer. Raw data was analyzed with MaxEnt 1. UV-Vis absorption spectra were obtained with a JASCO Spectrophotometer.

2.4 Preparative HPLC

Preparative HPLC of peptides was done on a Gilson PLC 2020 system using a Nucleodur C18 Htec Spum column (Macherey-Nagel, 100 A, 5 m, 250 mm x 32 mm, 30 mL/min). The following gradient was used in all purifications: A = H_2O + 0.1% trifluoroacetic acid (TFA), B = MeCN + 0.1% TFA 5% B 0-10 min, 5-50% B 10-60 min, 50-95% 60-80 min.

2.5 Analytical HPLC

Analytical HPLC for photocatalyst performance screening was conducted on a Shimadzu prominence HPLC system (Shimadzu Corp., Japan) with a CBM-20A communication bus module, a SIL-20A auto sampler, 2 pumps LC-20AT, and a SPDM20A UV/VIS detector, a CTO-20A column oven, using a Macherey-Nagel (DE) Nucleodur C18 EC HPLC column (5 µm, 250 x 4 mm) with a flow rate of 1.0 mL/min. The following mobile phase gradient was used for analysis where A = H_2O + 0.1% TFA and B = MeCN + 0.1% TFA: 5-75% B in 25 min, 75-95% B during 25-30 min, 95% B hold at 30-35 min then 95-5% B during 35-45 min.

2.6 Plasmid information

The plasmid pcDNA5_FRTcyto_Halo-SNAP-meGFP encoding a fusion protein expressed in the cytosol consisting of HTP, SNAP and meGFP was designed and constructed by Gibson assembly from pcDNA5_FRTcyto_Halo-SNAP (ref PMID: 31792385), and sequence verified by Sanger sequencing. The protein sequence is annotated below.

MEGGLGRAVCLLTGASRGFGRTLAPLLASLLSPGSVLVLSARNDEALRQLEAELGAERSGLRVVRVPADLGAEAGL
QQLLGALRELPRPKGLQRLLLINNAGSLGDVSKGFVDLSDSTQVNNYWALNLTSMLCLTSSVLKAFPDSPGLNRTV
VNISSLCALQPFKGWALYCAGKAARDMLFQVLALEEPNVRVLNYAPGPLDTDMQQLARETSVDPDMRKGLQELKAK
GKLVDCKVSAQKLLSLLEKDEFKSGAHVDFYDKEFGSEIGTGFPFDPHYVEVLGERMHYVDVGPRDGTPVLFLHGN
PTSSYVWRNIIPHVAPTHRCIAPDLIGMGKSDKPDLGYFFDDHVRFMDAFIEALGLEEVVLVIHDWGSALGFHWAK
RNPERVKGIAFMEFIRPIPTWDEWPEFARETFQAFRTTDVGRKLIIDQNVFIEGTLPMGVVRPLTEVEMDHYREPF
LNPVDREPLWRFPNELPIAGEPANIVALVEEYMDWLHQSPVPKLLFWGTPGVLIPPAEAARLAKSLPNCKAVDIGP
GLNLLQEDNPDLIGSEIARWLSTLEISGSGRPPPPPPPPPPPPPPPPPPPPPPPPPPPPPPGGRSRSLEMDKDCEM
KRTTLDSPLGKLELSGCEQGLHEIIFLGKGTSAADAVEVPAPAAVLGGPEPLMQATAWLNAYFHQPEAIEEFPVPA
LHHPVFQQESFTRQVLWKLLKVVKFGEVISYSHLAALAGNPAATAAVKTALSGNPVPILIPCHRVVQGDLDVGGYE
GGLAVKEWLLAHEGHRLGKPGLGKLVSKGEELFTGVVPILVELDGDVNGHKFSVSGEGEGDATYGKLTLKFICTTG
KLPVPWPTLVTTLTYGVQCFSRYPDHMKQHDFFKSAMPEGYVQERTIFFKDDGNYKTRAEVKFEGDTLVNRIELKG
IDFKEDGNILGHKLEYNYNSHNVYIMADKQKNGIKVNFKIRHNIEDGSVQLADHYQQNTPIGDGPVLLPDNHYLST
QSKLSKDPNEKRDHMVLLEFVTAAGITLGMDELYK*

SPR
Halo-tag
SNAP-tag
meGFP

2. pcDNA3_HaloTag-mGluR2 (ref PMID: 39610654). Sequence verified by Sanger sequencing.

2.7 General cell culture

Cell lines were maintained at 37 °C, 5% CO_2 in a humidified atmosphere and split every 2 days or until confluency reached 90%. SK-BR-3 (LMU München Biozentrum) cells were maintained in Dulbecco's Modified Eagle Medium (DMEM) medium with F-12 modification, stable L-glutamine, and 10 % fetal calf serum (FCS). HeLa (American Type Culture Collection, CCL2) and HEK293T (Leibniz-Institut DSMZ – Deutsche Sammlung von Mikroorganismen und Zellkulturen) cells were maintained in DMEM (High glucose) with 10% FCS.

2.8 Transfection

Cells were seeded into 6 cm dishes and were allowed to attach and reach 80% confluency. Plasmid (2.5 μg) and PLUS™ Reagent (1 μL/μg plasmid) was combined in 250 μL OptiMEM Reduced Serum Medium. In a separate tube, Lipofectamine LTX (12.5 μL) was added to 250 μL OptiMEM Reduced Serum Medium. The plasmid and the Lipofectamine solutions were combined, vortexed, and allowed to incubate at ambient temperature for 5 min. This combined solution was added directly to the prepared cells. For extracellular DarT labelling of HaloTag-mGluR2-expressing HeLa cells, cells were incubated for 48 h post-transfection. For the modified CAPA assay, transfected cells were harvested after 24 h and seeded into 24-well plates, and incubated for an additional 24 h before performing the assay.

2.9 LC-MS/MS

LC-MS/MS analysis was performed on an UltiMate 3000 RSLC nano LC system coupled on-line to an Orbitrap Fusion mass spectrometer or a Vanquish Neo coupled on-line to an Exploris 480 mass spectrometer (Thermo Fisher Scientific). For sample loading a PepMap C-18 trap-column (Thermo Fisher Scientific) of 0.075 mm ID x 50 mm length, 3 µm particle size and 100 Å pore size was used. The loading mobile phase A contained 1% ACN and 0.1% formic acid (FA) in water, and mobile phase B 0.1% FA in ACN. Reversed-phase separation was performed using a 50 cm analytical column (in-house packed with Poroshell 120 EC-C18, 2.7µm, Agilent Technologies) with mobile phase A contained 0.1% FA in water, and mobile phase B 0.1% FA in ACN using a 93 minutes gradient (4-5%B 0-8 minutes; 5-25%B in 8-74 minutes; 25-28%B 74-80 minutes; 28-31%B 80-86 minutes; 31-36%B 86-92 minutes; 36-40%B 92-95 minutes; 40-50%B 95-96 minutes; 50-80%B 96-101 minutes; 80%B 101-104 minutes; 80-4%B 104-104.1 minutes). Data was acquired using survey scans in a range of 375 to 1500 m/z with a resolution of 120k, an AGC target value of 4e5 and 50 ms maximum injection time. Precursor ions with charge states 2-5 were isolated with a mass selecting quadrupole (isolation window 1.6 m/z) with 40 sec dynamic exclusion (+/- 10 ppm). Precursor ions were fragmented using higher-energy collisional dissociation (HCD) applying a normalized collision energy (NCE) of 30%. The maximum injection time was set to 35 ms to collect 1e4 precursor ions. Fragment ion spectra were acquired in the Orbitrap at 30K resolution (AGC target value 5e4, 54 ms maximum injection time).

3 Experimental Procedures

3.1 Organic Synthesis

3.1.1 Photocatalysts

Thioxanthones, (**2a** and **2b**) were prepared following literature procedures.[10,11] Alloxazine (**S2**) was prepared as described in literature.[12] Iridium catalysts prepared according to referenced literature: Ir[dFCF$_3$CO$_2$Hppy]$_2$[diolbpy] (**3a**)[13,14] and Ir-G2-PEG$_4$-chloroalkane (**17**).[15] [Ir(dF(CF$_3$)ppy)$_2$(dtbbpy)]PF$_6$ (**3b**) was purchased from Sigma-Aldrich (DE).

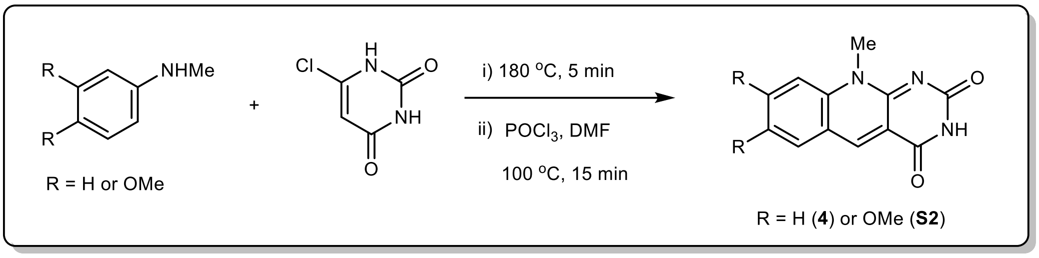

Scheme S1: Synthesis of deazaflavins.

10-Methylpyrimido[4,5-*b*]quinoline-2,4(3*H*,10*H*)-dione (4):

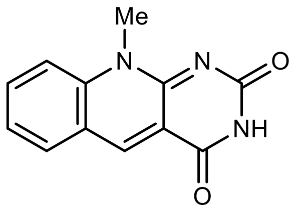

4 was prepared in a two-step procedure using modified protocols:[16] *N*-Methylaniline (5.00 mL, 46.2 mmol) and 6-chlorouracil (3.33 g, 22.7 mmol) were stirred under Ar for 5 minutes at 180 °C. The mixture was then allowed to cool to room temperature and the resulting precipitate was triturated using a mixture of methanol/diethyl ether (1:5, v/v) to give a white solid (4.90 g, 22.6 mmol) that was used in the following step without further purification. The resulting solid (4.9 g, 22.6 mmol) was suspended in DMF (40 mL) and POCl$_3$ (4.22 mL, 45.2 mmol) was added dropwise. The resulting suspension was stirred at room temperature for 1 hour, during which time the suspension gradually dissolved to form a yellow solution. The solution was then heated to 100 ˚C for 15 min to ensure complete cyclisation. The mixture was cooled to room temperature and then poured onto ice-water and neutralised with NaHCO$_3$. The resulting yellow precipitate was collected by filtration and dried under high vacuum (<0.1 mbar) (3.88 g, 17.1 mmol, 88% over 2 steps).

^{1}H NMR (600 MHz, DMSO-*d*$_6$) δ = 11.06 (s, 1H), 8.97 (s, 1H), 8.16 (dd, *J* = 7.9, 1.5 Hz, 1H), 7.98 – 7.89 (m, 2H), 7.56 – 7.50 (m, 1H), 4.03 (s, 3H) ppm.

^{13}C NMR (151 MHz, DMSO-*d*$_6$) δ = 162.1, 157.5, 156.5, 141.5, 140.8, 135.3, 131.6, 124.4, 120.9, 116.7, 115.1, 32.2 ppm.

HRMS (ESI) m/z: [M + Na]$^+$ Calcd for $C_{12}H_9N_3O_2Na$ 250.0587; Found 250.0649.

^{1}H NMR spectrum of **4**:

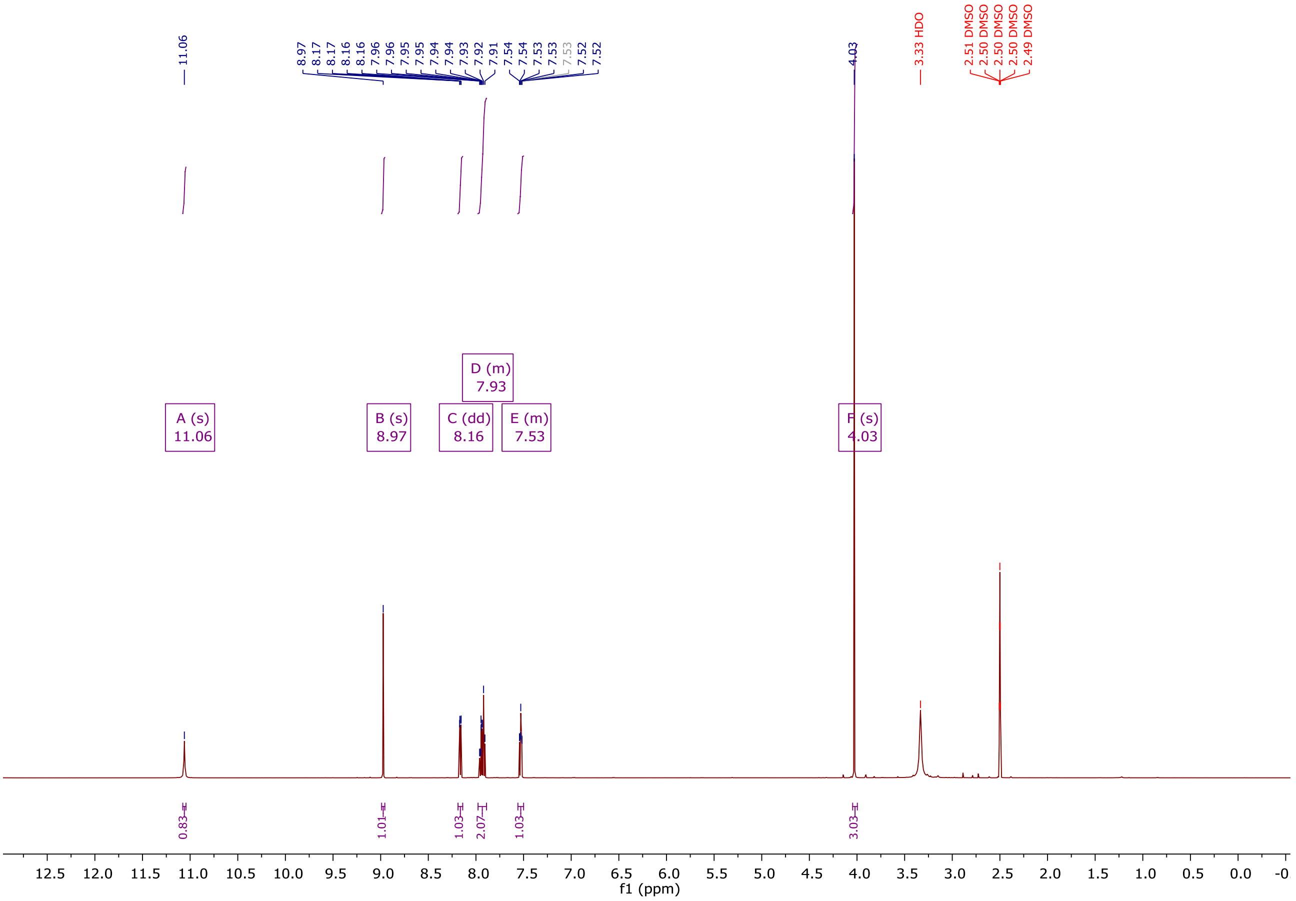

^{13}C NMR spectrum of **4**:

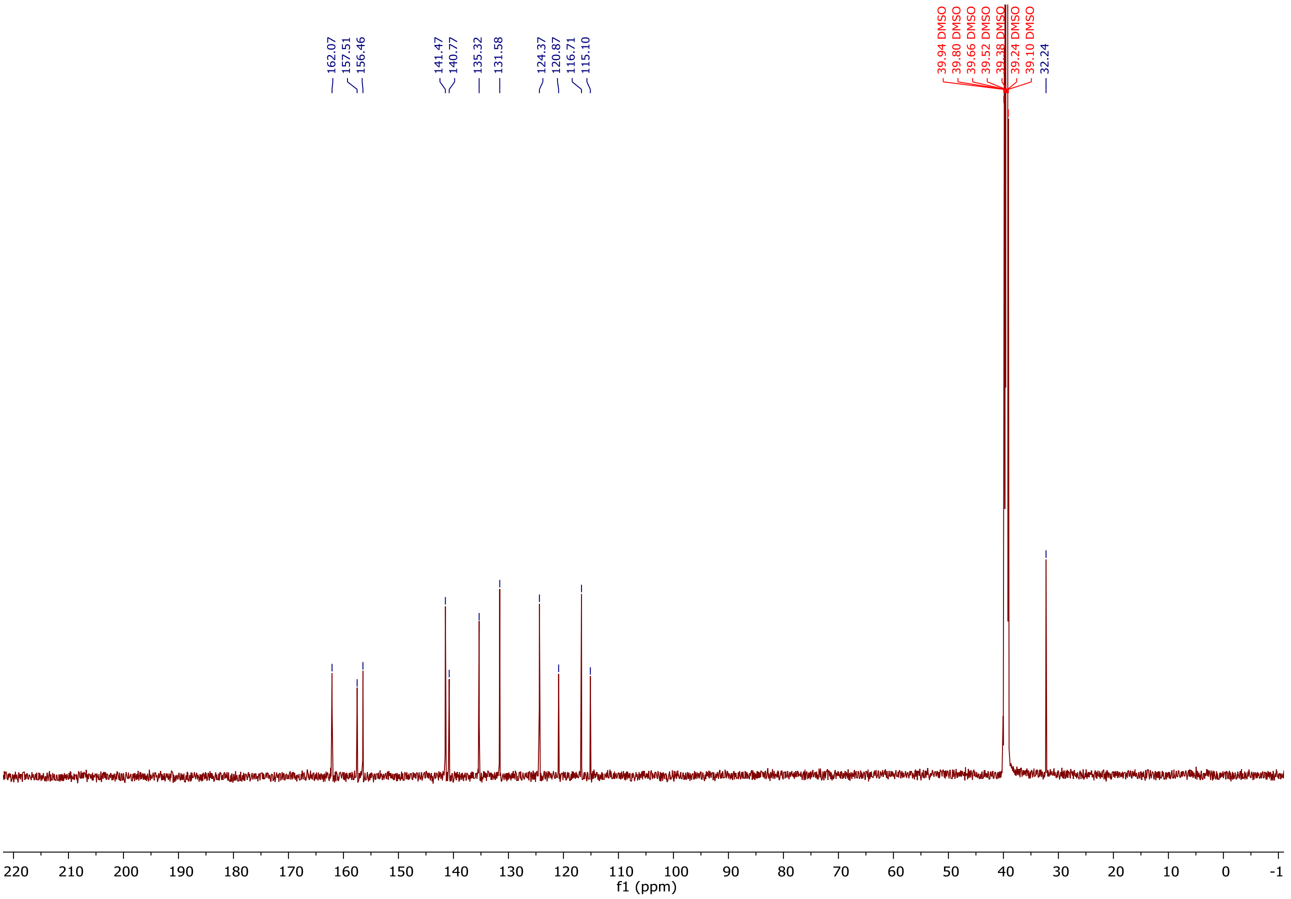

7,8-Dimethoxy-10-methylpyrimido[4,5-b]quinoline-2,4(3H,10H)-dione (diMeO-dFl) (S1)

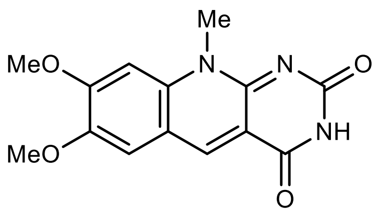

3,4-Dimethoxy-N-methylaniline (1.672 g 10 mmol)[17] and 6-chlorouracil (0.484 g, 3.3 mmol) were stirred under Ar for 10 minutes at 180 °C. The mixture was then allowed to cool to room temperature and the resulting precipitate was triturated in diethyl ether to give a white solid (0.915 g, 3.3 mmol) that was used in the following step without further purification. The resulting solid (0.915 g, 3.3 mmol) was suspended in DMF (33 mL, 0.1 M) and $POCl_3$ (0.466 mL, 5 mmol) was added dropwise. The resulting suspension was stirred at room temperature for 1 hour, during which time the suspension gradually dissolves to form a yellow solution. The solution was then heated to 100 °C for 30 min to ensure complete cyclisation. After cooling to room temperature, the yellow precipitate was filtered and recrystallized in a mixture of ethanol and dichloromethane (DCM) (1:2, v/v). The obtained product was collected by filtration and dried under high vacuum (<0.1 mbar) (0.646 g, 2.2 mmol, 67 % over 2 steps).

^{1}H NMR (600 MHz, TFA_2H) δ = 9.97 (s, 1H), 8.16 (s, 1H), 8.07 (s, 1H), 4.97 (s, 3H), 4.77 (s, 3H), 4.65 (s, 3H).

^{13}C NMR (151 MHz, TFA_2H) δ = 163.00, 161.17, 151.71, 150.46, 146.50, 145.49, 139.91, 121.78, 110.03, 108.31, 98.26, 57.19, 56.28, 36.61.

HRMS (ESI) m/z: $[M + H^{+}]^{+}$ Calcd for $C_{14}H_{14}N_{3}O_{4}$ 288.0979; Found 288.0911.

^{1}H NMR of **S1**:

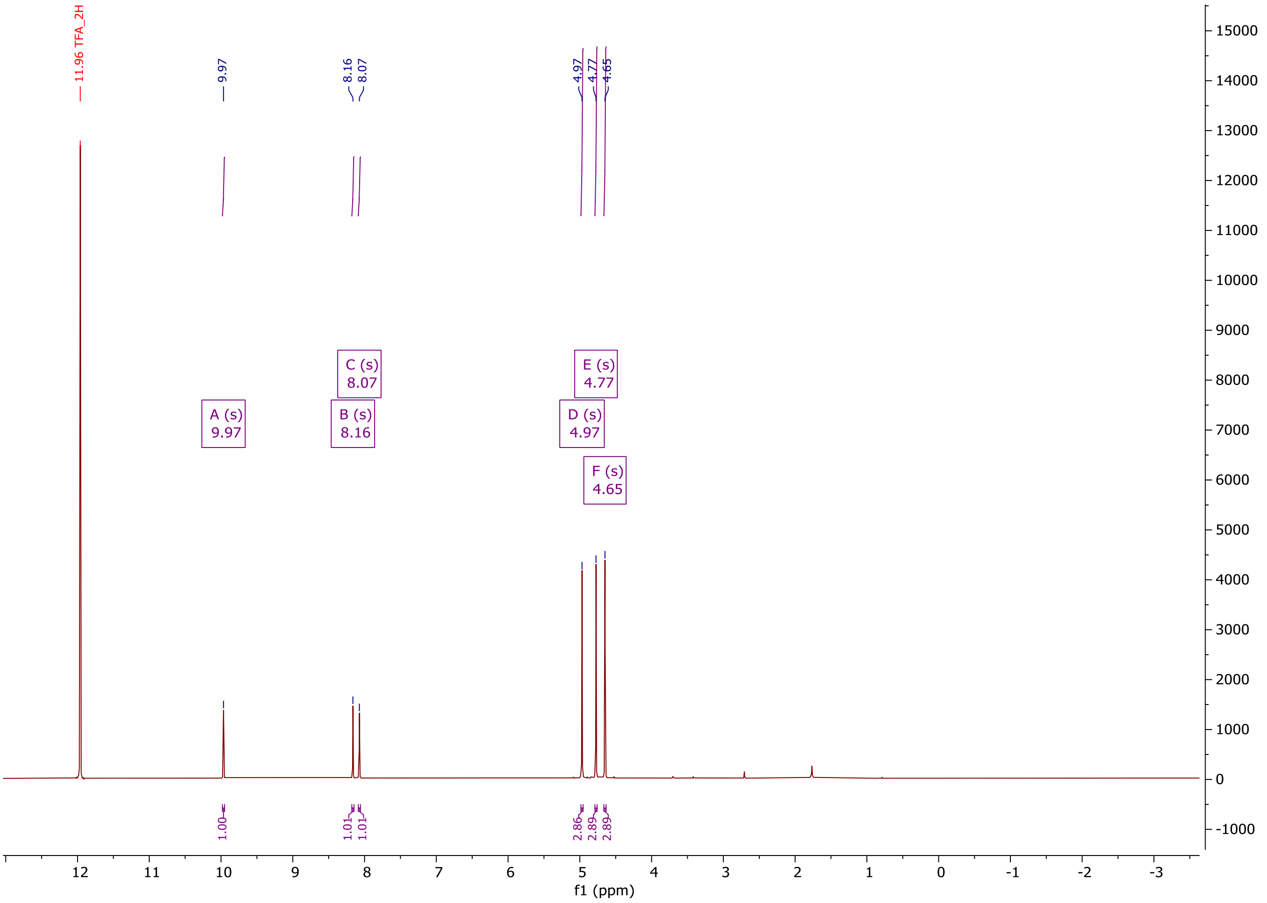

^{13}C NMR of **S1**:

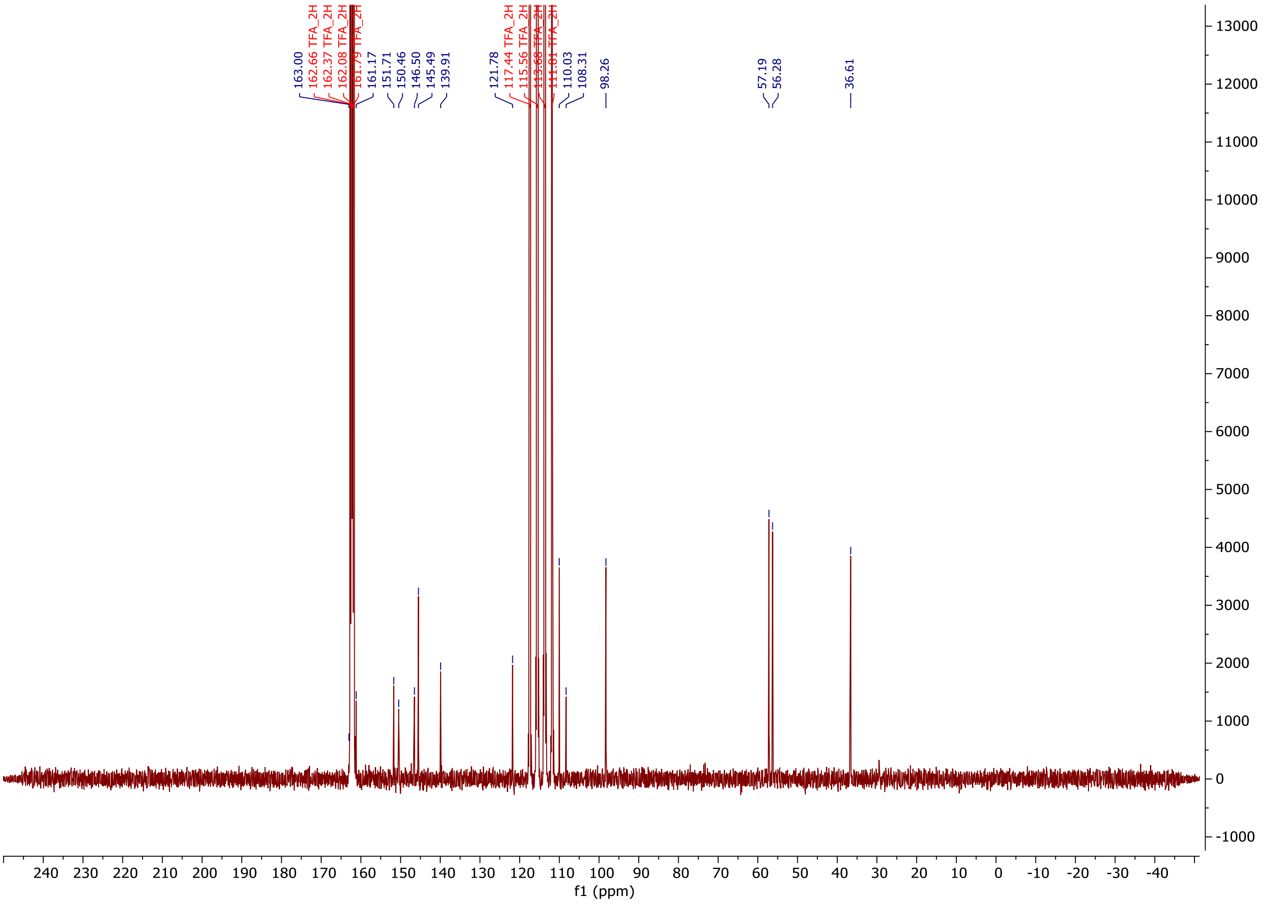

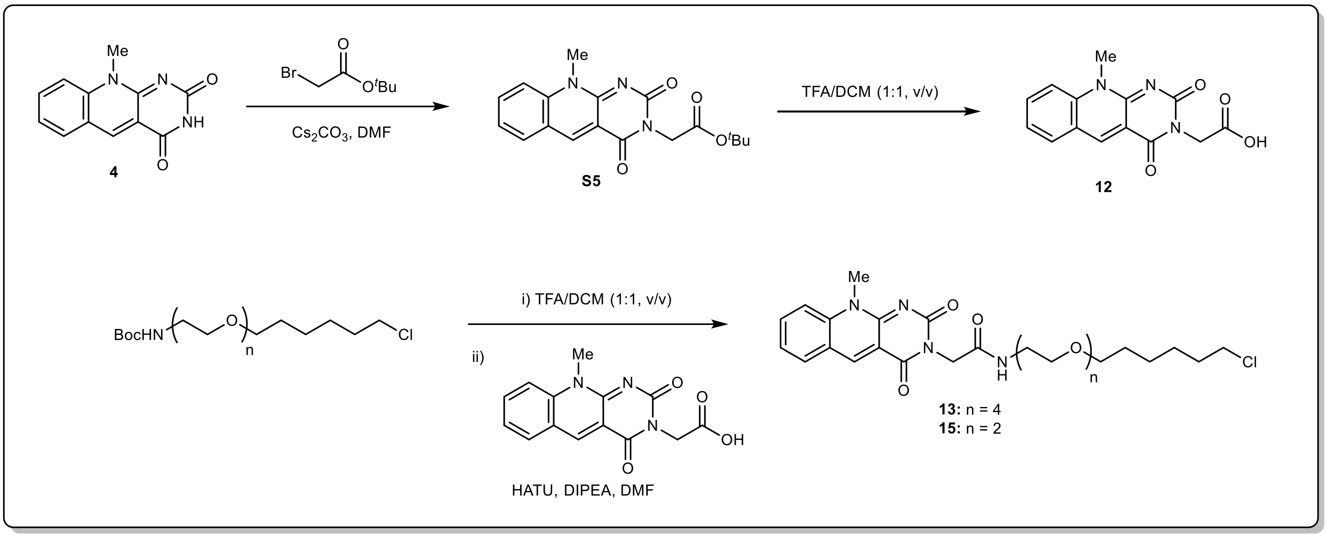

Scheme S2: Synthesis of **11** and chloroalkane-deazaflavins **12** and **16**.

***tert*-Butyl 2-(10-methyl-2,4-dioxo-4,10-dihydropyrimido[4,5-*b*]quinolin-3(2*H*)-yl)acetate (S5):**

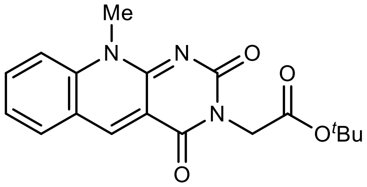

tert-Butyl bromoacetate (1.20 mL, 8.00 mmol) was added dropwise to a suspension of **4** (0.45 g, 2.00 mmol) and dry Cs_2CO_3 (0.98 g, 3.0 mmol) in dry DMF (20 mL) at 45 °C under Ar atomosphere. The resulting mixture was stirred at 45 °C for 16 h. The reaction mixture was then diluted with $CHCl_3$ (200 mL) and washed with water (3 x 100 mL). The organic phase was then dried over Na_2SO_4, filtered and concentrated *in vacuo*. The resulting yellow material was purified by silica gel column chromatography (0-10% EtOAc in DCM) to obtain the title compound as yellow needles (0.54 g, 1.57 mmol, 79%).

^{1}H NMR (600 MHz, $CDCl_3$) δ = 8.87 (s, 1H), 7.89 (dd, *J* = 8.3, 6.4 Hz, 2H), 7.69 (dt, *J* = 9.1, 0.9 Hz, 1H), 7.50 (ddd, *J* = 7.9, 7.2, 0.9 Hz, 1H), 4.72 (s, 2H), 4.15 (s, 3H), 1.47 (s, 9H) ppm.

^{13}C NMR (151 MHz, $CDCl_3$) δ = 167.1, 161.5, 156.6, 156.2, 142.7, 141.1, 135.6, 131.7, 124.8, 121.3, 116.0, 115.0, 82.0, 43.0, 32.5, 28.1 ppm.

HRMS (ESI) m/z: $[M + H]^+$ Calcd for $C_{18}H_{20}N_3O_4$ 342.1448; Found 342.1436.

^1H NMR of **S5**:

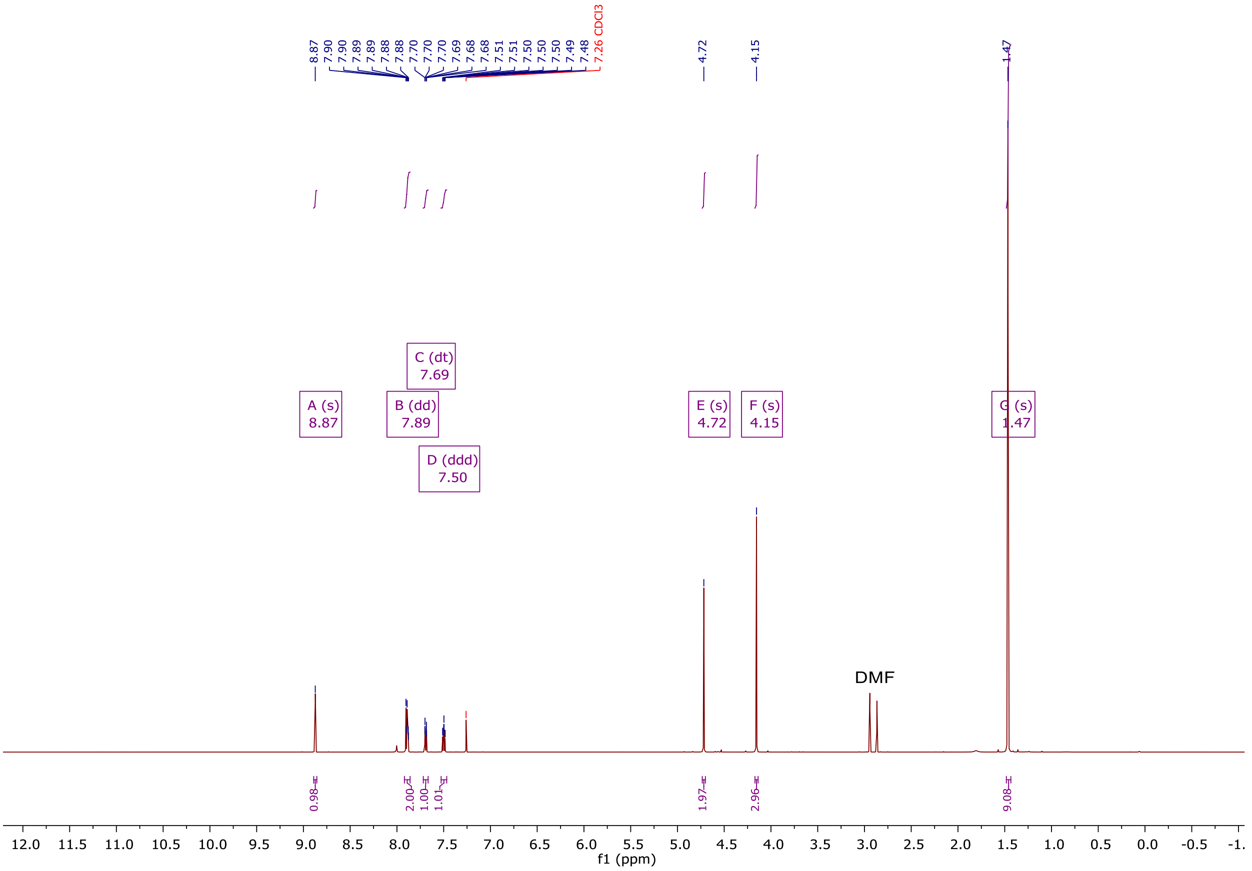

^{13}C NMR of **S5:**

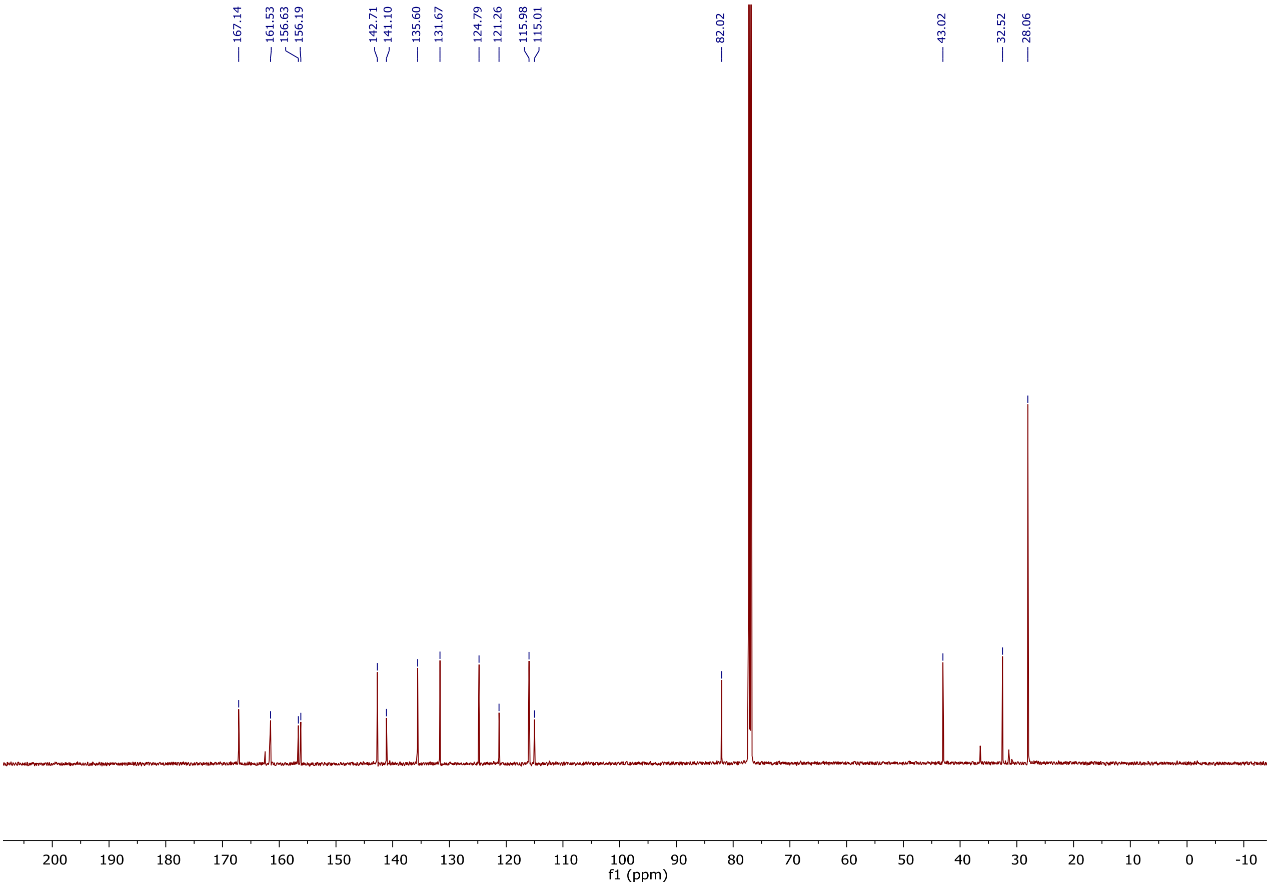

2-(10-Methyl-2,4-dioxo-4,10-dihydropyrimido[4,5-*b*]quinolin-3(2*H*)-yl)acetic acid (12):

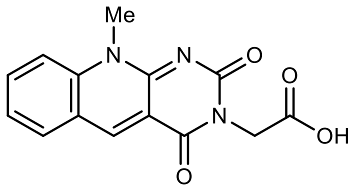

Deazaflavin **S3** (0.52 g, 1.52 mmol) was dissolved in TFA/DCM (1:1 *v/v*,15 mL) and stirred at room temperature for 6 h. The solvent was evaporated under a stream of N_2 and the product precipitated using DCM. The suspension was centrifuged to obtain a yellow solid (0.41 g, 95%).

^{1}H NMR (600 MHz, DMSO-d_6) δ = 9.06 (s, 1H), 8.21 (dd, *J* = 8.0, 1.4 Hz, 1H), 8.05 – 7.88 (m, 2H), 7.56 (td, *J* = 7.2, 6.6, 1.4 Hz, 1H), 4.55 (s, 2H), 4.06 (s, 3H) ppm.

^{13}C NMR (151 MHz, DMSO-d_6) δ = 170.0, 161.6, 156.4, 155.6, 143.2, 141.3, 136.2, 132.2, 125.0, 121.5, 117.3, 114.5, 42.3, 32.7 ppm.

HRMS (ESI) m/z: $[M + H]^+$ Calcd for $C_{14}H_{12}N_3O_4$ 286.0822; Found 286.0793.

^{1}H NMR of **12:**

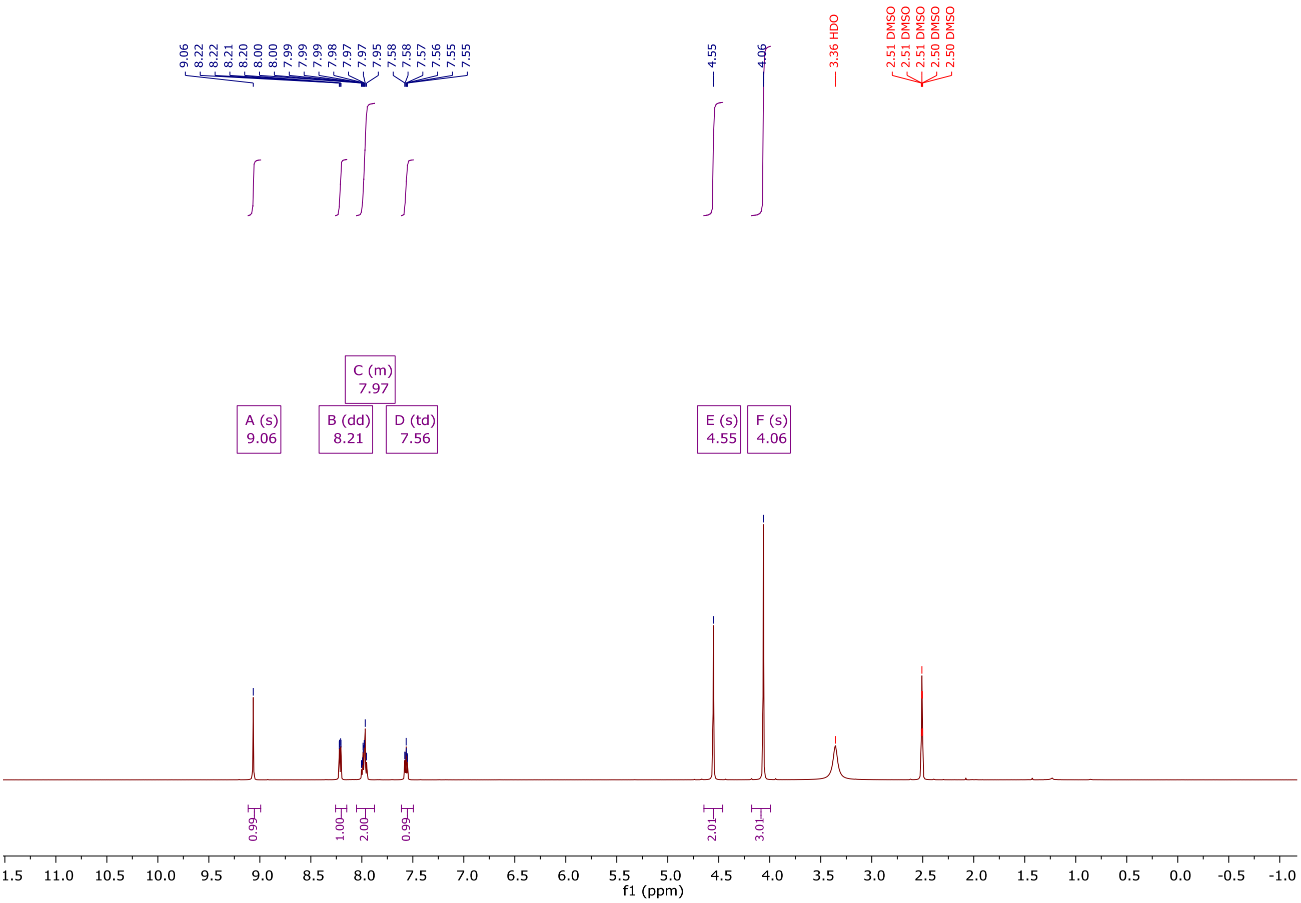

^{13}C NMR of **12:**

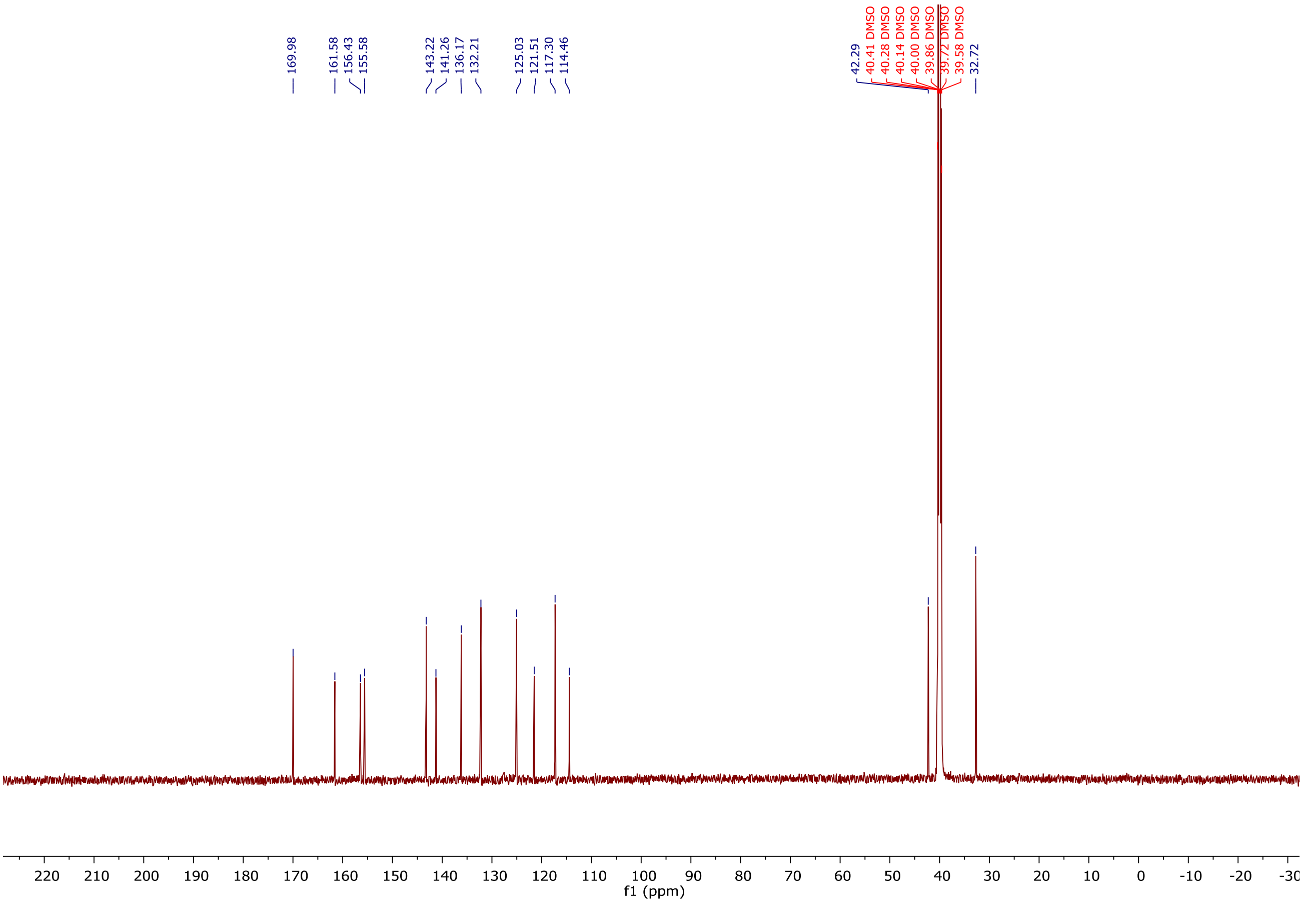

General procedure for deazaflavin-chloroalkane synthesis:

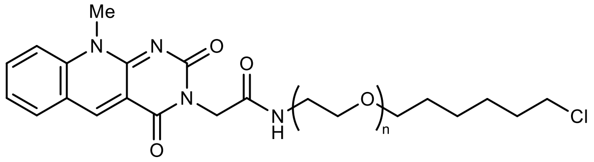

Chloroalkane-PEG$_n$-NHBoc (1.2 equiv.)[18] was dissolved in TFA/DCM (1:1, v/v, 0.1 M) and stirred at room temperature for 2 h. The solvent was then evaporated under a stream of N_2 to obtain a yellow oil that was dissolved in dry DMF (0.5 M) and added dropwise to a solution of deazaflavin **11** (1 equiv.), Hexafluorophosphate Azabenzotriazole Tetramethyl Uronium (HATU) (1.1 equiv.), *N,N*-diisopropylethylamine (DIPEA) (3 equiv.) in dry DMF (0.1 M). The solution was stirred at room temperature for 16 h under Ar atmosphere. The mixture was then diluted with DCM (50 mL) and washed with water (3 x 25 mL). The organic layer was then dried over Na_2SO_4, filtered and concentrated *in vacuo*. The residue was then purified by column chromatography (0-10% MeOH in DCM).

***N*-(18-chloro-3,6,9,12-tetraoxaoctadecyl)-2-(10-methyl-2,4-dioxo-4,10-dihydropyrimido[4,5-*b*]quinolin-3(2*H*)-yl)acetamide (13):** Prepared using chloroalkane-PEG$_4$-NHBoc (37.9 mg, 0.092 mmol) to obtain a yellow sticky solid (28.3 mg, 0.049 mmol, 54%).

^{1}H NMR (600 MHz, $CDCl_3$) δ = 8.93 (s, 1H), 7.96 – 7.91 (m, 2H), 7.73 (d, J = 8.7 Hz, 1H), 7.56 – 7.49 (m, 1H), 6.75 (t, J = 5.7 Hz, 1H), 4.78 (s, 2H), 4.19 (s, 3H), 3.70 (s, 4H), 3.67 (d, J = 9.2 Hz, 6H), 3.62 – 3.59 (m, 4H), 3.56 – 3.46 (m, 6H), 1.81 – 1.75 (m, 2H), 1.62 (dt, J = 14.8, 6.8 Hz, 2H), 1.50 – 1.42 (m, 2H), 1.44 – 1.34 (m, 2H) ppm. **^{13}C NMR** (151 MHz, $CDCl_3$) δ = 167.1, 161.7, 156.7, 156.5, 142.8, 141.1, 135.5, 131.7, 124.8, 121.4, 115.9, 115.2, 71.3, 70.6, 70.5, 70.3, 70.1, 69.8, 45.1, 43.9, 39.6, 32.6, 32.5, 29.4, 26.7, 25.4 ppm. **HRMS (ESI)** m/z: $[M + Na]^+$ Calcd for $C_{28}H_{39}ClN_4O_7Na$ 601.2405; Found 604.2415.

***N*-(2-(2-((6-Chlorohexyl)oxy)ethoxy)ethyl)-2-(10-methyl-2,4-dioxo-4,10-dihydropyrimido[4,5-*b*]quinolin-3(2*H*)-yl)acetamide** (**15**): Prepared using chloroalkane-PEG$_2$-NHBoc (0.14 g, 0.43 mmol) to obtain a yellow powder (96.0 mg, 0.19 mmol, 59%).

^{1}H NMR (600 MHz, $CDCl_3$) δ = 8.91 (s, 1H), 7.94 – 7.87 (m, 2H), 7.71 (d, *J* = 8.7 Hz, 1H), 7.54 – 7.47 (m, 1H), 6.40 (t, *J* = 5.7 Hz, 1H), 4.74 (s, 2H), 4.17 (s, 3H), 3.63 (dd, *J* = 5.9, 3.4 Hz, 2H), 3.57 (dd, *J* = 5.5, 3.8 Hz, 4H), 3.52 (t, *J* = 6.7 Hz, 2H), 3.49 (t, *J* = 5.3 Hz, 2H), 3.46 (t, *J* = 6.7 Hz, 2H), 1.81 – 1.73 (m, 2H), 1.64 – 1.56 (m, 2H), 1.44 (ddt, *J* = 9.1, 7.2, 3.8 Hz, 2H), 1.40 – 1.33 (m, 2H) ppm. **^{13}C NMR** (151 MHz, $CDCl_3$) δ 167.0, 161.6, 156.5, 156.3, 143.0, 141.1, 135.7, 131.7, 124.9, 121.4, 116.0, 115.1, 71.3, 70.4, 70.1, 69.7, 45.1, 44.0, 39.5, 32.7, 32.5, 29.5, 26.7, 25.4 ppm. **HRMS (ESI)** m/z: $[M + Na]^+$ Calcd for $C_{24}H_{31}ClN_4O_5Na$ 513.1875; Found 513.1940.

^{1}H NMR of **13:**

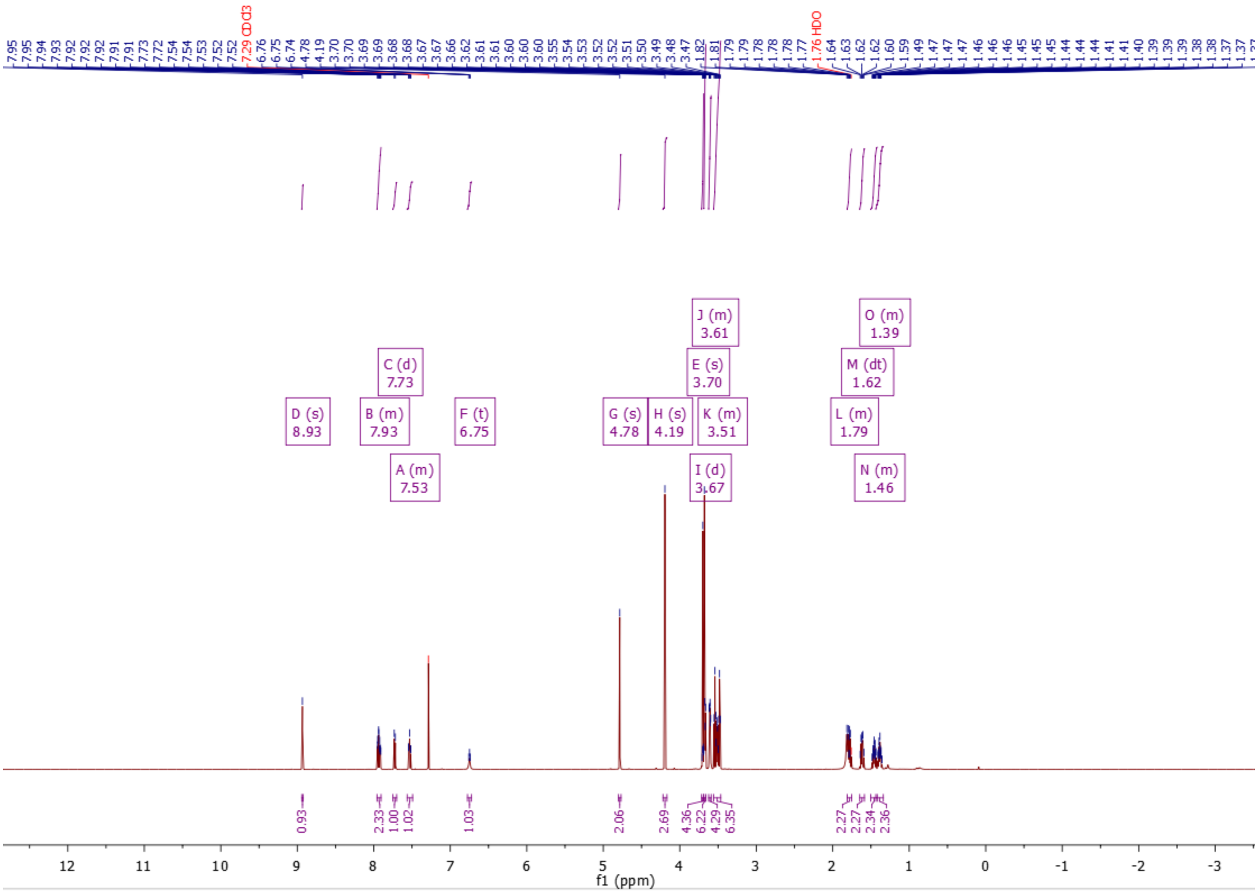

^{13}C NMR of **13:**

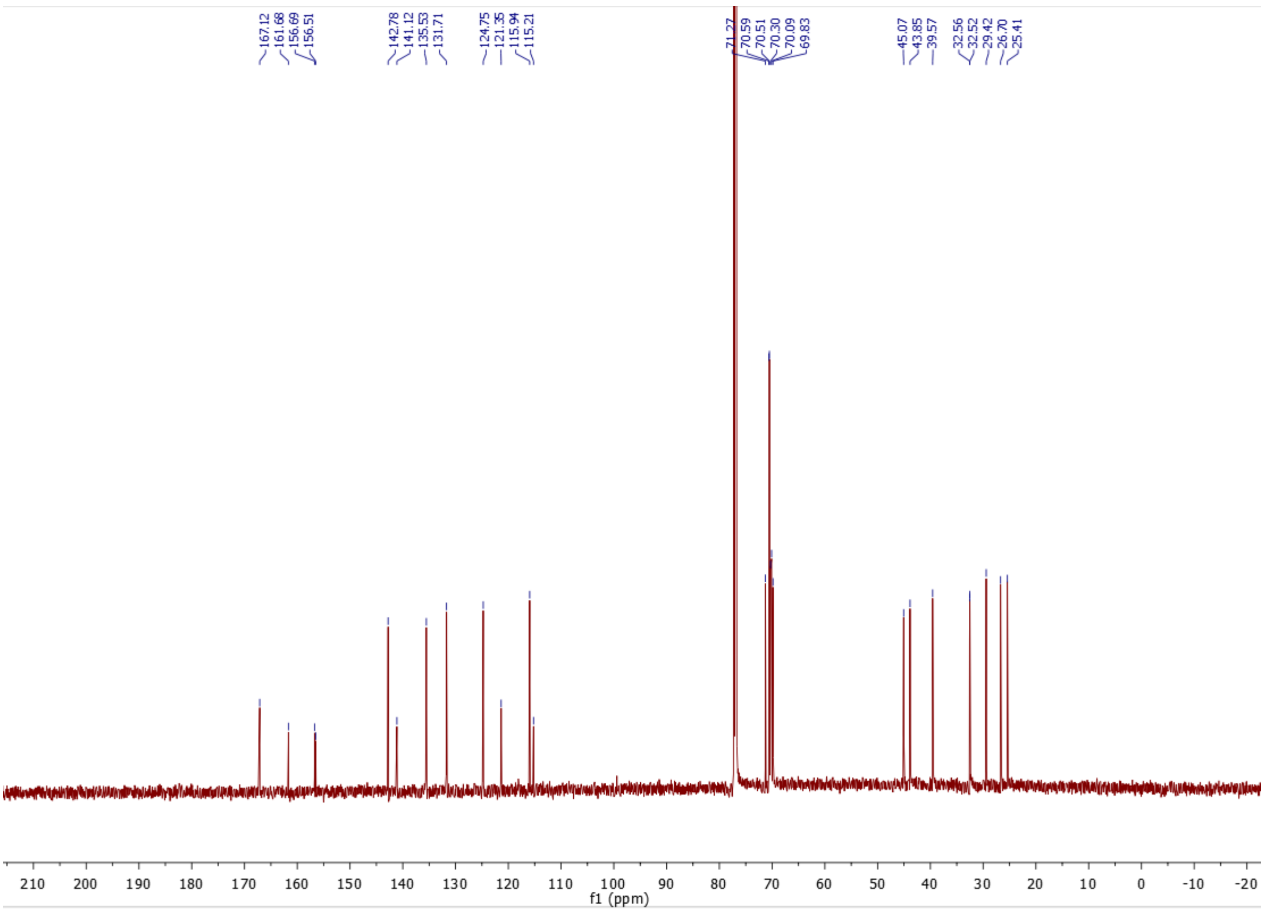

^{1}H NMR of **15:**

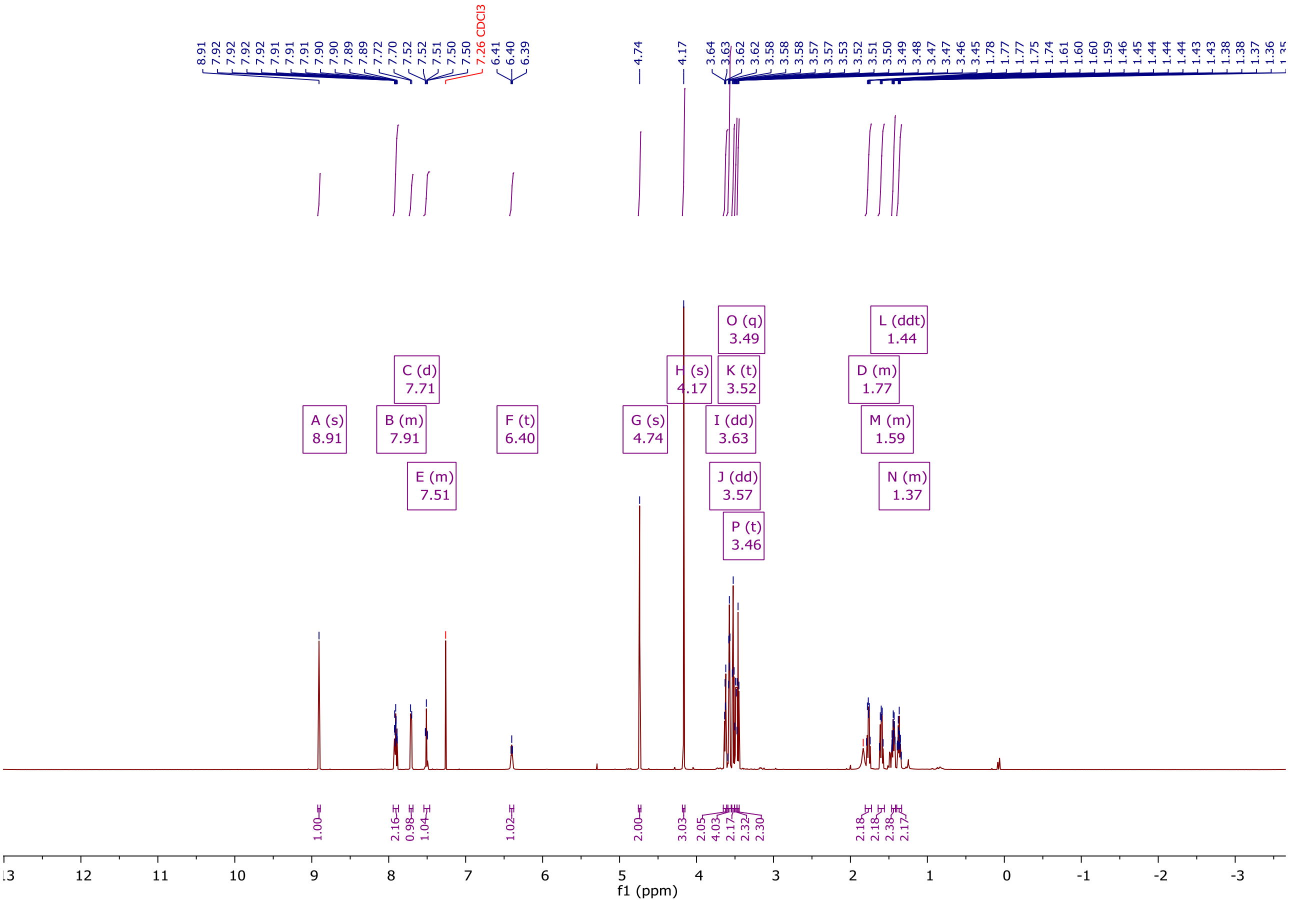

^{13}C NMR of **15:**

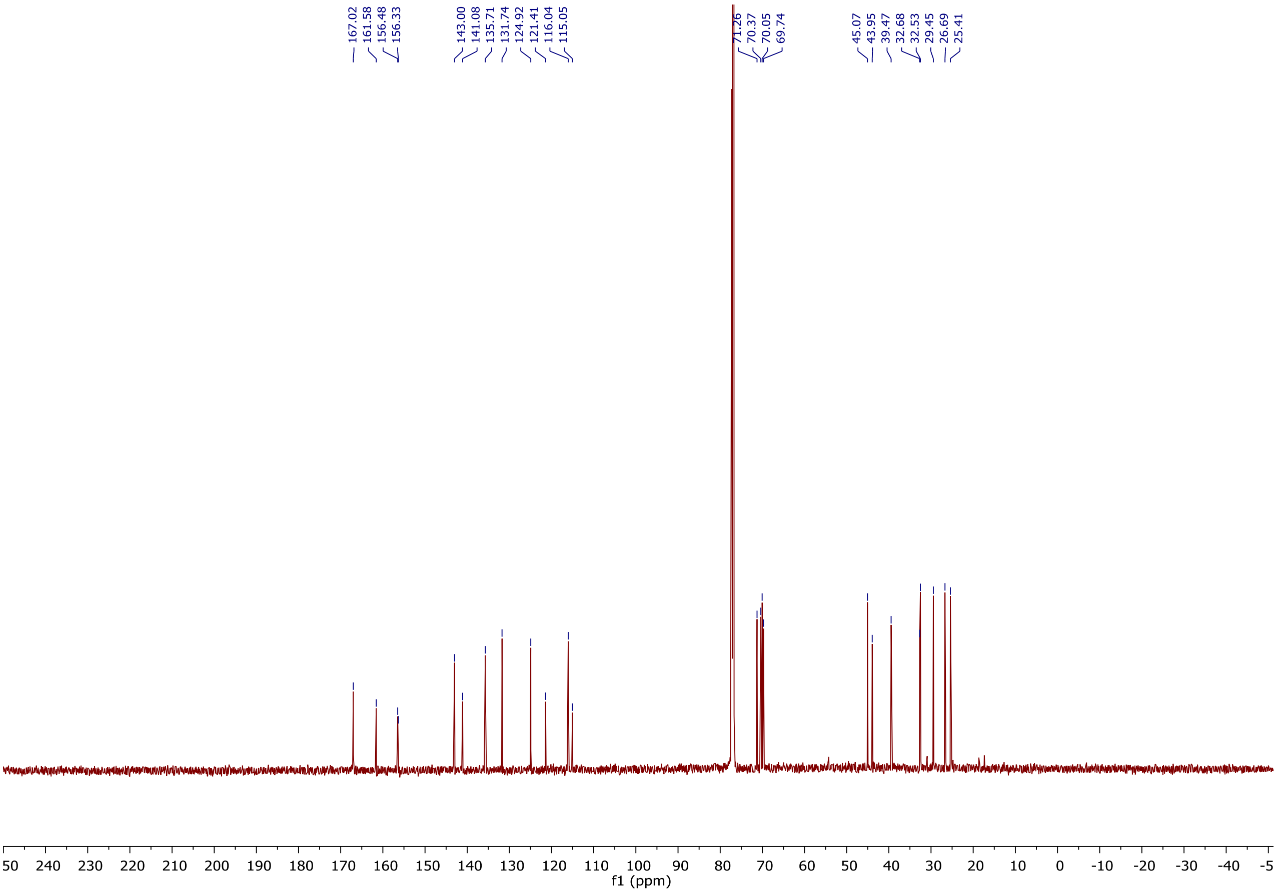

***N*-(2-(2-(2-methoxyethoxy)ethoxy)ethyl)-2-(10-methyl-2,4-dioxo-4,10-dihydropyrimido[4,5-*b*]quinolin-3(2*H*)-yl)acetamide** (**16**):

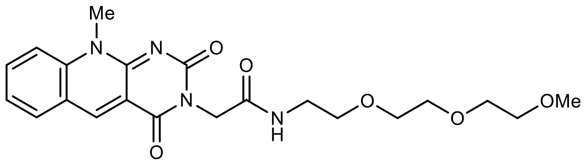

To a solution of deazaflavin **12** (80 mg, 0.28 mmol), HATU (144 mg, 0.28 mmol), DIPEA (0.19 mL, 0.60 mmol) in dry DMF (3 mL) was added mPEG$_2$-NH$_2$ (98 mg, 0.60 mmol). The solution was stirred at room temperature for 16 h under Ar atmosphere. The mixture was then diluted with EtOAc (100 mL) and washed with water (3 x 50 mL). The organic layer was then dried over Na_2SO_4, filtered and concentrated *in vacuo*. The residue was then purified by column chromatography (0-5% MeOH in DCM) to obtain a yellow powder (87 mg, 0.20 mmol, 72%).

^{1}H NMR (600 MHz, $CDCl_3$) δ = 8.91 (s, 1H), 7.94 – 7.87 (m, 2H), 7.72 (d, *J* = 8.7 Hz, 1H), 7.50 (t, *J* = 7.5 Hz, 1H), 4.73 (s, 2H), 4.15 (s, 3H), 3.65 (m, 6H), 3.59 – 3.55 (m, 4H), 3.47 (q, *J* = 5.1 Hz, 2H), 3.38 (s, 3H) ppm.

^{13}C NMR (151 MHz, $CDCl_3$) δ = 167.4, 167.3, 161.7, 156.5, 143.0, 141.1, 135.7, 131.7, 124.9, 121.4, 116.0, 115.1, 71.9, 70.5, 70.3, 70.2, 69.8, 59.0, 43.8, 39.5, 32.6 ppm.

HRMS (ESI) m/z: $[M + Na]^+$ Calcd for $C_{21}H_{26}N_4O_6Na$ 453.1745; Found 453.1793.

1H NMR of **16**:

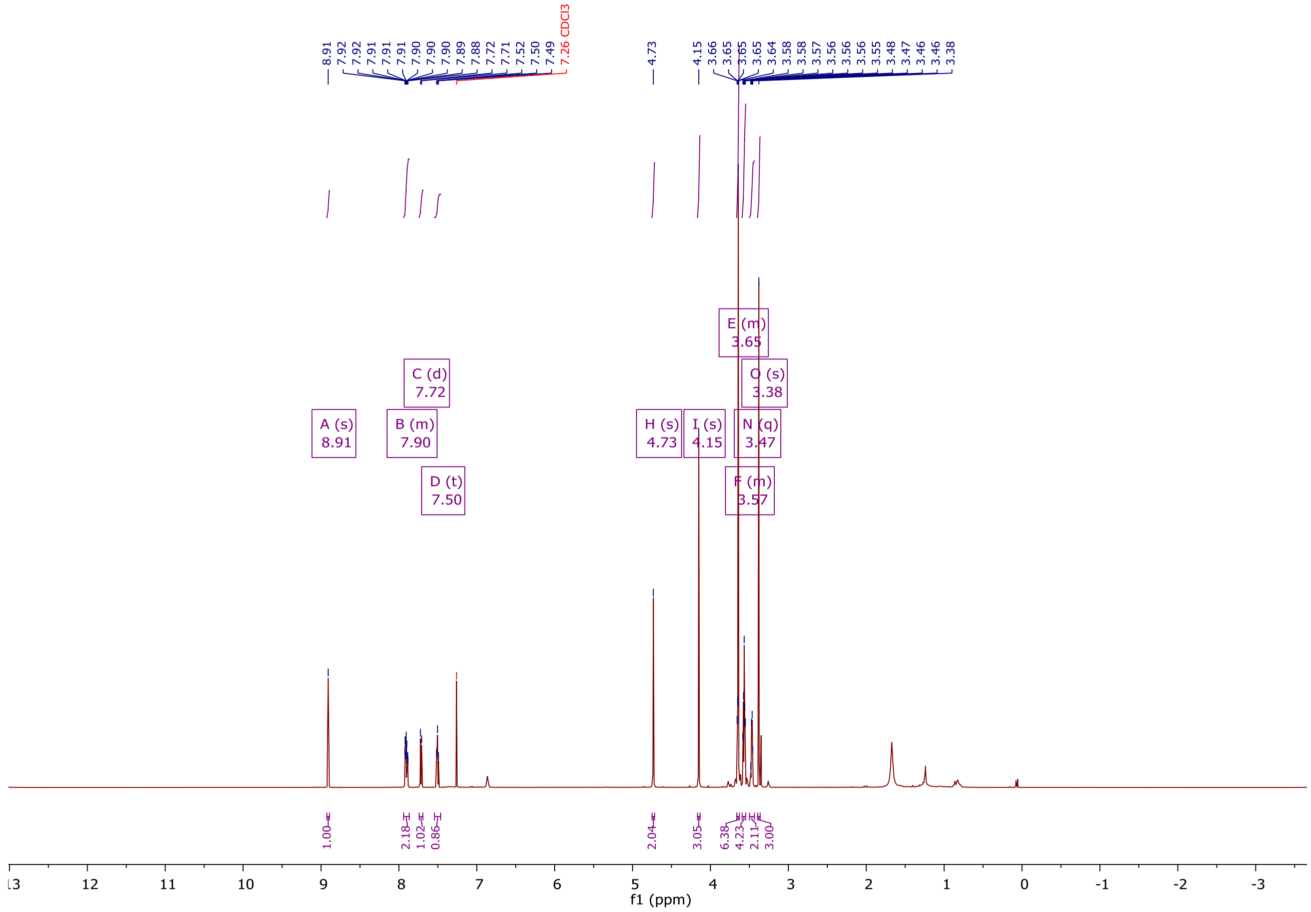

^{13}C NMR of **16:**

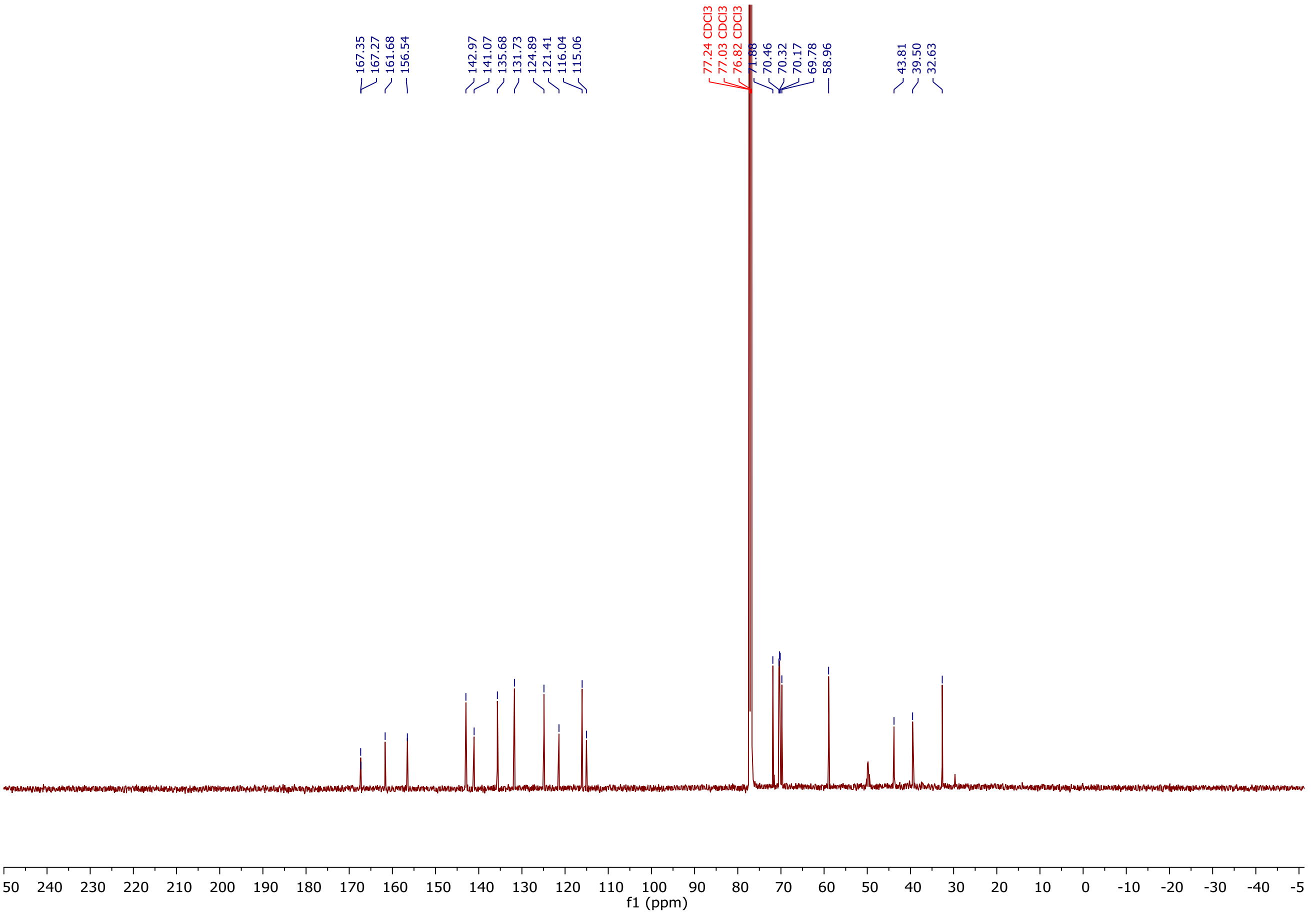

3-(hex-5-yn-1-yl)-10-methylpyrimido[4,5-b]quinoline-2,4(3H,10H)-dione (S6):

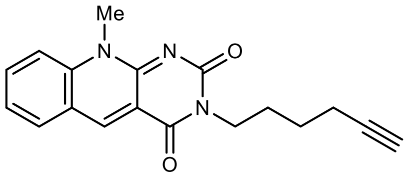

6-iodohex-1-yne (300 mg, 357 µL, 1.44 mmol) was added dropwise to a suspension of **4** (81.9 mg, 0.361 mmol) and dry Cs_2CO_3 (176 mg, 0.541 mmol) in dry DMF (2 mL) at 45 °C under Ar atomosphere. The resulting mixture was stirred at 45 °C for 16 h. The reaction mixture was then diluted with $CHCl_3$ (50 mL) and washed with water (3 x 25 mL). The organic phase was then dried over $MgSO_4$, filtered and concentrated *in vacuo*. The resulting yellow material was purified by silica gel column chromatography (1:2 EtOAc:DCM) to obtain the title compound as a yellow powder (58.2 mg, 190 µmol, 52.5 %).

^{1}H NMR (600 MHz, $CDCl_3$) δ = 8.92 (s, 1H), 7.94 (s, 1H), 7.90 (s, 1H), 7.71 (s, 1H), 7.51 (s, 1H), 4.15 (s, 3H), 4.08 (s, 2H), 2.24 (s, 2H), 1.93 (s, 1H), 1.81 (s, 2H), 1.61 (s, 2H) ppm.

^{13}C NMR (151 MHz, $CDCl_3$) δ = 166.0, 161.5, 160.3, 147.2, 145.0, 139.9, 135.7, 129.0, 125.5, 120.1, 118.7, 87.8, 72.4, 44.7, 36.3, 30.9, 29.8, 22.0 ppm.

HRMS (ESI) m/z: $[M + H]^+$ Calcd for $C_{18}H_{17}N_3O_2$ 308.1394; Found 308.1356.

^{1}H NMR of **S6:**

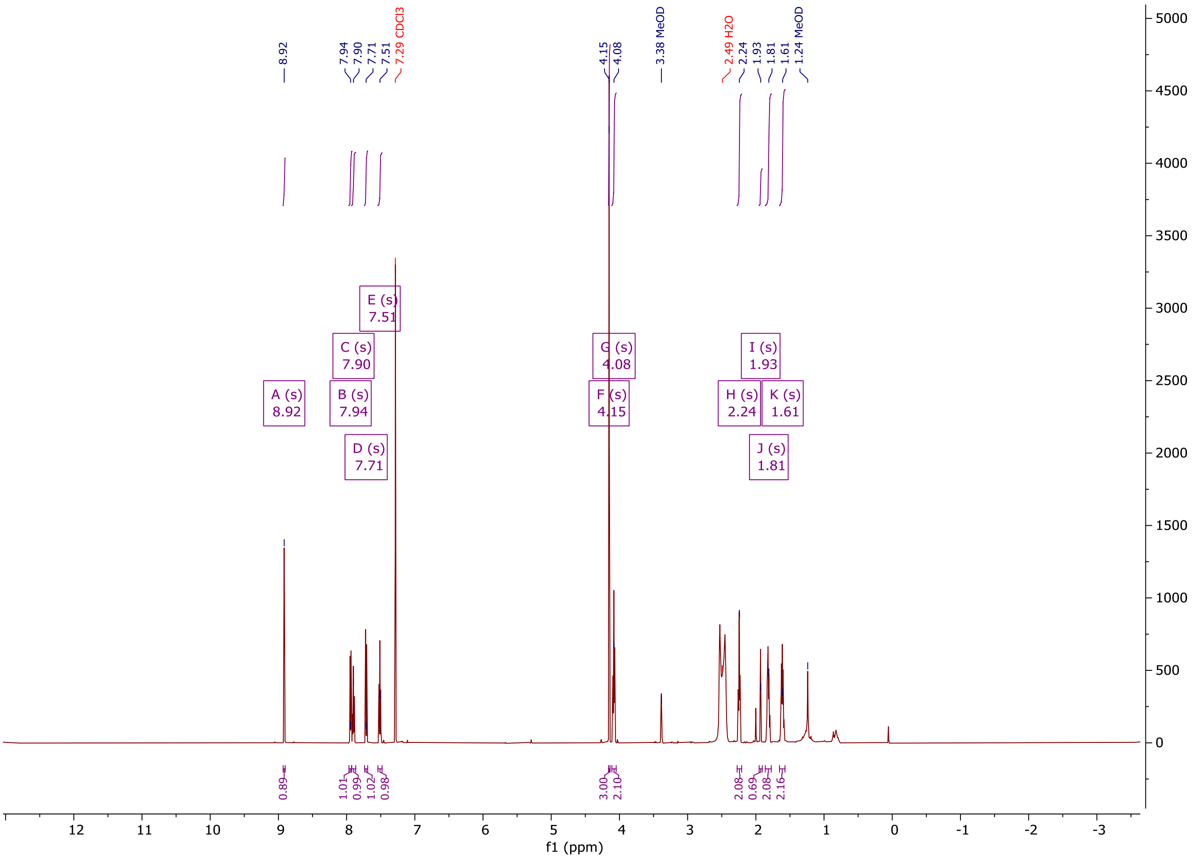

^{13}C NMR of **S6:**

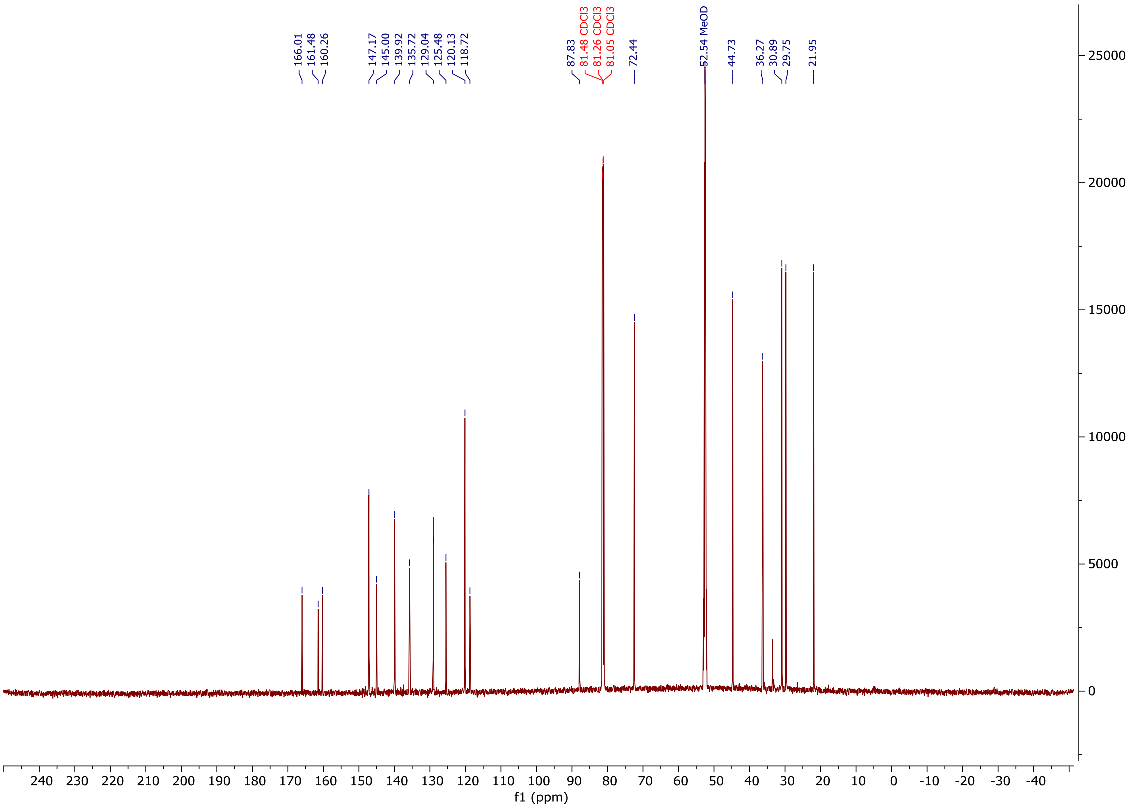

N-(20-azido-3,6,9,12,15,18-hexaoxaicosyl)-2-(10-methyl-2,4-dioxo-4,10-dihydropyrimido[4,5-b]quinolin-3(2H)-yl)acetamide (S7)

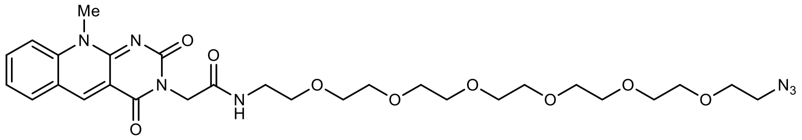

To a solution of **12** (20 mg, 70 µmol), HATU (29.2 mg, 77 µmol), DIPEA (24 µL, 0.14 mmol) in DMSO (0.5 mL) was added a solution of H_2N-PEG(6)-N_3 (36.8 mg, 0.105 mmol) in 0.2 mL DMSO. The solution was stirred at room temperature for 30 min, following purification by preparative HPLC. After lyophilisation, the product was obtained as a yellow powder (21.5 mg, 35 µmol, 50 %).

^{1}H NMR (600 MHz, DMSO-d6) δ = 9.06 (s, 1H), 8.23 (dd, *J* = 7.9, 1.4 Hz, 1H), 8.12 (t, *J* = 5.7 Hz, 1H), 8.03 – 7.96 (m, 2H), 7.58 (ddd, *J* = 8.0, 6.3, 1.7 Hz, 1H), 4.49 (s, 2H), 4.09 (s, 3H), 3.62 – 3.55 (m, 2H), 3.57 – 3.48 (m, 20H), 3.41 – 3.36 (m, 2H), 3.22 (q, *J* = 5.8 Hz, 2H) ppm.

^{13}C NMR (151 MHz, DMSO-d6) δ = 167.6, 161.8, 156.5, 155.9, 142.8, 141.2, 136.1, 132.2, 125.0, 121.5, 117.3, 115.0, 70.3, 70.2, 70.2, 70.2, 70.1, 69.7, 69.5, 50.5, 43.5, 32.6 ppm.

HRMS (ESI) m/z: $[M + H]^+$ Calcd for $C_{28}H_{39}N_7O_9$ 618.2887; Found 618.2876

^{1}H NMR of **S7:**

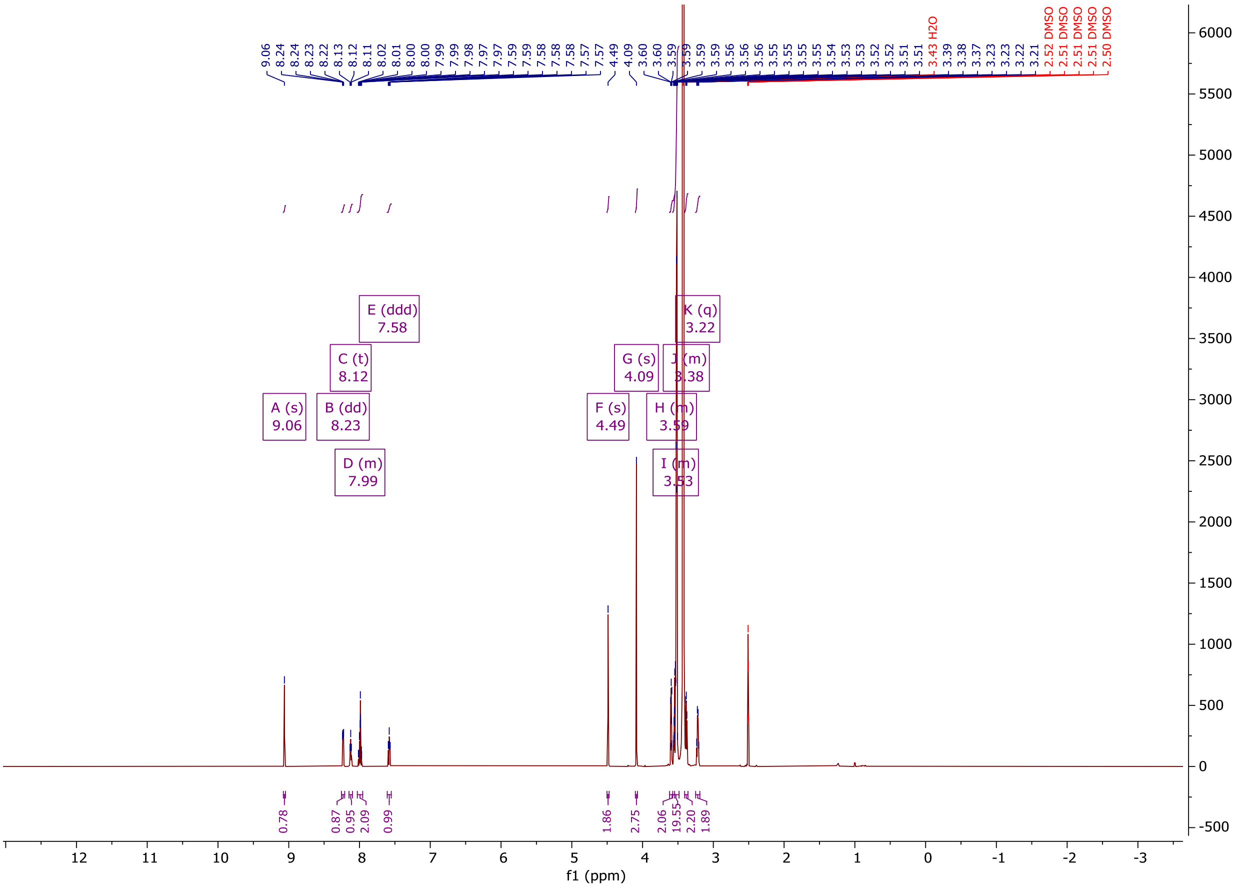

^{13}C NMR of **S7:**

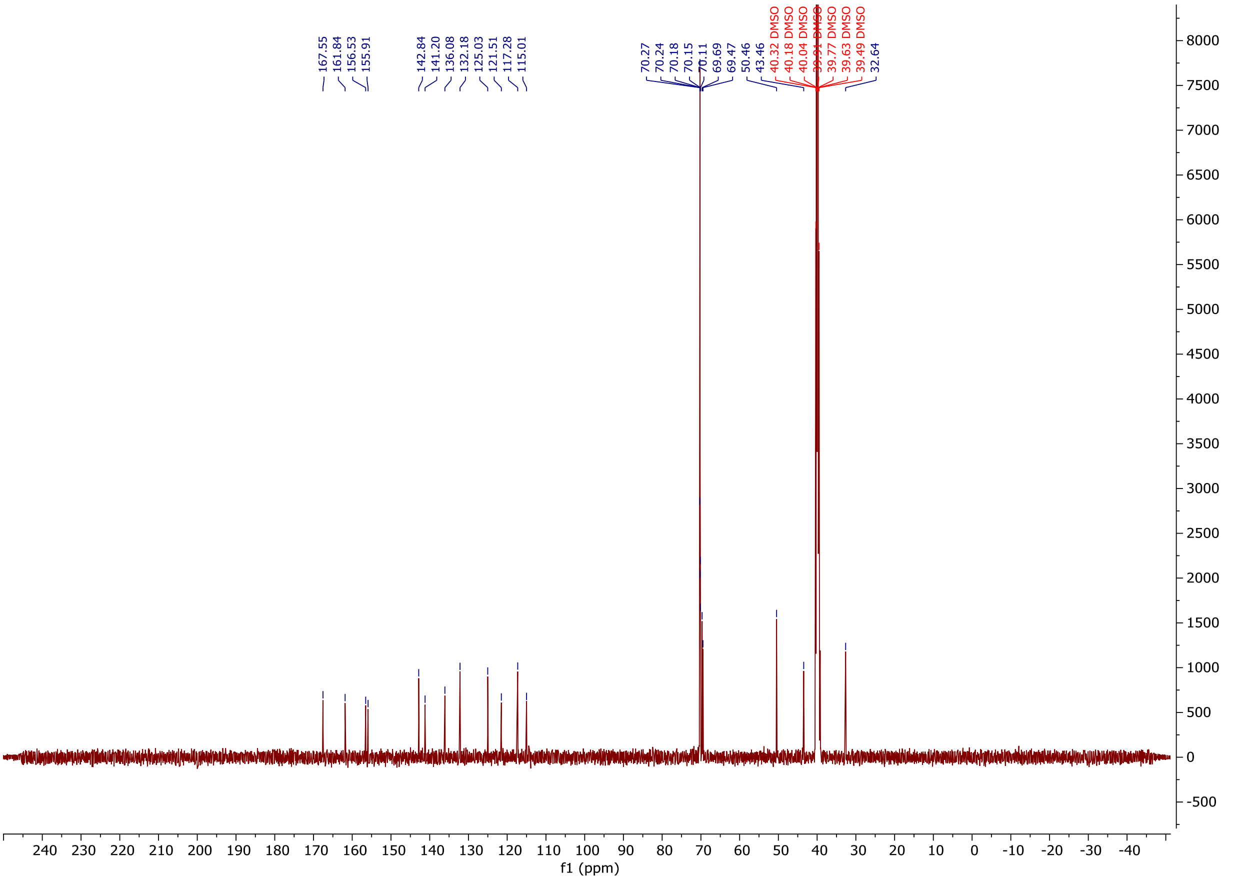

Ethyl ethynyl(1-(1-(10-methyl-2,4-dioxo-4,10-dihydropyrimido[4,5-b]quinolin-3(2H)-yl)-2-oxo-6,9,12,15,18,21-hexaoxa-3-azatricosan-23-yl)-1H-1,2,3-triazol-4-yl)phosphinate (S8):

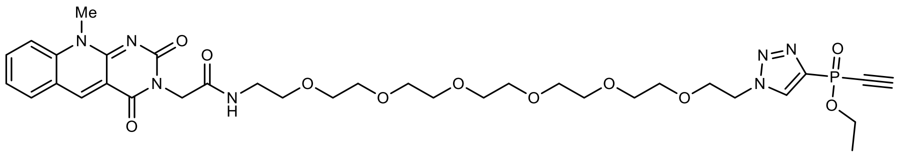

The synthesis of deazaflavin-Ethynyl-Triazolyl-Phosphinate (ETP, **S7**) was conducted as previously described.[19] Deazaflavin **S6** (9 mg, 14.6 µmol) was dissolved in 200 µL of 0.2 M phosphate buffer (pH=6). 100 µL acetonitrile were added to solubilise the compound. Ethyl diethynyl phosphinate[20] (20 mg, 100 µmol) was added subsequently. The click reaction was initiated by adding 10 µL of a saturated CuBr solution premixed with 30 µL of 100 mM Tris(3-hydroxypropyltriazolylmethyl)amine (THPTA) to the reactants. After 30 minutes, the reaction was quenched by the addition of 3 mL 0.1 % TFA in water and the product was purified via preparative HPLC (8.3 mg, 10.92 µmol, 75 %).

^{1}H NMR (600 MHz, DMSO-d6) δ = 9.07 (s, 1H), 8.67 (s, 1H), 8.27 – 8.21 (m, 1H), 8.12 (t, *J* = 5.7 Hz, 1H), 8.00 (dd, *J* = 6.3, 1.6 Hz, 1H), 7.58 (ddd, *J* = 8.0, 6.0, 2.0 Hz, 1H), 4.71 (dd, *J* = 11.6, 2.3 Hz, 1H), 4.63 (t, *J* = 5.1 Hz, 3H), 4.17 (dqd, *J* = 9.4, 7.0, 2.6 Hz, 2H), 4.09 (s, 2H), 3.86 (t, *J* = 5.3 Hz, 2H), 3.22 (q, *J* = 5.9 Hz, 2H), 1.31 (td, *J* = 7.0, 3.1 Hz, 3H) ppm.

^{13}C NMR (151 MHz, DMSO-d6) δ = 167.5, 161.8, 156.5, 155.9, 142.9, 141.2, 139.8, 138.3, 136.1, 132.5, 132.3, 132.2, 125.0, 121.5, 117.3, 115.0, 95.0, 77.0, 70.3, 70.2, 70.2, 70.2, 70.1, 70.1, 70.0, 69.5, 69.4, 68.7, 63.1, 50.1, 43.5, 32.7, 16.5 ppm.

^{31}P-NMR (243 MHz, DMSO-d6) δ = -4.77 ppm.

HRMS (ESI) m/z: $[M + H]^{+}$ Calcd for $C_{34}H_{46}N_{7}O_{11}$ 760.3071; Found 760.2972

^{1}H NMR of **S8:**

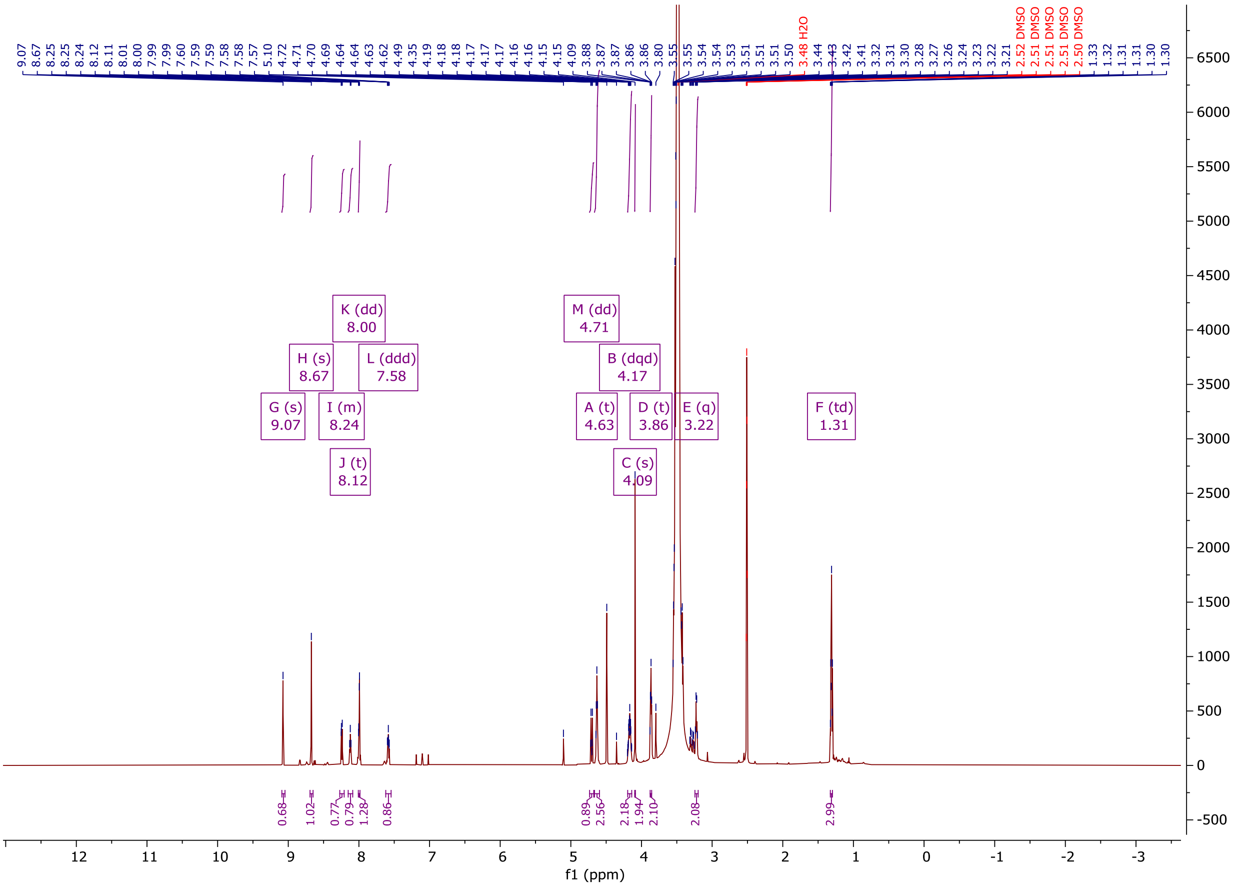

^{13}C NMR of **S8:**

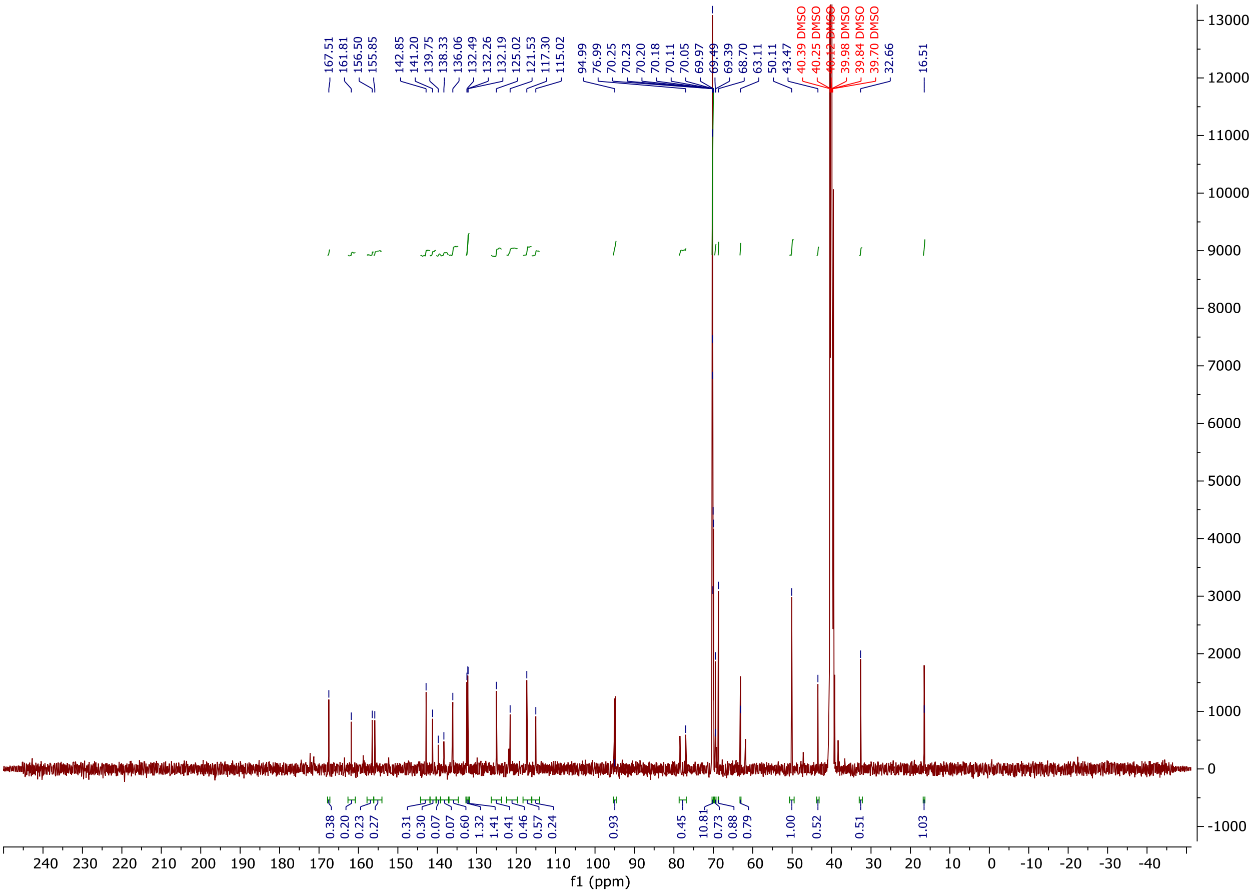

^{31}P NMR of **S8:**

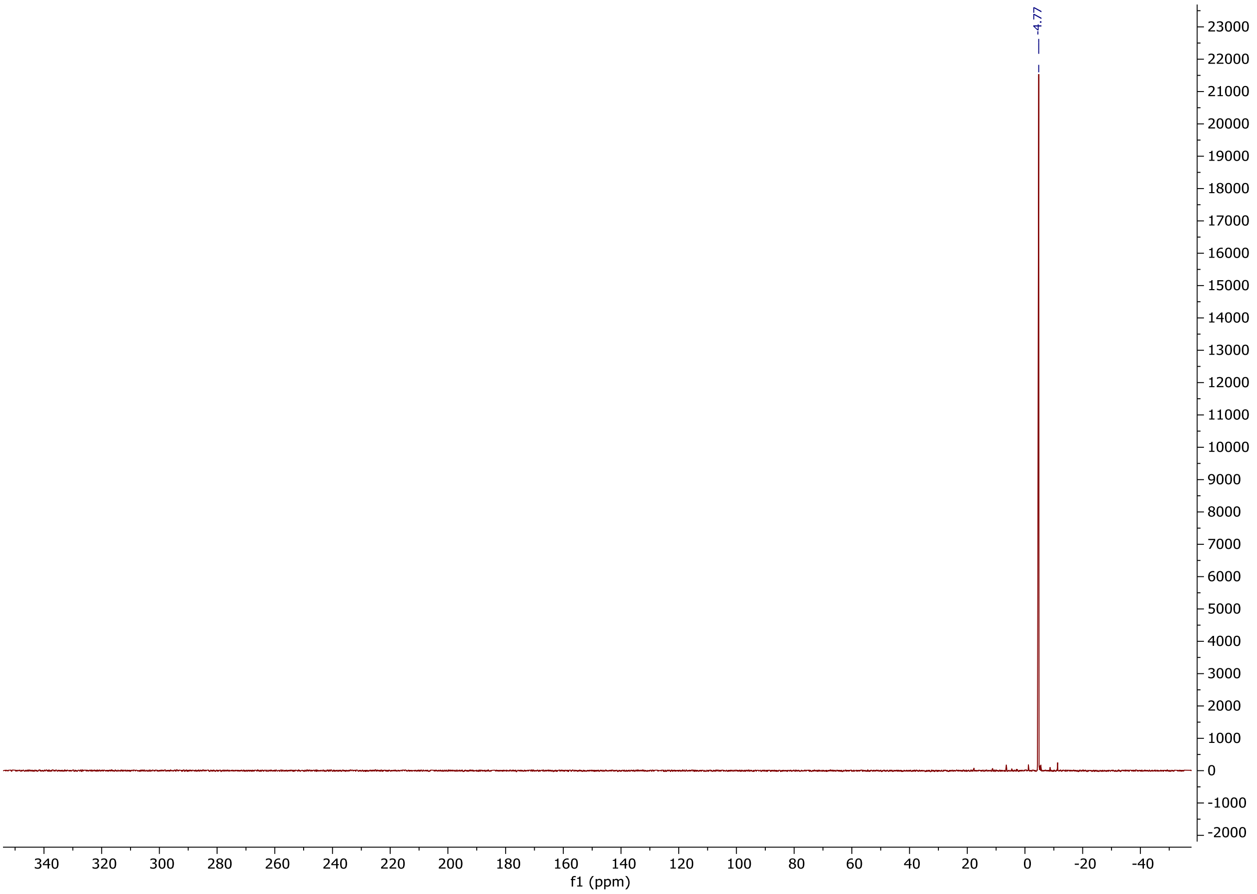

Synthesis of Ir-G3-PEG$_4$-chloroalkane (14)

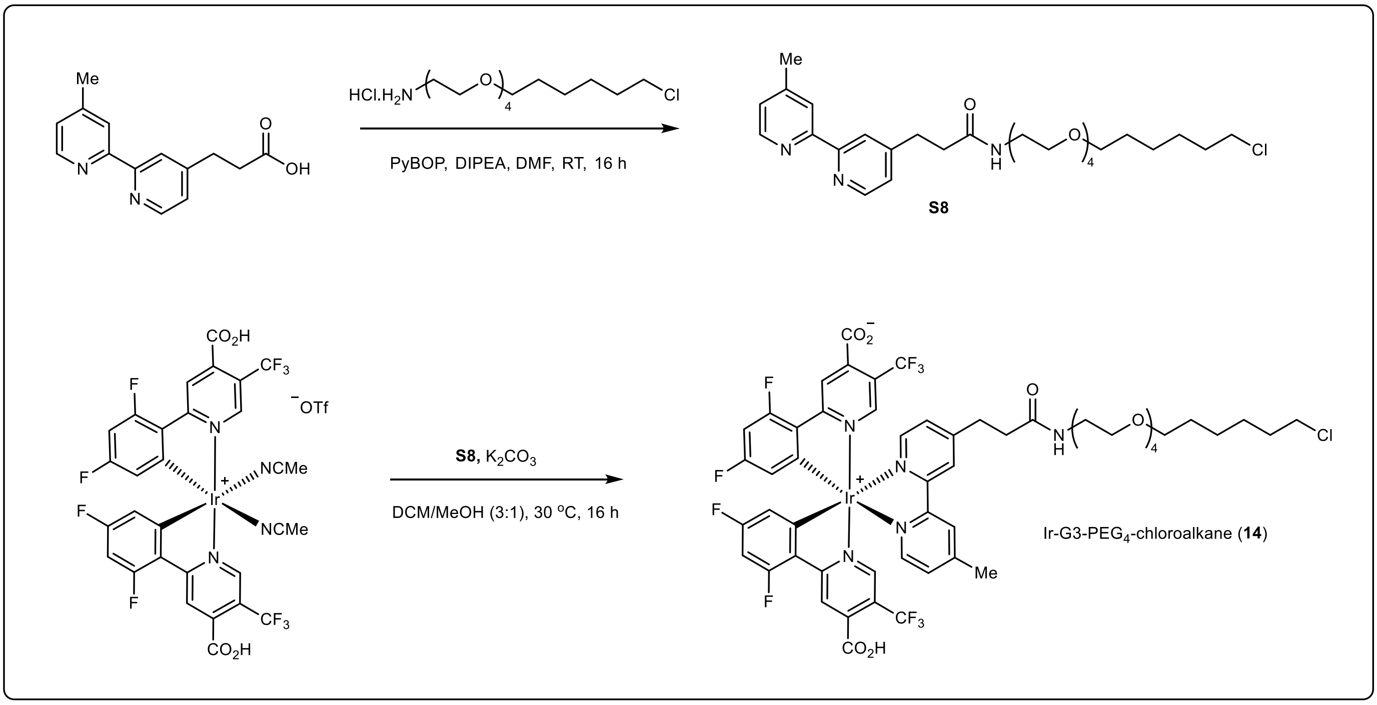

Scheme S3: Synthesis of Ir-G3-PEG$_4$-chloroalkane (**14**). The synthesis was achieved by adapting previously reported syntheses of Ir-G3 catalysts.[21]

***N*-(18-chloro-3,6,9,12-tetraoxaoctadecyl)-3-(4'-methyl-[2,2'-bipyridin]-4-yl)propanamide (S8):**

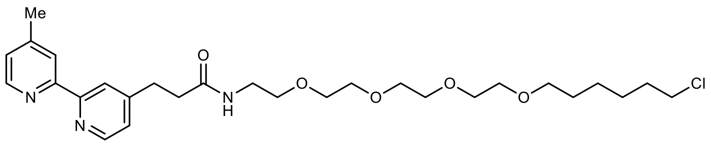

Chloroalkane-PEG$_4$-NH$_3$Cl (72.5 mg, 208.0 µmol) dissolved in dry DMF (0.5 mL) was added to a solution of 3-(4'-methyl-[2,2'-bipyridin]-4-yl)propanoic acid (42.0 mg, 173.4 µmol),[15] PyBOP (108.3 mg, 208.0 µmol) and DIPEA (90 µL, 520.0 µmol) in dry DMF (0.5 mL) under Ar atmosphere. The reaction mixture was stirred for 16 h at room temperature and subsequently diluted with EtOAc (50 mL) and washed with water (25 mL), sat. NaHCO$_3$ (25 mL) and brine (25 mL). The organic layer was then dried over Na$_2$SO$_4$ and concentrated under reduced pressure to provide a yellow oil that was purified by silica gel column chromatography (0-10% MeOH in DCM) to provide the desired compound as a colourless oil (87.6 mg, 163.4 µmol, 94%).

^{1}H NMR (600 MHz, CD$_3$CN) δ = 8.58 (dd, *J* = 7.2, 5.1 Hz, 2H), 8.33 – 8.28 (m, 2H), 7.35 (dt, *J* = 4.1, 1.9 Hz, 2H), 6.68 (s, 1H), 3.59 (t, *J* = 6.7 Hz, 2H), 3.55 (q, *J* = 3.4, 2.5 Hz, 6H), 3.52 – 3.49 (m, 4H), 3.49 – 3.47 (m, 2H), 3.41 (dt, *J* = 10.8, 6.1 Hz, 4H), 3.29 (q, *J* = 5.6 Hz, 2H), 3.04 (t, *J* = 7.4 Hz, 2H), 2.57 (t, *J* = 7.4 Hz, 2H), 2.50 (s, 3H), 1.75 (dt, *J* = 14.7, 6.9 Hz, 2H), 1.53 (dt, *J* = 14.6, 6.7 Hz, 2H), 1.47 – 1.38 (m, 2H), 1.38 – 1.30 (m, 2H) ppm.

^{13}C NMR (151 MHz, CD$_3$CN) δ = 171.3, 153.8, 153.8, 153.0, 150.4, 148.5, 147.9, 125.4, 124.7, 122.2, 121.4, 70.6, 70.2, 70.1, 70.1, 70.0, 69.9, 69.8, 69.4, 45.2, 38.9, 35.8, 32.3, 30.8, 29.3, 26.4, 25.2, 20.5 ppm.

HRMS (ESI) m/z: $[M + Na]^+$ Calcd for $C_{28}H_{42}ClN_3O_5Na$ 558.2706; Found 588.2755

^{1}H NMR of **S8:**

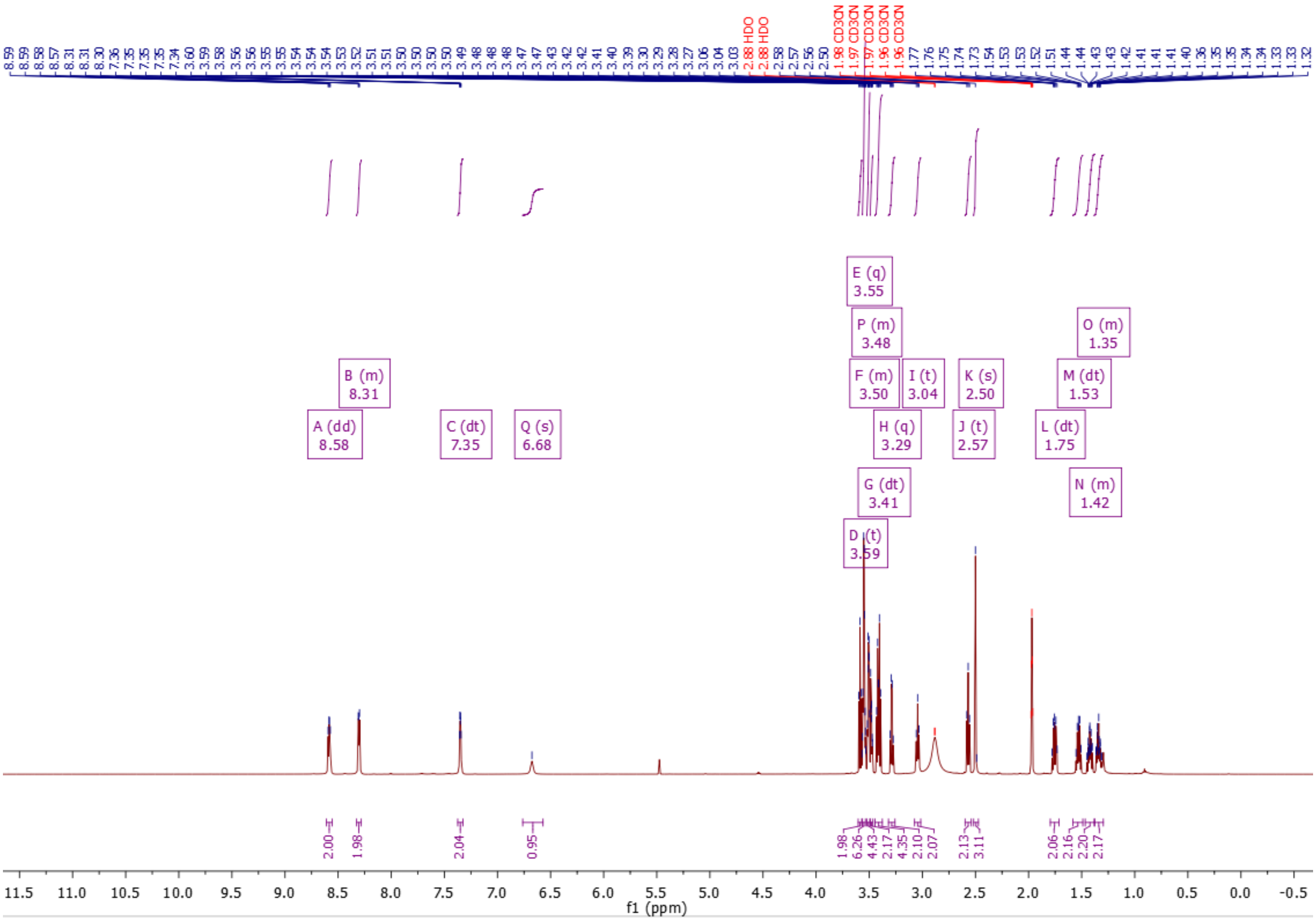

^{13}C NMR of **S8:**

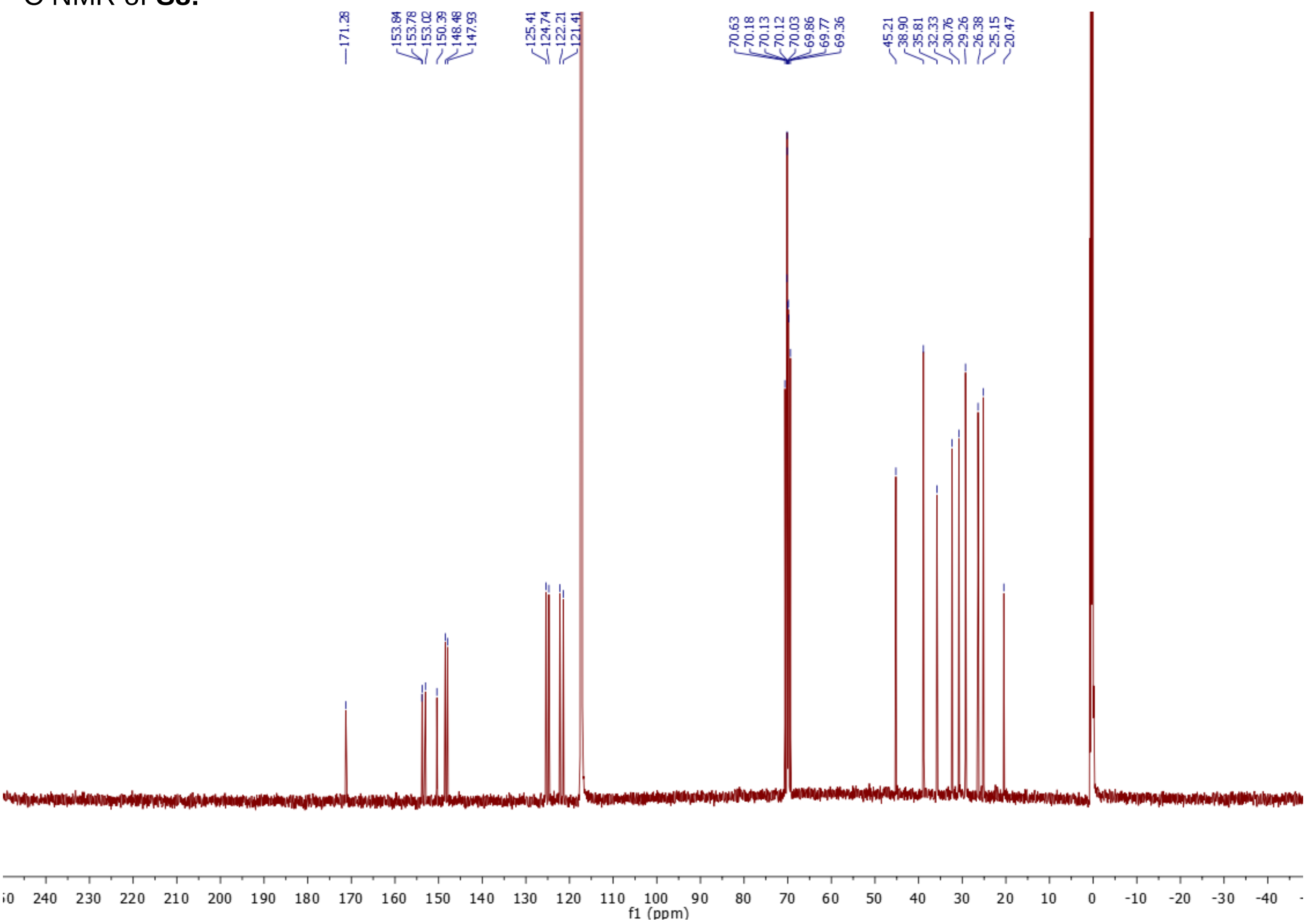

Ir-G3-PEG$_4$-chloroalkane (14):

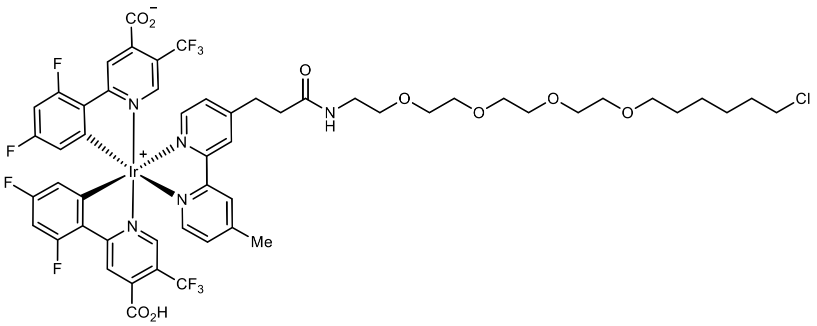

[Ir(dCO$_2$HdFCF3ppy)$_2$(MeCN)$_2$]OTf (60 mg, 58.4 µmol)[13] and K_2CO_3 (17.8 mg, 128.4 µmol) were suspended in DCM/MeOH (4 mL mL, 3:1) and compound **S8** (68.9 mg, 128.4 µmol) dissolved in DCM/MeOH (1 mL, 3:1) was added dropwise under Ar atmosphere**.** The resultant mixture was stirred at 30 °C for 16 hours before being concentrated under reduced pressure directly onto silica gel. The crude product was purified by flash silica gel column chromatography (0-30% MeOH in DCM) to provide the desired compound as a yellow solid (51.4 mg, 66% yield).

^{1}H NMR (600 MHz, MeOD) δ = 8.72 (s, 1H), 8.40 (dd, *J* = 6.4, 2.6 Hz, 2H), 7.98 (d, *J* = 5.7 Hz, 1H), 7.93 (d, *J* = 5.6 Hz, 1H), 7.73 – 7.50 (m, 4H), 6.80 – 6.73 (m, 2H), 5.86 (ddd, *J* = 12.9, 8.2, 2.3 Hz, 2H), 3.63 (s, 4H), 3.63 – 3.52 (m, 9H), 3.51 – 3.43 (m, 4H), 3.22 (td, *J* = 7.4, 1.9 Hz, 2H), 2.71 (t, *J* = 7.5 Hz, 2H), 2.67 (s, 3H), 1.75 (dt, *J* = 14.5, 6.8 Hz, 2H), 1.60 – 1.52 (m, 2H), 1.49 – 1.41 (m, 2H), 1.42 – 1.35 (m, 2H) ppm.

^{13}C NMR (151 MHz, MeOD) δ = 172.3, 169.4, 167.5, 164.77 (dd, *J* = 259.8, 12.5 Hz), 162.52 (dd, *J* = 262.7, 12.9 Hz), 156.2, 155.4, 155.2, 155.0 – 154.8 (m), 153.6, 151.0, 150.3, 149.9, 145.8 – 145.5 (m), 129.6, 128.8, 126.5, 125.9, 125.3, 122.8, 121.06 (d, J = 12.3 Hz), 113.79 (t, J = 15.7 Hz), 99.08 (t, J = 27.0 Hz), 70.7, 70.2, 70.2, 69.7, 69.1, 44.3, 38.9, 34.8, 32.3, 30.6, 29.1, 26.3, 25.1, 20.1 ppm.

^{19}F NMR (564 MHz, MeOD) δ -61.8 (s, 3F), -61.9 (s, 3F), -104.65 – -104.97 (m, 2F), -108.37 (q, *J* = 10.7 Hz, 2F).

HRMS (ESI) m/z: $[M + H]^+$ Calcd for $IrC_{54}H_{53}F_{10}N_5O_9Cl$ 1333.2996; Found 1333.2977

^{1}H NMR of **14:**

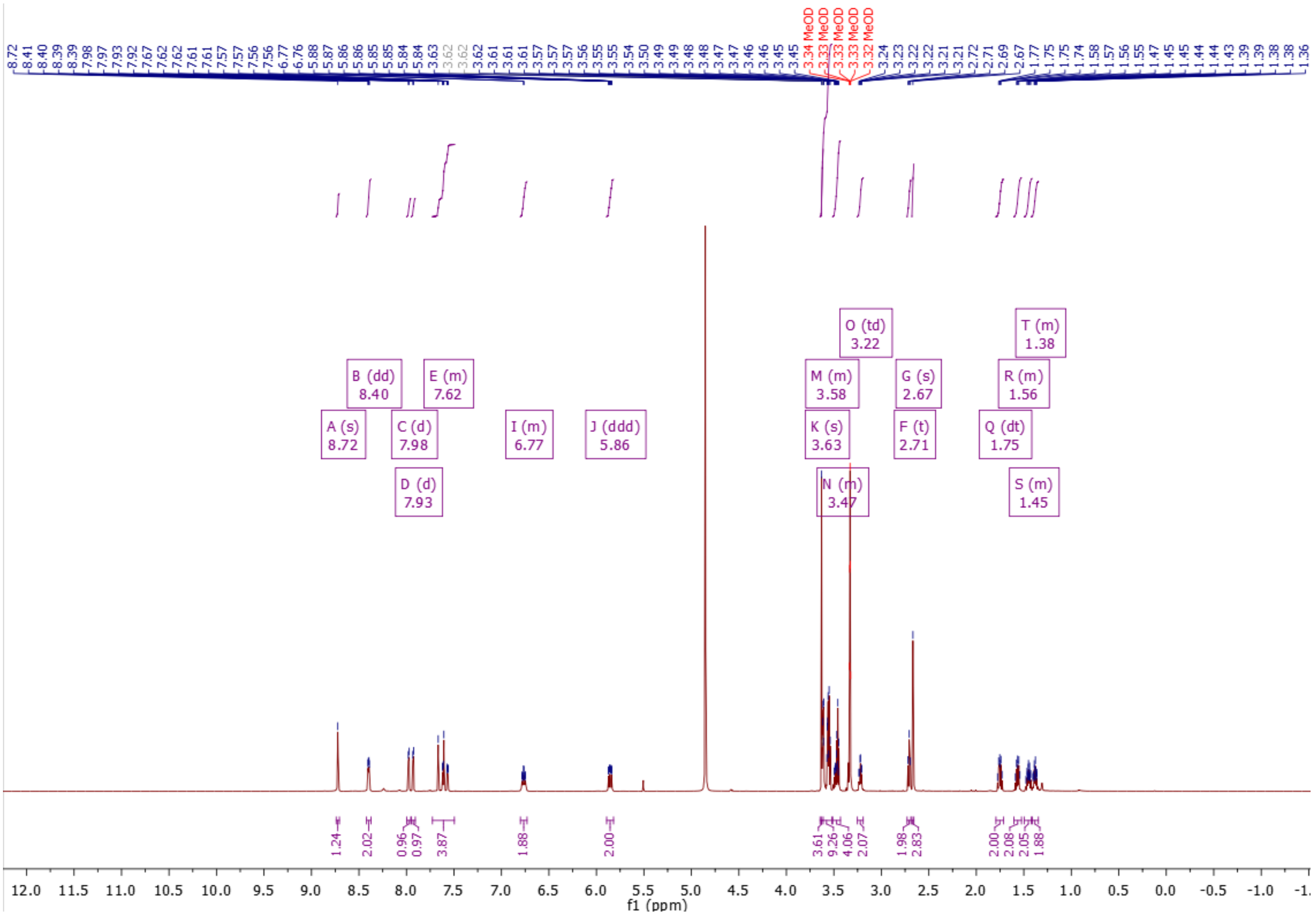

^{13}C NMR of **14:**

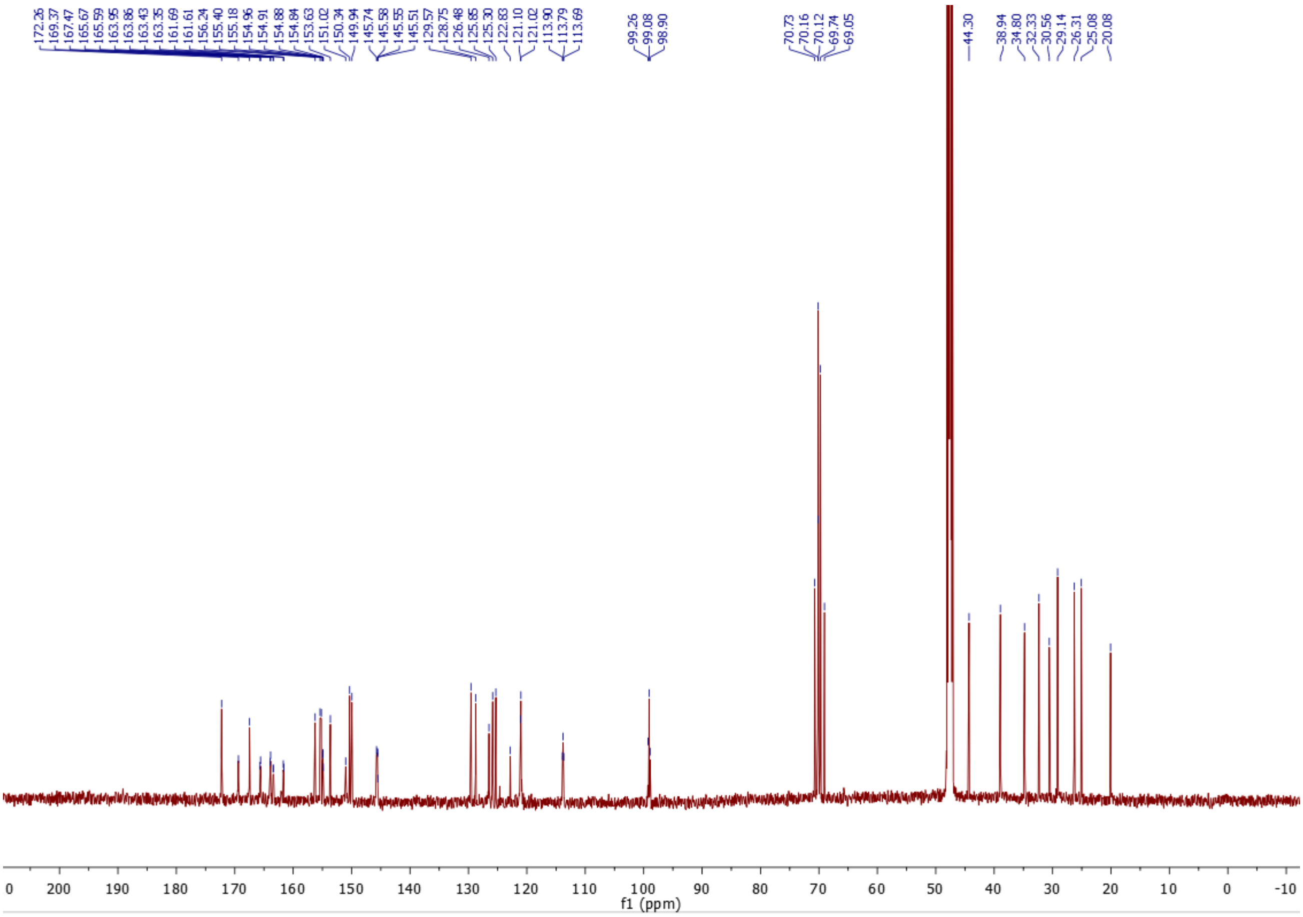

^{19}F NMR of **14:**

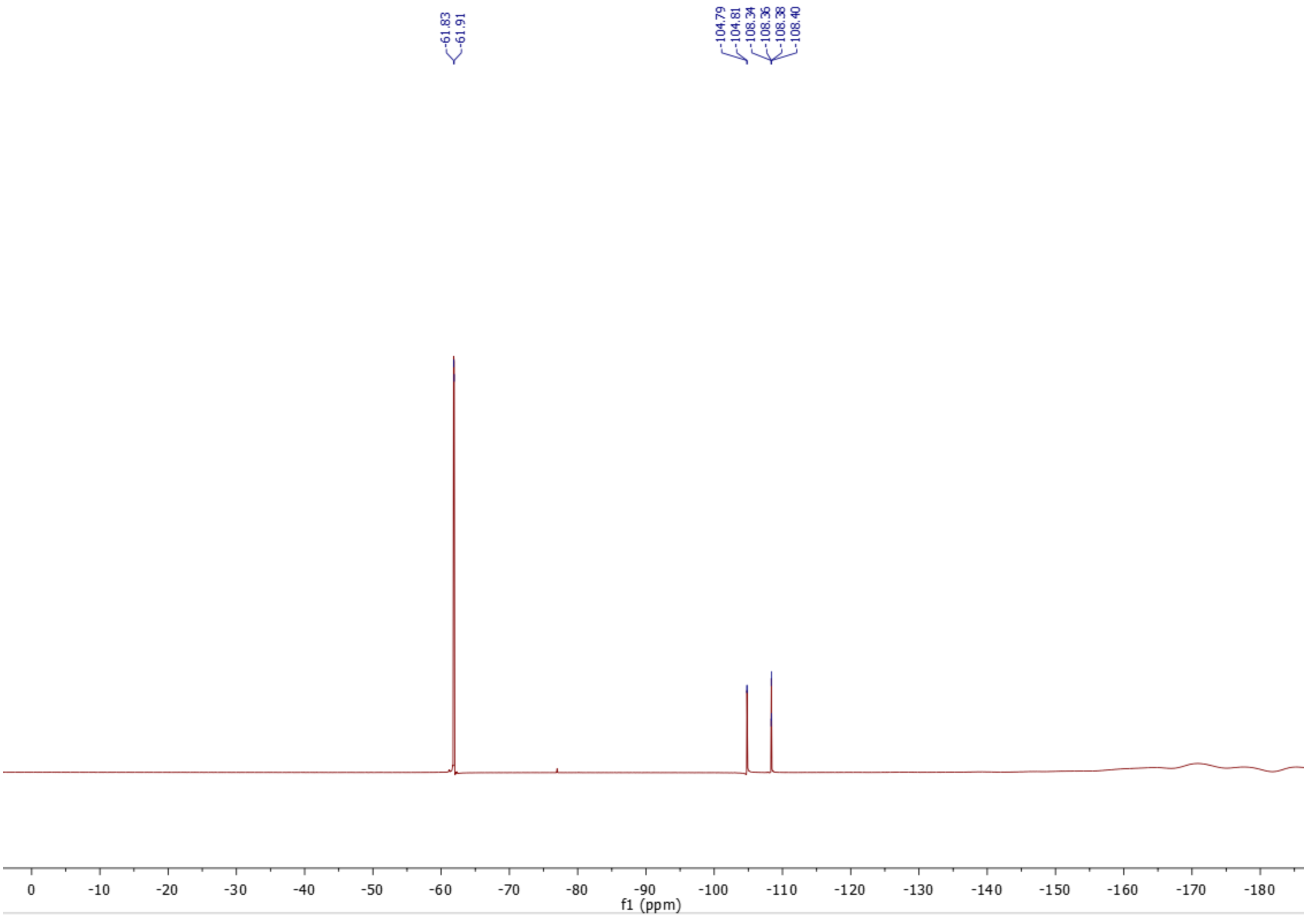

-61.83
-61.91
-104.79
-104.81
-108.34
-108.36
-108.38
-108.40
0 -10 -20 -30 -40 -50 -60 -70 -80 -90 -100 -110 -120 -130 -140 -150 -160 -170 -180
f1 (ppm)

3.1.2 Biotin and photoaffinity probes

Biotin-Phenol (**9**) and Biotin-PEG_3-phenyl azide (**10**) were purchased from Iris Biotech (DE).

Diazirine-PEG_4-biotin (8):

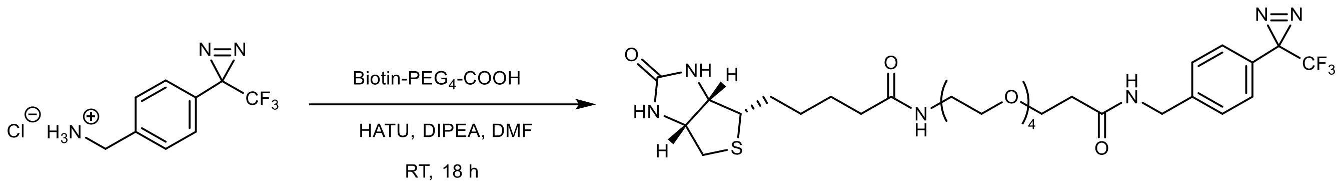

A solution of 4-[3-(trifluoromethyl)-3*H*-diazirin-3-yl]benzylamine hydrochloride (50 mg, 0.20 mmol) in DMF (1 mL) was added dropwise to a solution of Biotin-PEG_4-COOH (103 mg, 0.21 mmol), HATU (84 mg, 0.22 mmol) and DIPEA (0.11 mL, 0.64 mmol) in DMF (2 mL). The mixture was stirred at room temperature for 18 h under Ar atmosphere. The mixture was diluted with DCM (100 mL) and washed with water (50 mL), brine (50 mL), dried over Na_2SO_4, filtered, and concentrated *in vacuo*. The residue was then purified by silica gel column chromatography (2-10% MeOH in DCM) to obtain an off-white solid after drying under high vacuum (<0.1 mbar) (121 mg, 0.175 mmol, 88%).

^{1}H NMR (600 MHz, $CDCl_3$) δ = 7.32 (d, *J* = 8.2 Hz, 3H), 7.13 (d, *J* = 8.1 Hz, 2H), 6.84 (s, 1H), 4.49 – 4.45 (m, 1H), 4.43 (d, *J* = 5.9 Hz, 2H), 4.27 (dd, *J* = 7.6, 4.6 Hz, 1H), 3.75 (t, *J* = 5.8 Hz, 2H), 3.64 – 3.59 (m, 3H), 3.59 – 3.50 (m, 12H), 3.39 (s, 2H), 3.11 (q, *J* = 7.2 Hz, 1H), 2.87 (dd, *J* = 12.8, 4.9 Hz, 1H), 2.72 (d, *J* = 12.8 Hz, 1H), 2.52 (t, *J* = 5.8 Hz, 2H), 2.20 (t, *J* = 7.5 Hz, 2H), 1.74 – 1.61 (m, 4H), 1.47 – 1.36 (m, 3H) ppm.

^{13}C NMR (151 MHz, $CDCl_3$) δ = 173.5, 171.7, 164.1, 140.9, 127.9, 127.8, 126.6, 122.1 (q, *J* = 274.7 Hz), 70.4, 70.3, 70.2, 70.0, 67.3, 61.8, 60.2, 55.6, 42.6, 40.5, 39.2, 36.9, 35.9, 28.2, 28.1, 25.6 ppm.

HRMS (ESI) m/z: $[M + H]^+$ Calcd for $C_{30}H_{44}F_3N_6O_7S$ 689.2939; Found 689.2952.

^{1}H NMR of **8:**

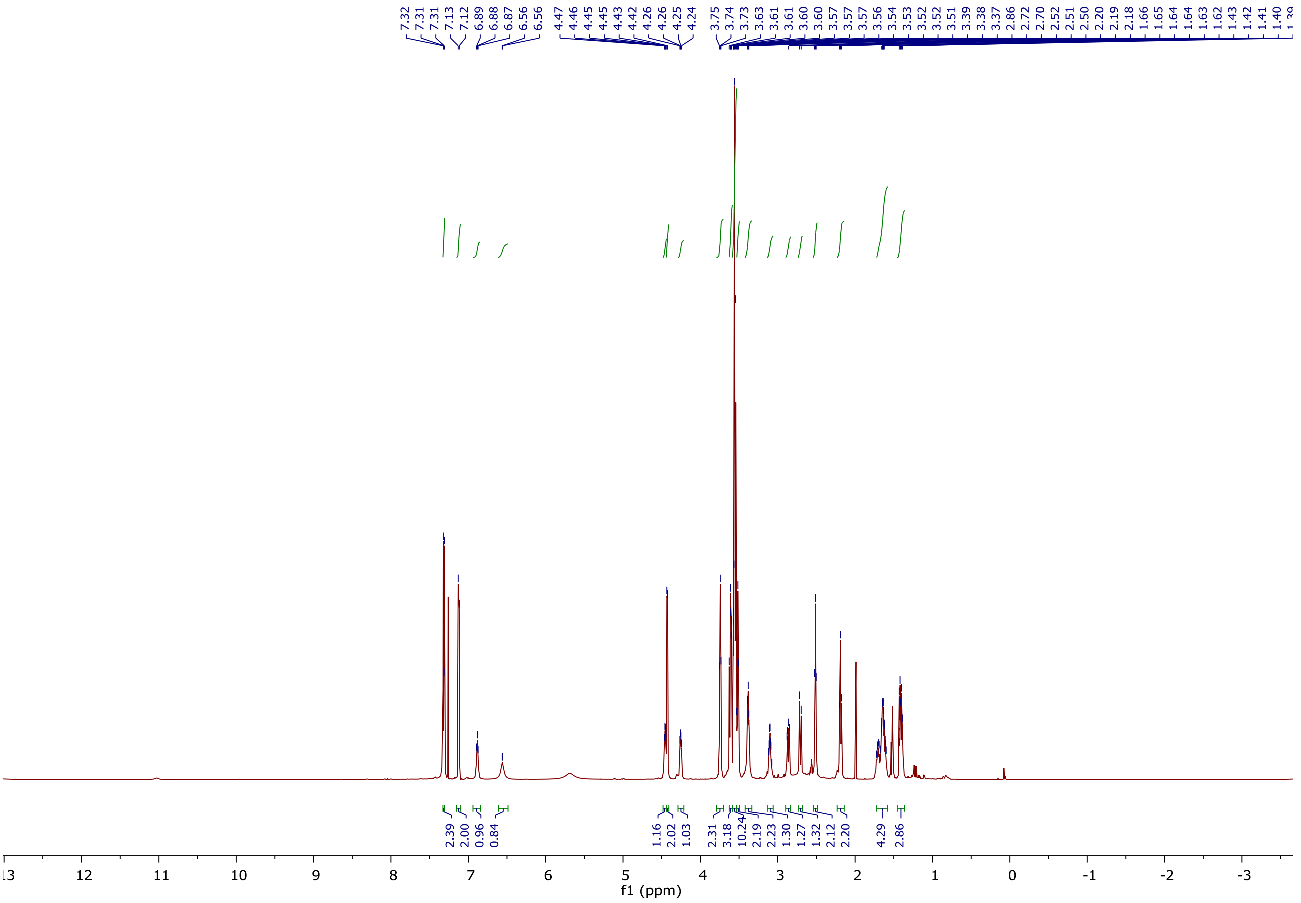

^{13}C NMR of **8:**

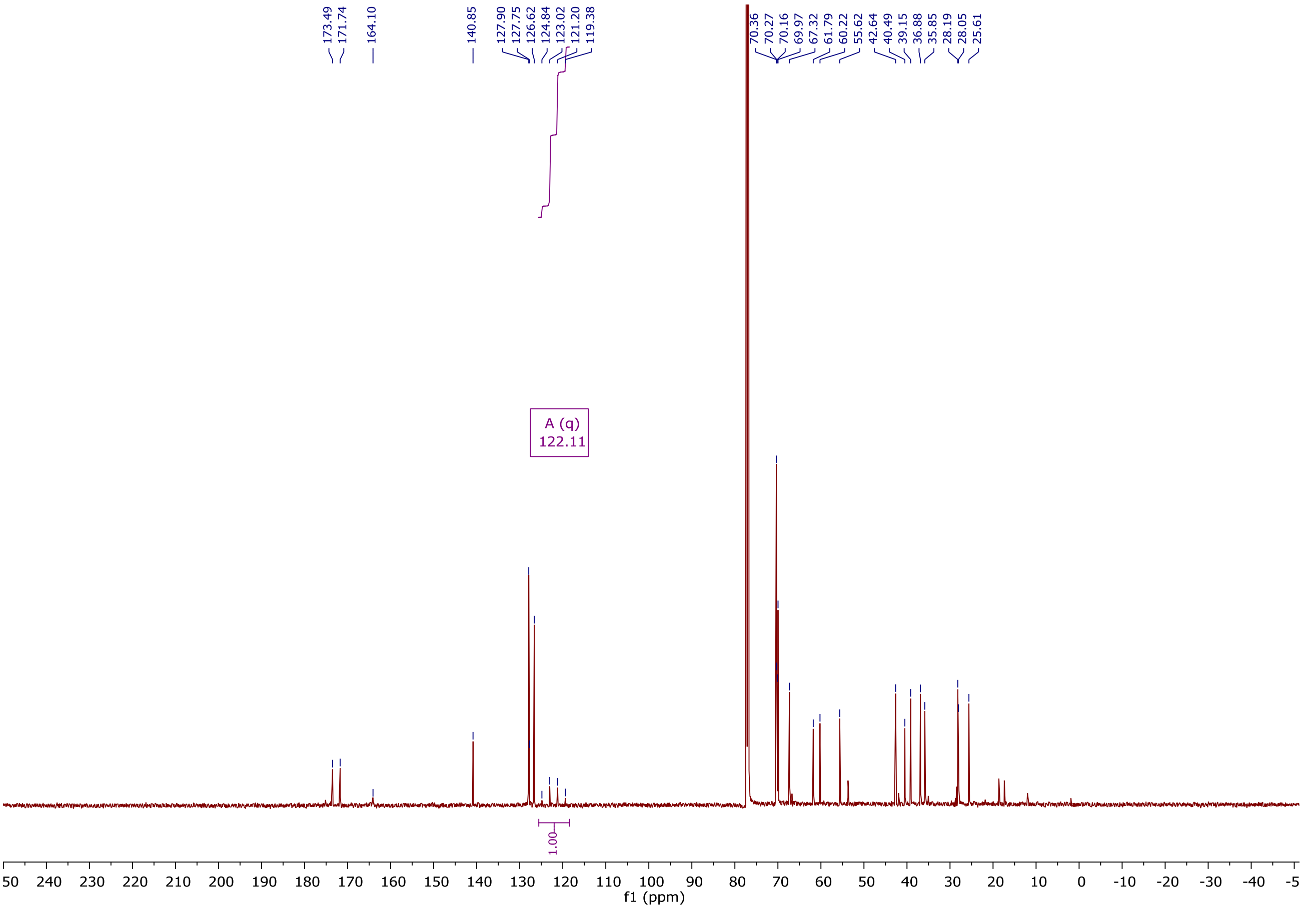

_N_-(4-(3-(trifluoromethyl)-3_H_-diazirin-3-yl)benzyl)acetamide (S4)

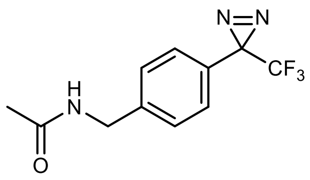

Acetic anhydride (0.125 mL, 1.34 mmol) was added dropwise to a solution of 4-[3-(trifluoromethyl)-3*H*-diazirin-3-yl]benzylamine hydrochloride (112 mg, 0.445 mmol) and DIPEA (0.155 mL, 0.890 mmol) in dry DCM (5 mL). The mixture was stirred at room temperature for 3 h under Ar atmosphere. The mixture was diluted with DCM (100 mL) and washed with sat. $NaHCO_3$ (50 mL), water (50 mL) and brine (50 mL), then dried over Na_2SO_4, filtered and concentrated *in vacuo* to obtain a white solid after drying under high vacuum (<0.1 mbar) (113 mg, 0.439 mmol, 99%).

^{1}H NMR (600 MHz, $CDCl_3$) δ = 7.31 (d, *J* = 8.3 Hz, 2H), 7.16 (d, *J* = 8.1 Hz, 2H), 4.43 (d, *J* = 5.9 Hz, 2H), 2.02 (s, 3H) ppm.

^{13}C NMR (151 MHz, $CDCl_3$) δ = 167.0, 140.2, 128.2, 126.9, 122.1 (q, *J* = 274.6 Hz), 43.1, 23.2 ppm.

HRMS (ESI) m/z: $[M + H]^+$ Calcd for $C_{11}H_{11}F_3N_3O$ 258.0849; Found 258.0898.

^{1}H NMR of **S4:**

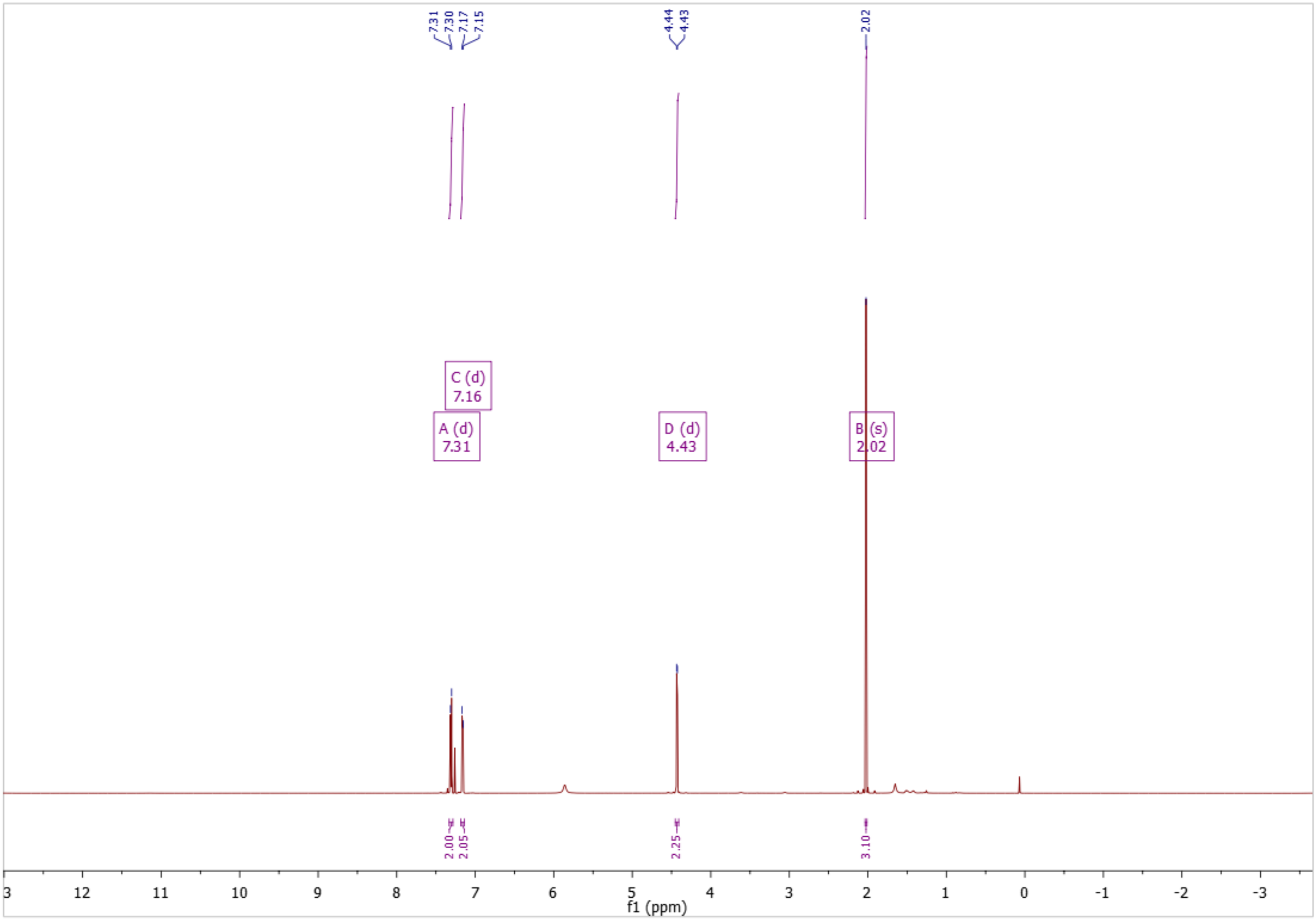

^{13}C NMR of **S4:**

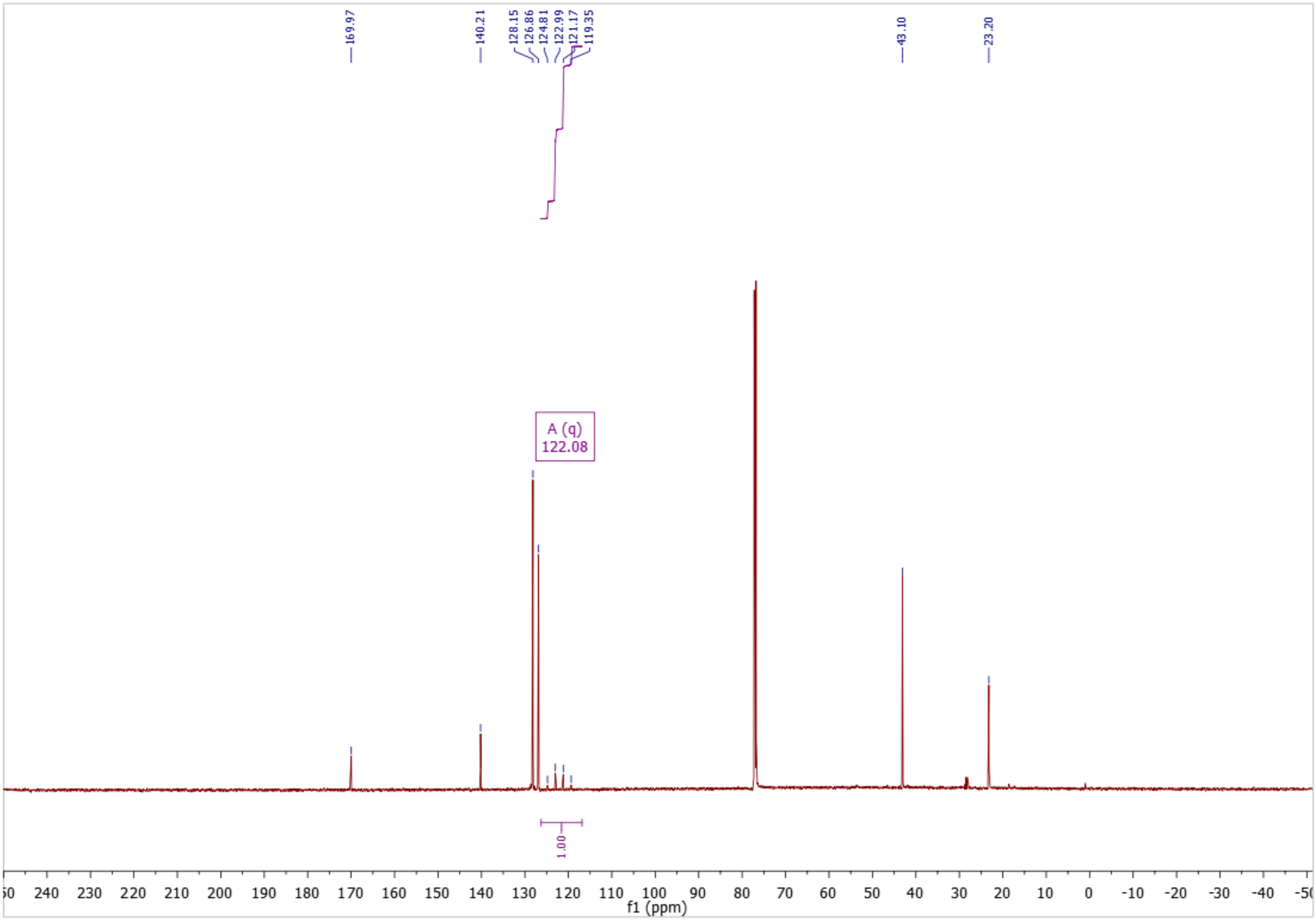

3.2 *Photocatalyst Screening*

3.2.1 Phenyl azide conversion *via* HPLC

To an 8 mL crimp neck vial with magnetic stirrer bar was added a solution of phenyl azide **S3** (1 mM, 10 µL of 100 mM DMSO stock) and photocatalyst (PC, 0.1 mM, 10 µL of 10 mM DMSO stock) in PBS pH = 7.4 (1 mL). The vial was sealed and sparged with Ar for 10 min before being irradiated with a 450 nm light (40W, EvoluChem, PhotoRedOx Box) for 15 min. The solution was then spiked with 4-trifluoromethylbenzoic acid as the internal standard (1 mM) and analysed using analytical HPLC (see Section 2.5 for further details). Conversions were calculated using standard calibration curve of phenyl azide **S3** vs. internal standard and are represented as the average of duplicate runs.

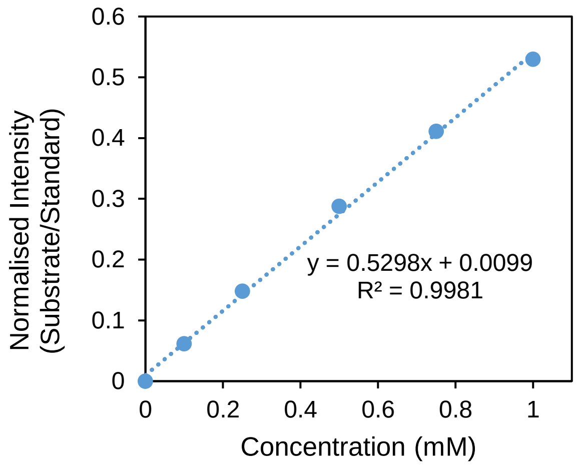

Calibration curve of phenyl azide **S3** vs. internal standard

3.3 Mechanistic Studies

For the photophysical characterisation only solvents of spectroscopic grade were used (from either Carl Roth or Merck).

3.3.1 Stationary Absorption and Emission Spectroscopy

Absorption spectra in the UV/Vis spectral range were recorded using a referenced single beam spectrometer (Cary 60, Agilent). The photostability (in the absence of a substrate) and the photocatalytic activity (in the presence of a substrate) of the photocatalysts were determined by the stationary absorption spectrum in the UV/Vis spectral range along a 10 mm pathlength after stepwise illumination of the sample along a 2 mm pathlength. For this the referenced single beam spectrometer was equipped with an LED (λ_{max} = 455 nm, M455L Thorlabs) as excitation light source orthogonally to the probe beam and the temporal width of the rectangular light pulses were precisely timed by an impulse generator (Aim-TTi TGP 110) as previously described.[22] Emission spectra either at room temperature or at 77 K (in liquid nitrogen) as well as the phosphorescence lifetime at 77 K were recorded with a steady-state fluorescence spectrometer (Fluorolog-3, Horiba Jobin Yvon). The fluorescence quantum yield at room temperature was determined *via* total integration using an Ulbricht sphere (Hamamatsu C9920-02 system equipped with a Spectralon® integrating sphere).

3.3.2 Time-resolved Emission Spectroscopy

A self-constructed Time Correlated Single Photon Counting (TCSPC) setup[23] with single detection wavelength was used to determine the fluorescence lifetimes at room temperature. The sample was excited along a 10 mm pathlength at λ_{exc} = 455 nm, and the emission was recorded orthogonally to this along a 2 mm pathlength at λ_{obs} as indicated in the corresponding Figs. The optical density of the sample was set to *ca.* 0.1 at the excitation wavelength over 10 mm pathlength.

3.3.3 Sub-picosecond Pump/Supercontinuum-Probe Spectroscopy

An in house build setup was used to record the transient absorption (TA) in the UV/Vis as described in detail elsewhere.[24,25] The deazaflavin sample was excited into the $S_1 \leftarrow S_0$ absorption band at λ_{exc} = 420 nm under magic angle (54.73°) with respect to the probe pulse in order to record the pure population dynamics of all excited states. The pump pulse energy was set to ca. 200 nJ at the sample position focused to *ca.* 80 µm. At the sample position the spot size of the probe pulse was *ca.* 40 µm. The sample solution was continuously stirred by a self-constructed magnetic stirrer bar of ca. 0.9 mm thickness in quartz cell with 1 mm pathlength (Starna) driven by an external rotating magnet (glued to a micromotor). The time axis was chosen to be linear from −1 ps up to 4.0 ps in 20 fs steps and logarithmic afterwards until the end of the delay stage of 6.5 ns. An average over 200 transient absorption spectra was recorded at each delay position of a scan, each calculated for a baseline-corrected and referenced single shot. Six independent scans along the time axis were averaged and result in the final spectra. The *ca.* 2 ps chirp of the white light supercontinuum used as probe was corrected for prior to data analysis using the

coherent artefact as an indicator for time zero at each wavelength. No smoothing or filtering procedures were applied to the data.

3.3.4 Nanosecond to millisecond transient absorption spectroscopy

A self-constructed transient absorption spectrometer using a streak camera-based detection system was used as previously reported.[23,26,27] The samples (3 mL) with photocatalysts (OD ~ 0.3 over 10 mm at 410 nm) were excited at 410 nm (4 mJ, ca. 5 ns) and stirred with a micro stirrer bar ensuring a total replacement of the sample prior to each individual recording cycle. No significant degradation of the photocatalyst was observed under the used conditions.

3.3.5 Analysis of transient absorption data and modelling

All transient absorption data were analysed by a well-established method using an in-house written program.[1,24–29] First, a global fit using an exponential mixing is performed resulting in so called decay associated difference spectra (DADS) and their associated optimised rate constants, which are the unique result of the global fit and this treatment does not require any model for the kinetics involved in the transient processes. The number of exponentials in the global fit may be determined by the SVD-based rank analysis, as previously described.[30] From the DADS species associated spectra (SAS) are generated by applying a model that relates the actual species kinetics to the elementary function. In this step the shape of the SAS in terms of identity with well-known spectra or following physical laws decides about the appropriateness of the model. Since this step does not change the χ^2 value of the global fit, this procedure has the advantage that all interpretation is performed with the same quality of fit. Alternatively, known species spectra recorded in this work, may be taken to decompose the recorded time-resolved data using the transpose of the data matrix and using the basis spectra instead of analytical functions. Then the resulting concentration profiles inform about the appropriateness of the basis spectra and the physical reasonability, *i.e.*, total sum of species being constant to 1.

3.3.6 Discussion on bimolecular reactions with the excited singlet state of 4 controlled by diffusion according to Smoluchowski theory

Smoluchowski theory[31–33] is used to quantify, on the one hand, the diffusion limits for a bimolecular reaction and, on the other hand, its efficiency on encounter of the reacting species. In accordance with the Smoluchowski theory, one would not expect to observe mono-exponential kinetics for bimolecular reactions under pseudo-first-order conditions, but instead an additional $e^{\sqrt{t}}$ dependence. The diffusion-controlled quenching rate using the sum of molecular Stokes-radii $r_0 = r_{0,A}+r_{0,B}$ and the sum of diffusion constants $D = D_A + D_B$ of two reacting species A and B is given by

$$k_{Diff}(t) = 4\pi r_0 D N_A p\left(1+\frac{p r_0}{\sqrt{\pi D t}}\right)$$

where p is a factor for the interaction probability upon encounter of the two reactants A and B. In the case p = 1, the reaction is totally diffusion controlled. According to the Stokes-Einstein relation the diffusion coefficient of a molecule can be estimated to:

$$D = \frac{k_B T}{6\pi\eta r_{0,\mathrm{molecule}}}$$

where $r_{0,\mathrm{molecule}}$ might be estimated as the radius of a sphere with the molecular volume. The molecular volume itself might be estimated by the volume enclosed by the solvent-excluded surface (SES). Strategies to determine the SES might be found in.[34] **Table S1** summarizes the corresponding parameters for the description of diffusion-controlled processes taking either DMSO/H_2O (50:50-v/v) or water as solvent, which have viscosities of $2.83 \cdot 10^{-4}$ kg (dm)$^{-1}$ s^{-1} and $8.9 \cdot 10^{-5}$ kg (dm)$^{-1}$ s^{-1} at a temperature of T = 293.15 K.[35]

Table S1: Parameters for the Smoluchowski model. The diffusion coefficients are for 293.15 K. SOLV = DMSO/H_2O (50:50-v/v).

Cat/Sub/Solv	$r_{0,\mathrm{Cat}}$ dm	$r_{0,\mathrm{Sub}}$ dm	D_{Cat} (dm)^{2}s^{-1}	D_{Sub} (dm)^{2}s^{-1}	$4\pi r_0 D N_A$ M^{-1}s^{-1}	$r_0/\sqrt{\pi D}$ $1/\sqrt{\mathrm{s}^{-1}}$
**4/5/PBS**	$3.49 \cdot 10^{-9}$	$3.36 \cdot 10^{-9}$	$7.03 \cdot 10^{-8}$	$7.30 \cdot 10^{-8}$	$7.43 \cdot 10^{9}$	$1.02 \cdot 10^{-5}$
**4/6/PBS**	$3.49 \cdot 10^{-9}$	$3.25 \cdot 10^{-9}$	$7.03 \cdot 10^{-8}$	$7.54 \cdot 10^{-8}$	$7.44 \cdot 10^{9}$	$9.97 \cdot 10^{-6}$
**4/7/PBS**	$3.49 \cdot 10^{-9}$	$3.09 \cdot 10^{-9}$	$7.03 \cdot 10^{-8}$	$7.95 \cdot 10^{-8}$	$7.46 \cdot 10^{9}$	$9.59 \cdot 10^{-6}$
**4/O_2/PBS**	$3.49 \cdot 10^{-9}$	$1.61 \cdot 10^{-9}$	$7.03 \cdot 10^{-8}$	$1.53 \cdot 10^{-7}$	$8.60 \cdot 10^{9}$	$6.09 \cdot 10^{-6}$
**4/O_2/SOLV**	$3.49 \cdot 10^{-9}$	$1.61 \cdot 10^{-9}$	$2.21 \cdot 10^{-8}$	$4.80 \cdot 10^{-8}$	$2.70 \cdot 10^{9}$	$1.09 \cdot 10^{-5}$

In the case of diffusion-controlled quenching of the excited singlet states of **4**, the non-linear contribution falls below 10% after *ca.* 10 ns for all discussed substrate classes. Therefore, the emission decay data obtained from TCSPC with an instruments response function (IRF) of *ca.* 1 ns can sufficiently be analysed by the pseudo-first-order approximation. At a concentration of 10 mM the limiting rates with negligible time-dependence would be in the order of $8 \cdot 10^7$ s^{-1}, which corresponds to a diffusion-controlled lifetime of 12.5 ns. Considering a S_1 lifetime of 4.1 ns for **4** in aqueous solution a yield for a bimolecular diffusion-controlled reaction is expected to be in the order of *ca.* (12.5 ns)$^{-1}$/((12.5 ns)$^{-1}$ + (4.1 ns)$^{-1}$) = 25%. However, in the case of labelling with **4** in live cell only substrate concentrations in the order of 250 μM were used. Correspondingly the bimolecular rate reduces to the order of $1.8 \cdot 10^6$ s^{-1}, which corresponds to a diffusion-controlled lifetime of 555.5 ns and a yield in the order of only *ca.* (555.6 ns)$^{-1}$/((555.6 ns)$^{-1}$ + (4.1 ns)$^{-1}$) = 0.73%. Similarly, due to the solubility of O_2 in water of *ca.* 0.25 mM,[36] only 0.87% of quenching of the S_1 state of **4** due to a bimolecular diffusion-controlled reaction with O_2 is expected. This illustrates that under the used or even possible concentration conditions of the substrates, a reaction with the excited singlet state of **4** does not play a significant role in all investigations performed.

3.4 Antibody conjugation

3.4.1 Cysteine modification of Trastuzumab with deazaflavin 4

200 µL of a 5 mg/mL solution of Trastuzumab in 50 mM Tris buffer containing 100 mM NaCl and 1 mM EDTA, pH 8.3, was mixed with 5.5 µL of a 10 mM TCEP stock (8 eq.). The reduction of the antibody's disulfides was carried out for 30 min at 37 °C. Afterwards, 1.5 µL of a 20 mM solution of deazaflvain **S8** (4 eq.) in DMSO were added and the mixture incubated for 4 hours at 25 °C. The sample was purified by size exclusion chromatography using a BioRad FPLC system on a Superdex 75 16/60 column in PBS. The degree of modification was determined both by intact protein MS as well as UV-Vis absorption spectroscopy. For the latter, the final antibody concentration was measured via the BCA Protein Assay kit according to manufacturer's instructions (Thermo Fisher Scientific, 23250). Concentration of deazaflavin was determined by its absorbance at 391 nm and calculating the concentration according to Beer–Lambert law with an experimentally determined extinction coefficient (ε = 10840 L·mol^{-1}·cm^{-1}). Intact protein mass spectra and UV-Vis absorption spectra revealed a Trastuzumab to deazaflavin ratio of 3.8 and 3.5, respectively. The good correlation between mass spectrometric and spectroscopic results encourages us to use UV/vis measurements as a reliable technique for polyclonal antibodies, since intact protein MS is not readily available.

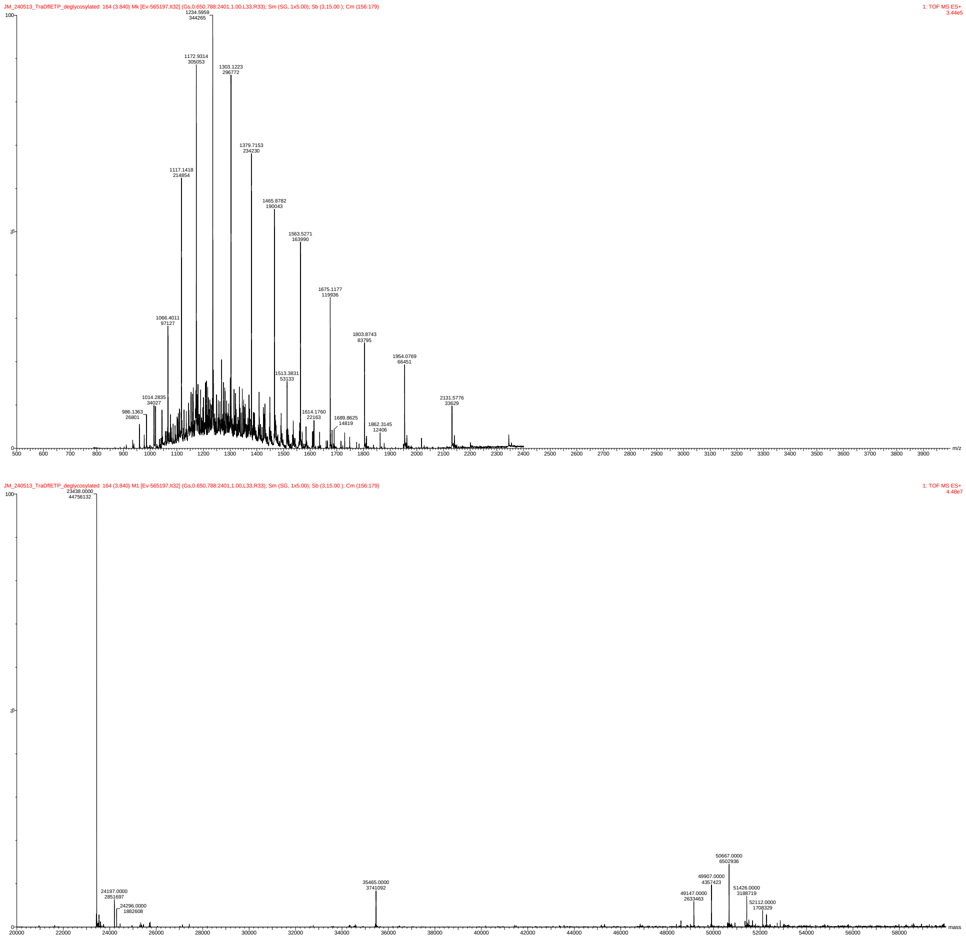

3.4.2 Lysine modification of polyclonal anti-mouse IgG with deazaflavin 4

Procedure adapted from Oslund *et al.* [37] 300 µL of a 2 mg/mL solution of polyclonal goat anti-mouse IgG (AP124, Sigma-Aldrich) was re-buffered in 100 mM $NaHCO_3$ buffer, pH = 8.5. 3 µL of a 100 mM azidobutyric acid NHS ester (Succinimidyl 4-Azidobutyrate) stock solution in DMSO were added and incubated for 1.5 h in the dark. Subsequently, 3 µL more of the stock solution were added and the reaction continued for another 1.5 h in the dark. To remove excess reagent and exchange the buffer, the sample was then filtered using a Zeba Spin desalting column (Thermo Fisher Scientific, 0.5 ml column, 7,000 MWCO) equilibrated with 50 mM Tris, pH = 8.0, according to manufacturer's instructions. For the click reaction, 200 µL of the azide-modified antibody were used. The Click-iT protein reaction buffer kit (Thermo Fisher Scientific, C10276) was applied with slight deviations from the manufacture's protocol. First, 15 µL of a stock solution containing 5 mM deazaflavin **S6** in DMSO were added to the antibody. Then, 12.5 µL Copper sulfate were added to the reaction, followed by 12.5 µL of additive 1. The sample was carefully mixed for 3 min at room temperature, followed by the addition of 15 µL of additive 2. The sample was then incubated in the dark for 30 min at room temperature. The sample was filtered using a Zeba Spin desalting column (Thermo Fisher Scientific, 0.5 ml column, 7,000 MWCO) and buffer exchanged into PBS twice. To determine the degree of modification, the final antibody concentration was measured via the BCA Protein Assay kit according to manufacturer's instructions (Thermo Fisher Scientific, 23250).

Concentration of deazaflavin was determined by its absorbance at 391 nm and calculating the concentration according to Beer–Lambert law with an experimentally determined extinction coefficient (ε = 10840 $L \cdot mol^{-1} \cdot cm^{-1}$). Under these conditions, an antibody to deazaflavin ratio of 3 could be achieved.

3.5 HaloTag-GluR2 expression and self-labelling validation by microscopy

HeLa cells were transfected according to the **Supplementary Information Section 2.8**. After 24 h, HeLa-HaloTag-mGluR2 cells were seeded onto 8-well plates for another 24 h. Prepared cells were washed twice with PBS, followed by treatment with **12**, **13**, or **14** (final concentration of 10 µM from 10 mM DMSO stocks) in HBSS for 30 min at 23 °C. Unreacted molecules were washed off with PBS, followed by treatment of the cells with TMR-CA (final concentration of 1 µM from 10 mM DMSO stock) in HBSS for 15 min. Excess dye was washed off with PBS, and imaging was performed by confocal laser scanning microscopy using the Zeiss LSM780 confocal microscope with a 40x, 1.4-NA Plan-Apochromat oil immersion objective. Images were processed using Fiji software.

3.6 Peptide Synthesis

All peptides were synthesised by standard fluorenylmethoxycarbonyl (Fmoc)-solid-phase peptide synthesis (SPPS) on Rink amide resin (0.1 mmol scale, 0.22 mmol/g). Amino acid couplings were done using 5 equivalents of amino acid with 5 equivalents of HATU with 10 equivalents of DIPEA in DMF. Fmoc removal was accomplished by incubating the resin in 20% piperidine in DMF for 3 x 5 min at room temperature. Arginine was incorporated with 2,2,4,6,7-pentamethyldihydrobenzofuran-5-sulfonyl (Pbf) protection. *N*-terminal deazaflavin conjugation was achieved using 2 equivalents of deazaflavin **11** and HATU with 4 equivalents of DIPEA in DMF for 2 h at room temperature in the dark. Cyclisation of the cyclic R_{10} (L/D alternating sequence) peptide was done by incorporation of a lysine and glutamic acid residue flanking the CPP sequence, orthogonally protected by monomethoxytrityl (Mmt) and 2-phenylisopropyl (2-PhiPr), respectively. The orthogonal protecting groups were removed by incubating the resin in 2% TFA and 5% TIS in DCM for 10 min at room temperature until a colourless solution was obtained. The peptide was then cyclised using 1 equivalent of HATU and 2 equivalents of DIPEA in DMF for 2 h at room temperature. Diazirine-containing peptides were prepared using Fmoc-L-Photo-Phe-OH (Iris Biotech, FAA5690).[38] Final deprotection and cleavage from the resin was performed using 95% TFA, 2.5% TIS and 2.5% H_2O for 6 h at room temperature. Crude peptides were precipitated in cold Et_2O and purified by preparative HPLC as described in **Section 2.4**.

Peptide sequences and analytical data:

Deazaflavin-R_{10} (18)

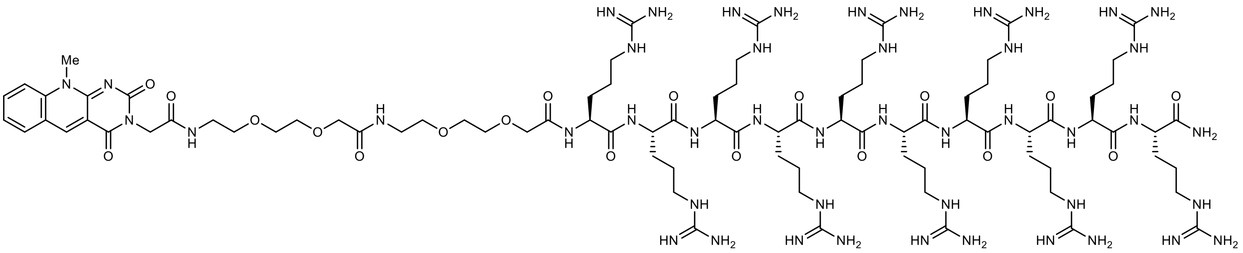

UPLC-UV (220 nm) trace:

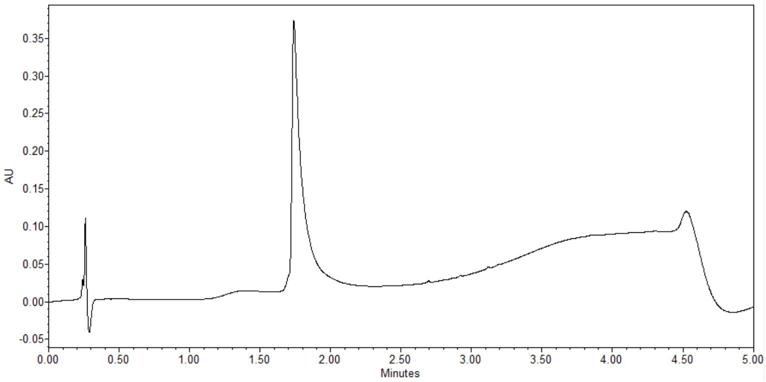

HRMS (ESI) m/z: $[M + 3H]^{3+}$ Calcd 712.7572; Found 712.7576. **Yield** = 38 mg (12%)

Deazaflavin-cR$_{10}$ (19)

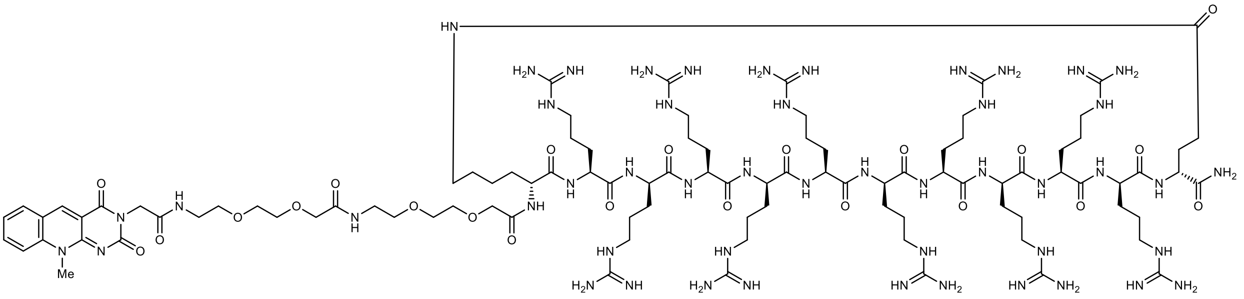

UPLC-UV (220 nm) trace:

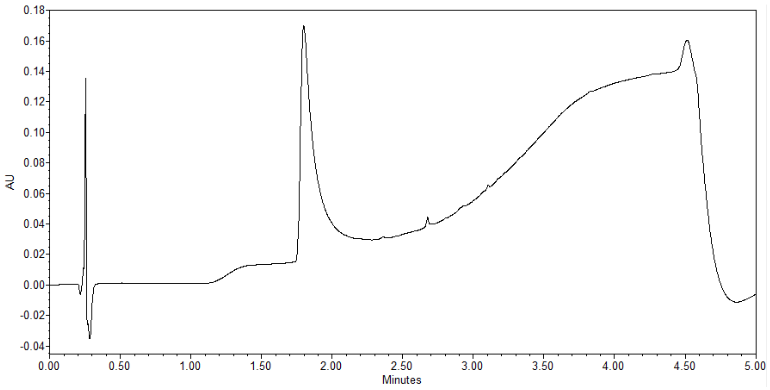

HRMS (ESI) m/z: $[M + 3H]^{3+}$ Calcd 792.4662; Found 792.4478. **Yield** = 21 mg (6%)

Biotin-diazirine-R$_{10}$ (20)

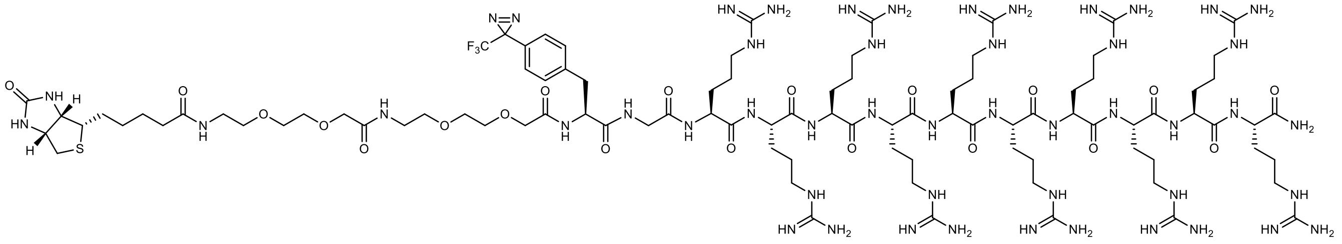

UPLC-UV (220 nm) trace:

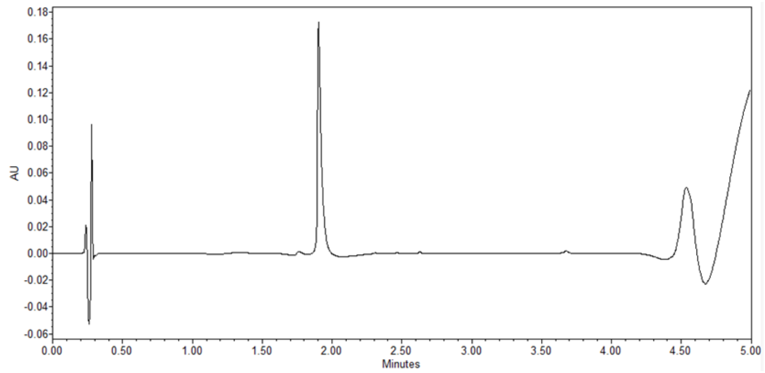

HRMS (ESI) m/z: $[M + 3H]^{3+}$ Calcd 803.4572; Found 803.4725. **Yield** = 50.5 mg (28%)

Biotin-diazirine-cR$_{10}$ (21)

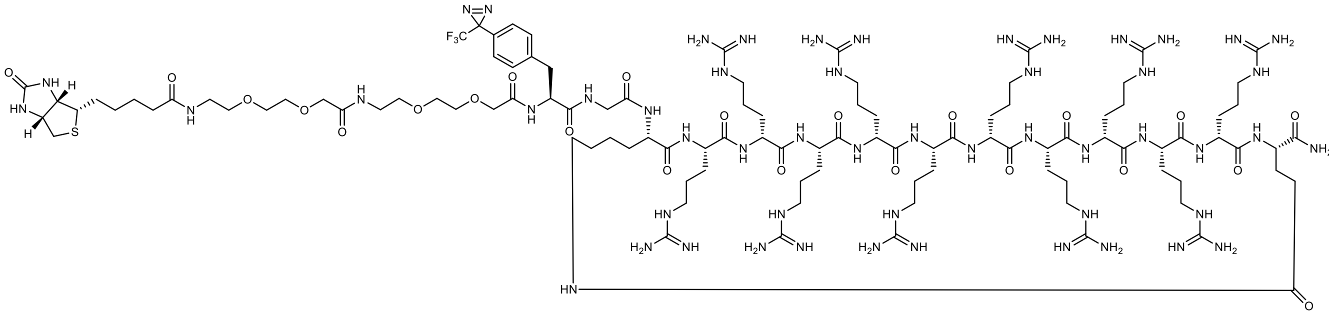

UPLC-UV (220 nm) trace:

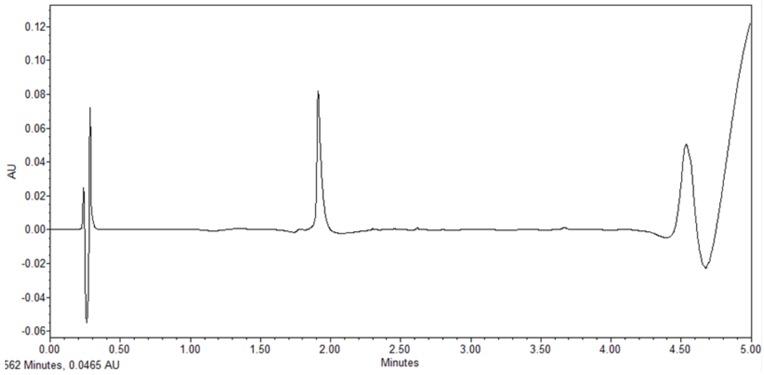

HRMS (ESI) m/z: $[M + 3H]^{3+}$ Calcd 883.1662; Found 883.1752. **Yield** = 11.6 mg (6%)

3.7 Intracellular DarT-labelling for western blot analysis

HeLa cells were seeded onto 6 cm dishes and were allowed to attach for 48 h or until 80% confluence. Cells were washed twice with PBS before treating with 5 µM **16**, **18,** or **19** in serum-free FluoroBrite DMEM (diluted from 10 mM DMSO stocks) at 37 °C for 1 h. The treatment solution was discarded and the cells were washed with PBS prior to the addition of 250 µM of diazirine **8** in FluoroBrite DMEM (diluted from 100 mM DMSO stock) and further incubation for 30 min. The dishes were transferred to the cold room (8 °C) and irradiated as stated **in Supplementary Information Section 2.1** for 0, 1, 3, 5, 10, 15 min for **Supplementary Fig. S12** and 15 min for **Supplementary Fig. S13**. Irradiated cells were placed on ice and washed twice with ice-cold PBS, making sure to aspirate out all residual liquid.

For **Supplementary Fig. S12,** cell lysates were made by adding 200 µL of radioimmunoprecipitation (RIPA) buffer with 1× cOmplete protease inhibitor (Roche) and 1× Benzonase nuclease (Merck) to each plate and scraping the cells. Lysates were collected into pre-chilled 1.5 mL microcentrifuge tubes and incubated at room temperature (~25 °C) for 15 min in a tabletop shaker. The lysates were centrifuged at 12000 × *g*, 5 min, 4 °C. The supernatants were transferred into new pre-chilled 1.5 microcentrifuge tubes. Protein concentrations were determined by BCA assay (Pierce™ Thermo Fisher).

For **Supplementary Fig. S13**, irradiated cells were dislodged and collected by scraping. Fractionation of cytosolic (Cyt), nuclear soluble (N^{sol}), and nuclear insoluble (N^{insol}) proteins was performed using a Nuclear extract Kit according to the manufacturer's specifications (Abcam). Briefly, collected cells were resuspended and incubated for 10 min in ice-cold cytoplasm extraction buffer with protease inhibitors and DTT. The suspension was centrifuged (1000 × *g*, 3 min, 4 °C) and the supernatant was collected (Cyt). The pellet was resuspended in Nuclear Extraction Buffer with protease inhibitors and DTT, and was incubated on ice for 15 min with vortexing every 5 min. The solution was centrifuged (5000 × *g*, 3 min, 4 °C), and the supernatant was collected (N^{sol}). Finally, the pellet was again resuspended in Nuclear Lysis Buffer with protease inhibitor and DTT, briefly sonicated (30% pulse, 10 s), and used as N^{insol}. Protein concentrations were determined by BCA assay (Pierce™ Thermo Fisher) for each fraction.

The samples (10 µg) were mixed with Laemmli buffer with β-mercaptoethanol followed by western blotting for biotin according to "**Western blot procedure**" Methods section in the Main Text. Lane intensities were determined by FIJI software and were normalized against β-actin band intensities.

References

1. Graml, A., Neveselý, T., Jan Kutta, R., Cibulka, R. & König, B. Deazaflavin reductive photocatalysis involves excited semiquinone radicals. *Nat. Commun.* **11**, 1–11 (2020).
2. Bliese, M., Launikonis, A., Loder, J. W., Mau, W. H. A. & Sasse, W. H. F. Photoreduction of deazaflavin. Spectroscopic investigations. *Aust. J. Chem.* **36**, 1873–1883 (1983).
3. Raimer, B., Jones, P. G. & Lindel, T. Quantum chemical calculation of 19F NMR chemical shifts of trifluoromethyl diazirine photoproducts and precursors. *J. Fluor. Chem.* **166**, 8–14 (2014).
4. Gao, B., Zhao, Y., Ni, C. & Hu, J. AgF-mediated fluorinative homocoupling of gem - difluoroalkenes. *Org. Lett.* **16**, 102–105 (2014).
5. Zhao, B. *et al.* Palladium catalyzed mono and difunctionalization of hexafluorobut-2-yne. *Tetrahedron Lett.* **57**, 4345–4347 (2016).
6. Bentea, L., Watzky, M. A. & Finke, R. G. Sigmoidal Nucleation and Growth Curves Across Nature Fit by the Finke−Watzky Model of Slow Continuous Nucleation and Autocatalytic Growth: Explicit Formulas for the Lag and Growth Times Plus Other Key Insights. (2008) doi:10.1021/acs.jpcc.6b12021.
7. Hanopolskyi, A. I., Smaliak, V. A., Novichkov, A. I. & Semenov, S. N. Autocatalysis: Kinetics, Mechanisms and Design. *ChemSystemsChem* **3**, e2000026 (2021).
8. Lutkus, L. V., Rickenbach, S. S. & McCormick, T. M. Singlet oxygen quantum yields determined by oxygen consumption. *J. Photochem. Photobiol. A Chem.* **378**, 131–135 (2019).
9. Kou, M. *et al.* Determination of singlet oxygen quantum yield based on the behavior of solvent dimethyl sulfoxide oxidation by singlet oxygen. *Anal. Chim. Acta* **1329**, 343222 (2024).
10. Geertsema, E. M., Schoevaars, A. M., Meetsma, A. & Feringa, B. L. Bisthioxanthylidene biscrown ethers as potential stereodivergent chiral ligands. *Org. Biomol. Chem.* **4**, 4101–4112 (2006).
11. Geertsema, E. M., Hoen, R., Meetsma, A. & Feringa, B. L. Asymmetric synthesis of bi(thio)xanthylidene overcrowded alkenes. *European J. Org. Chem.* 3596–3605 (2006) doi:10.1002/ejoc.200600280.
12. Mojr, V. *et al.* Tailoring flavins for visible light photocatalysis: organocatalytic [2+2] cycloadditions mediated by a flavin derivative and visible light. *Chem. Commun.* **51**, 12036–12039 (2015).
13. Geri, J. B. *et al.* Microenvironment mapping via Dexter energy transfer on immune cells. *Science (80-.).* **367**, 1091–1097 (2020).
14. Huth, S. W. *et al.* μMap Photoproximity Labeling Enables Small Molecule Binding Site Mapping. *J. Am. Chem. Soc.* **145**, 16289–16296 (2023).
15. Trowbridge, A. D. *et al.* Small molecule photocatalysis enables drug target identification via energy transfer. *Proc. Natl. Acad. Sci. U. S. A.* **119**, 1–8 (2022).
16. Yoneda, F. [38] Syntheses of 5-Deazaflavins. *Methods Enzymol.* **66**, 267–277 (1980).
17. Gockel, S. N., Buchanan, T. L. & Hull, K. L. Cu-Catalyzed Three-Component Carboamination of Alkenes. *J. Am. Chem. Soc.* **140**, 58–61 (2018).

18. Roßmann, K. *et al.* N-Methyl deuterated rhodamines for protein labelling in sensitive fluorescence microscopy. *Chem. Sci.* **13**, 8605–8617 (2022).
19. Stieger, C. E. *et al.* DFT-Guided Discovery of Ethynyl-Triazolyl-Phosphinates as Modular Electrophiles for Chemoselective Cysteine Bioconjugation and Profiling. *Angew. Chemie - Int. Ed.* **61**, (2022).
20. Stieger, C. E., Franz, L., Körlin, F. & Hackenberger, C. P. R. Diethynyl Phosphinates for Cysteine-Selective Protein Labeling and Disulfide Rebridging. *Angew. Chemie Int. Ed.* **60**, 15359–15364 (2021).
21. Oakley, J. V. *et al.* Radius measurement via super-resolution microscopy enables the development of a variable radii proximity labeling platform. *Proc. Natl. Acad. Sci. U. S. A.* **119**, 1–8 (2022).
22. Kutta, R. J. *et al.* The photochemical mechanism of a B 12-dependent photoreceptor protein. *Nat. Commun.* **6**, 1–11 (2015).
23. Kutta, R. J. Blitzlichtphotolyse. (Universität Regensburg, 2012).
24. Pavlovska, T. *et al.* Tuning Deazaflavins Towards Highly Potent Reducing Photocatalysts Guided by Mechanistic Understanding – Enhancement of the Key Step by the Internal Heavy Atom Effect. *Chem. - A Eur. J.* **28**, e202200768 (2022).
25. Bergwinkl, S., Nuernberger, P., Dick, B. & Kutta, R. J. Enhanced Intersystem Crossing in a Thiohelicene. *ChemPhotoChem* **8**, e202300343 (2024).
26. Kutta, R. J., Langenbacher, T., Kensy, U. & Dick, B. Setup and performance of a streak camera apparatus for transient absorption measurements in the ns to ms range. *Appl. Phys. B Lasers Opt.* **111**, 203–216 (2013).
27. Dick, B., Kensy, U. & Roger-Jan, K. Transient absorption. *Chem. Photocatal.* 295–318 (2013) doi:10.1515/9783110269246.295.
28. Kutta, R. J., Archipowa, N. & Scrutton, N. S. The sacrificial inactivation of the blue-light photosensor cryptochrome from: Drosophila melanogaster. *Phys. Chem. Chem. Phys.* **20**, 28767–28776 (2018).
29. Archipowa, N., Kutta, R. J., Heyes, D. J. & Scrutton, N. S. Stepwise Hydride Transfer in a Biological System: Insights into the Reaction Mechanism of the Light-Dependent Protochlorophyllide Oxidoreductase. *Angew. Chemie - Int. Ed.* **57**, 2682–2686 (2018).
30. Lanzl, K., Sanden-Flohe, M. V., Kutta, R. J. & Dick, B. Photoreaction of mutated LOV photoreceptor domains from Chlamydomonas reinhardtii with aliphatic mercaptans: Implications for the mechanism of wild type LOV. *Phys. Chem. Chem. Phys.* **12**, 6594–6604 (2010).
31. Smoluchowski, M. v. Drei vorträge über diffusion Brownsche molekular bewegung und koagulation von kolloidteichen. *Phys. Z.* **17**, 557–571, 585–599 (1916).
32. Smoluchowski, M. v. Versuch einer mathematischen Theorie der Koagulationskinetik kolloider Lösungen. *Zeitschrift für Phys. Chemie* **92U**, 129–168 (1918).
33. Birks, J. B. *Photophysics of Aromatic Molecules*. (Wiley-Interscience, 1970).
34. Pavanï, R. & Ranghino, G. A method to compute the volume of a molecule. *Comput. Chem.* **6**,

133–135 (1982).

35. Lebel, R. G. & Goring, D. A. I. Density, Viscosity, Refractive Index, and Hygroscopicity of Mixtures of Water and Dimethyl Sulfoxide. *J. Chem. Eng. Data* **7**, 100–101 (1962).

36. Montgomery, H. A. C., Thom, N. S. & Cockburn, A. Determination of dissolved oxygen by the winkler method and the solubility of oxygen in pure water and sea water. *J. Appl. Chem.* **14**, 280–296 (1964).

37. Oslund, R. C. *et al.* Detection of cell–cell interactions via photocatalytic cell tagging. *Nat. Chem. Biol.* **18**, 850–858 (2022).

38. Kawaguchi, Y. *et al.* Identification of cellular proteins interacting with octaarginine (R8) cell-penetrating peptide by photo-crosslinking. *Bioorganic Med. Chem. Lett.* **23**, 3738–3740 (2013).

4 Source data for Supplementary Figure 13

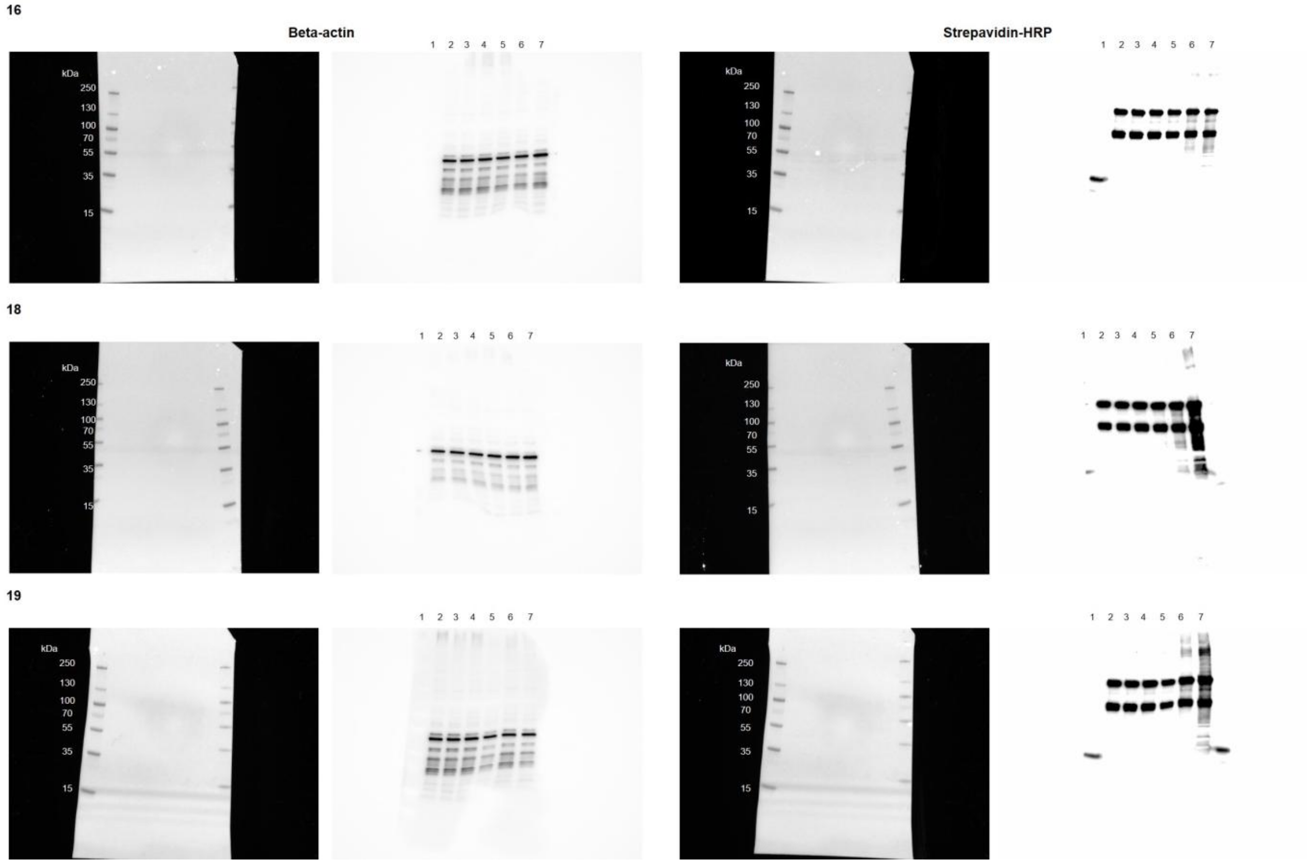

5 Source data for Supplementary Figure 14

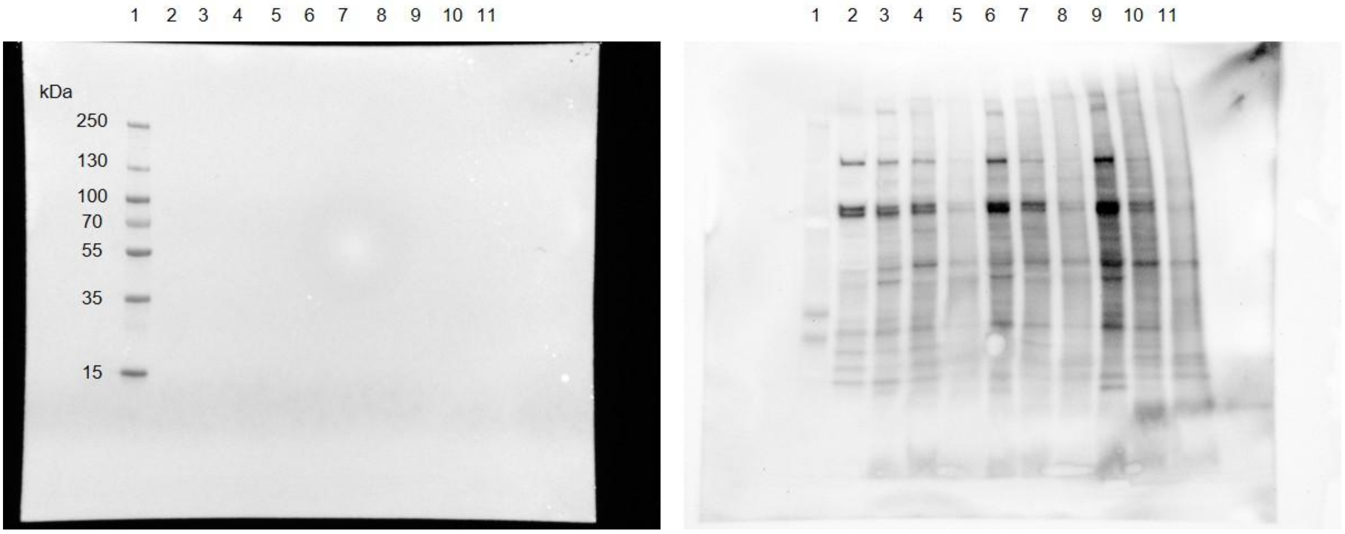

Supplement: Supplementary file 1 — Supplementary Figs. 1–18, Discussion, General information, Experimental procedures and Source data for Supplementary Figs. 13 and 14. [file 41557_2025_1931_MOESM1_ESM.pdf]
