# Supplementary material for: Energy-transfer photoproximity labelling in live cells using an organic cofactor
Source: Nat Chem. 2025 Sep 17;17(12):1928–40. doi: 10.1038/s41557-025-01931-8 (PMC12669049; doi:10.1038/s41557-025-01931-8)
Supplement: Supplementary file 2 — Reporting Summary [file 41557_2025_1931_MOESM2_ESM.pdf]

nature portfolio

Corresponding author(s): Christian P.R. Hackenberger

Last updated by author(s): Jul 3, 2025

Reporting Summary

Nature Portfolio wishes to improve the reproducibility of the work that we publish. This form provides structure for consistency and transparency in reporting. For further information on Nature Portfolio policies, see our Editorial Policies and the Editorial Policy Checklist.

Statistics

For all statistical analyses, confirm that the following items are present in the figure legend, table legend, main text, or Methods section.

n/a	Confirmed	
☐	☒	The exact sample size (*n*) for each experimental group/condition, given as a discrete number and unit of measurement
☐	☒	A statement on whether measurements were taken from distinct samples or whether the same sample was measured repeatedly
☐	☒	The statistical test(s) used AND whether they are one- or two-sided<br>*Only common tests should be described solely by name; describe more complex techniques in the Methods section.*
☒	☐	A description of all covariates tested
☒	☐	A description of any assumptions or corrections, such as tests of normality and adjustment for multiple comparisons
☐	☒	A full description of the statistical parameters including central tendency (e.g. means) or other basic estimates (e.g. regression coefficient) AND variation (e.g. standard deviation) or associated estimates of uncertainty (e.g. confidence intervals)
☒	☐	For null hypothesis testing, the test statistic (e.g. *F*, *t*, *r*) with confidence intervals, effect sizes, degrees of freedom and *P* value noted<br>*Give P values as exact values whenever suitable.*
☒	☐	For Bayesian analysis, information on the choice of priors and Markov chain Monte Carlo settings
☒	☐	For hierarchical and complex designs, identification of the appropriate level for tests and full reporting of outcomes
☒	☐	Estimates of effect sizes (e.g. Cohen's *d*, Pearson's *r*), indicating how they were calculated

Our web collection on statistics for biologists contains articles on many of the points above.

Software and code

Policy information about availability of computer code

Data collection	ChemDraw 22 (chemical structures)<br>FACSDiva (modified CAPA assay)<br>Nikon NIS Element AR v. 4 (Fluorescence microscopy)<br>BioRad Image Lab 6 (Western Blots)
Data analysis	GraphPad Prism 10 (graphs, CP50 calculation)<br>FIJI 2.16.0/1.54p (Image analysis)<br>BioRad Image Lab 6 (Western Blots)<br>FlowJo 10.6.1-1 (flow cytometry)<br>FragPipe v22.0 (proteomic data analysis)<br>ShinyGO 0.80 (GO term analysis)<br>RStudio v2024.04.2 (proteomic data presentation)

For manuscripts utilizing custom algorithms or software that are central to the research but not yet described in published literature, software must be made available to editors and reviewers. We strongly encourage code deposition in a community repository (e.g. GitHub). See the Nature Portfolio guidelines for submitting code & software for further information.

Data

Policy information about availability of data

All manuscripts must include a data availability statement. This statement should provide the following information, where applicable:

- Accession codes, unique identifiers, or web links for publicly available datasets
- A description of any restrictions on data availability
- For clinical datasets or third party data, please ensure that the statement adheres to our policy

All experimental data, materials and methods, analytical procedures, cell assays, copies of spectra are available in the Main text and in the electronic supplementary information (ESI). The mass spectrometry proteomics data have been deposited to the ProteomeXchange Consortium via the PRIDE partner repository with the dataset identifier PXD056778. Proteomic data are available from the corresponding author upon reasonable request.

Research involving human participants, their data, or biological material

Policy information about studies with human participants or human data. See also policy information about sex, gender (identity/presentation), and sexual orientation and race, ethnicity and racism.

Reporting on sex and gender	not applicable
Reporting on race, ethnicity, or other socially relevant groupings	not applicable
Population characteristics	not applicable
Recruitment	not applicable
Ethics oversight	not applicable

Note that full information on the approval of the study protocol must also be provided in the manuscript.

Field-specific reporting

Please select the one below that is the best fit for your research. If you are not sure, read the appropriate sections before making your selection.

☒ Life sciences ☐ Behavioural & social sciences ☐ Ecological, evolutionary & environmental sciences

For a reference copy of the document with all sections, see nature.com/documents/nr-reporting-summary-flat.pdf

Life sciences study design

All studies must disclose on these points even when the disclosure is negative.

Sample size	All biological experiments and proteomic experiments were performed N=3 biological replicates.<br>For imaging, different regions of interest were taken (n=3) per well.
Data exclusions	not applicable
Replication	All experiments were performed in three biological replicates per conditions.
Randomization	All biological samples originated from the same population of cells and were randomly assigned for group and treatment conditions.
Blinding	not appliable

Reporting for specific materials, systems and methods

We require information from authors about some types of materials, experimental systems and methods used in many studies. Here, indicate whether each material, system or method listed is relevant to your study. If you are not sure if a list item applies to your research, read the appropriate section before selecting a response.

Materials & experimental systems

n/a	Involved in the study	
☐	☒	Antibodies
☐	☒	Eukaryotic cell lines
☒	☐	Palaeontology and archaeology
☒	☐	Animals and other organisms
☒	☐	Clinical data
☒	☐	Dual use research of concern
☒	☐	Plants

Methods

n/a	Involved in the study	
☒	☐	ChIP-seq
☐	☒	Flow cytometry
☒	☐	MRI-based neuroimaging

Antibodies

Antibodies used	Trastuzumab,<br>polyclonal goat anti-mouse IgG (AP124, Sigma-Aldrich),<br>anti-HER2 antibody (Invitrogen MA513105),<br>Mouse IgG Isotype (Invitrogen P3.6.2.8.1)
Validation	antibody profiles are from online databases

Eukaryotic cell lines

Policy information about cell lines and Sex and Gender in Research

Cell line source(s)	HeLa - CCL2: American Type Culture Collection (ATCC);<br>HEK293T, MCF7: German Collection of Microorganisms and Cell Cultures;<br>SKBR3: LMU München Biozentrum
Authentication	Cell profiles are validated by manufacturers
Mycoplasma contamination	cells are not contaminated with mycoplasma
Commonly misidentified lines<br>(See ICLAC register)	not applicable

Plants

Seed stocks	not applicable
Novel plant genotypes	not applicable
Authentication	not applicable

Flow Cytometry

Plots

Confirm that:

☒ The axis labels state the marker and fluorochrome used (e.g. CD4-FITC).

☒ The axis scales are clearly visible. Include numbers along axes only for bottom left plot of group (a 'group' is an analysis of identical markers).

☒ All plots are contour plots with outliers or pseudocolor plots.

☒ A numerical value for number of cells or percentage (with statistics) is provided.

Methodology

Sample preparation	Cells were trypsinized, collected and washed with PBS 2x. After centrifugation, cells were resuspended in PBS before FACS analysis

Instrument	BD LSRFortessa
Software	FACSDIva, FlowJo 10.6.1-1
Cell population abundance	10,000 gated cells were collected for analysis (TMR fluorescence intensity)
Gating strategy	SSC vs. FSC (area) gating of live population. Mean fluorescence intensity of TMR intensity histogram was determined.

☐ Tick this box to confirm that a figure exemplifying the gating strategy is provided in the Supplementary Information.
