# Supplementary figures and images for: Energy-transfer photoproximity labelling in live cells using an organic cofactor
Source: Nat Chem. 2025 Sep 17;17(12):1928–40. doi: 10.1038/s41557-025-01931-8 (PMC12669049; doi:10.1038/s41557-025-01931-8)

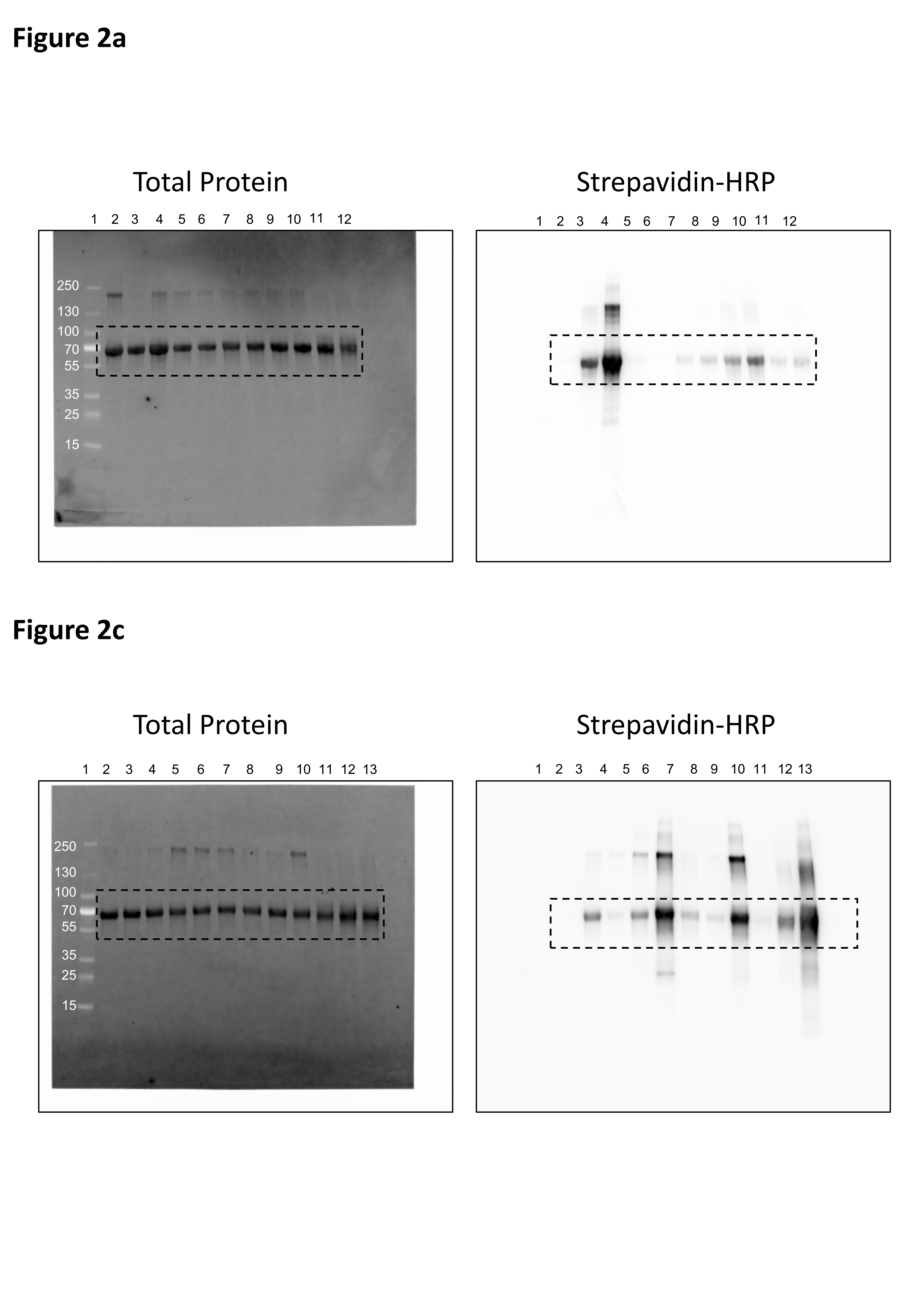

Supplement: Supplementary file 3 — Unprocessed western blots. [file 41557_2025_1931_MOESM3_ESM.tif]

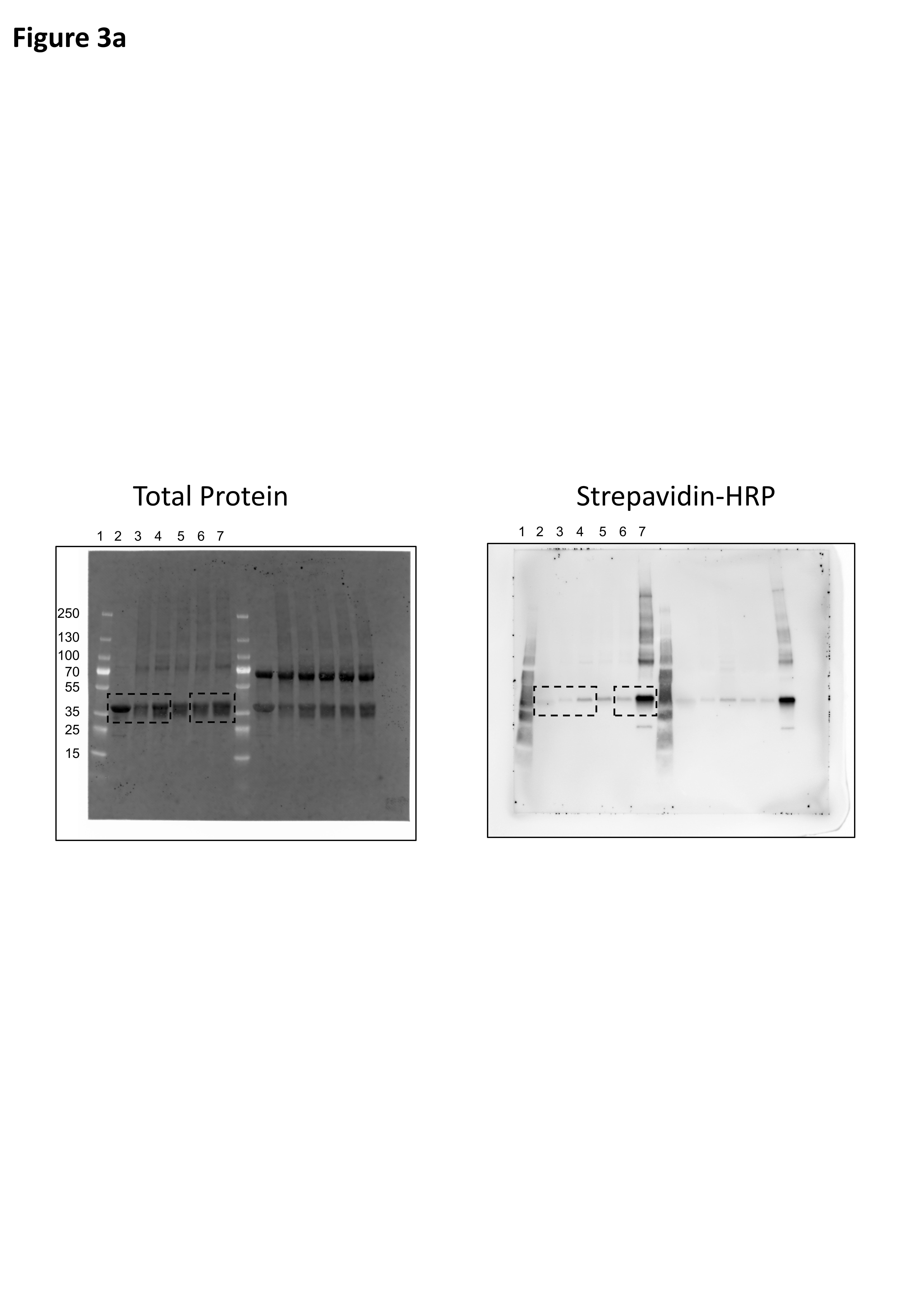

Supplement: Supplementary file 5 — Unprocessed western blots. [file 41557_2025_1931_MOESM5_ESM.tif]
