# Supplementary material for: Energy-transfer photoproximity labelling in live cells using an organic cofactor
Source: Nat Chem. 2025 Sep 17;17(12):1928–40. doi: 10.1038/s41557-025-01931-8 (PMC12669049; doi:10.1038/s41557-025-01931-8)

Extended Figure 2a

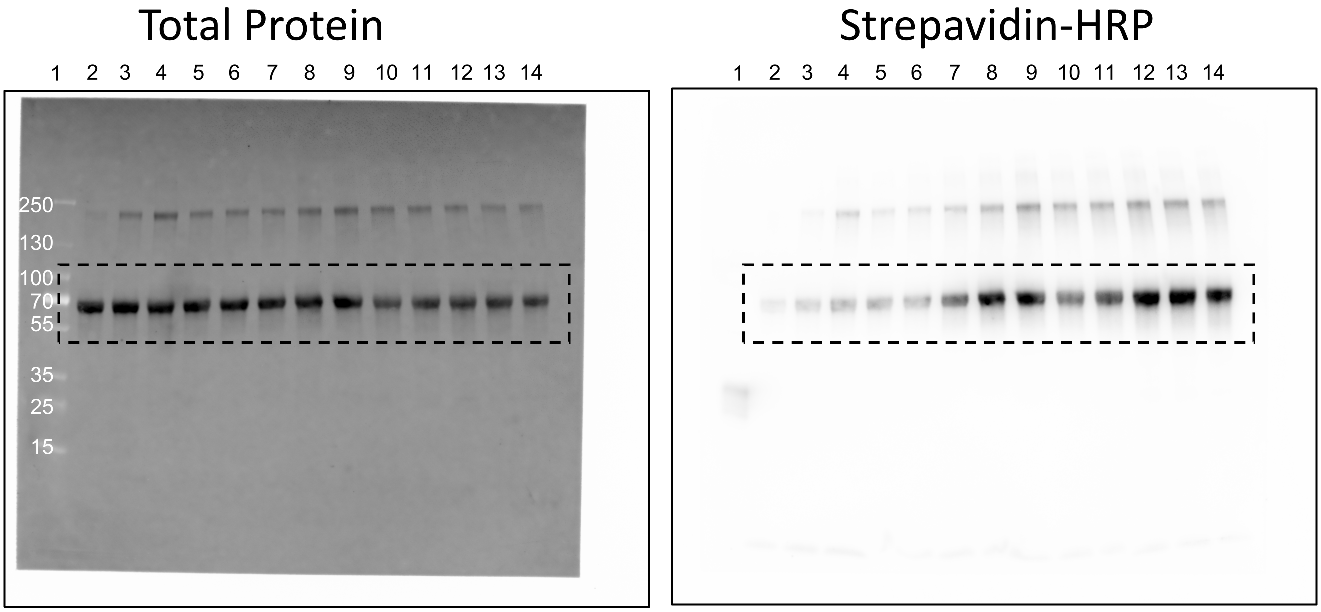

Extended Figure 2b

Bovine Serum Albumin

Total Protein | Strepavidin-HRP

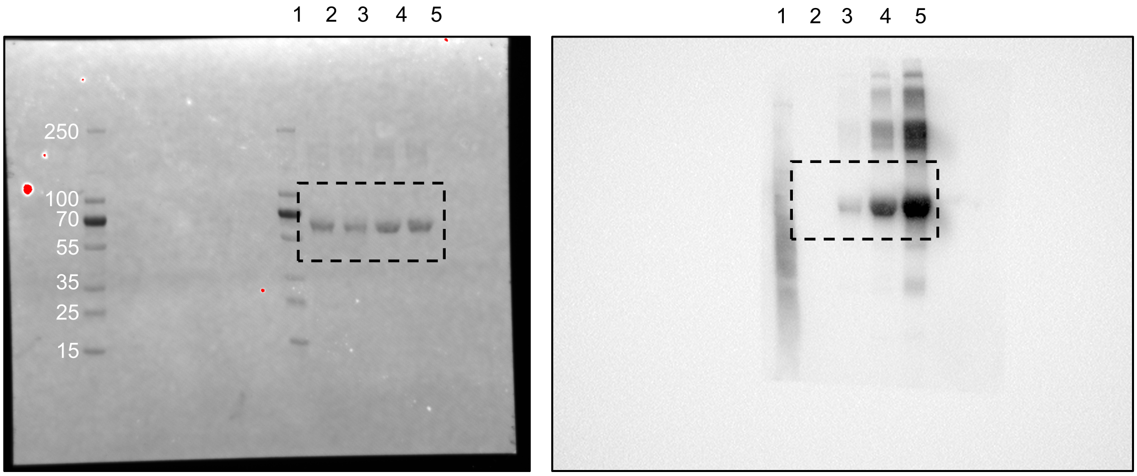

Carbonic anhydrase

(Colorimetric) | Total Protein | Strepavidin-HRP

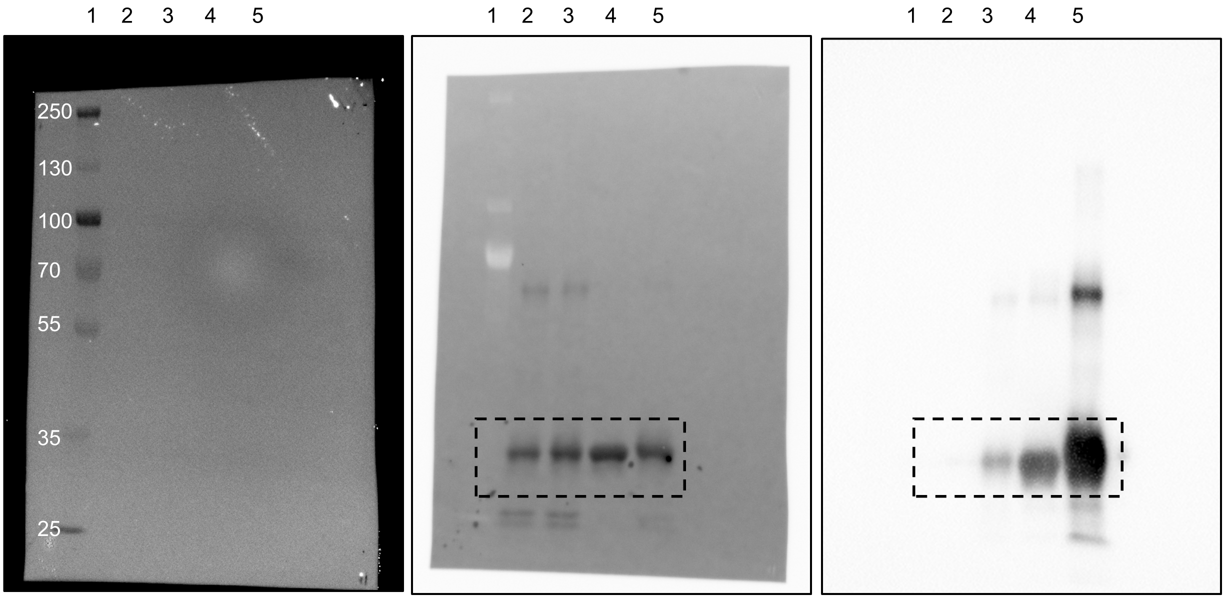

Extended Figure 2c

Total Protein

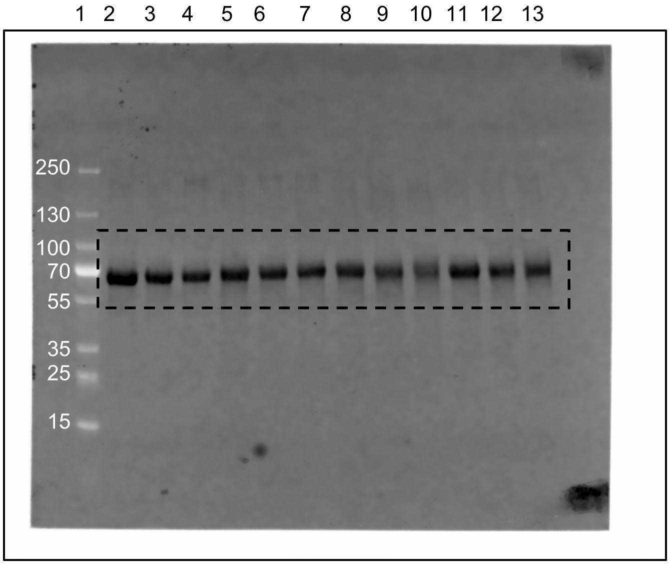

Strepavidin-HRP

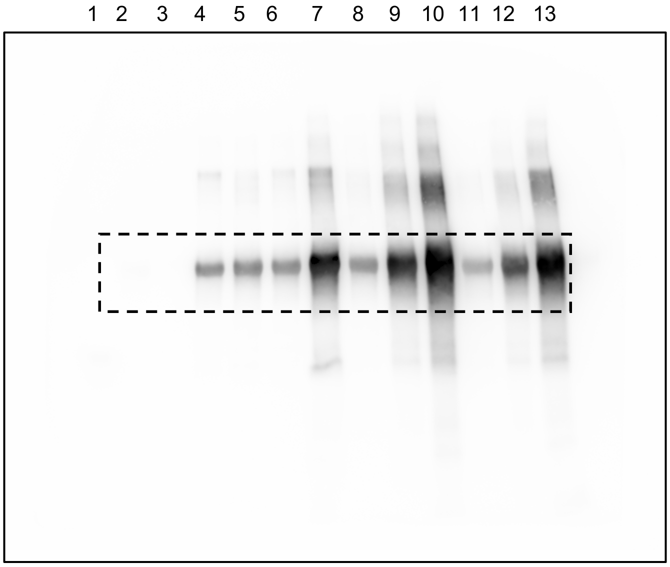

Supplement: Supplementary file 6 — Unprocessed western blots. [file 41557_2025_1931_MOESM6_ESM.pdf]
